# Exploring the antitumor potential of novel quinoline derivatives *via* tubulin polymerization inhibition in breast cancer; design, synthesis and molecular docking†

**DOI:** 10.1039/d4ra04371e

**Published:** 2024-07-12

**Authors:** Heba Abdelmegeed, Lina M. A. Abdel Ghany, Amira Youssef, Abd-Allah S. El-Etrawy, Noha Ryad

**Affiliations:** a Chemistry of Natural Compounds Department, Pharmaceutical and Drug Industries Research Institute, National Research Centre Giza 12622 Egypt; b Pharmaceutical Chemistry Department, College of Pharmaceutical Sciences and Drug Manufacturing, Misr University for Science and Technology (MUST) 6th of October City, P.O. Box 77 Giza Egypt Lina.ameen@must.edu.eg; c Pharmaceutical Organic Chemistry Department, College of Pharmaceutical Sciences and Drug Manufacturing, Misr University for Science and Technology (MUST) 6th of October City, P.O. Box 77 Giza Egypt; d Department of Chemistry, Basic Science, Misr University for Science and Technology (MUST) 6th of October City, P.O. Box 77 Giza Egypt

## Abstract

A series of quinoline derivatives was designed and synthesized as novel tubulin inhibitors targeting the colchicine binding site. All the rationalized compounds 3a–e, 4a–e, 5a–e, and 6a–e have been chosen for screening their cytotoxic activity against 60 cell lines by NCI. Compounds 3b, 3c, 4c, 5c and 6c demonstrated the most notable antitumor activity against almost all cell lines. Compound 4c emerged as the most potent compound as an antiproliferative agent. This compound was subsequently chosen for five-dose testing and it exhibited remarkable broad-spectrum efficacy with strong antitumor activity against several cell lines. Compound 4c significantly induced cell cycle arrest in MDA-MB-231 cells at G2 and M phases where the cell population increased dramatically to 22.84% compared to the untreated cells at 10.42%. It also increased the population in MDA-MB-231 cells at both early and late stages of apoptosis. Compound 4c can successfully inhibit tubulin polymerization with an IC_50_ value of 17 ± 0.3 μM. The β-tubulin mRNA levels were notably reduced in MDA-MB-231 cells treated with compound 4c which is similar to the effect observed with colchicine treatment. Docking studies revealed that compound 4c interacted well with crucial amino acids in the active site.

## Abstract 2

A series of quinoline derivatives was designed and synthesized as novel tubulin inhibitors targeting the colchicine binding site.

## Graphical abstract

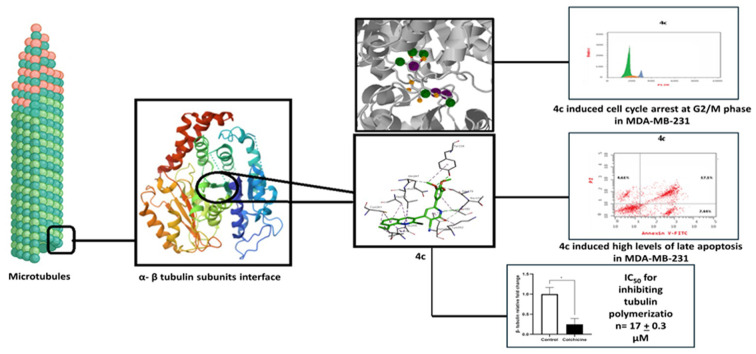

## 1. Introduction

Cancer is the development of abnormal cells that proliferate uncontrollably and affect human health and it is one of the leading causes of death globally.^1–3^ The latest data indicate that breast cancer is one of the most prevalently diagnosed cancers among women with an estimated 2.3 million new cases (11.7%) and the 5^th^ cause of cancer-related deaths with an estimated 6.9%.^1–3^ Currently, chemotherapy is the main approach for cancer treatment; since microtubules are essential for cell viability, especially for the fast division of cancer cells, drugs that interfere with the dynamics of microtubule/tubulin have become essential therapeutics.^4,5^ Most of these agents act by binding to the tubulin, an α/β heterodimer protein that forms the microtubule which is a major component of the eukaryotic cytoskeleton.^6^

**Fig. 1:**
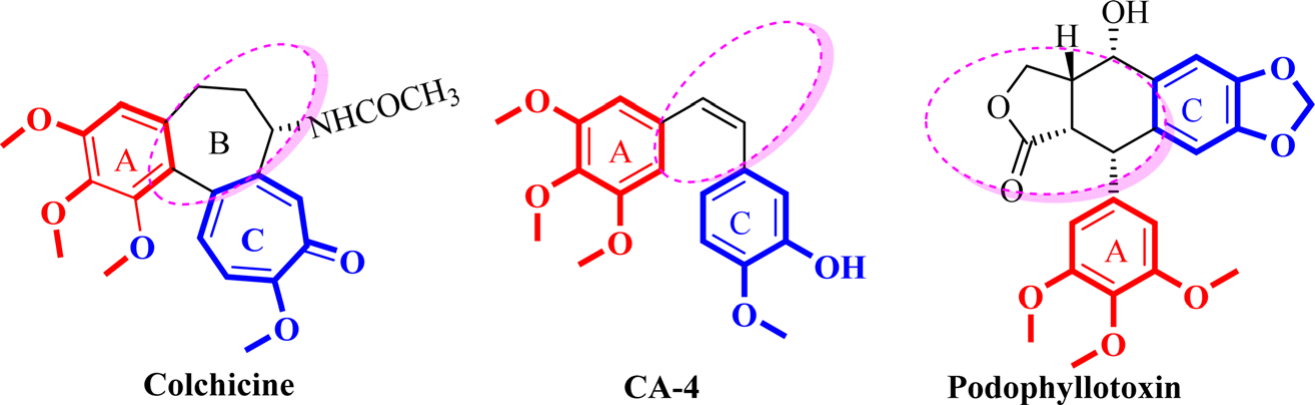
Structure of tubulin destabilizing agents.

Microtubule targeting agents (MTA) are also named antimitotic agents which bind to the tubulin in the microtubules and prohibit the proliferation of the cells. There are two distinct categories of microtubule targeting agents; those that bind to the binding site of paclitaxel, such as paclitaxel which are identified as microtubule stabilizing agents or tubulin promotors.^7^ Conversely, agents that bind to the binding site of colchicine, such as colchicine, combretastatin A-4 (CA-4), and podophyllotoxin or to the binding site of vinca alkaloid, such as vincristine, are identified as tubulin inhibitors or destabilizing agents (Fig. 1).^8^

The colchicine binding site (CBS), which sits at the interface between α and β-tubulin heterodimers, has been the subject of much research to identify potential anticancer drugs. Colchicine was the first tubulin destabilizing agent. It was extracted from the poisonous meadow saffron *Colchicum autumnale* and it binds with high affinity to β-tubulin subunit, interfering with microtubule polymerization and causing mitosis.^5,9,10^

**Fig. 2:**
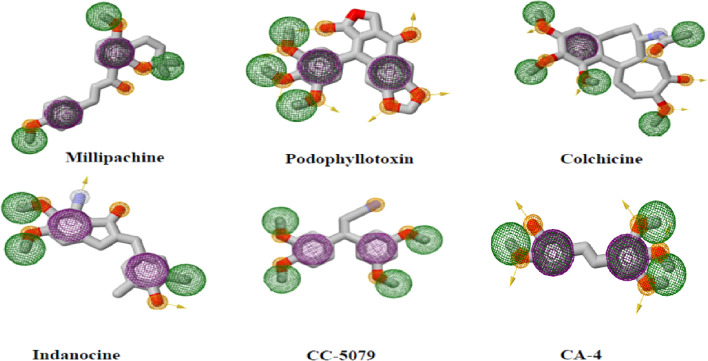
Two-dimensional structures of reported CBSI's with pharmacophoric features necessary to impart depolymerization upon tubulin where purple spheres represent aromatic pharmacophoric points (AR), the orange spheres with and without arrow denote the functional groups that act as hydrogen bond donor (HBD) and acceptor (HBA), respectively. Finally green spheres represent hydrophobic centers (HY).

Pharmacophoric patterns identified in studies of colchicine binding site inhibitors (CBSIs) consistently displayed specific structural characteristics and recurring interactions between tubulin and ligands at pharmacophoric points. These studies proposed that the different structural classes of CBSIs can be linked by a six to seven-point pharmacophore consisting of three hydrogen bond acceptors (HBA), one hydrogen bond donor (HBD), one or two hydrophobic center (HY), and/or one planar group.^11^ Ten top-scored hypothetical pharmacophores were generated in another study using the HypoGen algorithm, the training set consisted of 26 drugs with tubulin inhibitory activity ranging from 0.52 to 13.800 nM. One hydrogen-bond acceptor (HBA), one hydrogen-bond donor (HBD), one ring aromatic feature (RA), one hydrophobic feature (HY), and three excluded volumes (EV) made up the greatest hypothetical pharmacophore.^12^ Another training set for the 3D QSAR pharmacophore model used the structures of 21 different drugs defined four different feature types: the hydrophobic feature (HY), hydrophobic aromatic group (HY-AR), hydrogen bond acceptor (HBA), and hydrogen bond donor (HBD) (Fig. 2).^13–15^ The previously mentioned features are necessary to ensure stable and effective binding to the CBS.^16^ The presence of many hydrophobic and aromatic groups ensures a fit to the hydrophobic pocket of the β-tubulin subunit, while the HBA and HBD groups extend the inhibitors towards the hydrophilic α-tubulin subunit.

SAR analysis of colchicine revealed that the important trimethoxyphenyl group is oriented within β-tubulin. The interaction between ring A and CBS provides the strength of colchicine binding to tubulin. On the other hand, interactions between the CBS and the oxygen atoms on ring C control the inhibition. It is suggested that within the binding locus, ring A anchors and keeps the B and C rings oriented correctly.^5,9,10^

**Fig. 3:**
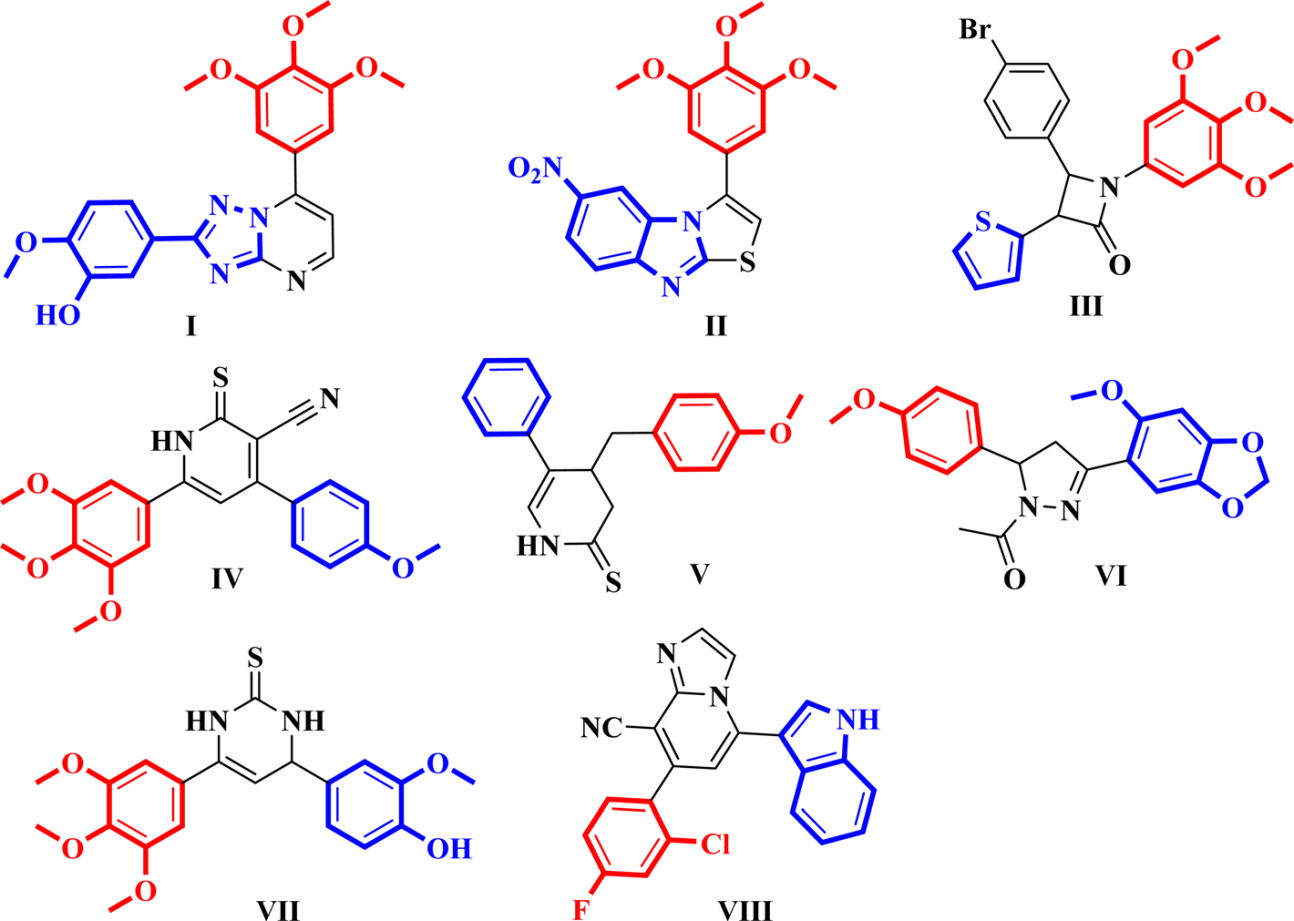
Chemical structure of reported CA-4 analogs with cytotoxic activity.

Moreover, colchicine showed its anticancer effect on apoptotic genes of human breast cancer cell lines.^10,17,18^CA-4 binds at the CBS in a manner akin to that of colchicine.^10^ Nevertheless, the instability of the *cis*-double bond, which might change into an inactive *trans*-conformation, has hindered the development of CA-4.^19^ In recent years, various conformationally restricted analogs of CA-4 and colchicine-bearing triazolopyrimidine I, benzimidazole II, azetidine III, pyridine IV and VI, pyrazole V, pyrimidine VII, imidazopyridine VIII have been reported as potent tubulin inhibitors (Fig. 3).^20–26^

**Fig. 4:**
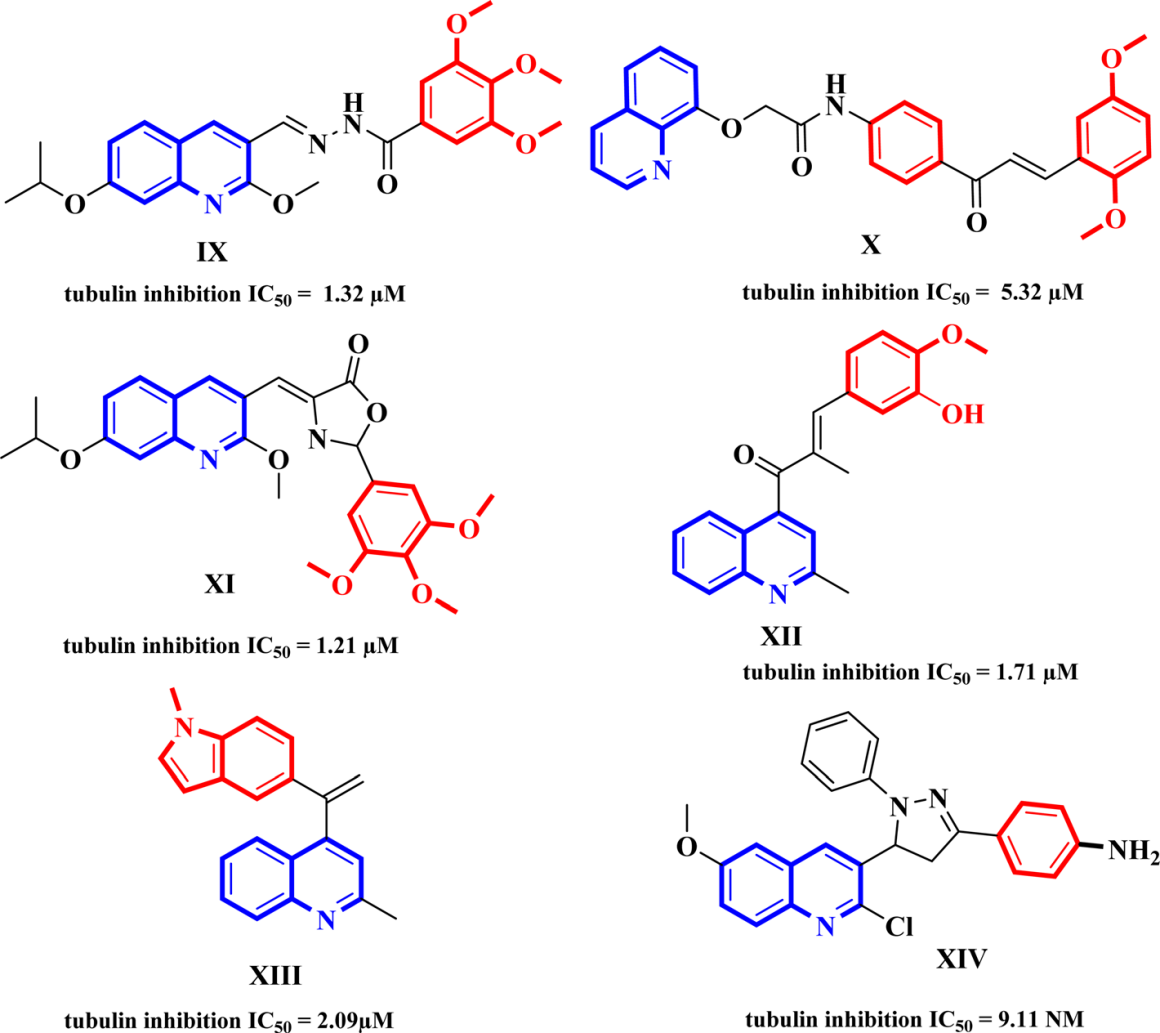
Some quinoline-containing antimitotic agents and tubulin polymerization inhibitors.

Quinoline is a privileged scaffold in the expansion of anticancer drugs as they have exhibited potent antiproliferative activity *via* different mechanisms of action including cell cycle arrest, induction of apoptosis, and inhibition of angiogenesis, and cell migration disruption. Many tubulin inhibitors IX–XIV also, possess quinoline motifs (Fig. 4), these compounds exhibited their antiproliferative activity because of tubulin polymerization inhibition and the disruption of microtubule assembly.^27–32^

**Fig. 5:**
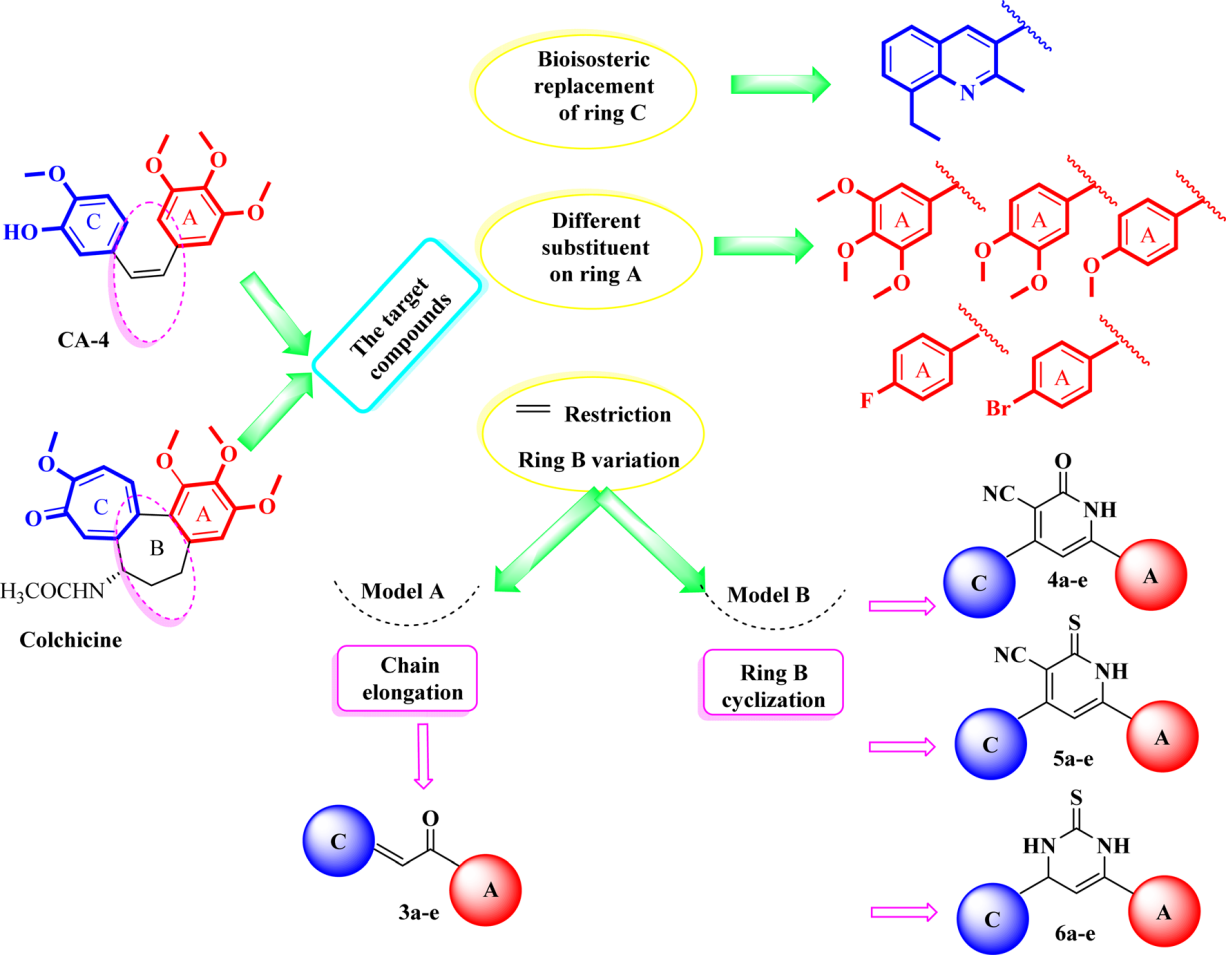
Design strategy of the rationalized compounds 3a–e, 4a–e, 5a–e, and 6a–e.

**Scheme 1:**
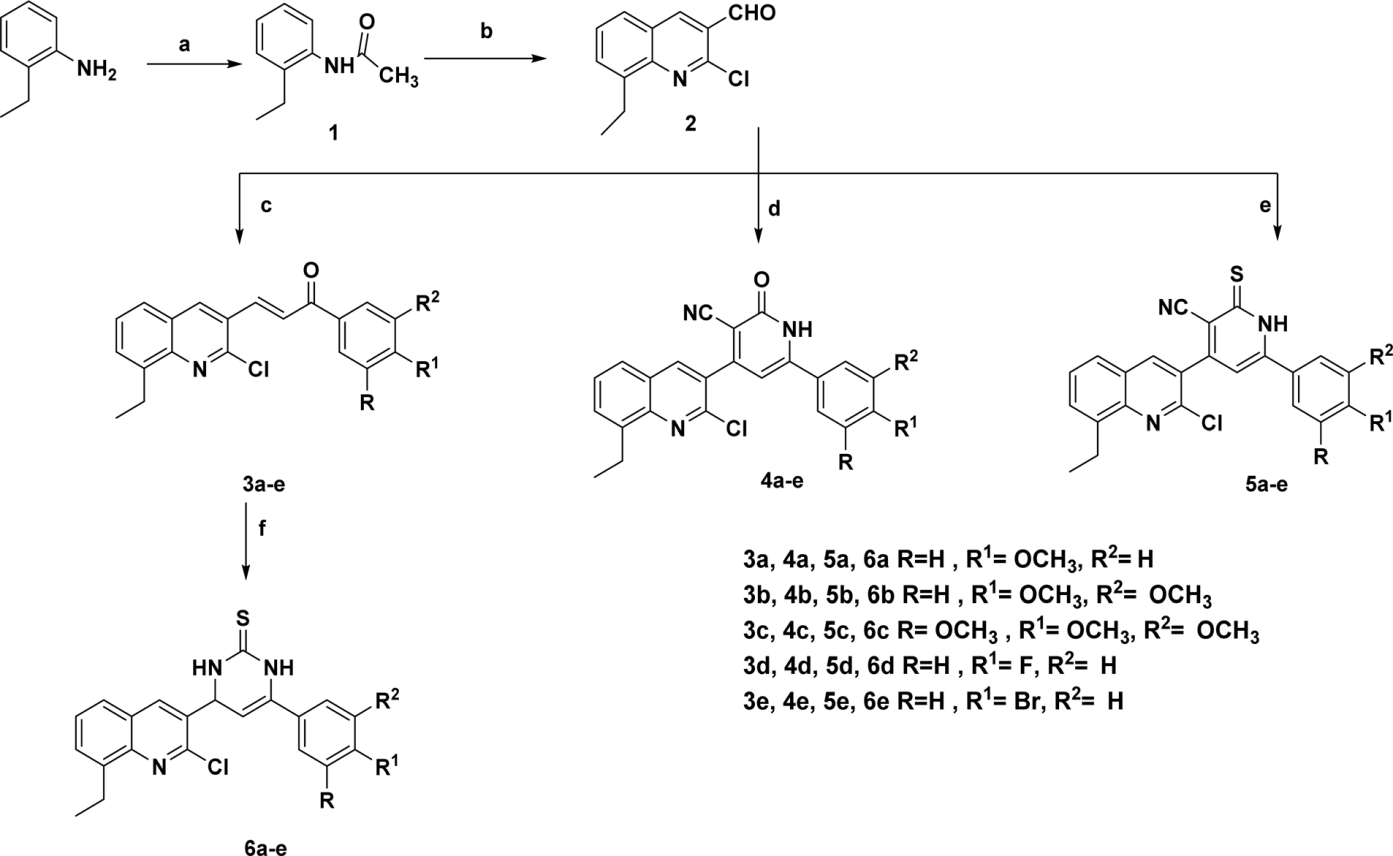
Synthesis of the target compounds 3a–e, 4a–e, 5a–e, and 6a–e.

**Fig. 6:**
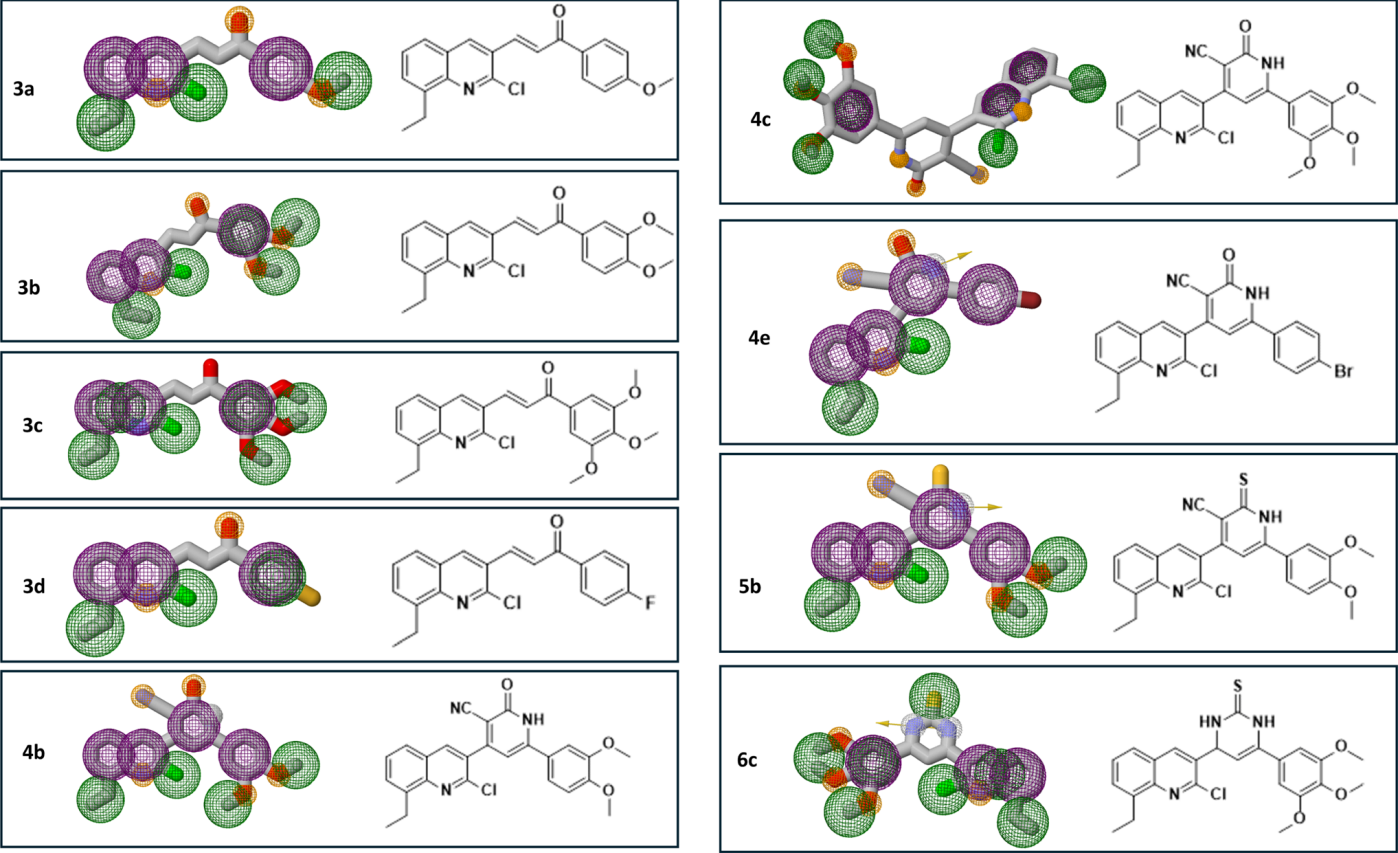
Some of the designed titled compounds with common structural features to reported tubulin depolymerization agents.

In light of the aforementioned findings, we aimed to synthesize novel molecules with promising tubulin polymerization inhibition activity *via* structure optimization of lead compounds CA-4 and colchicine. As shown in our design strategy (Fig. 5), bioisosteric replacement of ring C in colchicine and CA-4 was performed with a quinoline ring. 3,4,5-Trimethoxyphenyl moiety (ring A) in colchicine and CA-4 was retained in certain synthesized derivatives, while others were embellished with 3,4-dimethoxyphenyl, 4-methoxyphenyl, 4-fluorophenyl or 4-bromophenyl moieties. Chain elongation in compounds 3a–e by the introduction of 2-propen-1-one instead of an olefinic bond aimed to increase the rigidity of the structure to overcome the drawbacks of CA-4. For more rigidification, cyclization with rigid core ring B was performed by 2-pyridinone 4a–e, pyridinethione 5a–e and pyrimidinethione ring 6a–e. Our designed plan was achieved according to Scheme 1 (Fig. 6). The novel compounds were also fully characterized for their cytotoxic activity on NCI 60-cell lines and additional mechanistic biochemical tests were conducted for the molecule with the highest potency. Moreover, docking studies were conducted in an attempt to interpret the outcomes of biological tests.

## 2. Results and discussion

### 2.1. Chemistry

The present study described the synthetic plan of novel compounds 3a–e, 4a–e, 5a–e, and 6a–e as illustrated in Scheme 1. Compound 1 and the key intermediate 2 were prepared in excellent yields as described in the reported literature.^33–36^ Chalcone derivatives 3a–e were prepared following the Claisen–Schmidt condensation reaction by reacting a mixture of the carbaldehyde derivative 2 with the appropriate substituted acetophenone in the presence of 50% aqueous potassium hydroxide. The IR spectrum of each synthesized compound exhibited a strong absorption band corresponding to the carbonyl (C=O) functional group, which was observed in the range of 1600–1656 cm^−1^. ^1^H NMR charts showed signals corresponding to α, β alkene protons, as a doublet signal at *δ* 8.12–8.20 ppm referred to the α-CH alkene proton while another doublet appeared at *δ* 7.64–8.11 ppm corresponding to β-CH alkene proton. On the other hand, their ^13^C NMR spectra showed a peak referring to the carbonyl (C=O) functional group at *δ* 186.8–188.0 ppm. Refluxing the 8-ethylquinoline-3-carbaldehyde derivative 2 with different acetophenones and either ethyl cyanoacetate or 2-cyanothioacetamide in the presence of ammonium acetate afforded the target pyridine derivatives 4a–e and 5a–e, respectively. The IR spectra of the carbonitrile derivatives 4a–e and 5a–e showed a peak at 2218–2239 cm^−1^ of the added CN group and a band of NH appeared at 3398–3433 cm^−1^. ^1^H NMR spectra of 2-oxo-1,2-dihydropyridine derivatives 4a–e showed a singlet at *δ* 6.85–7.16 ppm corresponding to the proton of 1,2-dihydropyridine ring, while ^13^C NMR spectra revealed signals at *δ* 116.4–118.2 and 156.0–159.6 ppm for nitrile and carbonyl groups, respectively. ^1^H NMR spectra of the isosteric analog; 2-thioxo-1,2-dihydropyridine derivatives 5a–e showed similar singlet at *δ* 6.78–7.17 ppm corresponding to the proton of 1,2-dihydropyridine ring, although, their ^13^C NMR spectra revealed signals at *δ* 116.0–117.0 and 156.5–162.1 ppm for nitrile and thiocarbonyl groups, respectively. Additionally, mass spectrometric analysis was conducted to further confirm the structure of the synthesized 1,2-dihydropyridine derivative compounds, designated as 4a–e and 5a–e. The mass spectrometric data showed the presence of molecular ion peaks that corresponded to the expected molecular weights of the respective synthesized compounds. 3,4-Dihydropyrimidinethione derivatives 6a–e were synthesized by refluxing the corresponding chalcone 3a–e with thiourea in an alkaline medium. The afforded compounds were prepared with an overall good yield of 70–74%. The IR spectra confirmed that the carbonyl functional group present in the starting materials 3a–e was no longer detected in the final synthesized compounds but an absorption band appeared at 3162–3169 cm^−1^ corresponding to NH. ^1^H NMR of 6a–e showed two doublets at *δ* 5.39–5.61 ppm which is characteristic to the dihydropyrimidine ring protons along with the exchangeable protons of 2NH that appeared at *δ* 9.10–9.10 and 10.04–10.20 ppm. Additionally, ^13^C NMR showed a peak at *δ* 96.5–99.7 ppm pointing to C5 of the pyrimidine ring and a peak at *δ* 174.0–177.0 ppm referring to C=S. Finally, it is worth mentioning that all our novel compounds were subjected to elemental analyses for further authentication of the synthesized compounds.

### 2.2. Biology

#### 2.2.1. *In vitro* cytotoxic activity screening

**Fig. 7:**
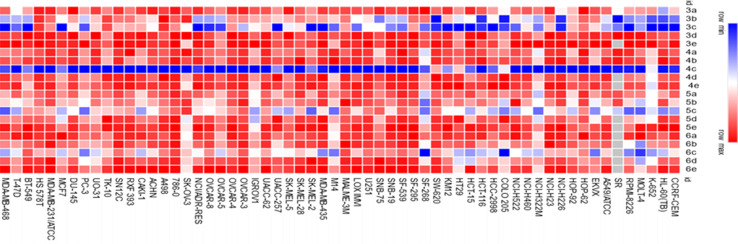
Heatmap of all the synthesized quinoline derivatives 3a–e, 4a–e, 5a–e and 6a–e demonstrating their effect on tumor cells viability of 60 different cancer cell lines. Red color indicates higher tumor cells viability, while blue color indicates less tumor cells viability.

In order to determine the cytotoxic activity of 20 recently synthesized quinoline derivatives, their activity against a comprehensive panel of 60 cancer cell lines, including those representing leukemia, melanoma, lung, colon, central nervous system (CNS), ovary, kidney, prostate, and breast cancers, was screened at the National Cancer Institute (NCI) in Bethesda, MD, USA. This screening involved administering a single dose of 10 μM and the data were reported as percent growth (*G*%) of the treated cell lines (Fig. S68–S87†) and then expressed as percentage growth inhibition (GI%) (Tables S1 and S2†). The heatmap shown in Fig. 7 illustrates the anti-proliferative activity (cells viability percentage) of all quinoline derivatives tested against all 60 cancer cell lines. Based on the findings, the majority of the newly created compounds exhibited cytotoxic effects ranging from moderate to high, as indicated by their growth inhibition percentages (GI%). Some of the compounds demonstrated only mild cytotoxic activity. Chalcone derivative 3a (methoxy-substituted) showed selective remarkable cytotoxic activity on non-small cell lung cancer (NCI-H226) with GI value of 79.88%. Chalcone derivative 3b (dimethoxy-substituted) demonstrated potent cytotoxic activity against leukemia (SR), CNS cancer (SF-539) with GI value of 80.08 and 99.38%, respectively. Additionally, it showed lethal effect on non-small cell lung cancer (NCI-H226) and CNS cancer (SNB-75). Chalcone derivative 3c (trimethoxy-substituted) displayed potent antitumor activity against leukemia (CCRF-CEM) with GI value of 87.78%, non-small cell lung cancer (NCI-H522) with GI values of 80.68%, colon cancer (KM12 and HT29) with GI value of 80.66 and 77.4%, respectively and ovarian cancer (OVCAR-8) with GI value of 84%. Other chalcones 3d (fluoro-substituted) and 3e (bromo-substituted) exhibited low cytotoxic activity across all tested cell lines.

Pyridin-2-one 4c (trimethoxy-substituted) demonstrated the highest level of cytotoxic activity among the synthesized compounds. It possessed potent cytotoxic activity against wide range of tumor cells as leukemia (CCRF-CEM, MOLT-4, RPMI-8226, and SR) with GI values of 80.39, 76.43, 74.51 and 77.32%, respectively, non-small cell lung cancer (A549/ATCC and HOP-62) with GI values of 88.16 and 87.86%, respectively, CNS cancer (SF-295 and SNB-19) with GI values of 96.38 and 89.58%, respectively, melanoma (LOX IMVI, MALME-3M and SK-MEL-5) with GI values of 91.58, 89.41, 74.51 and 78.58%, respectively, ovarian cancer (OVCAR-8) with GI value of 92.11%, renal cancer (ACHN and CAKI-1) with GI values of 75.69 and 95.06%, respectively, prostate cancer (DU-145) with GI value of 80.55% and breast cancer (T-47D) with GI value of 91.56%. Its lethal effect was evident across a wide range of tumor cells including non-small cell lung cancer (HOP-92, NCI-H226 and NCI-H522), CNS cancer (SF-539, SNB-75 and U251), melanoma (UACC-62), ovarian cancer (OVCAR-3 and OVCAR-4), renal cancer (786-0, A498, RXF 393, SN12C, TK-10 and UO-31) and breast cancer (MDA-MB-231/ATCC and HS 578T). Other derivatives in the series including pyridin-2-one derivative 4a (methoxy-substituted) showed moderate activity over CNS cancer (SNB-75) with GI value of 54.69%, where, compound 4b (dimethoxy-substituted), 4d (fluoro-substituted) and 4e (bromo-substituted) possessed mild cytotoxic activity over multiple cell lines.

Pyridinethione derivative 5c displayed significant cytotoxic activity on non-small cell lung cancer (HOP-92) with GI value of 77.38%, while, its moderate cytotoxic activity appeared over numerous cell lines as leukemia (CCRF-CEM, HL-60(TB), MOLT-4 and RPMI-8226) with GI values of 57.63, 51.76, 61.16 and 63.06%, respectively, non-small cell lung cancer (NCI-H522) with GI value of 65.68%, renal cancer (CAKI-1) with GI value of 69.81%, and breast cancer (MDA-MB-231/ATCC, T-47D and MDA-MB-468) with GI values of 52.51, 60.59 and 50.55%, respectively. Additional derivatives bearing pyridinethione namely, 5a (methoxy-substituted), 5b (dimethoxy-substituted), 5d (fluoro-substituted) and 5e (bromo-substituted) showed a slight cytotoxic activity over multiple cell lines.

Finally, pyrimidine derivative 6c (trimethoxy-substituted) possessed moderate cytotoxic effect over leukemia (MOLT-4 and RPMI-8226) with GI values of 67.65 and 57.59%, respectively, non-small cell lung cancer (NCI-H226 and NCI-H522) with GI values of 57.97 and 50.3%, respectively and renal cancer (CAKI-1 and UO-31) with GI values of 64.11 and 67.70%, respectively. Pyrimidine derivative 6b (dimethoxy-substituted) possessed moderate cytotoxic effect over leukemia (MOLT-4) with GI value of 53.3%. Other derivatives bearing pyrimidine moiety specially, 6a (methoxy-substituted), 6d (fluoro-substituted) and 6e (bromo-substituted) showed limited cytotoxic activity over multiple cell lines.

In summary, upon comparing all the newly synthesized compounds (Fig. 7), clearly shows that 4c > 3c > 3b > 5c > 6c derivatives demonstrated the highest antitumor activity against almost all cell lines. Notably, compound 4c being the most potent compound as anti-proliferative agent. Interestingly, 4c derivative along with the other compounds with the highest activity (*i.e.*, 3c, 5c, 6c) belonged to the c derivatives series which is characterized by trimethoxy group substitution in the main ring. Hence, the trimethoxy group substitution is shown to be superior to all other modifications in the main ring (methoxy, dimethoxy, fluorine, and bromine). On the other hand, cyclization with pyridine-2-one cyclization is more effective than (open chain > pyridinethione > pyrimidine). This suggests that trimethoxy group and pyridin-2-one are responsible for the compounds' high antitumor activity.

#### 2.2.2. Five doses testing of compound 4c

**Fig. 8:**
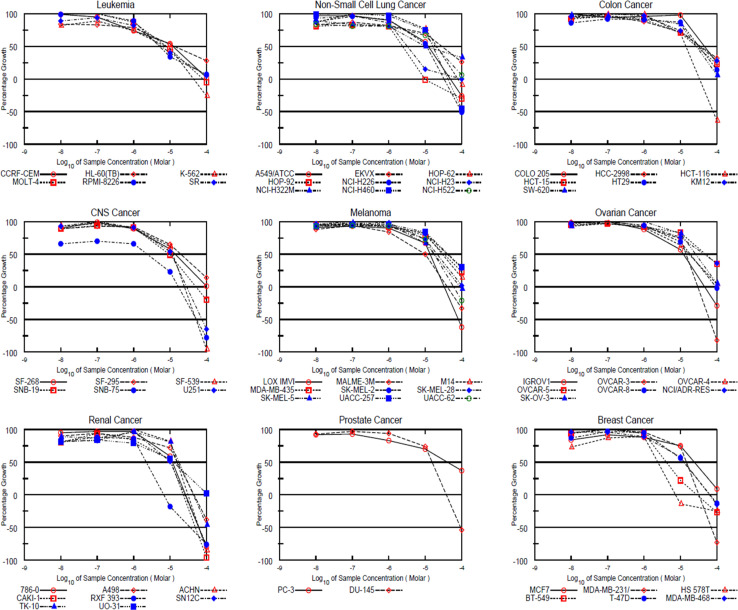
Dose response curves of 4c derivative against all 60 cell lines.

Since pyridin-2-one derivative 4c exhibited the highest level of cytotoxic activity against almost all tumor cell lines, it has progressed to the full 5-dose assay for the determination of median growth inhibitory (GI_50_, μM), total growth inhibitory (TGI, μM) and median lethal (LC_50_) concentrations (Table 1). The dose response curves of 4c derivative against all different cancer cell lines are shown in (Fig. 8). Compound 4c showed remarkable cytotoxic activity across various cell lines. In particular, it demonstrated notable activity on leukemia (K-562, MOLT-4, RPMI-8226 and SR) with GI_50_ values of 7.72, 8.17, 5.16 and 5.70 μM, respectively, non-small cell lung cancer (HOP-92 and NCI-H23) with GI_50_ values of 2.37 and 3.20 μM, respectively, CNS cancer (SNB-75) with GI_50_ value of 2.38 μM, renal cancer (RXF 393) with GI_50_ value of 2.21 μM and breast cancer (HS 578T, BT-549) with GI_50_ values of 2.38 and 4.11 μM, respectively. Additionally, compound 4c also disclosed moderate cytotoxic activity across leukemia (CCRF-CEM and HL-60(TB)) with GI_50_ values of, 12.00 and 13.40 μM, respectively, non-small cell lung cancer (A549/ATCC and NCI-H226, NCI-H322M) with GI_50_ values of 12.20, 10.90 and 10.50 μM, respectively, colon cancer (HCT-116) with GI_50_ value of 14.10 μM, CNS cancer (SF-268, SF-539, SNB-19, U251) with GI_50_ values of 13.80, 12.00, 9.25 and 10.80 μM, respectively, ovarian cancer (IGROV1 and OVCAR-3) with GI_50_ values of 12.20 and 14.20 μM, respectively, renal cancer (786-0, CAKI-1, SN12C and UO-31) with GI_50_ values of 11.70, 10.70, 10.30, and 12.60 μM, respectively, and breast cancer (HS 578T) with GI_50_ value of 14.50 μM.

**Table 1:** Median growth inhibitory (GI_50_, μM), total growth inhibitory (TGI, μM) and median lethal (LC_50_) concentrations of compound 4c

Subpanel tumor cell lines	Activity			Subpanel tumor cell lines	Activity		
	GI_50_	TGI	LC_50_		GI_50_	TGI	LC_50_
**Leukemia**				**Melanoma**			
CCRF-CEM	12.00	>100	>100	M14	29.70	>100	>100
HL-60(TB)	13.40	>100	>100	MDA-MB-435	32.60	>100	>100
K-562	7.72	43.80	>100	SK-MEL-2	39.80	>100	>100
MOLT-4	8.17	79.90	>100	SK-MEL-28	21.10	>100	>100
RPMI-8226	5.16	>100	>100	SK-MEL-5	17.50	90.20	>100
SR	5.70	>100	>100	UACC-257	42.60	>100	>100
				UACC-62	17.50	60.10	>100
**Non-small cell lung cancer**							
A549/ATCC	12.20	48.70	>100	**Ovarian cancer**			
EKVX	28.60	>100	>100	IGROV1	12.20	46.30	>100
HOP-62	20.70	78.90	>100	OVCAR-3	14.20	29.80	62.30
HOP-92	2.37	9.68	>100	OVCAR-4	17.20	>100	>100
NCI-H226	10.90	32.60	97.70	OVCAR-5	48.40	>100	>100
NCI-H23	3.20	>100	>100	OVCAR-8	18.50	93.60	>100
NCI-H322M	10.50	>100	>100	NCI/ADR-RES	44.90	>100	>100
NCI-H460	16.10	41.80	>100	SK-OV-3	26.00	>100	>100
NCI-H522	19.10	>100	>100				
				**Renal cancer**			
**Colon cancer**				786-0	11.70	26.70	61.10
COLO 205	45.60	>100	>100	A498	15.80	45.20	>100
HCC-2998	35.70	>100	>100	ACHN	15.60	31.00	61.80
HCT-116	14.10	33.30	78.40	CAKI-1	10.70	22.90	49.20
HCT-15	27.60	>100	>100	RXF 393	2.21	6.69	35.40
HT29	32.10	>100	>100	SN12C	10.30	25.20	61.80
KM12	33.60	>100	>100	TK-10	17.50	34.50	>100
SW-620	27.50	>100	>100	UO-31	12.60	>100	>100
	
**CNS cancer**				**Prostate cancer**			
SF-268	13.80	>100	>100	PC-3	40.30	>100	>100
SF-295	19.40	>100	>100	DU-145	15.50	38.00	93.50
SF-539	12.00	24.80	51.30				
SNB-19	9.25	50.90	>100	**Breast cancer**			
SNB-75	2.38	16.90	52.60	MCF7	24.00	>100	>100
U251	10.80	28.40	74.90	MDA-MB-231/ATCC	14.50	31.80	69.80
				HS 578T	2.38	7.26	>100
**Melanoma**				BT-549	4.11	28.10	>100
LOX IMVI	13.50	33.20	81.30	T-47D	12.20	64.10	>100
MALME-3M	10.10	40.00	>100	MDA-MB-468	12.70	61.60	>100

#### 2.2.3. Cell cycle analysis by flow cytometry

Cell cycle is a well-coordinated cellular division process which is composed of several phases. The mitotic phase (M phase) is a central phase which is characterized by the segregation of the duplicate DNA content into two daughter cells.^37^ Accurate spindle assembly is required for normal cell division and interfering with the formation of microtubules, which is composed of α-tubulin and β-tubulin subunits, hinders cell division leading to cell cycle arrest.^38^ Therefore, several tubulin polymerization inhibitors have been developed.^39^ Since we developed our quinoline derivatives to act as tubulin polymerization inhibitors, their ability to induce cell cycle arrest at G2 and M phases was evaluated. Hence, we focused on elucidating the cellular mechanisms underlying its potent cytotoxic activity against MDA-MB-231. Breast cancer cell lines are among the tumor cells against which pyridin-2-one derivative 4c showed high cytotoxic activity. Breast cancer is one of the most diagnosed cancers among women with 2.26 million new cases in 2020.^40^ It has several subtypes such as luminal breast cancer, Her-2/neu positive type, and triple-negative breast cancer.^41^ MDA-MB-231 cell line is one example of the cell lines used to study triple-negative breast cancer. It is used to identify pathways that regulate metastasis when used for xenografts.^42^ Therefore, MDA-MB-231 cell line is considered a valuable tool for developing new antiproliferative drugs against metastatic breast cancer.^43^

**Fig. 9:**
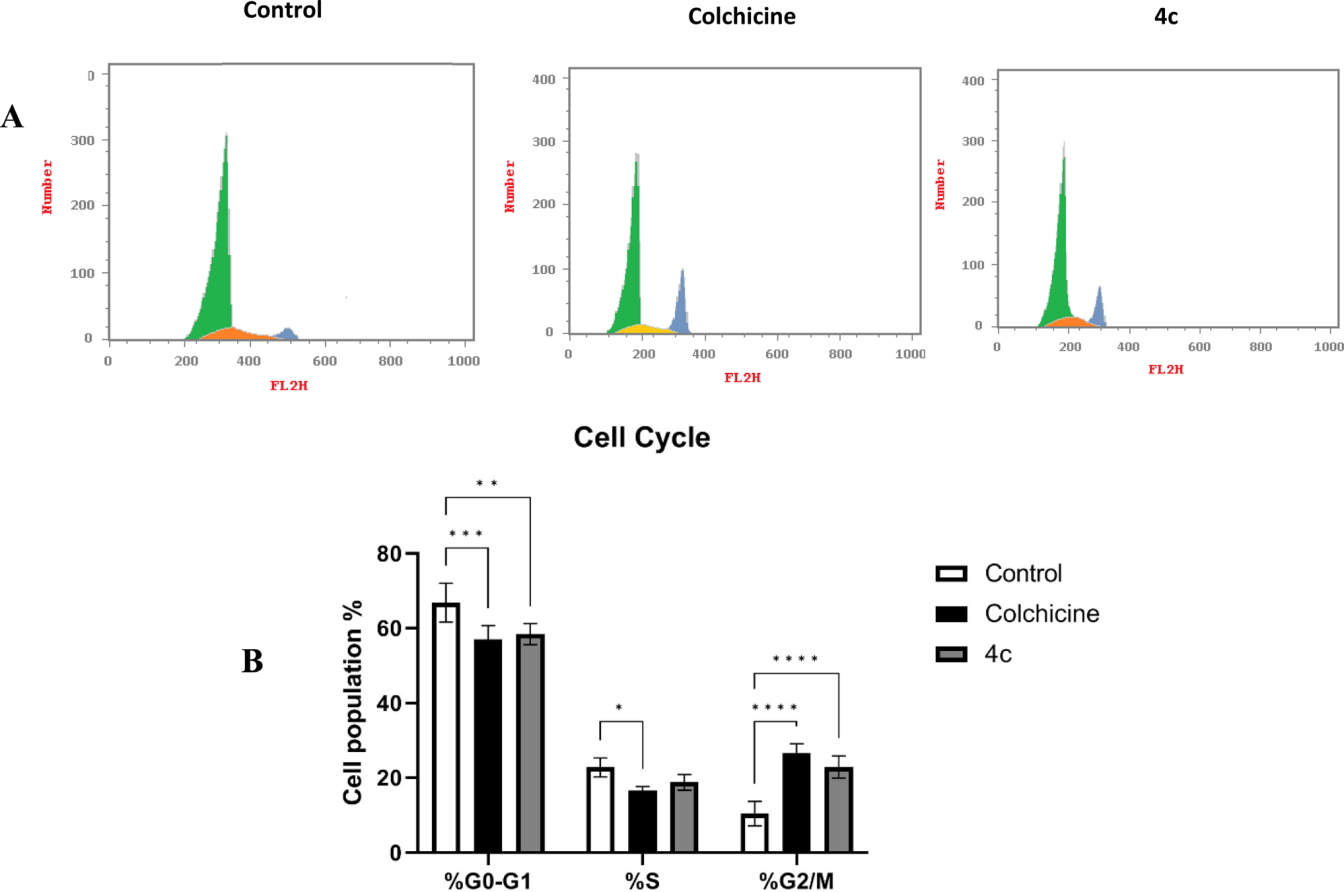
(A) Histograms of cell cycle phase distribution of control, colchicine, and 4c treated cells using PI staining for FACS analysis. (B) Percentages of cells accumulation at G0–G1, S, and G2/M cell cycle phases induced by control, colchicine, and 4c. Data are represented as the mean ± SD of three independent experiments. Statistical analysis was conducted using two-way ANOVA followed by Tukey's multiple comparison test; *p < 0.05, **p < 0.01, ***p < 0.001, ****p < 0.0001 compared to the control.

Hence, we investigated the distribution of cells at the different phases of cell cycle after MDA-MB-231 cells treatment with the newly synthesized 4c derivative and the positive control colchicine which is a well-established tubulin polymerization inhibitor. Fig. 9A shows the histograms of the control non-treated cells in addition to 4c, and colchicine-treated cells demonstrating propidium iodide PI signals at each phase. Both 4c and colchicine histograms showed an increase in signal intensity at G2/M phase. Moreover, the distribution of cells in each phase of the cell cycle was measured. As illustrated in Fig. 9B, 4c and colchicine significantly induced cell cycle arrest at G2 and M phases where the cells population increased dramatically to 22.84% and 26.51%, respectively compared with control non-treated cells which were 10.42%. There was no significant difference between 4c and colchicine in inducing cell cycle arrest at G2 and M phases. Hence, 4c derivative exerts its cytotoxic action against breast cancer *via* inhibiting tubulin polymerization causing cell cycle arrest at G2 and M phases.

#### 2.2.4. Cell apoptosis analysis

**Fig. 10:**
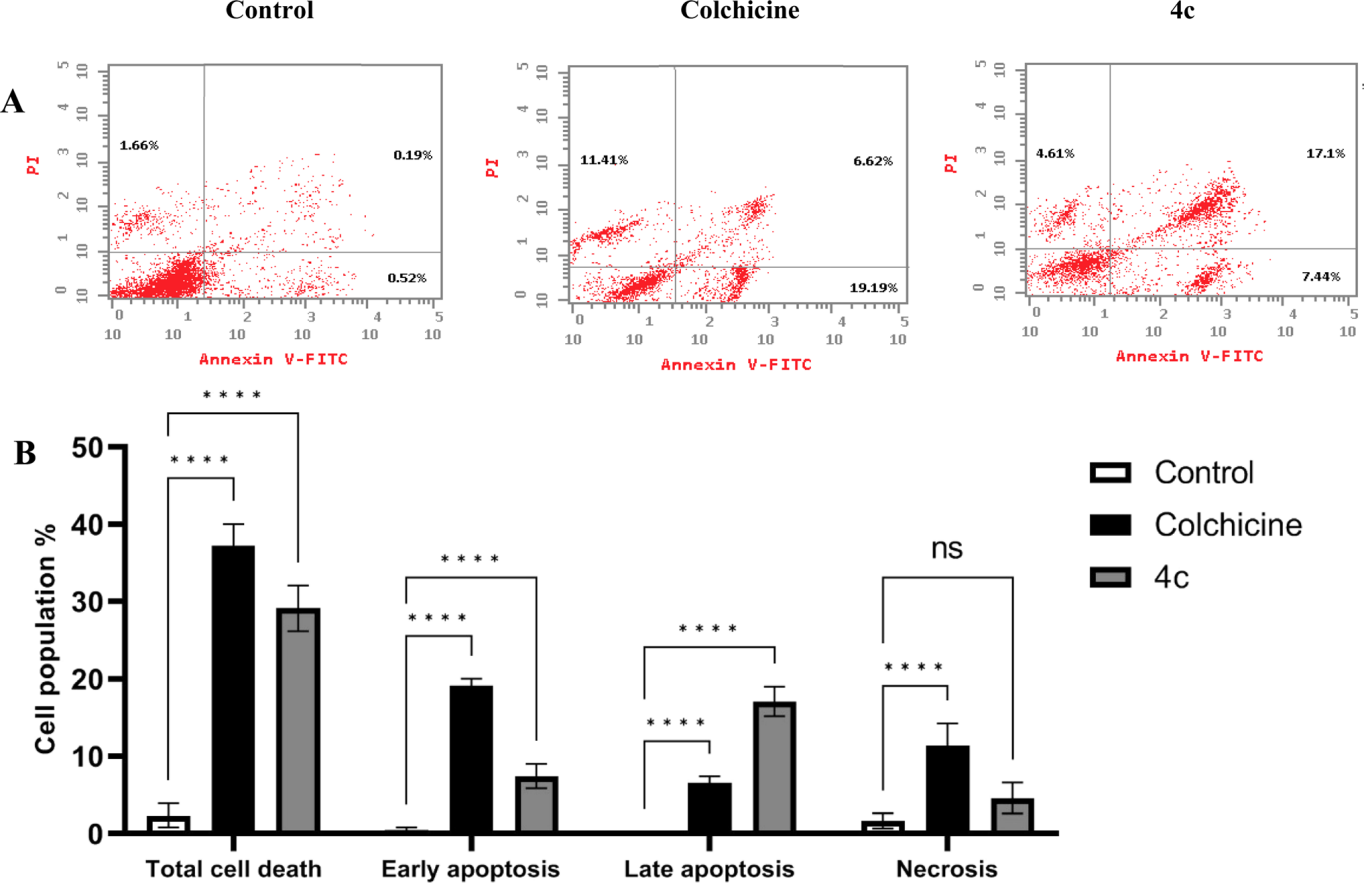
(A) Dot blots of apoptotic cells populations using PI/annexin V-FITC staining for FACS analysis. (B) Percentages of early apoptosis, late apoptosis and necrosis induced by 4c and colchicine in MDA-MB-231 cells compared with non-treated control cells. Data are represented as mean ± SD of three individual experiments. Statistical analysis was done by applying either two-way ANOVA followed by with Dunnett's multiple comparison test; *p < 0.05, **p < 0.01, ***p < 0.001, ****p < 0.0001 compared to the control.

Tubulin polymerization inhibitors can trigger cellular apoptosis due to cell cycle progression hindrance at M phase and the accumulation of mitotic cells.^44^ Hence, we examined the ability of compound 4c to induce cellular apoptosis in MDA-MB-231 cells. Following the reported protocol by staining MDA-MB-231 cells stained with both PI and annexin V-FITC. Fig. 10A shows the dot blots of the control non-treated cells, 4c and colchicine treated cells. The percentages of apoptotic cells significantly increased after treatment with 4c and colchicine treatment compared with non-treated cells. Both 4c and colchicine significantly increased the percentages of cells at both early and late apoptosis compared with the control. Table S3† shows the percentages of the cells at each apoptotic phase. As displayed in (Fig. 10B) colchicine significantly induced high levels of early apoptosis, while 4c induced higher levels of late apoptosis. This difference indicates that 4c acts with a faster rate in promoting apoptosis, thus reaching the late apoptosis phase earlier compared with colchicine. Additionally, there was no significant difference in necrosis levels between 4c treated and non-treated cells. This indicates the higher safety levels of compound 4c compared with colchicine which significantly increased necrosis levels to 11.41%. This is in alignment with other studies that shows the ability of tubulin polymerization inhibitors to cause apoptosis.^45,46^

#### 2.2.5. Tubulin polymerization inhibition assay

To verify whether the newly synthesized compound 4c can successfully inhibit tubulin polymerization, the enzymatic IC_50_ for inhibiting tubulin polymerization was measured. Table 2 shows the IC_50_ for tubulin polymerization inhibition of compound 4c, colchicine, and combretastatin also, graphs of tubulin polymerization inhibition assay data are represented in Fig. S88 and S90.† All compounds were able to inhibit tubulin polymerization with CA-4 being the most active with the lowest IC_50_. This is in alignment with several studies that showed the ability of different quinoline derivatives to inhibit tubulin polymerization.^28,47,48^

**Table 2:** IC_50_ values of 4c, colchicine and CA-4 for tubulin polymerization inhibition

Compound	IC_50_ (μM) for tubulin polymerization inhibition
4c	17 ± 0.3
Colchicine	7.48 ± 0.11
CA-4	4.647 ± 0.06

#### 2.2.6. Tubulin expression assay

**Fig. 11:**
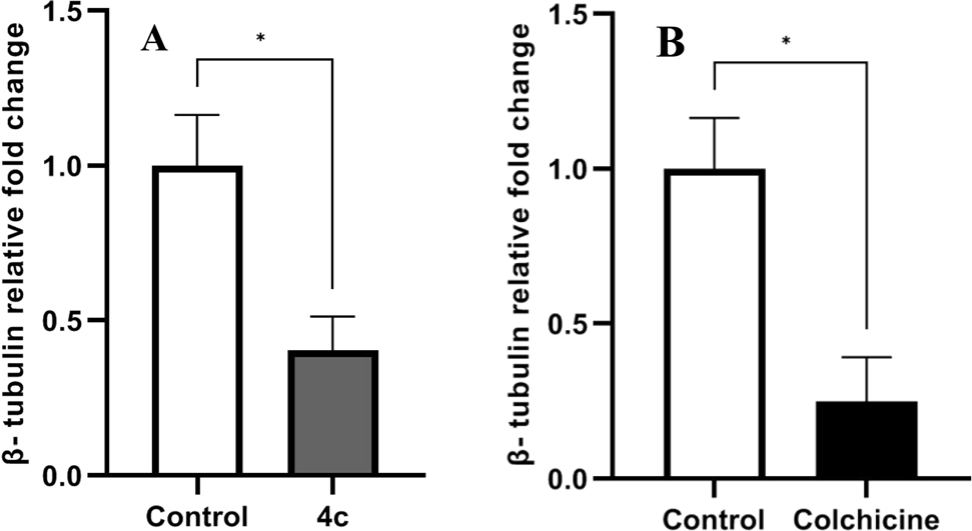
Relative gene expression of β-tubulin induced by (A) 4c and (B) colchicine compared with the control. Data are represented as mean ± SD of three individual experiments. Statistical analysis was done by applying Mann–Whitney test; *p < 0.05, **p < 0.01, ***p < 0.001, ****p < 0.0001 compared to the control.

α-Tubulin and β-tubulin subunits are the main building blocks of microtubules.^6^ Several tubulin polymerization inhibitors interfere with microtubule assembly.^44^ However, such inhibitors can additionally suppress β-tubulin expression. Thus, we investigated the ability of compound 4c to decrease β-tubulin mRNA levels. MDA-MB-231 cells were treated with either compound 4c or colchicine and the expression of β-tubulin was determined by real-time PCR. As demonstrated in Fig. 11A and B, both compound 4c and colchicine significantly suppressed β-tubulin transcription compared with non-treated cells.

### 2.3. *In silico* studies

#### 2.3.1. Docking studies

Discovery Studio client software was used for the analysis of the possible binding modes between our titled compounds and CBS of tubulin. According to reports, the colchicine binding site comprises two hydrophilic sites partially located at the α-tubulin subunit that are prone to form additional hydrogen bonds with ligands that add stability to the interactions^49,50^ and three hydrophobic pockets located at the β-tubulin subunit that serve as the main locations for ligand interactions. αThr179, αVal181, and βLeu248 are residues that engage in hydrophilic interactions, whereas the hydrophobic pockets, which are the primary target for compounds in the “deep binding mode” category, include essential amino acids as αAla180, βCys241, βAla250, βAsn258, βAla316, βIle318, and βLys352.^50^

**Fig. 12:**
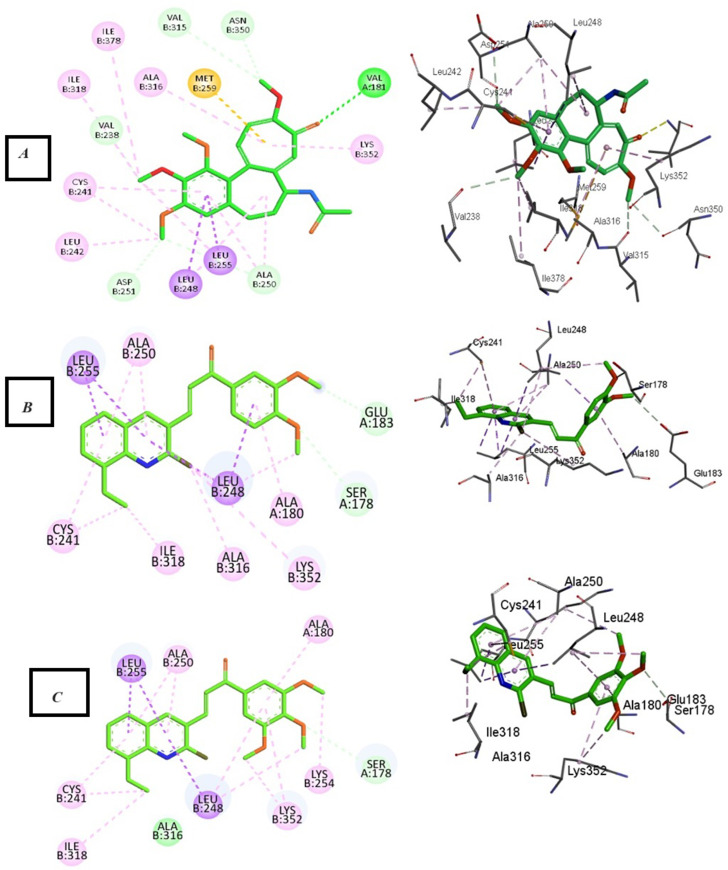
2D and 3D interaction diagrams of (A) colchicine, (B) compound 3b and (C) compound 3c into the colchicine binding site of tubulin enzyme.

**Fig. 13:**
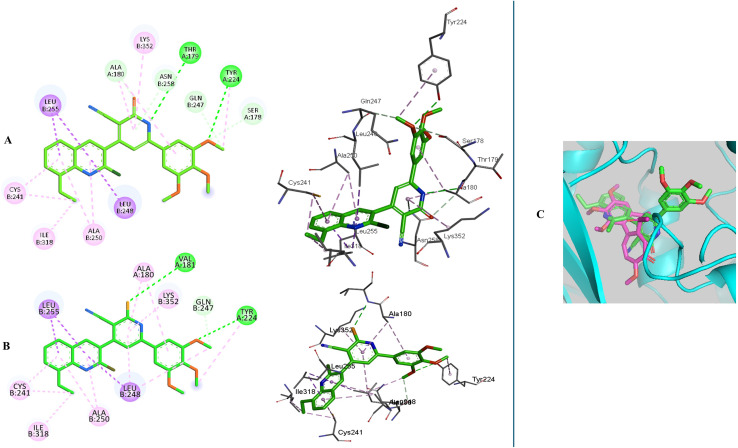
2D and 3D interaction diagrams of (A) compound 4c, (B) compound 5c into the colchicine binding site of tubulin enzyme and (C) aligned docking pose of colchicine (pink) and 4c into the CBS.

In the current simulation, redocking of colchicine was done which revealed the reported binding mode of the compound.^51,52^ After validation of our docking procedure, the titled compounds 3b, 3c, 4c, and 5c displayed correct binding modes into the CBS. Chalcone derivatives 3b and 3c which were lethal against non-small cell lung cancer cells; NCI-H226, CNS cancer cells; SF-539 and SNB-75 and ovarian cancer cells; OVCAR-3, showed a high binding interaction within the hydrophobic pocket of the β-tubulin subunit with the correct pose equal to −9.0 and −8.0 kcal mol^−1^, respectively. As can be deduced from (Fig. 12) both compounds revealed identical binding modes where they were deeply buried in the colchicine site at the α, β intradimer interface and formed mainly hydrophobic contacts with several residues of β-tubulin where the 2-chloro-8-ethylquinoline ring bonded through several π-alkyl and π-sigma interactions to βCys241, βLeu248, βAla250, βLeu255, βAla316, βIle318 and βLys352 (Table S4†). The dimethoxy and trimethoxyphenyl rings of 3b and 3c, respectively made H-bond interactions with αSer178 (3.33 and 3.29 Å, respectively) acting as hydrogen acceptors, both rings made π-alkyl interactions with αAla180. The dimethoxy group in 3b formed an extra H-bond with αGlu183 (3.48 Å) acting as a hydrogen donor while the trimethoxy group in 3c extended towards βLys254 forming π-alkyl interaction with the residue. The 2-oxo-1,2-dihydropyridine derivative 4c was the most cytotoxic synthesized compound against 60 cell lines. It inhibited the growth of most of the tested cells and was lethal towards many non-small cell lung cancer cell lines, CNS cancer cells, renal and breast cancer cells, while its isosteric derivative 2-thioxo-1,2-dihydropyridine containing compound 5c revealed less cytotoxic effect than 4c and exhibited reasonable cytotoxicity against most of the tested cell lines. Compound 4c revealed the highest predicted binding interaction with CBS of tubulin compared with 5c and colchicine equal to −11.5, −10.9 and −9.8 kcal mol^−1^, respectively, which support the results of the biological assay. Compounds 4c and 5c fitted comfortably into the active space of the CBS of tubulin, their mode of binding was almost identical and was like that of colchicine as revealed by superimposing both compounds (Fig. 13). The 2-chloro-8-ethylquinoline ring oriented itself towards the hydrophobic pocket of the CBS of tubulin forming multiple π-alkyl and π-sigma interactions with βCys241, βLeu248, βAla250, βLeu255 and βIle318. The trimethoxy phenyl ring in both compounds faced the hydrophilic region of CBS of tubulin. Both compounds are linked to αTyr224 and βGln247. Compound 4c formed hydrogen bond interactions with both residues (3.28 and 3.26 Å, respectively) acting as hydrogen acceptor and donor, respectively. Compound 5c bonded to the same two residues with π-alkyl and π-sigma interactions, respectively. Moreover, 4c made an additional hydrogen bond through its trimethoxy group with αSer178 (3.34 Å) acting as a hydrogen acceptor. π-Alkyl interaction occurred between βLys352 and the 2-oxo-1,2-dihydropyridine ring in compound 4c, as well as with the 2-thioxo-1,2-dihydropyridine ring in compound 5c. 2-Oxo-1,2-dihydropyridine moiety in 4c linked by three hydrogen bonds to αThr179, αAla180 and βAsn258 (3.37, 3.29, 4.12 Å, respectively) acting as hydrogen donor, acceptor and donor, respectively. Conversely, in compound 5c the 2-thioxo-1,2-dihydropyridine ring in 5c formed a hydrogen bond with αVal181 (3.65 Å) acting as hydrogen acceptor. Close analysis of the binding mode of 4c revealed that it formed many hydrogen bond interactions with the hydrophilic part of CBS of tubulin in addition to the hydrophobic interactions with the β-tubulin subunit. These interactions support the promising activity of compound 4c towards tubulin, as well as its cytotoxicity against numerous cancer cell lines (Table 3).

**Table 3:** CDocker energy of the tested compounds 3b, 3c, 4c and 5c and colchicine

Compound	CDocker energy (kcal mol^−1^), tubulin enzyme (PDB ID: 4O2B)
3b	−9.0
3c	−8.0
4c	−11.5
5c	−10.9
Colchicine	−9.8

#### 2.3.2. Physicochemical properties

**Fig. 14:**
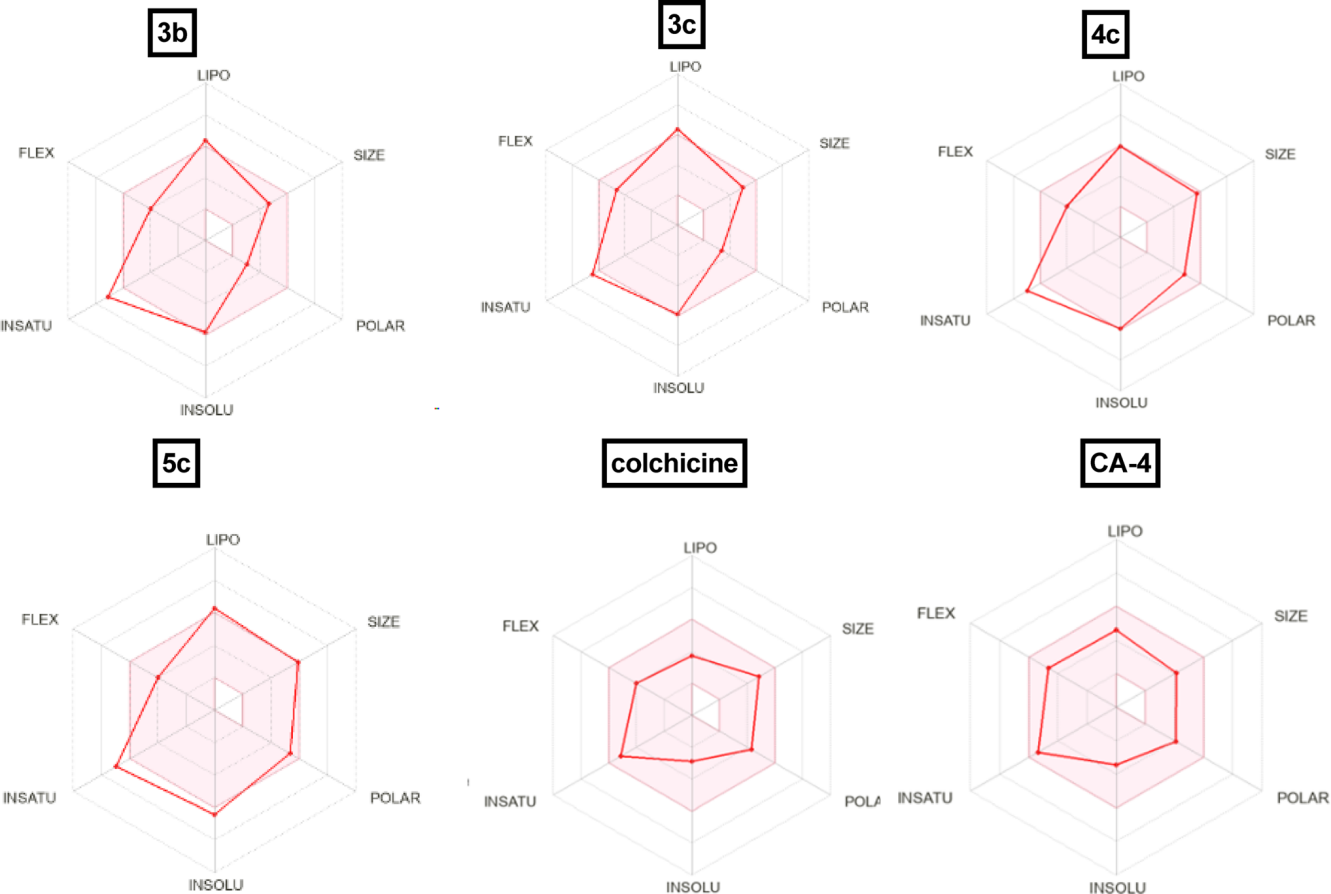
Radar chart showing six predicted physicochemical properties of the tested compound 3b, 3c, 4c, 5c, colchicine and CA-4.

**Fig. 15:**
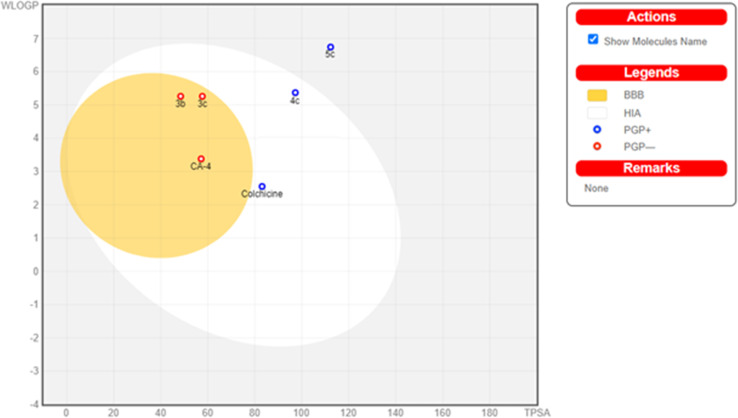
Boiled egg model of the tested compounds 3b, 3c, 4c, 5c, colchicine and CA-4.

The physicochemical features of the most promising cytotoxic compounds 3b, 3c, 4c and 5c against various tested cell lines were investigated compared to reference drugs colchicine and CA-4. The results of the ADME revealed six physicochemical properties: lipophilicity, size, polarity, solubility, saturation, and flexibility. Compounds 3b, 3c and 5c showed a slight deviation in the saturation and solubility properties from the validated range compared to colchicine and CA-4 while compound 4c showed a slight deviation in saturation only (Fig. 14) and compound 6c showed no deviation in all properties. Other physicochemical parameters include topological polar surface area, pan assay interference structures, lipophilicity petameter WLOGP, number of rotatable bonds, number of hydrogen bond acceptors, number of hydrogen bond donors, gastrointestinal absorption blood–brain barrier permeability and drug likeness are shown in Table 4. Moreover, compounds 3b, 3c and CA-4 could not be good substrates for *P*-glycoprotein (Pgp) while compounds 4c, 5c and colchicine could be good substrates for Pgp. In addition, compound 4c and colchicine showed high GI absorption as they were located in the Boiled-Egg's white but compound 5c showed low GI absorption as it wasn't located in the Boiled-Egg's white. Finally, 3b, 3c and CA-4 which were located in the Boiled-Egg yolk could penetrate the blood–brain barrier (Fig. 15).

**Table 4:** The predicted pharmacokinetic profile for the investigated compounds 3b, 3c, 4c, 5c, colchicine and CA-4

Comp. no.	TPSA^a^	PAINS^b^	WLOGP^c^	NRB^d^	HBD^e^	HBA^f^	GI absorption^g^	BBB permeability^h^	Lipinski^i^
3b	48.42	0	5.26	6	0	4	High	Yes	0 violation
3c	57.65	0	5.26	7	0	5	High	Yes	0 violation
4c	97.23	0	5.37	6	1	6	High	No	0 violation
5c	112.25	0	6.74	6	1	5	Low	No	0 violation
Colchicine	83.09	0	2.55	6	1	6	High	No	0 violation
CA-4	77.38	0	2.38	7	2	6	High	Yes	0 violation

a Topological polar surface area.

b Pan assay interference structures.

c Lipophilicity petameter WLOGP.

d Number of rotatable bonds.

e Number of hydrogen bond acceptors.

f Number of hydrogen bond donor.

g Gastrointestinal absorption.

h Blood–brain barrier permeability.

i Lipiniki (drug likeness).

## 3. Conclusion

To sum up, a group of novel quinoline derivatives 3a–e, 4a–e, 5a–e, and 6a–e were designed and synthesized as tubulin polymerization inhibitors targeting the colchicine binding site. Their cytotoxic activity against 60 cell lines by NCI were evaluated. Compounds 3b, 3c, 4c, 5c and 6c possessed the most remarkable antitumor activity against almost all cell lines, especially compound 4c (pyridin-2-one ring substituted with 3,4,5-trimethoxy groups) which was the most potent compound as an antiproliferative agent. Compound 4c significantly induced cell cycle arrest at G2 and M phases and encouraged apoptosis, as evidenced by a rise in the number of cells in both early and late phases of apoptosis. Compound 4c successfully inhibited tubulin polymerization with IC_50_ value 17 ± 0.3 μM. The β-tubulin mRNA levels were markedly reduced in MDA-MB-231 cells treated with compound 4c, resembling the effect observed with colchicine treatment. Molecular docking exhibited the interaction mode of compound 4c with tubulin including formation of hydrogen bonds and hydrophobic interactions, which demonstrated energy score −11.5 kcal mol^−1^ in comparison to colchicine with energy score −9.8 kcal mol^−1^, and it interacted with essential amino acids in the active sites. Compound 4c could has high GI absorption, drug-like properties and the ability to penetrate the blood–brain barrier. Taken together, compound 4c is a highly promising candidate for further preclinical studies against breast cancer.

## 4. Experimental

### 4.1. Chemistry

#### 4.1.1. Procedure for synthesis of *N*-(2-ethylphenyl)acetamide (1)^33,34,36^

To a solution of 2-ethyl aniline (20 mmol, 2.42 mL) in glacial acetic acid (5 mL), acetic anhydride (20 mmol, 2.04 mL) was added, and the mixture was stirred in ice bath for 2 h. The mixture was poured onto ice-cooled water after completion of the reaction, the formed precipitate was collected by filtration and then dried to be used in the next step.

#### 4.1.2. Procedure for synthesis of 2-chloro-8-ethylquinoline-3-carbaldehyde (2)^35,36^

DMF (62.5 mmol, 4.55 mL) in an ice bath (0–5 °C) was mixed continuously while phosphorus oxychloride (175 mmol, 26.82 mL) was added dropwise. Acetanilide 1 (25 mmol, 4.08 g) was then added portion-wise. The reaction mixture was heated in a water bath for 16 h, at 70 to 90 °C, and after that, it was poured onto ice-cold water and stirred until a precipitate was produced. The mixture was let to stand overnight before being filtered, dried, and crystallized from ethyl acetate to produce compound 2.

#### 4.1.3. General procedure for synthesis of 3-(2-chloro-8-ethylquinolin-3-yl)-1-(substituted phenyl)prop-2-en-1-one (3a–e)

To a mixture of 2-chloro-8-ethylquinoline-3-carbaldehyde (2) (10 mmol, 2.19 g) and the appropriate substituted acetophenone (10 mmol) in absolute ethanol (30 mL), an aqueous solution of potassium hydroxide (50%, 5 mL) was added while stirring. Then the reaction mixture was stirred for an additional 3 h at room temperature. The separated product was collected by filtration and crystallized from methanol to give compounds 3a–e.

##### 3-(2-Chloro-8-ethylquinolin-3-yl)-1-(4-methoxyphenyl)prop-2-en-1-one (3a)

Yellow powder, yield 85%, mp 138–140 °C. IR (KBr, cm^−1^): 3022 (CH aromatic), 2960 (CH aliphatic), 1656 (C=O), 1568 (C=N). ^1^H NMR (400 MHz, DMSO-*d*_6_), *δ* ppm: 1.30 (t, 3H, *J* = 8.0 Hz, CH_2_CH̲_3_), 3.11–3.16 (q, 2H, *J* = 8.0 Hz, CH̲_2_CH_3_), 3.89 (s, 3H, OCH_3_), 7.12 (d, 2H, *J* = 8.0 Hz, Ar-H), 7.64 (t, 1H, *J* = 8.0 Hz, Ar-H), 7.71 (d, 1H, *J* = 8.0 Hz, Ar-H), 7.91 (d, 1H, *J* = 8.0 Hz, Ar-H), 8.02 (d, 1H, *J* = 16 Hz, CH alkene β proton), 8.16 (d, 1H, *J* = 16 Hz, CH alkene α proton), 8.21 (d, 2H, *J* = 8.0 Hz, Ar-H), 9.21 (s, 1H, Ar-H). ^13^C NMR (100 MHz, DMSO-*d*_6_), *δ* ppm: 15.2, 24.0, 56.0, 114.5 (2C), 126.2, 126.9, 127.0, 127.4, 128.1, 130.4, 130.6, 131.5 (2C), 137.3, 138.0, 141.6, 145.9, 149.1, 163.9, 187.2. Anal. calcd for C_21_H_18_ClNO_2_ (351.83): C, 71.69; H, 5.16; N 3.98; found: C, 71.85; H, 5.33; N, 4.21.

##### 3-(2-Chloro-8-ethylquinolin-3-yl)-1-(3,4-dimethoxyphenyl)prop-2-en-1-one (3b)

Yellow powder, yield 84%, mp 135–137 °C. IR (KBr, cm^−1^): 3060 (CH aromatic), 2964 (CH aliphatic), 1653 (C=O), 1577 (C=N). ^1^H NMR (400 MHz, DMSO-*d*_6_), *δ* ppm: 1.29 (t, 3H, *J* = 8.0 Hz, CH_2_CH̲_3_), 3.09–3.15 (q, 2H, *J* = 8.0 Hz, CH̲_2_CH_3_), 3.87 (s, 3H, OCH_3_), 3.89 (s, 3H, OCH_3_), 7.13 (d, 1H, *J* = 8.0 Hz, Ar-H), 7.62 (t, 2H, *J* = 8.0 Hz, Ar-H), 7.70 (d, 1H, *J* = 8.0 Hz, Ar-H), 7.89–7.95 (m, 2H, Ar-H), 8.00 (d, 1H, *J* = 16 Hz, CH alkene β proton), 8.13 (d, 1H, *J* = 16 Hz, CH alkene α proton), 9.15 (s, 1H, Ar-H). ^13^C NMR (100 MHz, DMSO-*d*_6_), *δ* ppm: 15.3, 24.0, 56.1, 56.3, 111.2, 111.3, 124.3, 126.5, 127.0, 127.3, 127.5, 128.3, 130.5, 130.8, 137.4, 138.2, 141.7, 146.0, 149.2, 149.3, 154.0, 187.3. Anal. calcd for C_22_H_20_ClNO_3_ (381.86): C, 69.20; H, 5.28; N 3.67; found: C, 69.43; H, 5.40; N, 3.85.

##### 3-(2-Chloro-8-ethylquinolin-3-yl)-1-(3,4,5-trimethoxyphenyl)prop-2-en-1-one (3c)

White powder, yield 86%, mp 140–142 °C. IR (KBr, cm^−1^): 3064 (CH aromatic), 2958 (CH aliphatic), 1639 (C=O), 1583 (C=N). ^1^H NMR (400 MHz, DMSO-*d*_6_), *δ* ppm: 1.28 (t, 3H, *J* = 8.0 Hz, CH_2_CH̲_3_), 3.06–3.15 (q, 2H, *J* = 8.0 Hz, CH̲_2_CH_3_), 3.79 (s, 3H, OCH_3_), 3.92 (s, 6H, 2× OCH_3_), 7.46 (s, 2H, Ar-H), 7.64 (t, 1H, *J* = 8.0 Hz, Ar-H), 7.72 (d, 1H, *J* = 8.0 Hz, Ar-H), 7.93 (d, 1H, *J* = 8.0 Hz, Ar-H), 8.04 (d, 1H, *J* = 16 Hz, CH alkene β proton), 8.12 (d, 1H, *J* = 12 Hz, CH alkene α proton), 9.14 (s, 1H, Ar-H). ^13^C NMR (100 MHz, DMSO-*d*_6_), *δ* ppm: 15.3, 24.0, 56.7, 60.7 (2C), 106.8 (2C), 126.1, 126.2, 127.0, 127.4, 130.8, 132.8, 138.2, 138.3, 141.7, 142.7, 145.9, 149.1, 150.7, 153.3 (2C), 188.0 Anal. calcd for C_23_H_22_ClNO_4_ (411.88): C, 67.07; H, 5.38; N 3.40; found: C, 67.41; H, 5.45; N, 3.62.

##### 3-(2-Chloro-8-ethylquinolin-3-yl)-1-(4-fluorophenyl)prop-2-en-1-one (3d)

White powder, yield 83%, mp 215–217 °C. IR (KBr, cm^−1^): 3078 (CH aromatic), 2964 (CH aliphatic), 1643 (C=O), 1598 (C=N). ^1^H NMR (400 MHz, DMSO-*d*_6_), *δ* ppm: 1.31 (t, 3H, *J* = 8.0 Hz, CH_2_CH̲_3_), 3.11–3.17 (q, 2H, *J* = 8.0 Hz, CH̲_2_CH_3_), 7.46 (t, 2H, *J* = 8.0 Hz, Ar-H), 7.60 (d, 1H, *J* = 7.2 Hz, Ar-H), 7.64 (d, 1H, *J* = 16 Hz, CH alkene β proton), 7.74 (t, 1H, *J* = 8.0 Hz, Ar-H), 8.06–8.09 (m, 3H. Ar-H), 8.20 (d, 1H, *J* = 16 Hz, CH alkene α proton), 9.29 (s, 1H, Ar-H). ^13^C NMR (100 MHz, DMSO-*d*_6_), *δ* ppm: 15.5, 23.6, 115.3 (2C), 126.1, 126.9, 127.0, 127.5, 128.1, 130.4, 130.6, 131.6 (2C), 137.4, 138.1, 141.6, 146.0, 149.2, 167.3, 187.5. Anal. calcd for C_20_H_15_ClFNO (339.79): C, 70.70; H, 4.45; N 4.12; found: C, 70.59; H, 4.62; N, 4.37.

##### 1-(4-Bromophenyl)-3-(2-chloro-8-ethylquinolin-3-yl)prop-2-en-1-one (3e)

White powder, yield 83%, mp 210–212 °C. IR (KBr, cm^−1^): 3101 (CH aromatic), 2958 (CH aliphatic), 1600 (C=O), 1583 (C=N). ^1^H NMR (400 MHz, DMSO-*d*_6_), *δ* ppm: 1.31 (t, 3H, *J* = 8.0 Hz, CH_2_CH̲_3_), 3.06–3.15 (q, 2H, *J* = 8.0 Hz, CH̲_2_CH_3_), 7.06 (d, 1H, *J* = 8 Hz, Ar-H), 7.36 (t, 1H, *J* = 8.0 Hz, Ar-H), 7.61 (m, 1H, Ar-H), 7.73 (d, 2H, *J* = 8.4 Hz, Ar-H), 7.91 (d, 2H, *J* = 8.4 Hz, Ar-H), 8.11 (d, 1H, *J* = 15.2 Hz, CH alkene β proton), 8.17 (d, 1H, *J* = 16 Hz, CH alkene α proton), 8.50 (s, 1H, Ar-H). ^13^C NMR (100 MHz, DMSO-*d*_6_), *δ* ppm: 15.9, 23.6, 126.2, 126.8, 127.1, 127.5, 128.2, 128.5 (2C), 129.1, 130.3, 130.6, 131.7 (2C), 137.9, 139.9, 142.3, 146.0, 150.3, 186.8. Anal. calcd for C_20_H_15_ClBrNO (400.70): C, 59.95; H, 3.77; N 3.50; found: C, 60.23; H, 3.91; N, 3.64.

#### 4.1.4. General procedure for synthesis of 4-(2-chloro-8-ethylquinolin-3-yl)-6-(substituted phenyl)-2-oxo-1,2-dihydropyridine-3-carbonitrile (4a–e)

2-Chloro-8-ethylquinoline-3-carbaldehyde (2) (1 mmol, 0.219 g) was heated under reflux for 4–6 h with the appropriate substituted acetophenone (1 mmol), ethyl cyanoacetate (1 mmol, 0.11 g), and ammonium acetate (8 mmol, 0.62 g) in absolute ethanol (10 mL). After cooling the formed solid was filtered, dried and crystallized from methanol to give compounds 4a–e.

##### 4-(2-Chloro-8-ethylquinolin-3-yl)-6-(4-methoxyphenyl)-2-oxo-1,2-dihydropyridine-3-carbonitrile (4a)

Yellow powder, yield 79%, mp 270–272 °C. IR (KBr, cm^−1^): 3421 (NH), 3095 (CH aromatic), 2962 (CH aliphatic), 2218 (CN), 1647 (C=O), 1514 (C=N). ^1^H NMR (400 MHz, DMSO-*d*_6_), *δ* ppm: 1.34 (t, 3H, *J* = 8.0 Hz, CH_2_CH̲_3_), 3.14–3.20 (m, 2H, CH̲_2_CH_3_ + s, 1H, NH, D_2_O exchangeable), 3.84 (s, 3H, OCH_3_), 7.00 (s, 1H, CH pyridine), 7.08 (d, 2H, *J* = 8.8 Hz, Ar-H), 7.69 (t, 1H, *J* = 8.0 Hz, Ar-H), 7.80 (d, 1H, *J* = 6.8 Hz, Ar-H), 7.92 (d, 2H, *J* = 8.8 Hz, Ar-H), 7.96 (d, 1H, *J* = 8.0 Hz, Ar-H), 8.66 (s, 1H, Ar-H). ^13^C NMR (100 MHz, DMSO-*d*_6_), *δ* ppm: 15.3, 23.9, 55.7, 106.4, 115.0 (2C), 116.4, 124.8, 126.1, 126.8, 126.9, 127.8, 127.9, 128.6, 129.8, 129.9 (2C), 131.0, 140.3, 141.9, 145.9, 152.9, 156.7, 162.4. MS *m*/*z* (%): 417.40 (M + 2, 5.89), 415.49 (M^+^, 13.79), 125.14 (100.00). Anal. calcd for C_24_H_18_ClN_3_O_2_ (415.88): C, 69.31; H, 4.36; N 10.10; found: C, 69.08; H, 4.50; N, 10.29.

##### 4-(2-Chloro-8-ethylquinolin-3-yl)-6-(3,4-dimethoxyphenyl)-2-oxo-1,2-dihydropyridine-3-carbonitrile (4b)

Yellow powder, yield 71%, mp 280–282 °C. IR (KBr, cm^−1^): 3431 (NH), 3040 (CH aromatic), 2962 (CH aliphatic), 2239 (CN), 1641 (C=O), 1517 (C=N). ^1^H NMR (400 MHz, DMSO-*d*_6_), *δ* ppm: 1.34 (t, 3H, *J* = 8.0 Hz, CH_2_CH̲_3_), 2.52 (s, 1H, NH, D_2_O exchangeable), 3.17–3.26 (m, 2H, CH̲_2_CH_3_), 3.80 (s, 3H, OCH_3_), 3.83 (s, 3H, OCH_3_), 6.85 (s, 1H, CH pyridine), 7.00 (d, 1H, *J* = 8.4 Hz, Ar-H), 7.60–7.67 (m, 3H, Ar-H), 7.75 (d, 1H, *J* = 6.8 Hz, Ar-H), 7.95 (d, 1H, *J* = 8.0 Hz, Ar-H), 8.52 (s, 1H, Ar-H). ^13^C NMR (100 MHz, DMSO-*d*_6_), *δ* ppm: 15.5, 22.6, 56.0, 56.1, 105.8, 110.5, 110.8, 111.0, 111.8, 181.2, 121.0, 123.7, 126.7, 127.1, 128.1, 130.4, 130.9, 140.0, 141.9, 145.8, 146.8, 149.3, 151.3, 156.1, 173.9. MS *m*/*z* (%): 447.88 (M + 2, 3.22), 445.74 (M^+^, 9.87), 313.27 (100.00). Anal. calcd for C_25_H_20_ClN_3_O_3_ (445.90): C, 67.34; H, 4.52; N 9.42; found: C, 67.53; H, 4.61; N, 9.68.

##### 4-(2-Chloro-8-ethylquinolin-3-yl)-2-oxo-6-(3,4,5-trimethoxyphenyl)-1,2-dihydropyridine-3-carbonitrile (4c)

Yellow powder, yield 72%, mp 297–299 °C. IR (KBr, cm^−1^): 3446 (NH), 3140 (CH aromatic), 2953 (CH aliphatic), 2220 (CN), 1645 (C=O), 1581 (C=N). ^1^H NMR (400 MHz, DMSO-*d*_6_), *δ* ppm: 1.33 (t, 3H, *J* = 8.0 Hz, CH_2_CH̲_3_), 3.14–3.23 (m, 3H (2H, CH̲_2_CH_3_ + 1H, NH, D_2_O exchangeable)), 3.73 (s, 3H, OCH_3_), 3.86 (s, 6H, 2OCH_3_), 7.16 (s, 1H, CH pyridine), 7.26 (s, 2H, Ar-H), 7.69 (t 1H, *J* = 8.0 Hz, Ar-H), 7.80 (d, 1H, *J* = 8 Hz, Ar-H), 7.98 (d, 1H, *J* = 8 Hz, Ar-H), 8.66 (s, 1H, Ar-H). ^13^C NMR (100 MHz, DMSO-*d*_6_), *δ* ppm: 16.2, 23.9, 56.7, 60.8 (2C), 105.6 (2C), 107.1, 116.3, 116.7, 126.9, 127.6, 128.0, 128.6, 129.8, 130.7, 140.3, 140.5, 141.8, 145.9, 146.1, 152.6, 153.6 (2C), 156.7, 162.6. MS *m*/*z* (%): 477.68 (M + 2, 11.23), 475.05 (M^+^, 34.00), 42.89 (100.00). Anal. calcd for C_26_H_22_ClN_3_O_4_ (475.93): C, 65.62; H, 4.66; N 8.83; found: C, 65.49; H, 4.85; N, 9.07.

##### 4-(2-Chloro-8-ethylquinolin-3-yl)-6-(4-fluorophenyl)-2-oxo-1,2-dihydropyridine-3-carbonitrile (4d)

White powder, yield 77%, mp 260–262 °C. IR (KBr, cm^−1^): 3431 (NH), 3047 (CH aromatic), 2966 (CH aliphatic), 2222 (CN), 1656 (C=O), 1535 (C=N). ^1^H NMR (400 MHz, DMSO-*d*_6_), *δ* ppm: 1.34 (t, 3H, *J* = 8.0 Hz, CH_2_CH̲_3_), 3.11–3.21 (m, 2H, CH̲_2_CH_3_ + s, 1H, NH, D_2_O exchangeable), 7.11 (s, 1H, CH pyridine),7.39 (t, 2H, *J* = 8.0 Hz, Ar-H), 7.63–7.71 (m, 1H, Ar-H), 7.80 (d, 1H, *J* = 6.8 Hz, Ar-H), 7.96–7.803 (m, 3H, Ar-H), 8.68 (s, 1H, Ar-H). ^13^C NMR (100 MHz, DMSO-*d*_6_), *δ* ppm: 16.6, 23.3, 106.2, 114.6 (2C), 117.6, 119.5, 120.6, 122.5, 124.2, 125.9, 127.2 (2C), 127.9, 129.2, 129.9, 131.3, 144.6, 147.3, 153.6, 159.7, 161.7, 162.4. MS *m*/*z* (%): 405.62 (M + 2, 4.18), 403.80 (M^+^, 11.20), 376.34 (100.00). Anal. calcd for C_23_H_15_ClFN_3_O (403.84): C, 68.41; H, 3.74; N, 10.41; found: C, 68.70; H, 3.86; N 10.64.

##### 6-(4-Bromophenyl)-4-(2-chloro-8-ethylquinolin-3-yl)-2-oxo-1,2-dihydropyridine-3-carbonitrile (4e)

White powder, yield 78%, mp 290–262 °C. IR (KBr, cm^−1^): 3419 (NH), 3034 (CH aromatic), 2964 (CH aliphatic), 2222 (CN), 1647 (C=O), 1589 (C=N). ^1^H NMR (400 MHz, DMSO-*d*_6_), *δ* ppm: 1.33 (t, 3H, *J* = 8.0 Hz, CH_2_CH̲_3_), 1.87 (s, 1H, NH, D_2_O exchangeable), 3.06–3.15 (m, 2H, CH̲_2_CH_3_), 7.00 (s, 1H, CH pyridine),7.69–7.70 (m, 3H, Ar-H), 7.79 (t, 1H, *J* = 8.0 Hz, Ar-H), 7.91–7.96 (m, 3H, Ar-H), 8.61 (s, 1H, Ar-H). MS *m*/*z* (%): 466.63 (M + 2, 29.98), 465.61 (M + 1, 54.96), 464.52 (M^+^, 48.74), 353.35 (100.00). Anal. calcd for C_23_H_15_BrClN_3_O (464.75): C, 59.44; H, 3.25; N 9.04; found: C, 59.32; H, 3.41; N, 9.29.

#### 4.1.5. General procedure for synthesis of 4-(2-chloro-8-ethylquinolin-3-yl)-6-(substituted phenyl)-2-thioxo-1,2-dihydropyridine-3-carbonitrile (5a–e)

A mixture of 2-chloro-8-ethylquinoline-3-carbaldehyde (2). (1 mmol, 0.219 g), required acetophenones (1 mmol), 2-cyanothioacetamide (1 mmol, 0.1 g), and ammonium acetate (8 mmol, 0.62 g) in absolute ethanol (10 mL) was heated under reflux for 6–8 h. After the reaction mixture was cooled to room temperature, the separated solid was filtered, dried and crystallized from methanol to afford compounds 5a–e.

##### 4-(2-Chloro-8-ethylquinolin-3-yl)-6-(4-methoxyphenyl)-2-thioxo-1,2-dihydropyridine-3-carbonitrile (5a)

Yellow powder, yield 71%, mp 290–292 °C. IR (KBr, cm^−1^): 3433 (NH), 3070 (CH aromatic), 2962 (CH aliphatic), 2218 (CN), 1577 (C=N), 1147 (C=S). ^1^H NMR (400 MHz, DMSO-*d*_6_), *δ* ppm: 1.33 (t, 3H, *J* = 8.0 Hz, CH_2_CH̲_3_), 3.15–3.21 (q, 2H, *J* = 8.0 Hz, CH̲_2_CH_3_ + s, 1H, NH, D_2_O exchangeable), 3.83 (s, 3H, OCH_3_), 6.99 (s, 1H, CH pyridine), 7.08 (d, 2H, *J* = 9.3 Hz, Ar-H), 7.68 (t, 1H, *J* = 8.0 Hz, Ar-H), 7.79 (d, 1H, *J* = 7.2 Hz, Ar-H), 7.92 (d, 2H, *J* = 8.8 Hz, Ar-H), 7.96 (d, 1H, *J* = 7.6 Hz, Ar-H), 8.65 (s, 1H, Ar-H). ^13^C NMR (100 MHz, DMSO-*d*_6_), *δ* ppm: 15.3, 24.3, 56.3, 106.7, 114.9 (2C), 117.0, 120.7, 121.4, 124.8, 125.4, 126.7, 126.9, 128.6, 130.0 (2C), 131.0, 140.3, 142.0, 145.8, 152.8, 156.5, 160.1, 162.4. MS *m*/*z* (%): 433.93 (M + 2, 16.24), 432.21 (M + 1, 40.09), 431.34 (M^+^, 50.68), 423.98 (100.00). Anal. calcd for C_24_H_18_ClN_3_OS (431.94): C, 66.74; H, 4.20; N 9.73; found: C, 66.83; H, 4.34; N, 9.91.

##### 4-(2-Chloro-8-ethylquinolin-3-yl)-6-(3,4-dimethoxyphenyl)-2-thioxo-1,2-dihydropyridine-3-carbonitrile (5b)

Yellow powder, yield 71%, mp 280–282 °C. IR (KBr, cm^−1^): 3446 (NH), 3151 (CH aromatic), 2964 (CH aliphatic), 2218 (CN), 1577 (C=N), 1145 (C=S). ^1^H NMR (400 MHz, DMSO-*d*_6_), *δ* ppm: 1.33 (t, 3H, *J* = 8.0 Hz, CH_2_CH̲_3_), 1.88 (s, 1H, NH, D_2_O exchangeable), 3.20 (s, 2H, CH̲_2_CH_3_), 3.83 (s, 3H, OCH_3_), 3.84 (s, 3H, OCH_3_), 7.02 (s, 1H, CH pyridine), 7.08 (d, 1H, *J* = 8.4 Hz, Ar-H), 7.33–7.61 (m, 2H, Ar-H), 7.68 (t, 1H, *J* = 8.0 Hz, Ar-H), 7.79 (d, 1H, *J* = 6.8 Hz, Ar-H), 7.96 (d, 1H, *J* = 7.6 Hz, Ar-H), 8.63 (s, 1H, Ar-H). ^13^C NMR (100 MHz, DMSO-*d*_6_), *δ* ppm: 15.3, 24.3, 56.1, 56.2, 100.0, 106.5, 110.9, 112.3, 116.4, 121.6, 124.6, 124.7, 126.8, 126.9, 128.3, 129.9, 130.8, 140.3, 141.8, 145.9, 146.1, 149.3, 152.1, 156.7, 162.5 MS *m*/*z* (%): 463.93 (M + 2, 2.78), 462.63 (M + 1, 9.64), 461.68 (M^+^, 6.26), 43.21 (100.00). Anal. calcd for C_25_H_20_ClN_3_O_2_S (461.96): C, 65.00; H, 4.36; N 9.10; found: C, 64.88; H, 4.51; N, 9.32.

##### 4-(2-Chloro-8-ethylquinolin-3-yl)-2-thioxo-6-(3,4,5-trimethoxyphenyl)-1,2-dihydropyridine-3-carbonitrile (5c)

Yellow powder, yield 69%, mp 279–281 °C. IR (KBr, cm^−1^): 3446 (NH), 3051 (CH aromatic), 2966 (CH aliphatic), 2220 (CN), 1585 (C=N), 1128 (C=S). ^1^H NMR (400 MHz, DMSO-*d*_6_), *δ* ppm: 1.31 (t, 3H, *J* = 8.0 Hz, CH_2_CH̲_3_), 3.18–3.22 (m, 2H, CH̲_2_CH_3_ + s, 1H, NH, D_2_O exchangeable), 3.73 (s, 3H, OCH_3_), 3.86 (s, 6H, 2× OCH_3_), 7.17 (s, 1H, CH pyridine), 7.25 (s, 2H, Ar-H), 7.67 (t, 1H, *J* = 8.0 Hz, Ar-H), 7.80 (d, 1H, *J* = 8 Hz, Ar-H), 7.98 (d, 1H, *J* = 8 Hz, Ar-H), 8.69 (s, 1H, Ar-H). ^13^C NMR (100 MHz, DMSO-*d*_6_), *δ* ppm: 14.9, 23.7, 56.8 (2C), 60.5, 101.8, 106.4, 109.2, 116.0 (2C), 126.1, 126.9, 127.4, 128.1, 129.6, 130.8, 132.7, 138.0, 138.4, 146.1, 149.3, 153.2 (2C), 159.5, 173.7, 175.8. MS *m*/*z* (%): 493.53 (M + 2, 26.83), 491.95 (M^+^, 72.17), 441.90 (100.00). Anal. calcd for C_26_H_22_ClN_3_O_3_S (491.99): C, 63.47; H, 4.51; N 8.54; found: C, 63.60; H, 4.63; N, 8.70.

##### 4-(2-Chloro-8-ethylquinolin-3-yl)-6-(4-fluorophenyl)-2-thioxo-1,2-dihydropyridine-3-carbonitrile (5d)

Yellow powder, yield 76%, mp 235–237 °C. IR (KBr, cm^−1^): 3398 (NH), 3049 (CH aromatic), 2966 (CH aliphatic), 2222 (CN), 1508 (C=N), 1165 (C=S). ^1^H NMR (400 MHz, DMSO-*d*_6_), *δ* ppm: 1.34 (t, 3H, *J* = 8.0 Hz, CH_2_CH̲_3_), 3.11–3.22 (m, 2H, CH̲_2_CH_3_ + s, 1H, NH, D_2_O exchangeable), 6.78 (s, 1H, CH pyridine), 7.23 (t, 2H, Ar-H), 7.65 (d, 1H, *J* = 8.0 Hz, Ar-H), 7.73–7.77 (m, 1H, Ar-H) 7.94 (d, 1H, *J* = 8 Hz, Ar-H), 8.08 (d, 2H, *J* = 7.6 Hz, Ar-H), 8.49 (s, 1H, Ar-H). ^13^C NMR (100 MHz, DMSO-*d*_6_), *δ* ppm: 15.0, 22.0, 105.5, 115.9, 116.1 (2C), 118.3, 126.1, 126.7, 128.1, 128.3 (2C), 128.5, 129.7, 130.0, 133.9, 141.5, 145.2, 145.7, 149.7, 162.2, 166.4, 173.6. MS *m*/*z* (%): 421.12 (M + 2, 9.84), 419.78 (M^+^, 27.88), 64.08 (100.00). Anal. calcd for C_23_H_15_ClFN_3_S (419.90): C, 65.79; H, 3.60; N 10.01; found: C, 66.05; H, 3.74; N, 10.29.

##### 6-(4-Bromophenyl)-4-(2-chloro-8-ethylquinolin-3-yl)-2-thioxo-1,2-dihydropyridine-3-carbonitrile (5e)

Buff powder, yield 79%, mp charring 250 °C. IR (KBr, cm^−1^): 3446 (NH), 3126 (CH aromatic), 2910 (CH aliphatic), 2218 (CN), 1564 (C=N), 1195 (C=S). ^1^H NMR (400 MHz, DMSO-*d*_6_), *δ* ppm: 1.33 (t, 3H, *J* = 8.0 Hz, CH_2_CH̲_3_), 3.17–3.26 (m, 2H, CH̲_2_CH_3_ + s, 1H, NH, D_2_O exchangeable), 6.99 (s, 1H, CH pyridine),7.05 (d, 2H, *J* = 9.2 Hz, Ar-H), 7.66 (t, 1H, *J* = 8.0 Hz, Ar-H), 7.77 (d, 1H, *J* = 7.2 Hz, Ar-H), 7.91–7.97 (m, 3H, Ar-H), 8.67 (s, 1H, Ar-H). MS *m*/*z* (%): 482.73 (M + 2, 19.45), 481.83 (M + 1, 17.34), 480.23 (M^+^, 41.55), 474.31 (100.00). Anal. calcd for C_23_H_15_BrClN_3_S (480.81): C, 57.46; H, 3.14; N 8.74; found: C, 57.70; H, 3.23; N, 8.97.

#### 4.1.6. General procedure for synthesis of 4-(2-chloro-8-ethylquinolin-3-yl)-6-(substituted phenyl)-3,4-dihydropyrimidine-2(1*H*)-thione (6a-e)

The corresponding chalcone 3a–e (10 mmol) and thiourea (10 mmol, 0.76 g) were added successively to a solution of sodium ethoxide (10 mmol) in absolute ethanol (15 mL). The reaction mixture was heated under reflux for 2 h. After completion of the reaction, the solvent was concentrated under reduced pressure then, poured into ice-cooled water and acidified with acetic acid (2 mL). The obtained precipitate was filtered, washed with water, dried and crystallized from ethanol to afford pyrimidine derivatives 6a–e.

##### 4-(2-Chloro-8-ethylquinolin-3-yl)-6-(4-methoxyphenyl)-3,4-dihydropyrimidine-2(1*H*)-thione (6a)

White powder, yield 71%, mp above 300 °C. IR (KBr, cm^−1^): 3165 (NH), 3016 (CH aromatic), 2964 (CH aliphatic), 1130 (C=S). ^1^H NMR (400 MHz, DMSO-*d*_6_), *δ* ppm: 1.28 (t, 3H, *J* = 8.0 Hz, CH_2_CH̲_3_), 3.12–3.19 (q, 2H, *J* = 8.0 Hz, CH̲_2_CH_3_), 3.76 (s, 3H, OCH_3_), 5.39 (d, 1H, CH pyrimidine, *J* = 4.4 Hz), 5.57 (d, 1H, CH pyrimidine, *J* = 4.4 Hz), 6.91 (d, 2H, *J* = 8.8 Hz, Ar-H), 7.45 (d, 2H, *J* = 8.8 Hz, Ar-H), 7.60 (t, 1H, *J* = 8.0 Hz, Ar-H), 7.69 (d, 1H, *J* = 7.6 Hz, Ar-H), 7.89 (d, 1H, *J* = 7.6 Hz, Ar-H), 8.21 (s, 1H, Ar-H), 9.16 (s, 1H, NH, D_2_O exchangeable), 10.04 (s, 1H, NH, D_2_O exchangeable). ^13^C NMR (100 MHz, DMSO-*d*_6_), *δ* ppm: 16.3, 24.1, 55.3, 58.3, 99.7, 120.7 (2C), 124.1, 125.8, 126.5, 126.8, 128.8, 130.2, 131.9 (2C), 136.3, 139.3, 141.7, 145.8, 148.5, 159.4, 175.6. Anal. calcd for C_22_H_20_ClN_3_OS: C, 64.46; H, 4.92; N 10.25; found: C, 64.32; H, 5.11; N, 10.49.

##### 4-(2-Chloro-8-ethylquinolin-3-yl)-6-(3,4-dimethoxyphenyl)-3,4-dihydropyrimidine-2(1*H*)-thione (6b)

Orange powder, yield 74%, mp 237–239 °C. IR (KBr, cm^−1^): IR (KBr, cm^−1^): 3196 (NH), 3080 (CH aromatic), 2960 (CH aliphatic), 1172 (C=S). ^1^H NMR (400 MHz, DMSO-*d*_6_), *δ* ppm: 1.29 (t, 3H, *J* = 8.0 Hz, CH_2_CH̲_3_), 3.12–3.16 (m, 2H, CH̲_2_CH_3_), 3.75 (s, 3H, OCH_3_), 3.78 (s, 3H, OCH_3_), 5.42 (d, 1H, CH pyrimidine, *J* = 4 Hz), 5.58 (d, 1H, CH pyrimidine, *J* = 4 Hz), 6.92 (d, 1H, *J* = 8 Hz, Ar-H), 7.05–7.08 (m, 2H, Ar-H), 7.61 (d, 1H, *J* = 7.6 Hz, Ar-H), 7.69 (d, 1H, *J* = 7.2 Hz, Ar-H), 7.90 (d, 1H, *J* = 8 Hz, Ar-H), 8.22 (s, 1H, Ar-H), 9.10 (s, 1H, NH, D_2_O exchangeable), 10.16 (s, 1H, NH, D_2_O exchangeable). ^13^C NMR (100 MHz, DMSO-*d*_6_), *δ* ppm: 15.0, 24.1, 56.1, 56.2, 56.2, 97.5, 111.3, 124.0, 124.5, 126.3, 126.7, 127.1, 127.5, 128.2, 130.4, 130.8, 137.5, 146.0, 149.0, 149.4, 151.5, 154.0, 174.3. Anal. calcd for C_23_H_22_ClN_3_O_2_S: C, 62.79; H, 5.04; N 9.55; found: C, 62.51; H, 5.25; N, 9.76.

##### 4-(2-Chloro-8-ethylquinolin-3-yl)-6-(3,4,5-trimethoxyphenyl)-3,4-dihydropyrimidine-2(1*H*)-thione (6c)

Yellow powder, yield 72%, mp 235–237 °C. IR (KBr, cm^−1^): 3165 (NH), 3099 (CH aromatic), 2964 (CH aliphatic), 1130 (C=S). ^1^H NMR (400 MHz, DMSO-*d*_6_), *δ* ppm: 1.29 (t, 3H, *J* = 8.0 Hz, CH_2_CH̲_3_), 3.12–3.18 (q, 2H, *J* = 8.0 Hz, CH̲_2_CH_3_), 3.65 (s, 3H, OCH_3_), 3.80 (s, 6H, 2OCH_3_), 5.51 (d, 1H, CH pyrimidine, *J* = 4.4 Hz), 5.61 (d, 1H, CH pyrimidine, *J* = 4.4 Hz), 6.81 (s, 2H, Ar-H), 7.60 (t, 1H, *J* = 8.0 Hz, Ar-H), 7.69 (d, 1H, *J* = 8 Hz, Ar-H), 7.92 (d, 1H, *J* = 8 Hz, Ar-H), 8.31 (s, 1H, Ar-H), 9.10 (s, 1H, NH, D_2_O exchangeable), 10.16 (s, 1H, NH, D_2_O exchangeable). ^13^C NMR (100 MHz, DMSO-*d*_6_), *δ* ppm: 15.2, 24.1, 53.3, 56.0 (2C), 60.7, 99.4, 103.4 (2C), 126.3, 127.6, 128.2, 128.9, 129.8, 134.6, 135.7, 137.9, 138.6, 141.6, 145.2, 147.3, 153.0 (2C), 177.0. Anal. calcd for C_24_H_24_ClN_3_O_3_S: C, 61.33; H, 5.15; N 8.94; found: C, 61.08; H, 5.32; N, 9.09.

##### 4-(2-Chloro-8-ethylquinolin-3-yl)-6-(4-fluorophenyl)-3,4-dihydropyrimidine-2(1*H*)-thione (6d)

Orange powder, yield 70%, mp 220–222 °C. IR (KBr, cm^−1^): 3167 (NH), 3016 (CH aromatic), 2960 (CH aliphatic), 1197 (C=S). ^1^H NMR (400 MHz, DMSO-*d*_6_), *δ* ppm: 1.29 (t, 3H, *J* = 8.0 Hz CH_2_CH̲_3_), 3.12–3.17 (q, 2H, *J* = 8.0 Hz, CH̲_2_CH_3_), 5.44 (d, 1H, CH pyrimidine, *J* = 4.4 Hz), 5.59 (d, 1H, CH pyrimidine, *J* = 4.4 Hz), 7.19 (t, 2H, *J* = 8.0 Hz, Ar-H), 7.53–7.62 (m, 3H, Ar-H), 7.69 (d, 1H, *J* = 6.8 Hz, Ar-H), 7.90 (d, 1H, *J* = 8 Hz, Ar-H), 8.22 (s, 1H, Ar-H), 9.19 (s, 1H, NH, D_2_O exchangeable), 10.17 (s, 1H, NH, D_2_O exchangeable). ^13^C NMR (100 MHz, DMSO-*d*_6_), *δ* ppm: 16.0, 24.1, 59.2, 97.3, 115.8 (2C), 125.0, 126.6, 127.0, 128.2, 128.7, 129.9, 130.4 (2C), 131.1, 136.5, 145.5, 146.8, 149.7, 162.2, 174.0. Anal. calcd for C_21_H_17_ClFN_3_S: C, 63.39; H, 4.31; N 10.56; found: C, 63.54; H, 4.45; N, 10.78.

##### 6-(4-Bromophenyl)-4-(2-chloro-8-ethylquinolin-3-yl)-3,4-dihydropyrimidine-2(1*H*)-thione (6e)

Orange powder, yield 74%, mp 248–240 °C. IR (KBr, cm^−1^): 3162 (NH), 3018 (CH aromatic), 2964 (CH aliphatic), 1197 (C=S). ^1^H NMR (400 MHz, DMSO-*d*_6_), *δ* ppm: 1.29 (t, 3H, *J* = 8.0 Hz, CH_2_CH̲_3_), 3.12–3.19 (m, 2H, CH̲_2_CH_3_), 5.50 (d, 1H, CH pyrimidine, *J* = 4.4 Hz), 5.60 (d, 1H, CH pyrimidine, *J* = 4.4 Hz), 7.46 (d, 2H, *J* = 8.4 Hz, Ar-H), 7.54–7.62 (m, 3H, Ar-H), 7.69 (d, 1H, *J* = 7.2 Hz, Ar-H), 7.90 (d, 1H, *J* = 7.6 Hz, Ar-H), 8.25 (s, 1H, Ar-H), 9.18 (s, 1H, NH, D_2_O exchangeable), 10.20 (s, 1H, NH, D_2_O exchangeable). ^13^C NMR (100 MHz, DMSO-*d*_6_), *δ* ppm: 15.0, 24.5, 57.9, 96.5, 123.1, 123.8, 124.5, 129.1 (2C), 129.8, 130.5, 132.6 (2C), 133.3, 137.2, 139.7, 140.8, 148.9, 152.8, 153.5, 174.4. Anal. calcd for C_21_H_17_BrClN_3_S: C, 54.98; H, 3.73; N 9.16; found: C, 54.87; H, 3.90; N, 9.35.

### 4.2. Biology

#### 4.2.1. *In vitro* cytotoxic activity screening

The cytotoxic activity of the synthesized quinoline derivatives was determined by the national cancer institute (NCI, Bethesda, Maryland, USA) against 60 different cancer cell lines at a single high dose (10^−5^ M) in the full NCI 60 cell panel using the sulforhodamine B assay for cell viability evaluation. In summary, 5 × 10^3^ cells were in 96-well plate and incubated in complete media for 24 h. Cells were treated with 100 μL of media containing each newly synthesized compound. After drug exposure, cells were fixed by replacing media with 150 μL of 10% TCA and incubated at 4 °C for 1 h. The TCA solution was removed, and the cells were washed 5 times with distilled water. Aliquots of 70 μL SRB solution (0.4% w/v) were added and incubated in a dark place at room temperature for 10 min. The plates were washed 3 times with 1% acetic acid and allowed to air-dry overnight. Then, 150 μL of TRIS (10 mM) was added to dissolve protein-bound SRB stain. The absorbance was measured at 540 nm.% Cytotoxicity = [1 − (AV_*x*_/AV_NC_)] × 100where AV_*x*_ denotes the average absorbance of the sample well and AV_NC_ denotes the average absorbance of the negative control.

##### 4.2.1.1. Five doses testing of compound 4c

The methodology of the NCI anticancer screening has been described in details at (http://www.dtp.nci.nih.gov). The anticancer assay was performed in full NCI 60 cell lines derived from nine tumor subpanels, including leukemia, lung, colon, CNS, melanoma, ovarian, renal, prostate, and breast cancer cell lines.

The cell lines were first cultured for 48 h, after being exposed to a single dosage of the compound. Using the protein-binding dye sulforhodamine B dye, cell proliferation and viability were evaluated (SRB assay). The results were presented for each chemical as GI%. Compound that met NCI criteria for substantial activity were further evaluated using a five-dose experiment. The NCI60 cell lines were treated with the chemicals in this assay at five different concentration levels (0.01, 0.1, 1, 10, and 100 μM). Following cell line incubation, the results were depicted using three parameters: GI_50_, which signifies the molar concentration resulting in a 50% reduction in all cell count after incubation; TGI, indicating the molar concentration leading to total growth inhibition; and LC_50_, representing the molar concentration causing a 50% loss of the initial cells after incubation.

#### 4.2.2. Cell cycle analysis by flow cytometry

Propidium Iodide Flow Cytometry Kit for Cell Cycle Analysis (Abcam) was used according to manufacturer's instructions. Briefly, MDA-MB 231 cells (2 × 10^5^ cells) were seeded in 6-well plates and cultured for 24 h. The cells were treated with IC_50_ of the synthesized compound for 24 h. Cells were collected, washed with PBS and centrifuged. Subsequently, the cells were re-suspended and fixed in 66% ethanol on ice for at least 2 h. The cells were stained with a working solution containing propidium iodide (PI) in the dark at 37 °C for 30 min. DNA content of the cells were measured by flow cytometry using FACSCalibur (BD Biosciences, Mountain View, CA). The data were analyzed using the CellQuest software (BD Biosciences).^21^

#### 4.2.3. Cell apoptosis analysis

Annexin-V-FITC Apoptosis Detection Kit (Biovision, USA) was used to determine apoptotic cells according to manufacturer's instructions. Briefly, MDA-MB 231 cells (2 × 10^5^ cells) were seeded in 6-well plates and cultured for 24 h. The cells were treated with IC_50_ of the synthesized compound for 24 h. Cells were collected, washed with PBS and centrifuged. The cells were re-suspended in 500 μL of 1× binding buffer. 5 μL of annexin V-FITC and 5 μL of propidium iodide (PI 50 mg mL^−1^) were added and the cells were incubated in the dark at room temperature for 5 min. The fluorescence of the stained cells was analyzed by flow cytometry using FACSCalibur (BD Biosciences, Mountain View, CA). The data were analyzed using the CellQuest software (BD Biosciences).^53,54^

#### 4.2.4. Tubulin polymerization inhibition assay

Tubulin polymerization inhibition assay was performed using Tubulin Polymerization Assay Kit (Cytoskeleton, USA). 2 mg mL^−1^ tubulin was dissolved in 80 mM PIPES pH 6.9, 2.0 mM MgCl_2_, 0.5 mM EGTA, 1.0 mM GTP and 15% glycerol. A 96-well plate was warmed to 37 °C for 10 minutes. Serial dilutions of the test compounds were prepared. The assay components were mixed. Polymerization was monitored using a plate reader at 360 nm and emission at 420 nm at 37 °C every 1 min intervals for 60 min. A dose–response curve was generated and the IC_50_ for tubulin polymerization inhibition was calculated.

#### 4.2.5. Tubulin expression assay

##### 4.2.5.1. RNA isolation

The cells were seeded in a six-well plate at a concentration of 3 × 10^5^ cells per well. After 24 h. incubation, the IC_50_ dose of compound 4c and the positive control colchicine was applied to each well for 48 h. The cells were washed, collected and centrifuged at 2500 rpm for 15 min at 4 °C. The total RNA isolation kit (RNeasy extraction kit – QIAGEN) was used to isolate RNA according to the manufacturer's instructions.

##### 4.2.5.2. Gene expression analysis by quantitative real-time PCR

Gene expression of β-tubulin was determined using iScript One-Step RT-PCR kit with SYBR Green (BIORAD) according to the manufacturer protocol. Primers sequences used for qPCR are illustrated in (Table 5). The genes expression was normalized to β-actin as the housekeeping gene. The protocol used was cDNA synthesis for 10 min at 50 °C, iScript Reverse transcriptase inactivation for 5 min at 95 °C and PCR cycling and detection (30 to 45 cycles): 10 s at 95 °C and 30 s at 55 °C to 60 °C.

**Table 5:** Primers sequences of different genes

Gene	Primer sequence
β-Tubulin	F: 5′-TCAGCGTCTACTACAACGAGGC-3′
	R: 5′-GCCTGAAGAGATGTCCAAAGGC-3′
β-Actin	F: 5′-CATTGCTGACAGGATGCAGAAGG-3′
	R: 5′-TGCTGGAAGGTGGACAGTGAGG-3′

### 4.3. *In silico* studies

#### 4.3.1. Docking studies

**Fig. 16:**
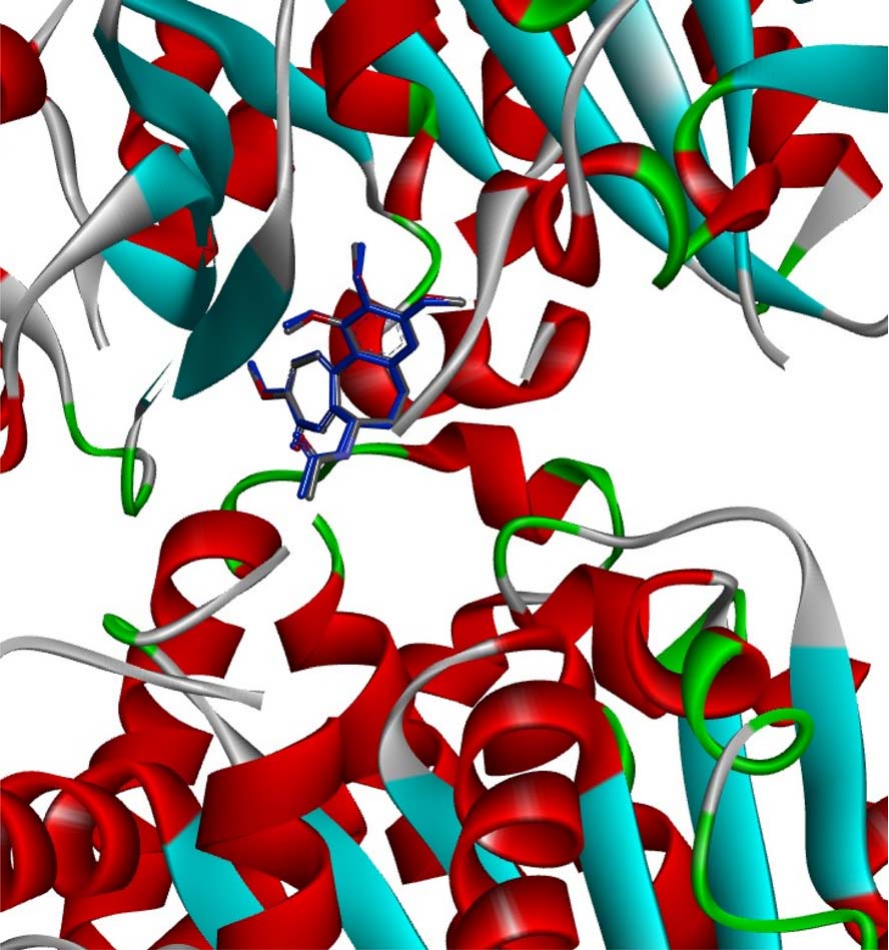
Alignment between the conformation of the co-crystallized ligand (colchicine) colored in grey and the best-docked pose (colored in blue) into α–β tubulin enzyme with RMSD value equal to 0.17 Å.

The docking simulation was performed to ascertain the fit of the titled compounds 3b, 3c, 4c, and 5c in the active site of the tubulin receptor. Docking was performed on AutoDock software using the Vina plugin.^55,56^ Protein structure was downloaded from PDB (PDB ID: 4O2B with a resolution of 2.30 Å). The simulation started with the preparation of protein through the removal of all the subunits except chain A (α-tubulin subunit) and chain B (β-tubulin subunit) having the colchicine binding site (CBS), water was removed, protonation, and addition of Kollman charges was performed, finally, the prepared enzyme, co-crystallized ligand (colchicine, PDB entry: LOC), and titled compounds were saved in pdbqt format. Grid box coordinates were kept at 17.30 × 66.41 × 42.90 Å, while exhaustiveness was kept at 8. The internal ligand of the enzyme was redocked to validate the method and the RMSD value was reported using the DockRMSD server.^57^ The result revealed that the re-docked colchicine showed an RMSD value of 0.17 Å, while the pose of the redocked ligand aligned perfectly with the native ligand (Fig. 16) which revealed a validated docking procedure. After docking completion 2D and 3D images were visualized using DS client software.^58^ The key amino acid residues, bonded atom or group and the interaction energies in kcal mol^−1^ of the new compounds as well as for the co-crystallized ligand were summarized (Table S5†).

#### 4.3.2. Physicochemical properties

The SwissADME web tool was used to estimate physicochemical and pharmacokinetics characteristics *in silico* (SwissADME: http://www.swissadme.ch/).^59^

## Data availability

The authors of this article declare that and data sharing should be upon request.

## Conflicts of interest

There are no conflicts to declare.

## Supplementary Material

RA-014-D4RA04371E-s001
