# Supplementary material for: Exploring the antitumor potential of novel quinoline derivatives via tubulin polymerization inhibition in breast cancer; design, synthesis and molecular docking
Source: RSC Adv. 2024 Jul 12;14(31):22092–112. doi: 10.1039/d4ra04371e (PMC11240139; doi:10.1039/d4ra04371e)
Supplement: RA-014-D4RA04371E-s001 [file RA-014-D4RA04371E-s001.pdf]

Electronic Supplementary Material (ESI) for RSC Advances.
This journal is © The Royal Society of Chemistry 2024

Exploring the Antitumor Potential of Novel Quinoline Derivatives *Via* Tubulin Polymerization Inhibition in Breast Cancer; Design, Synthesis and Molecular Docking

Heba Abdelmegeed [a], Lina M. A. Abdel Ghany [b], Amira Youssef [c], Abd-Allah S. El-Etrawy [c,d], Noha Ryad [c]

[a] *Chemistry of Natural Compounds Department, Pharmaceutical and Drug Industries Research Institute, National Research Centre, Giza 12622, Egypt.*

[b] *Pharmaceutical Chemistry Department, College of Pharmaceutical Sciences and Drug Manufacturing, Misr University for Science and Technology (MUST), 6th of October City, Giza, P.O. Box 77, Egypt.*

[c] *Pharmaceutical Organic Chemistry Department, College of Pharmaceutical Sciences and Drug Manufacturing, Misr University for Science and Technology (MUST), 6th of October City, Giza, P.O. Box 77, Egypt.*

[d] *Department of Chemistry, Basic Science, Misr University for Science and Technology (MUST), 6th of October City, Giza, P.O. Box 77, Egypt.*

---

* Corresponding author: Lina M. A. Abdel Ghany

Email:

Lina.ameen@must.edu.eg

Supplementary data:

The data are divided into three parts; chemistry, biological studies, and docking studies.

Chemicals and reagents:

Solvents and reagents were obtained from Aldrich and were used without further purification unless otherwise indicated. Melting points were determined by the open capillary tube method using the Stuart SMP10 melting point apparatus and were uncorrected. The elemental analysis was carried out by Thermo Scientific TM FLASH 2000 CHNS/O analyzer, by Thermo Fisher Scientific at The Regional Center for Mycology and Biotechnology, Al-Azhar University, Egypt. Infrared Spectra were recorded as potassium bromide discs on Bruker FT-IR spectrophotometer, MUST university and expressed in wave number υ_{max} (cm^{-1}). ^{1}H NMR spectra were performed on Bruker 400 MHz spectrophotometer using TMS as internal standard, chemical shifts(δ) were recorded in ppm on δ scale at Ain Shams University, Egypt. ^{13}C NMR spectra were carried out using Bruker 100 MHz using TMS as internal standard, chemical shifts (δ) were recorded in ppm on δ scale at Ain Shams University, Egypt. Mass spectra were run on Hewlett Packard 5988 spectrometer or Shimadzu QP-2010 plus at The Regional Center for Mycology & Biotechnology, Al-Azhar University, Egypt. Progress of the reactions was monitored by TLC using precoated aluminum sheets silica gel (Merck 60 F_{254}) using chloroform:methanol (9.5:0.5) as the eluting system and was visualized by UV lamp.

Spectral data:

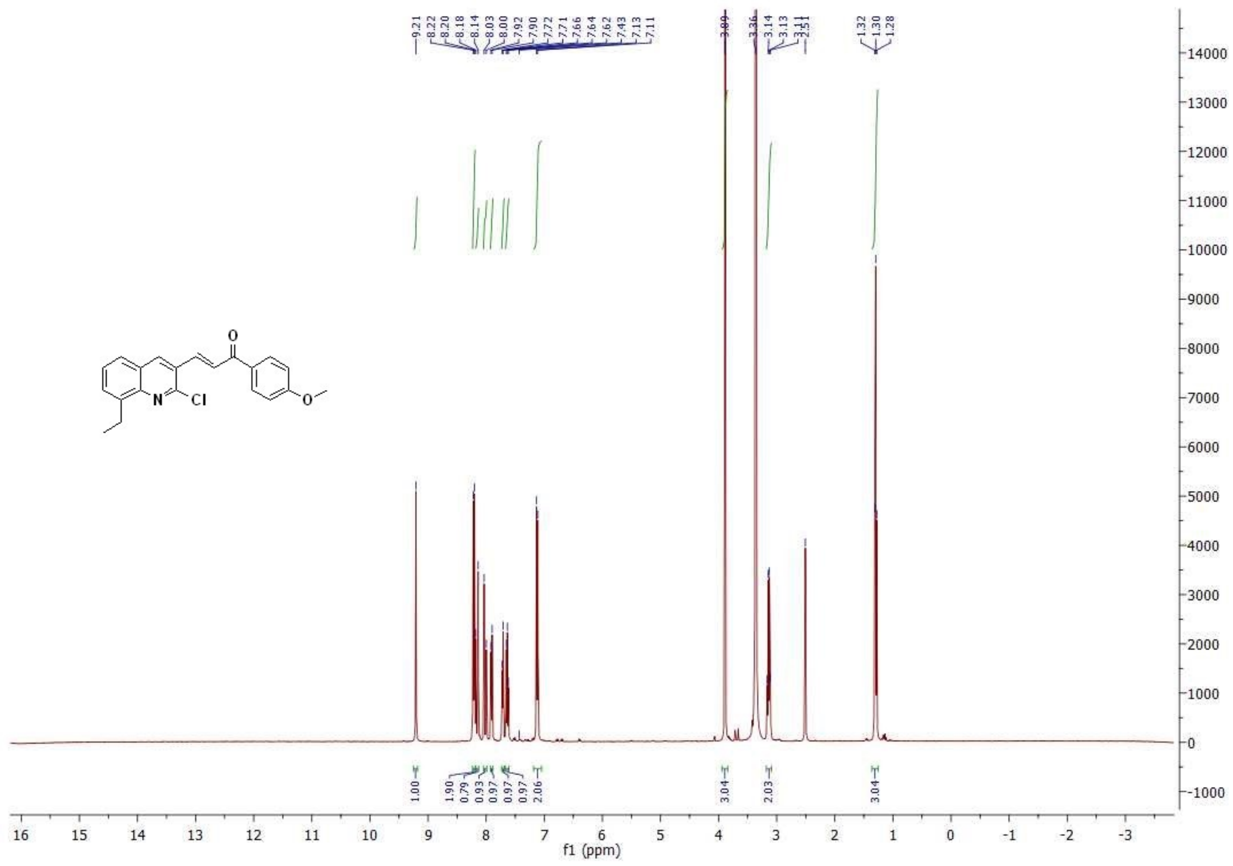

Fig. S1: ^{1}H NMR (400 MHz, DMSO-d_6), spectrum of the compound **3a**.

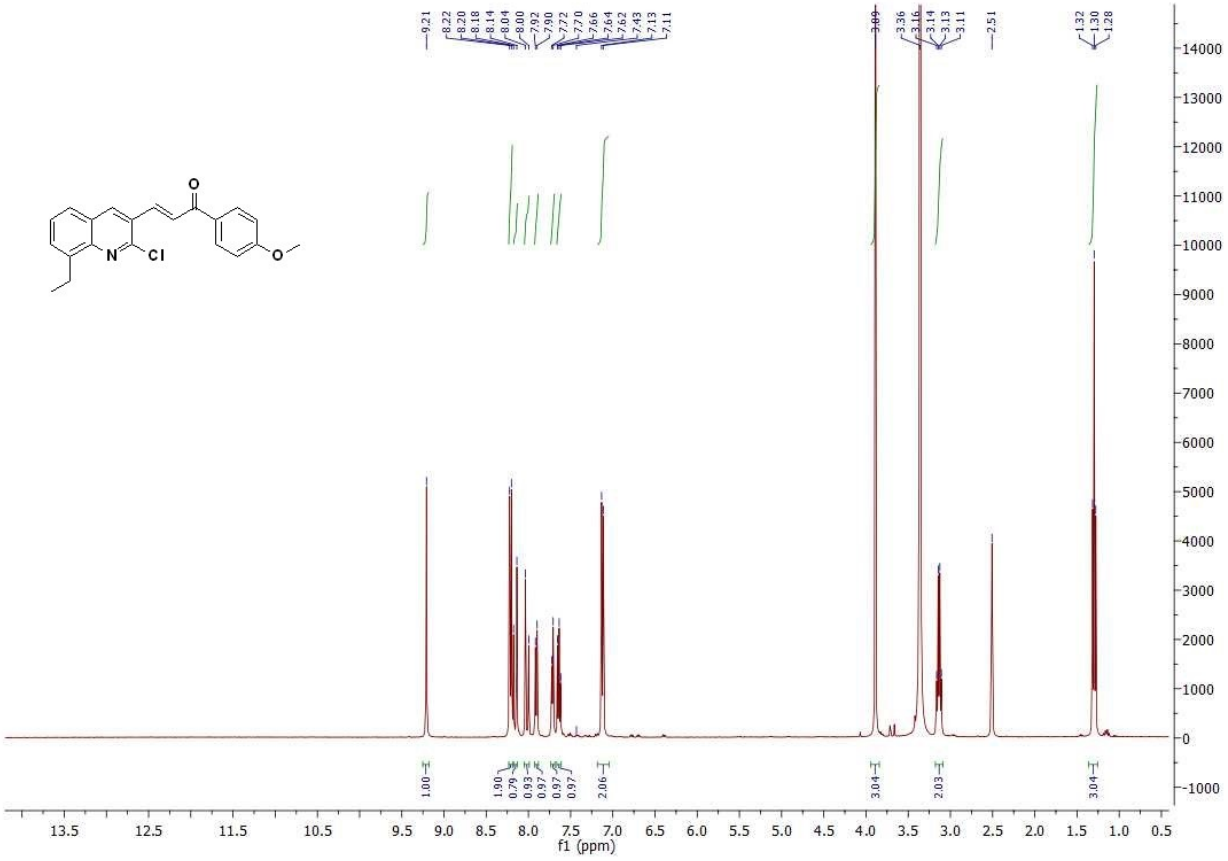

Fig. S2: ^{1}H NMR (400 MHz, DMSO-d_6) magnified spectrum of the compound **3a**.

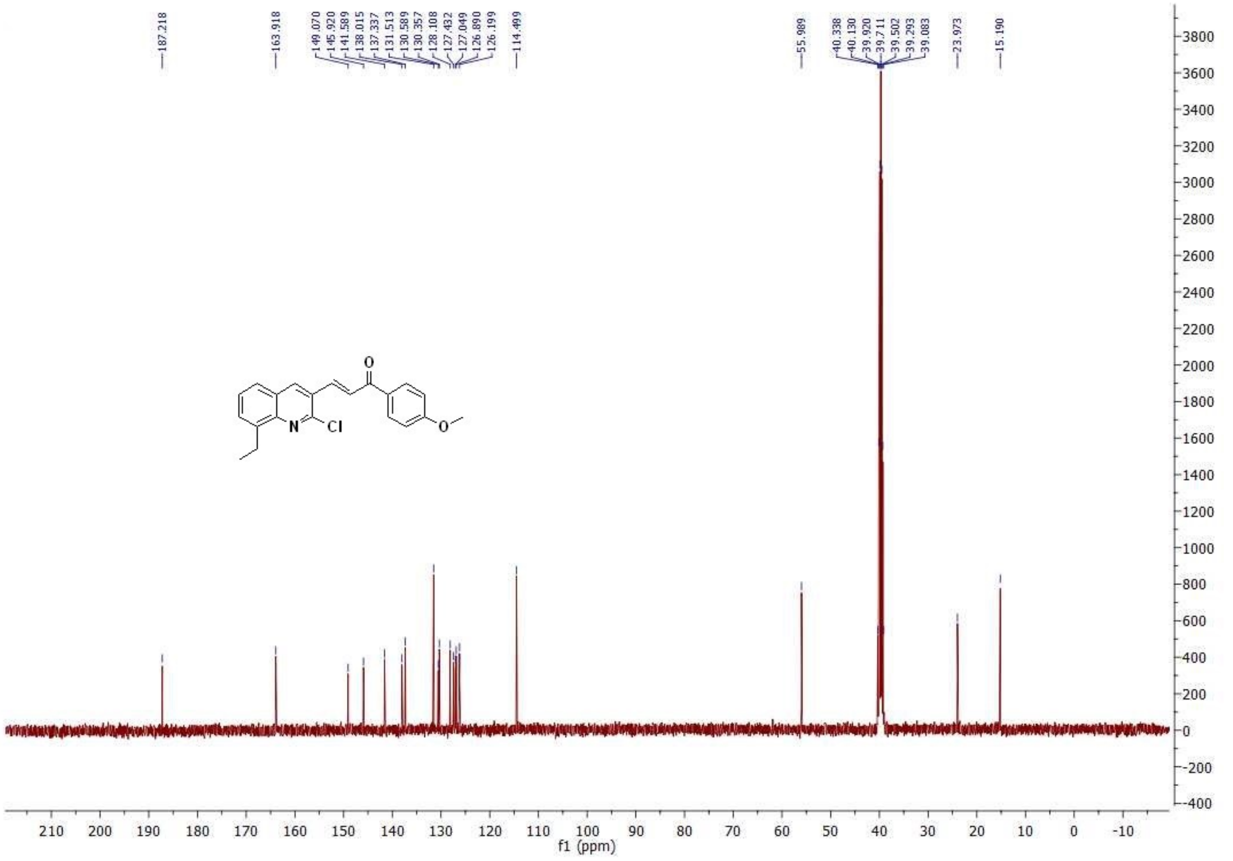

Fig. S3: ^{13}C NMR (100 MHz, DMSO-d_6) spectrum of the compound **3a**.

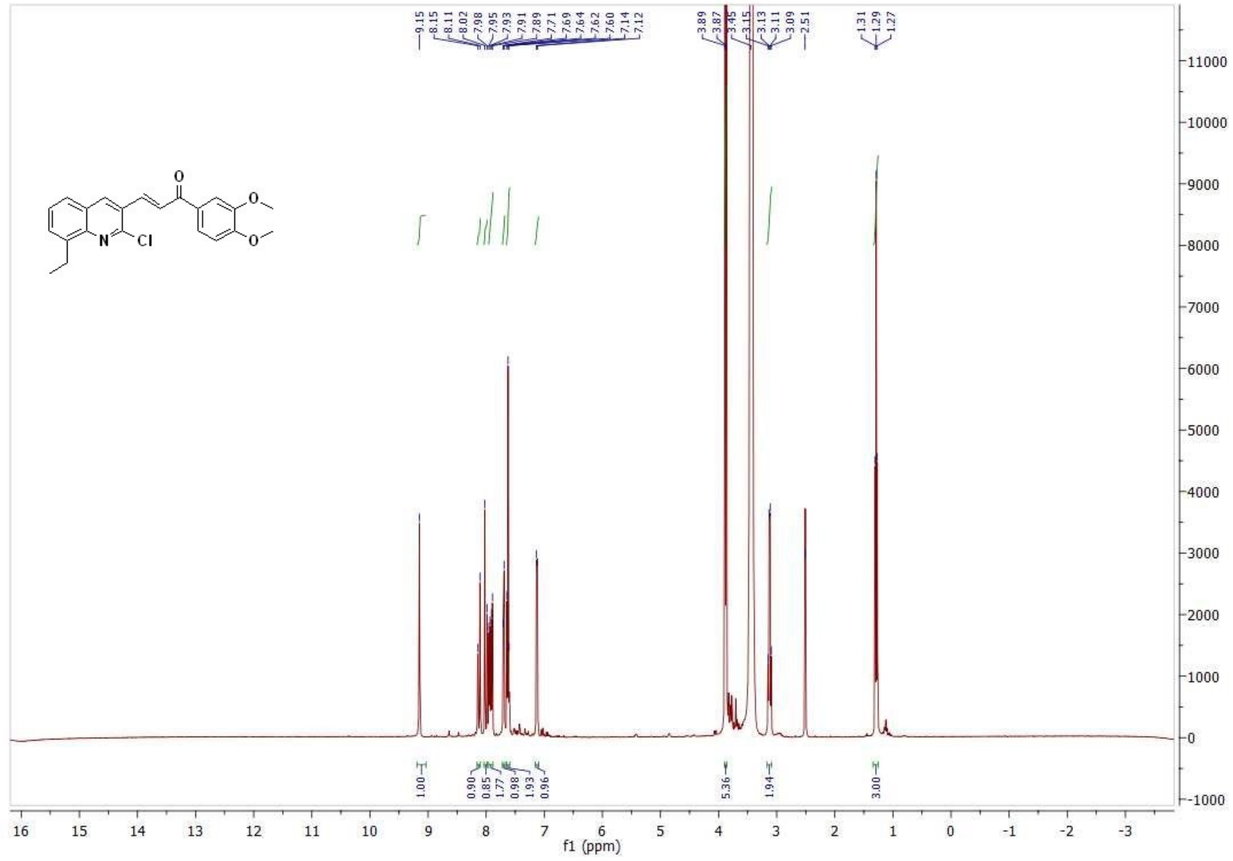

Fig. S4: ^{1}H NMR (400 MHz, DMSO-d_6) spectrum of the compound **3b**.

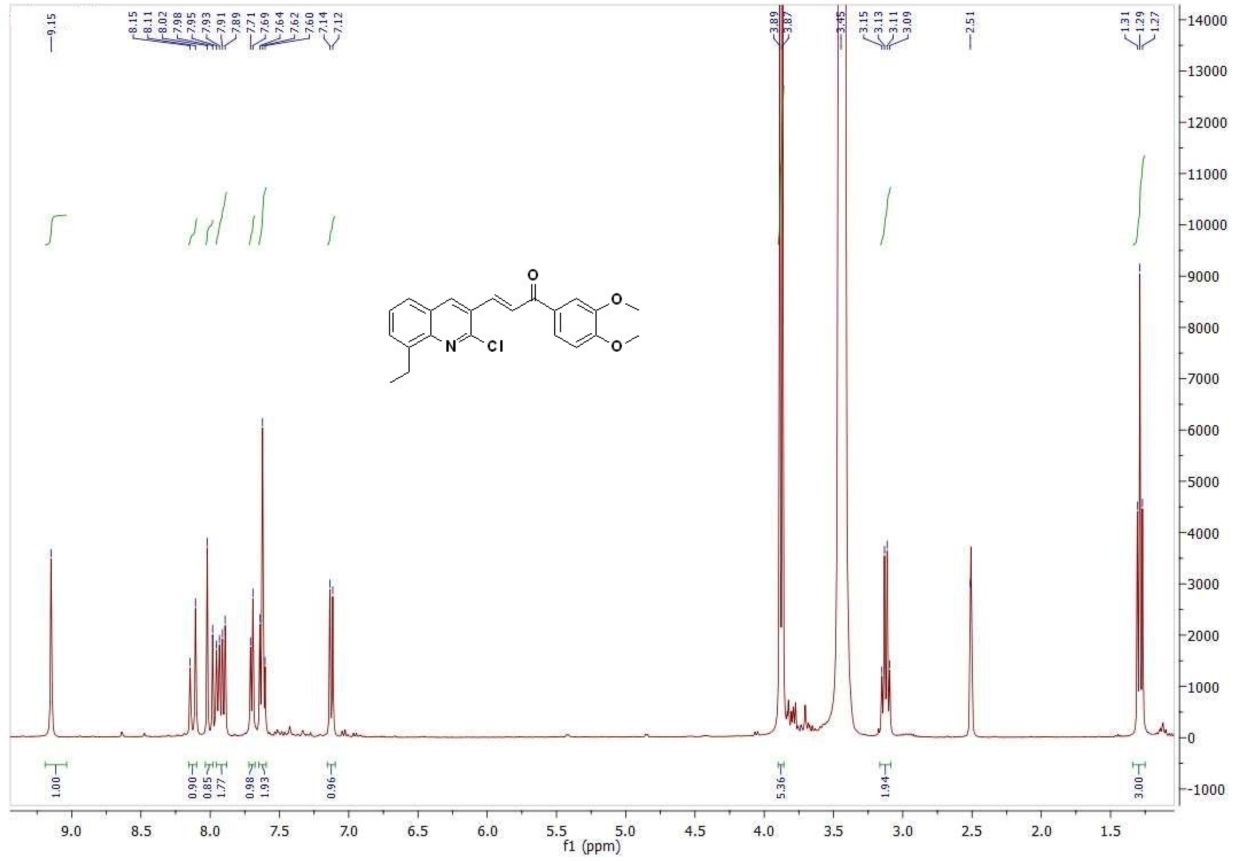

Fig. S5: ^{1}H NMR (400 MHz, DMSO-d_6) magnified spectrum of the compound **3b**.

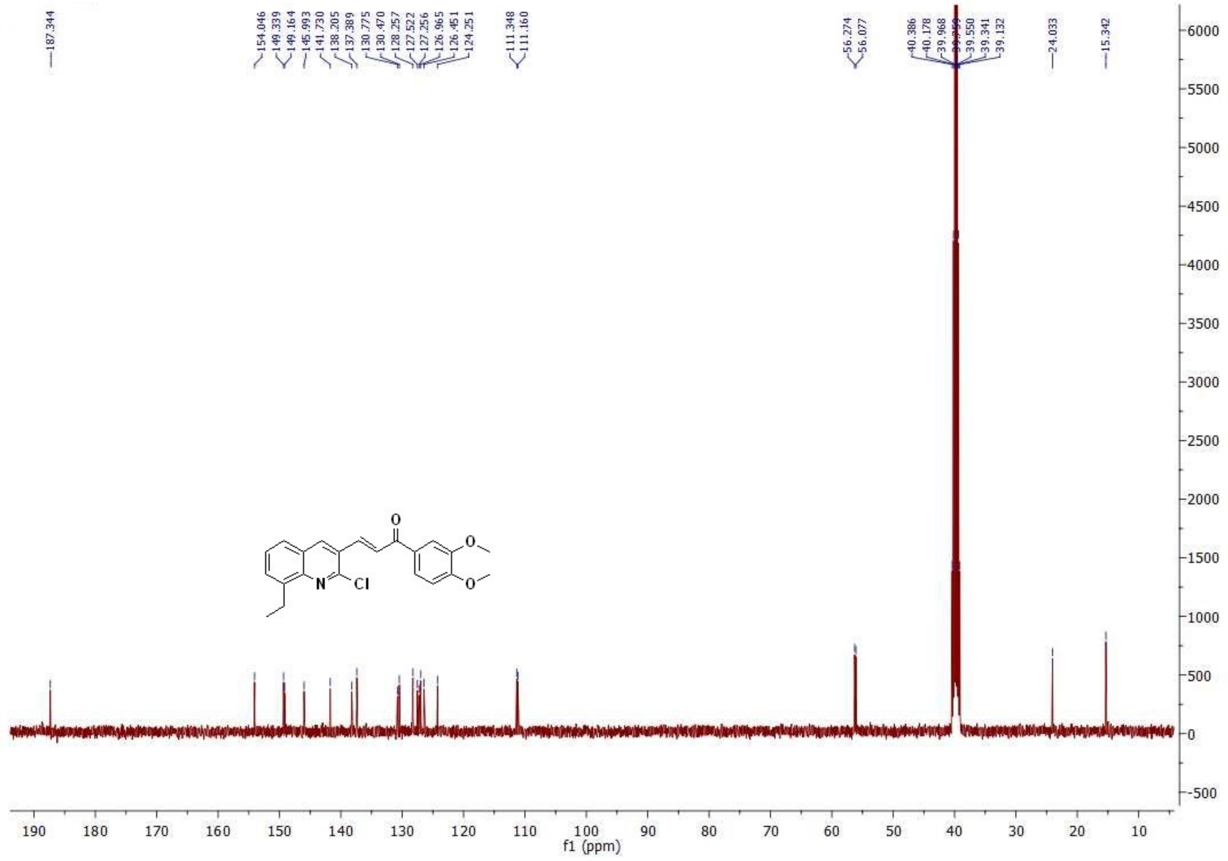

Fig. S6: ^{13}C NMR (100 MHz, DMSO-d_6) spectrum of the compound **3b**.

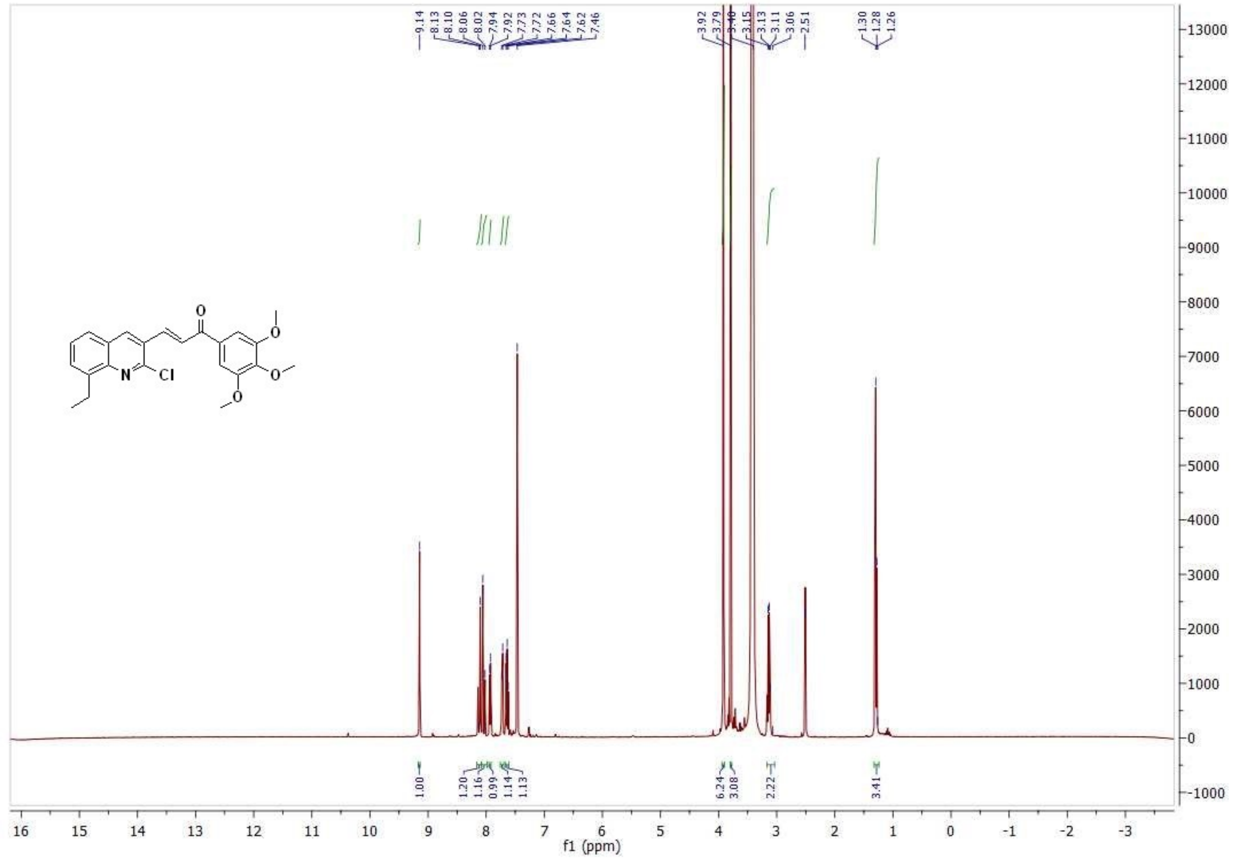

Fig. S7: ^{1}H NMR (400 MHz, DMSO-d_6) spectrum of the compound **3c**.

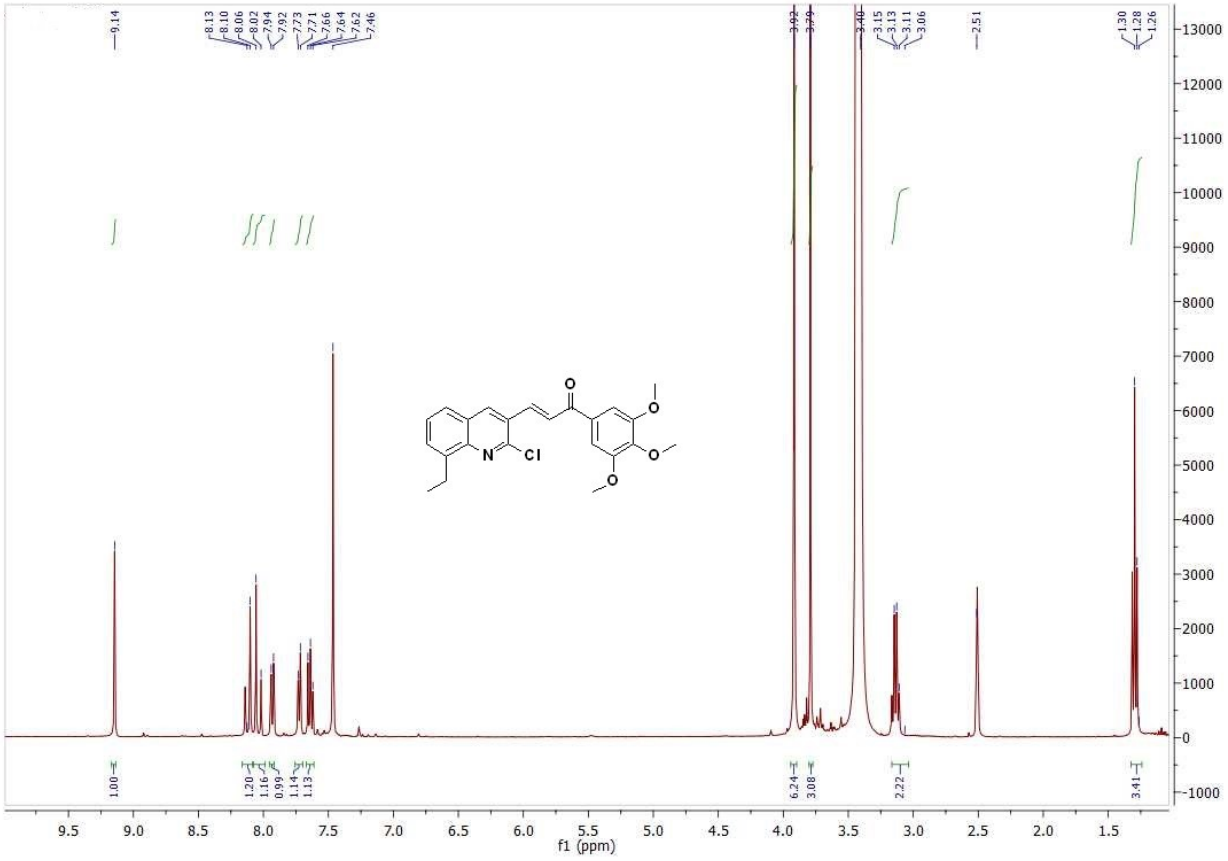

Fig. S8: ^{1}H NMR (400 MHz, DMSO-d_6) magnified spectrum of the compound **3c**.

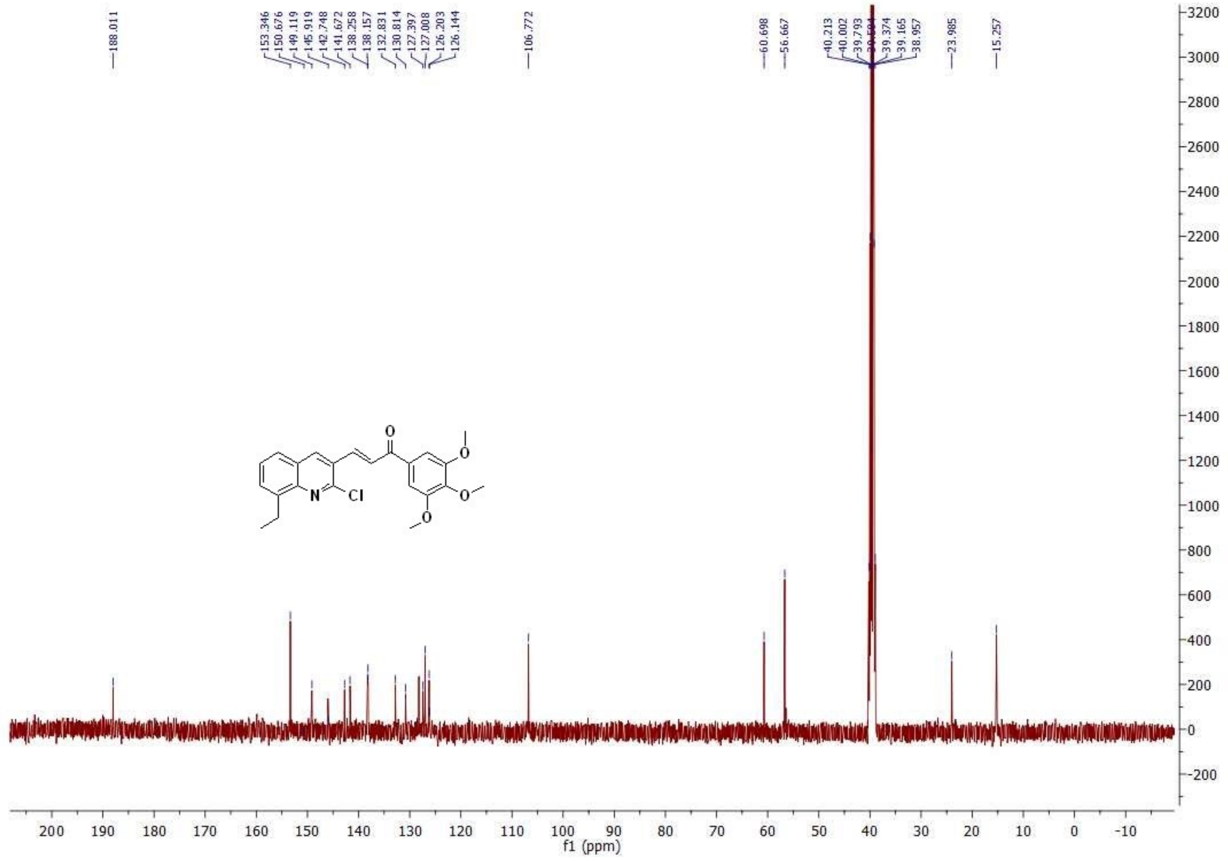

Fig. S9: ^{13}C NMR (100 MHz, DMSO-d_6) spectrum of the compound **3c**.

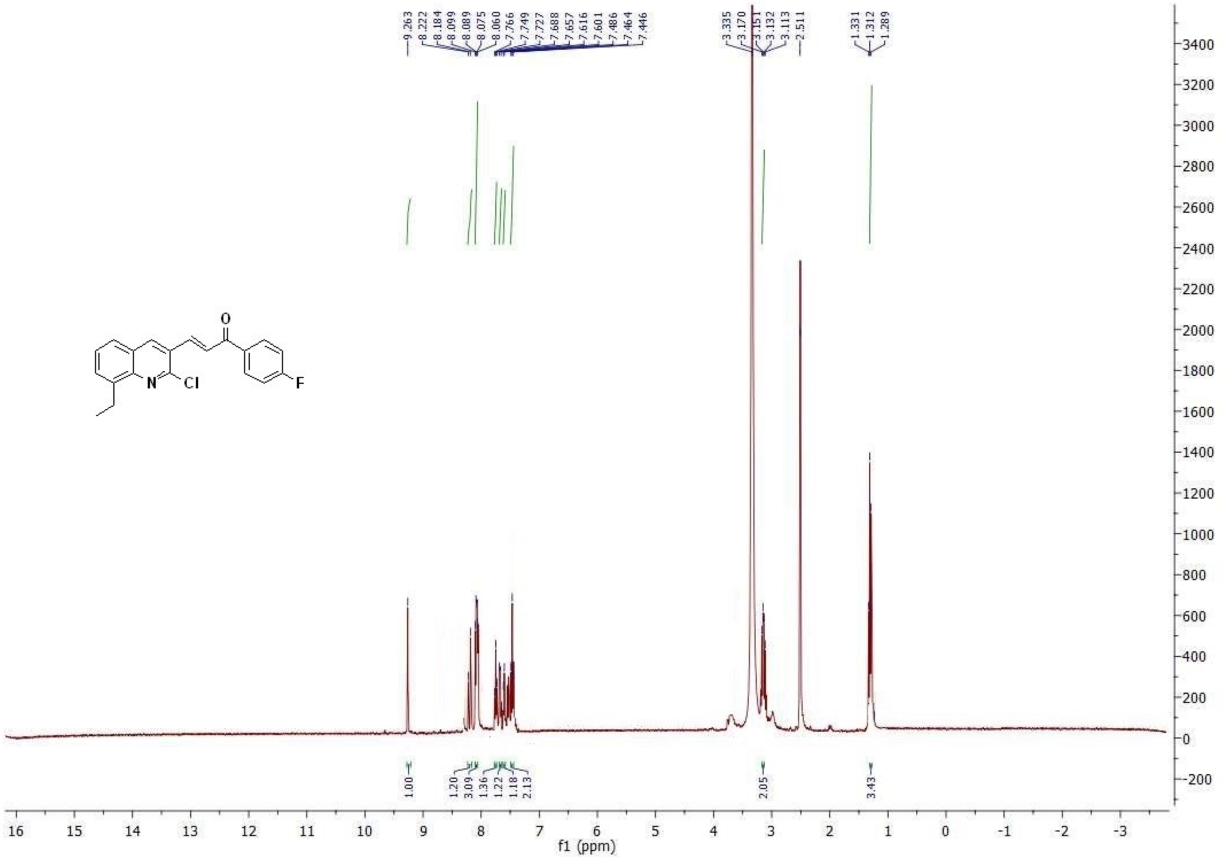

Fig. S10: ^{1}H NMR (400 MHz, DMSO-d_6) spectrum of the compound **3d**.

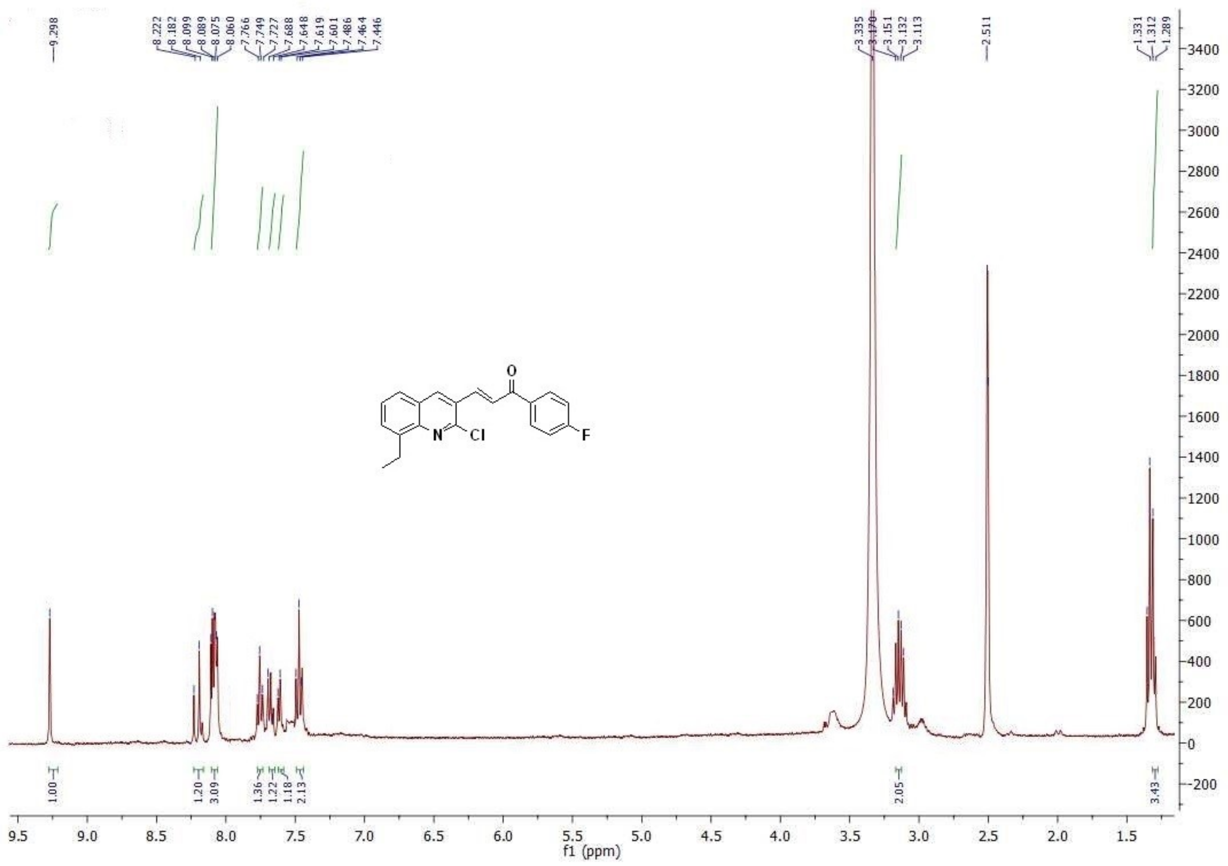

Fig. S11: ^{1}H NMR (400 MHz, DMSO-d_6) magnified spectrum of the compound **3d**.

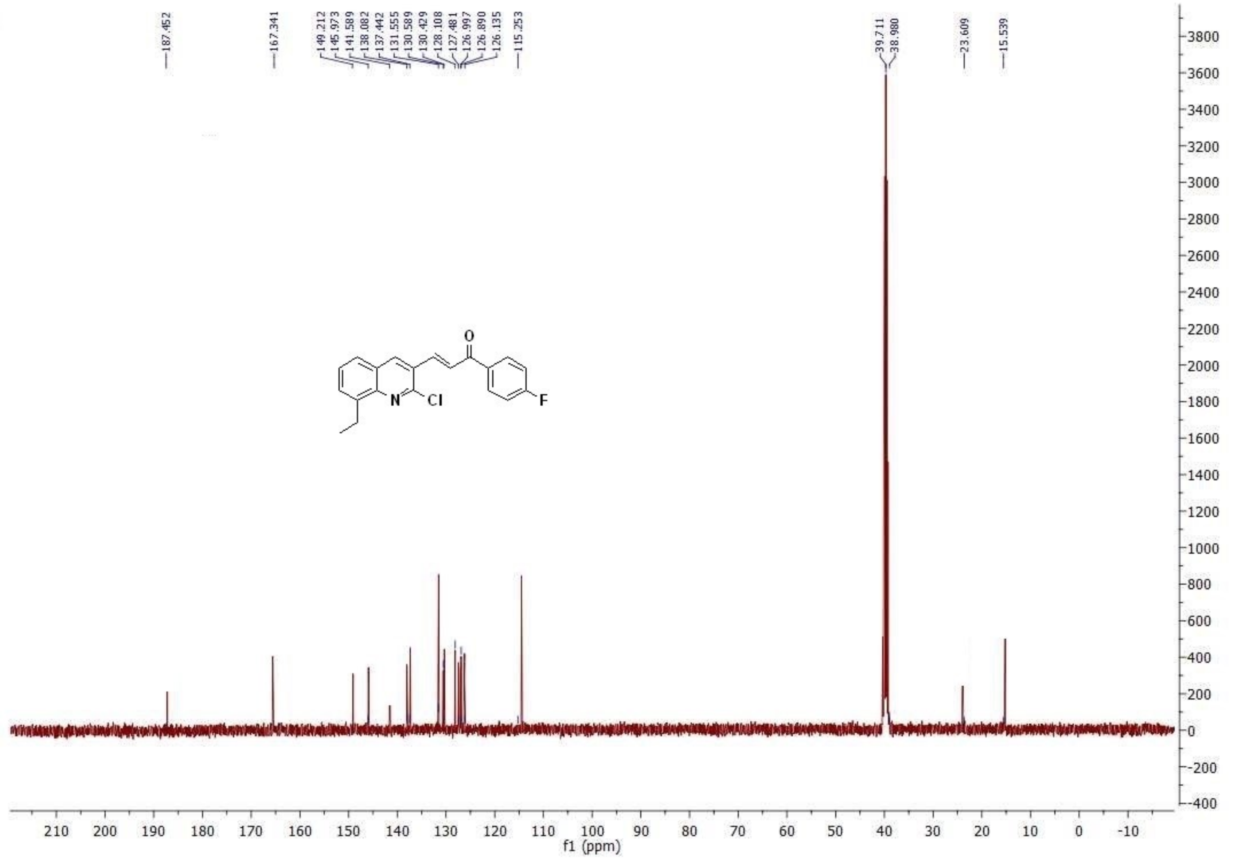

Fig. S12: ^{13}C NMR (100 MHz, DMSO-d_6) spectrum of the compound **3d**.

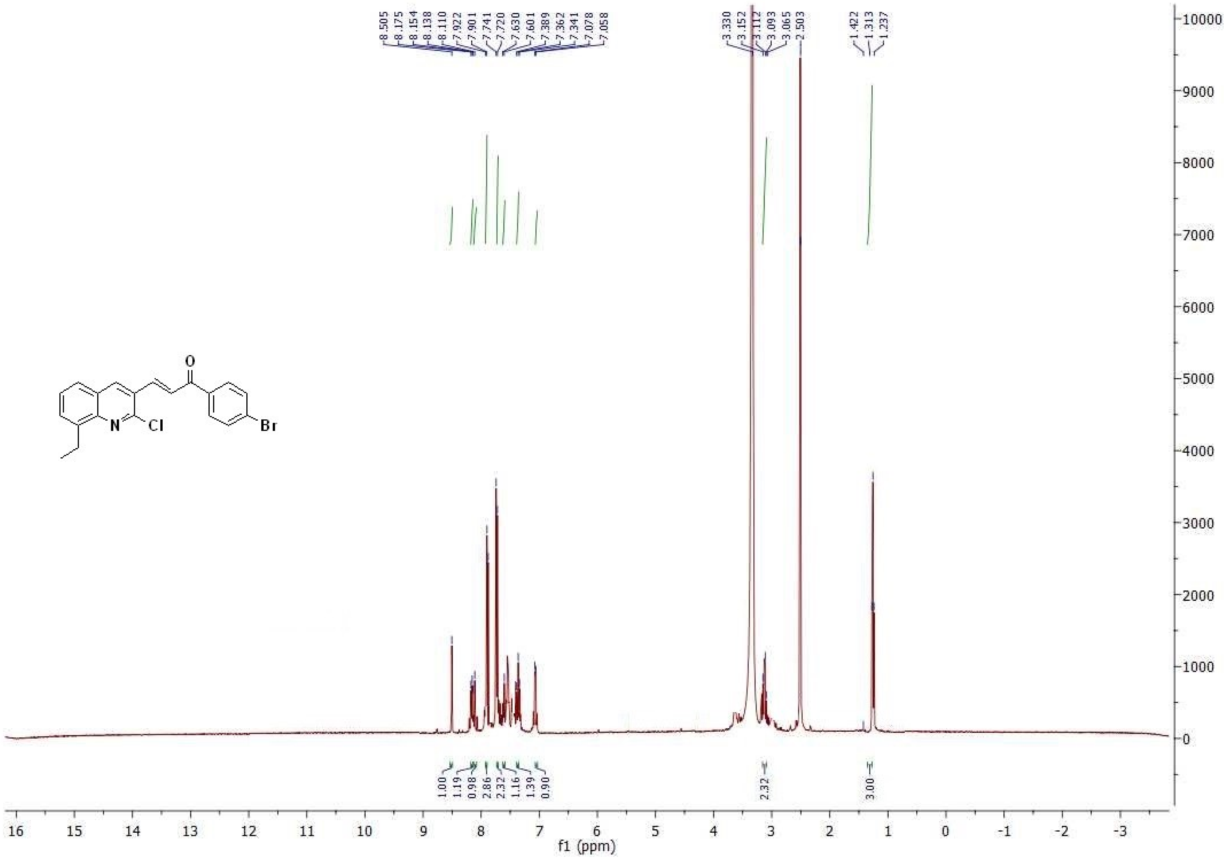

Fig. S13: ^{1}H NMR (400 MHz, DMSO-d_6) magnified spectrum of the compound **3e**.

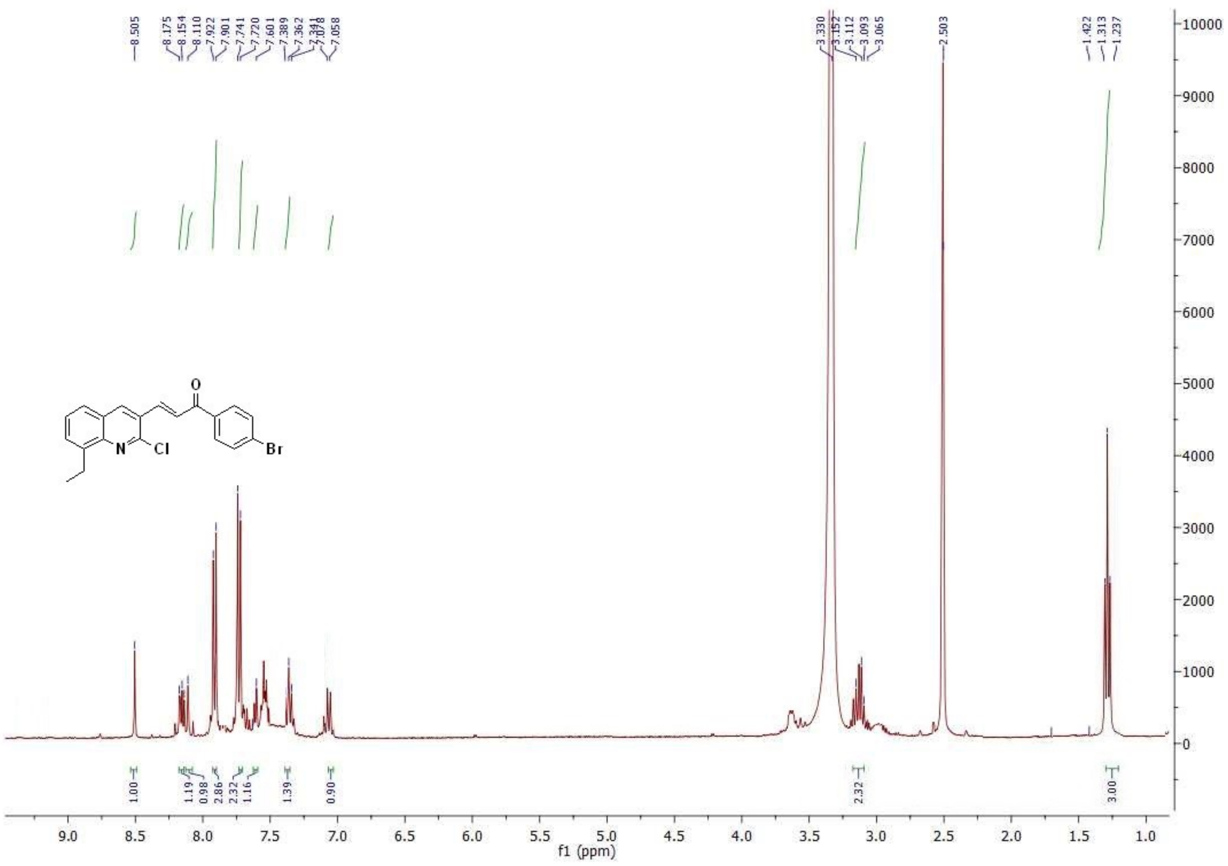

Fig. S14: ^{1}H NMR (400 MHz, DMSO-d_6) magnified spectrum of the compounds **3e**.

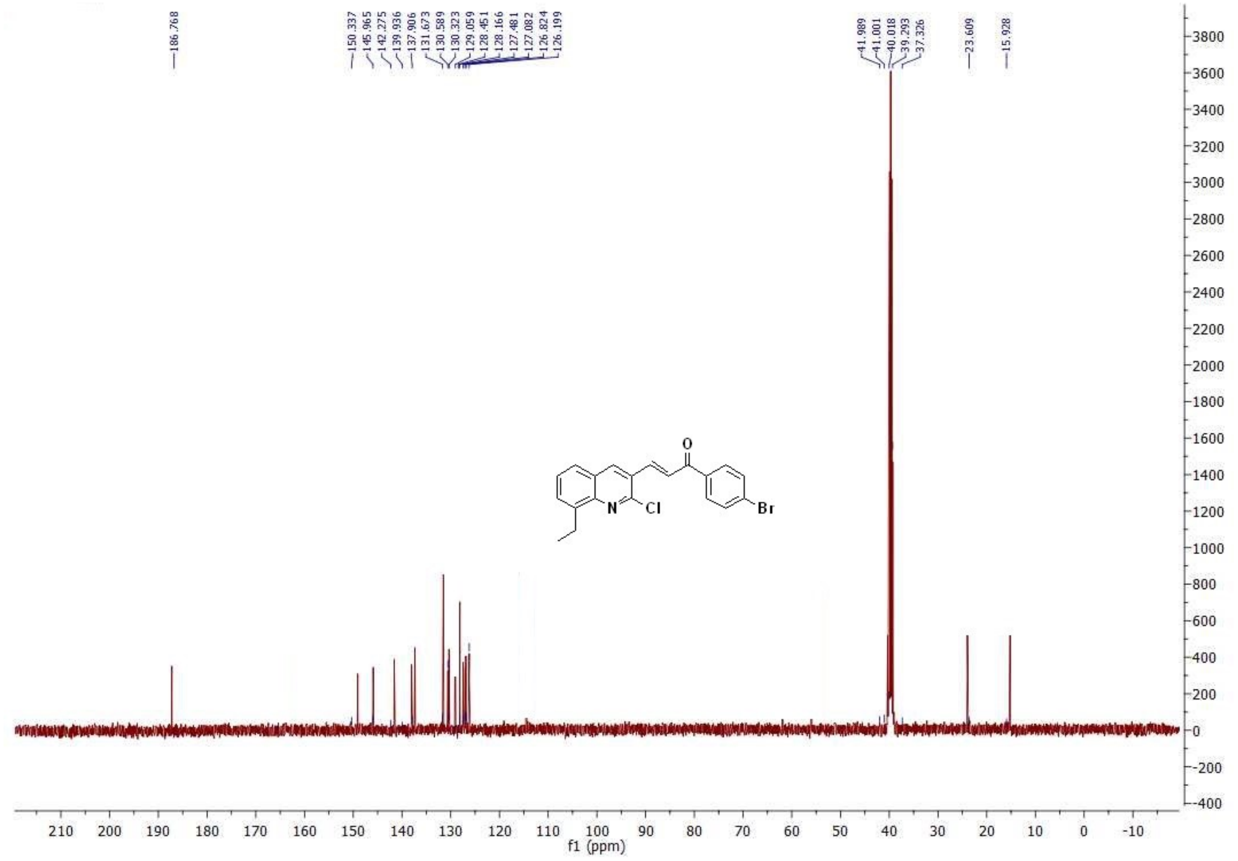

Fig. S15: ^{13}C NMR (100 MHz, DMSO-d_6) spectrum of the compound **3e**.

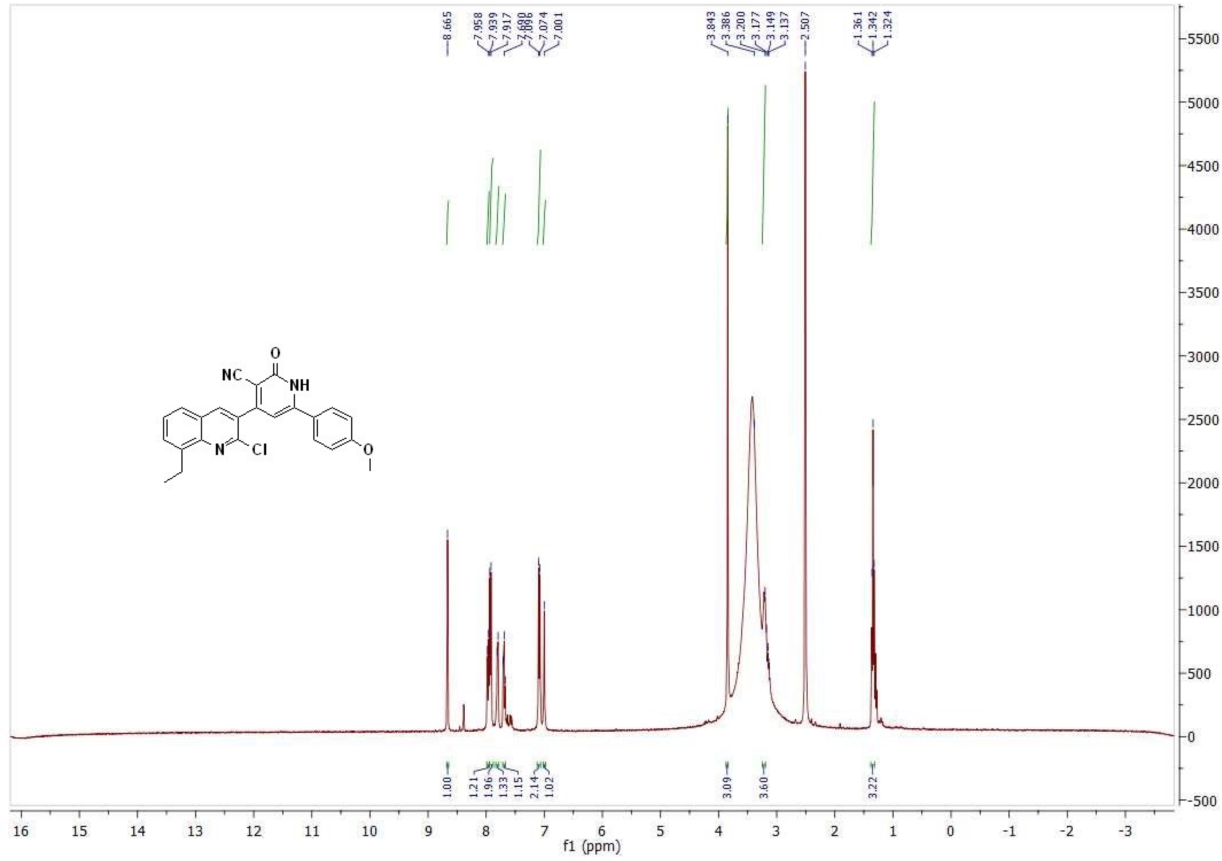

Fig. S16: ^{1}H NMR (400 MHz, DMSO-d_6) spectrum of the compound **4a**.

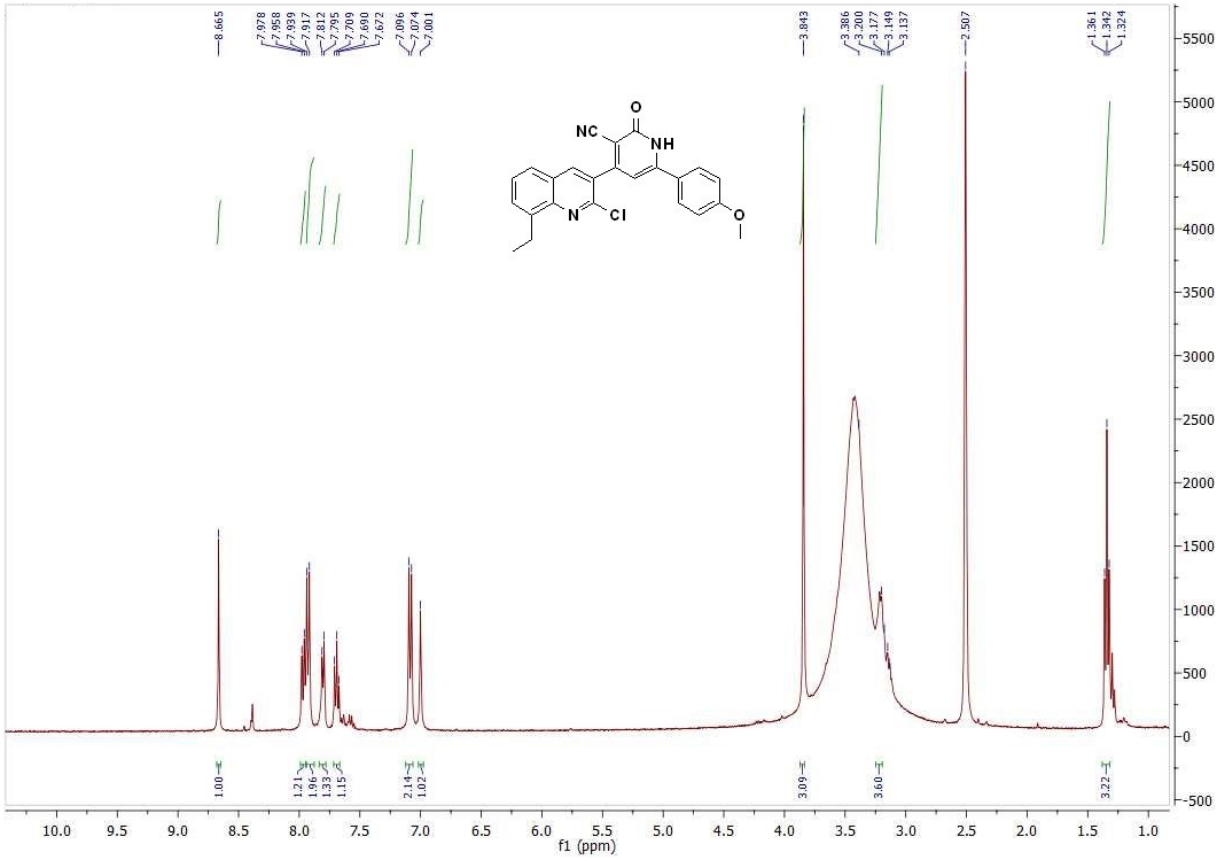

Fig. S17: ^{1}H NMR (400 MHz, DMSO-d_6) magnified spectrum of the compound **4a**.

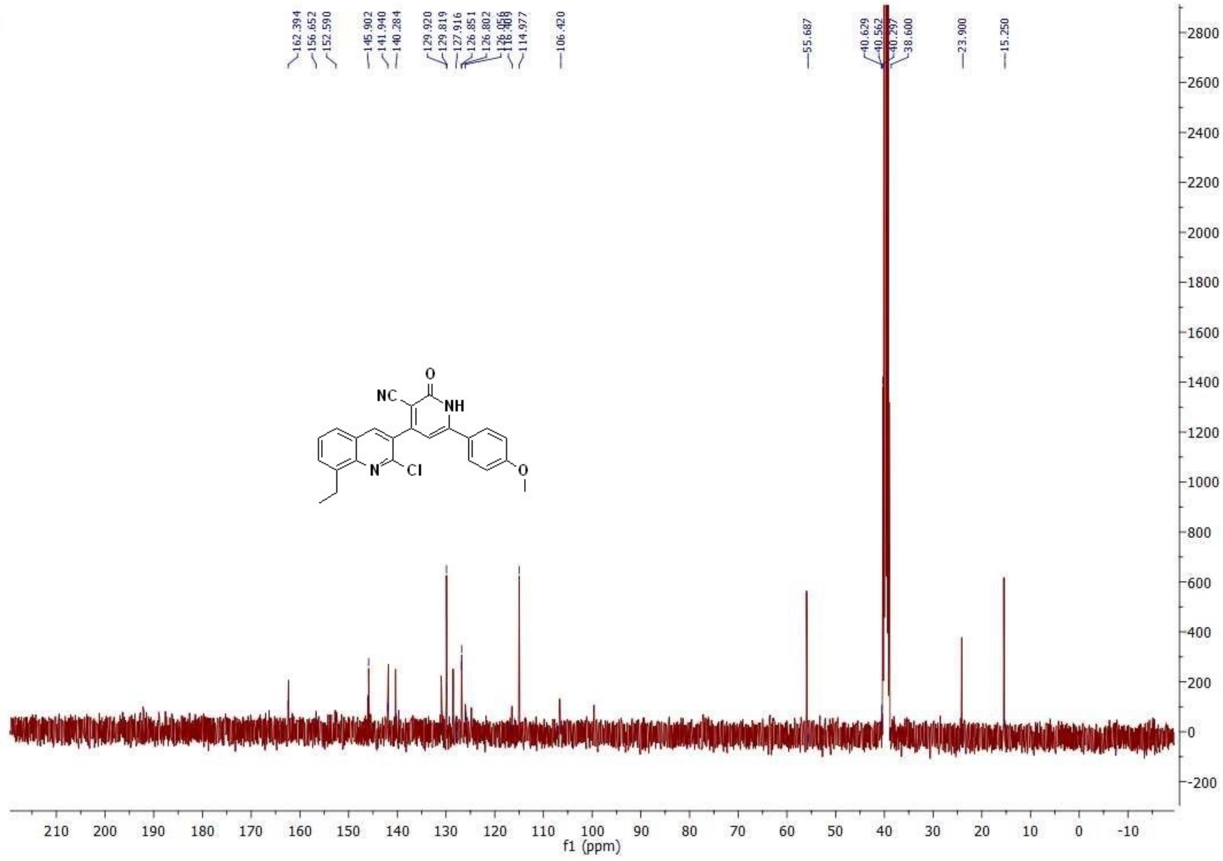

Fig. S18: ^{13}C NMR (100 MHz, DMSO-d_6) spectrum of the compound **4a**.

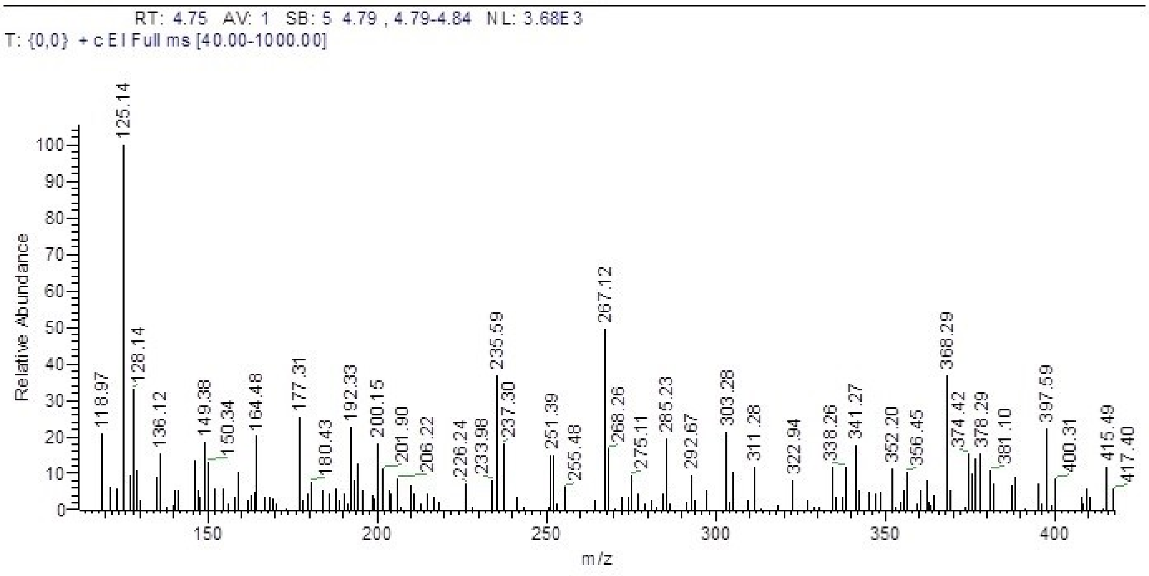

Fig. S19: MS of the compound **4a**.

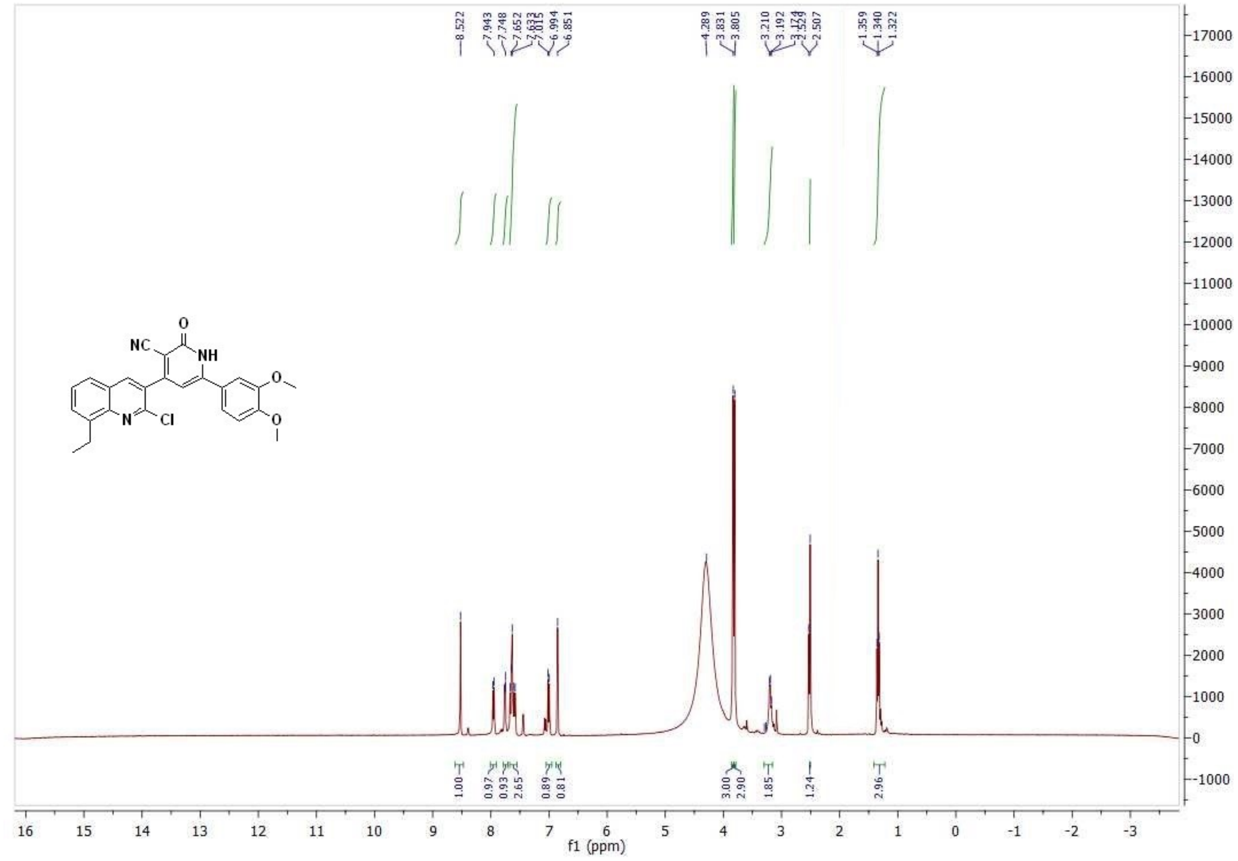

Fig. S20: ^{1}H NMR (400 MHz, DMSO-d_6) spectrum of the compound **4b**.

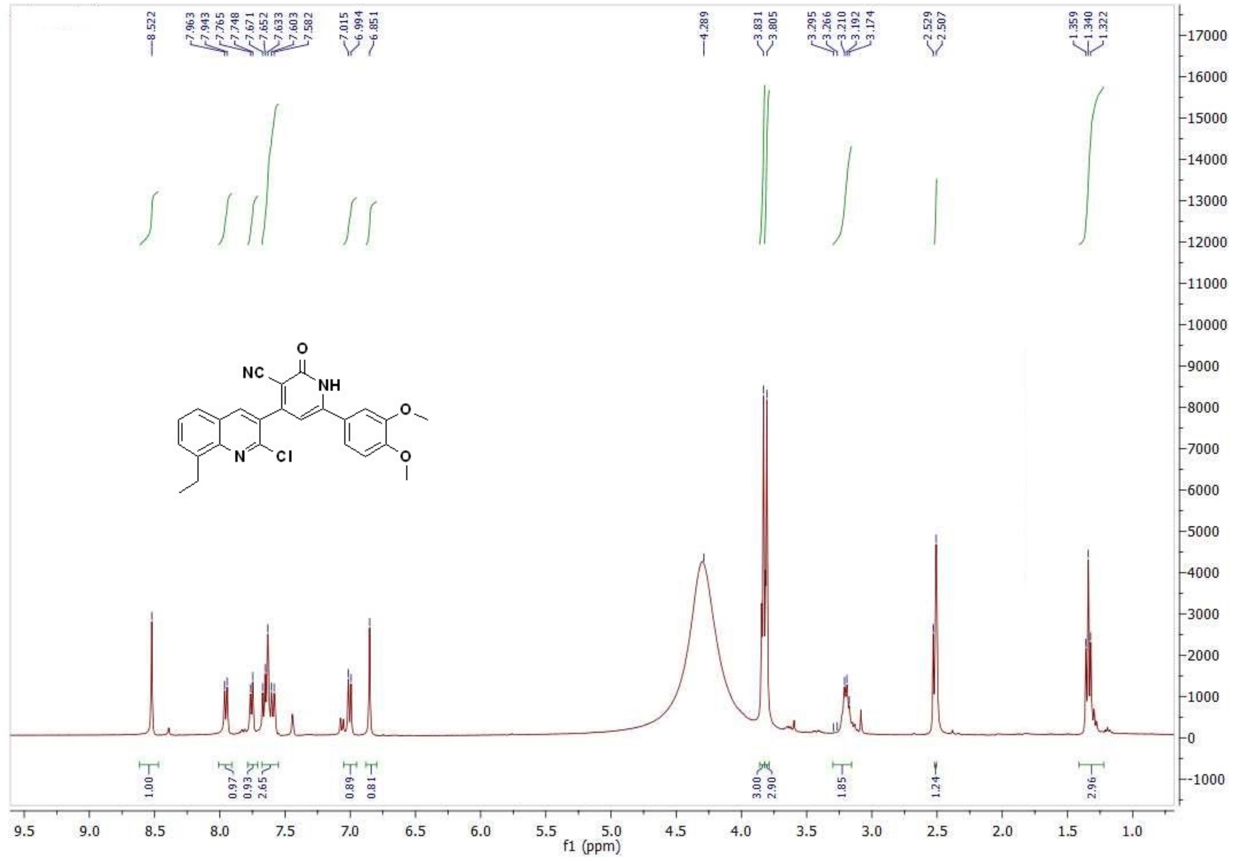

Fig. S21: ^{1}H NMR (400 MHz, DMSO-d_6) magnified spectrum of the compound **4b**.

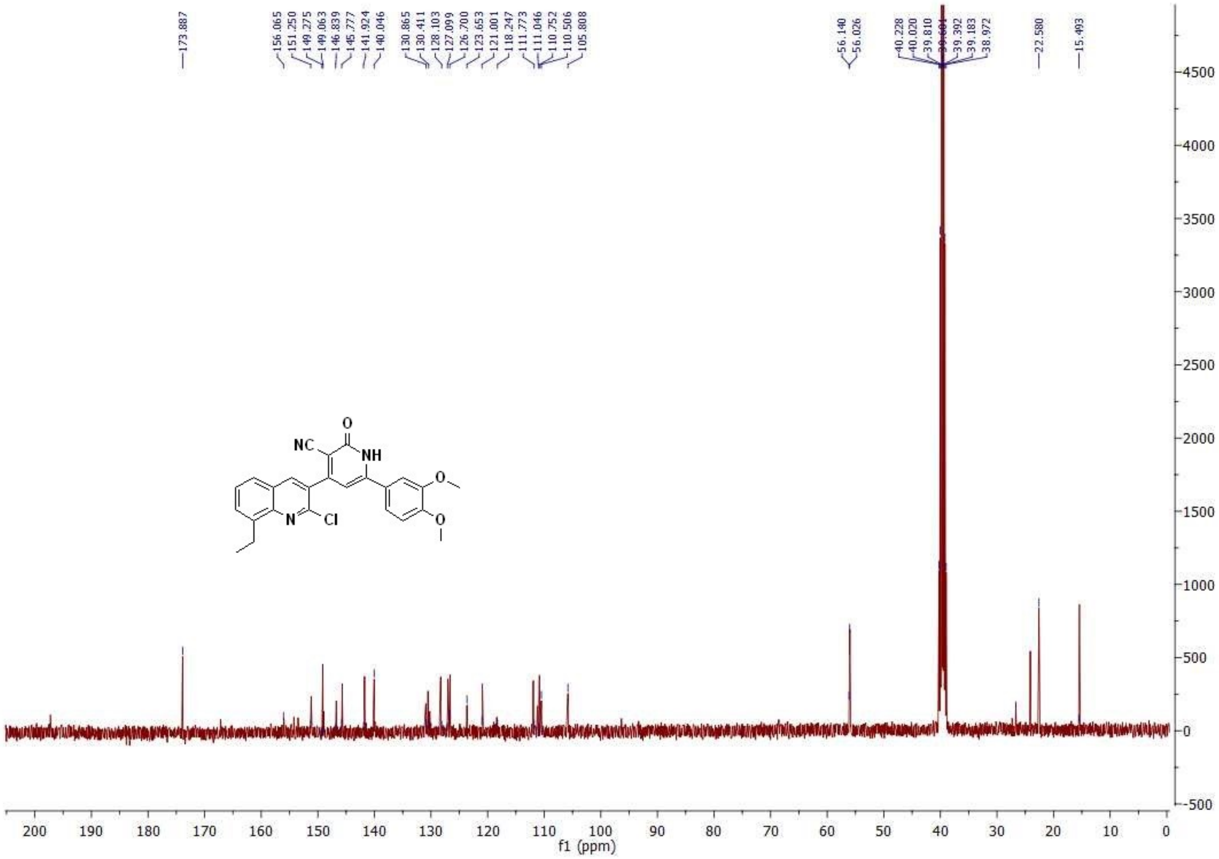

Fig. S22: ^{13}C NMR (100 MHz, DMSO-d_6) spectrum of the compound **4b**.

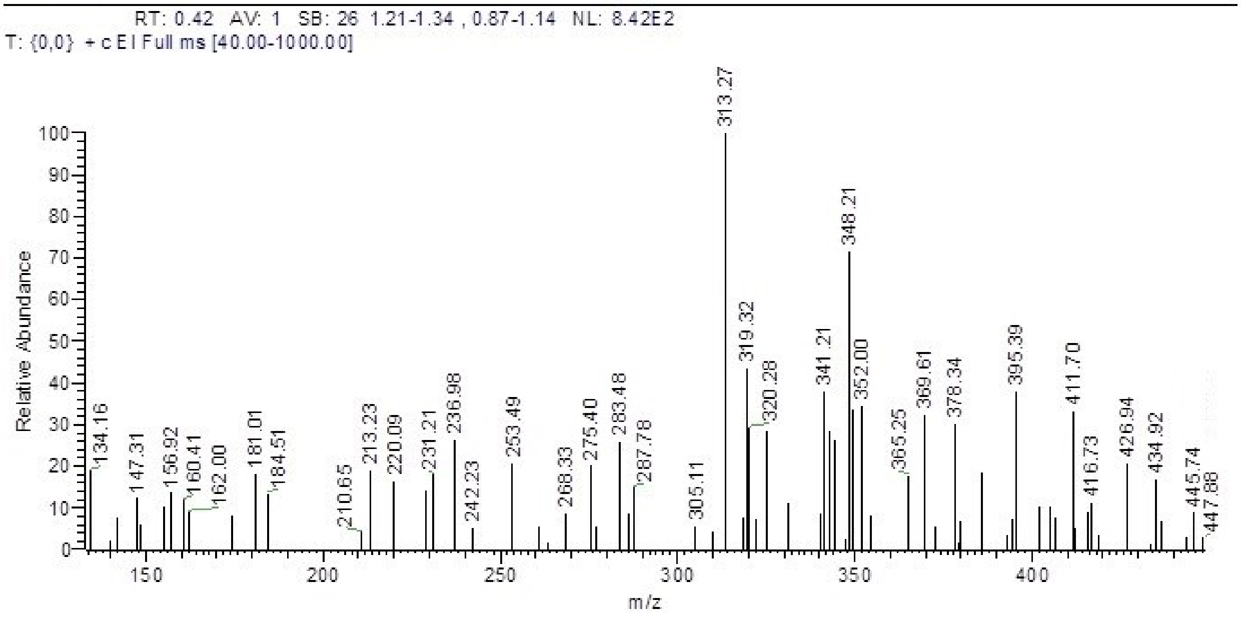

Fig. S23: MS of the compound **4b**.

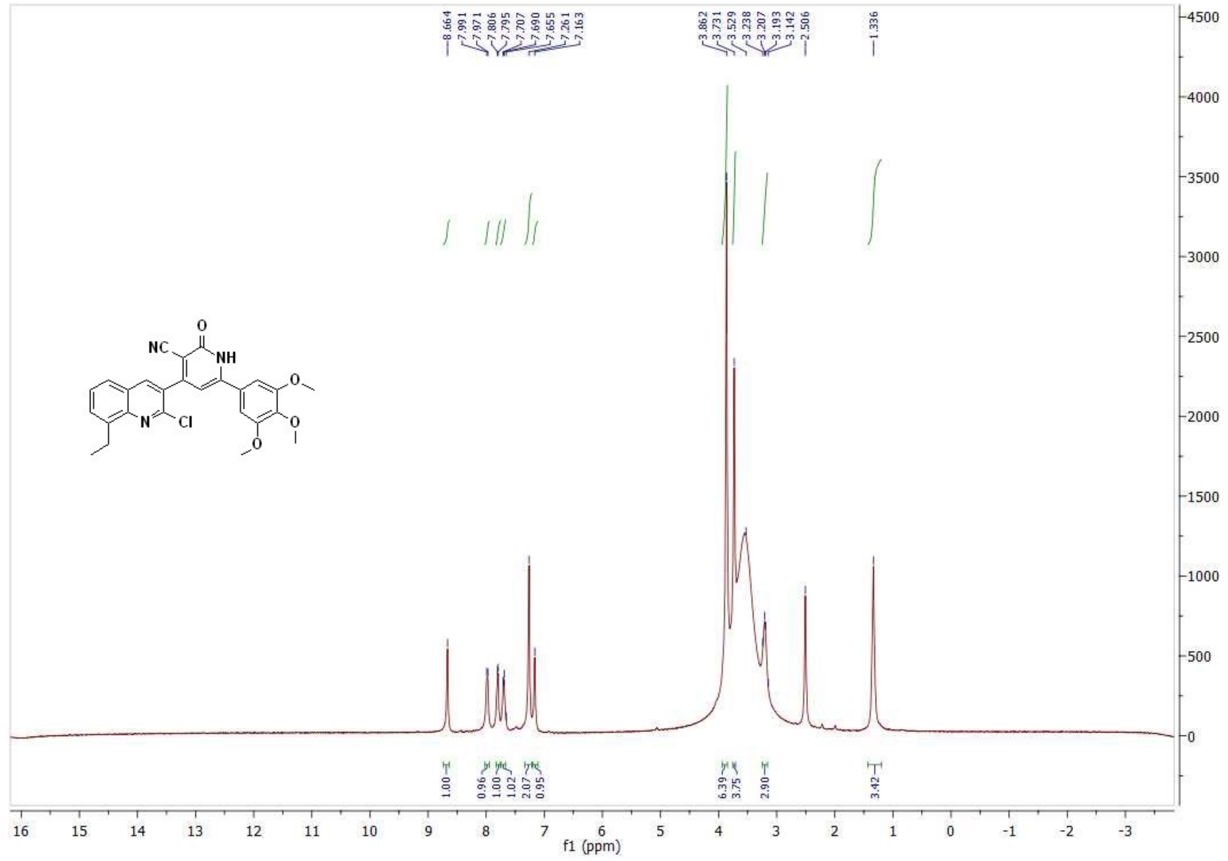

Fig. S24: ^{1}H NMR (400 MHz, DMSO-d_6) spectrum of the compound **4c**.

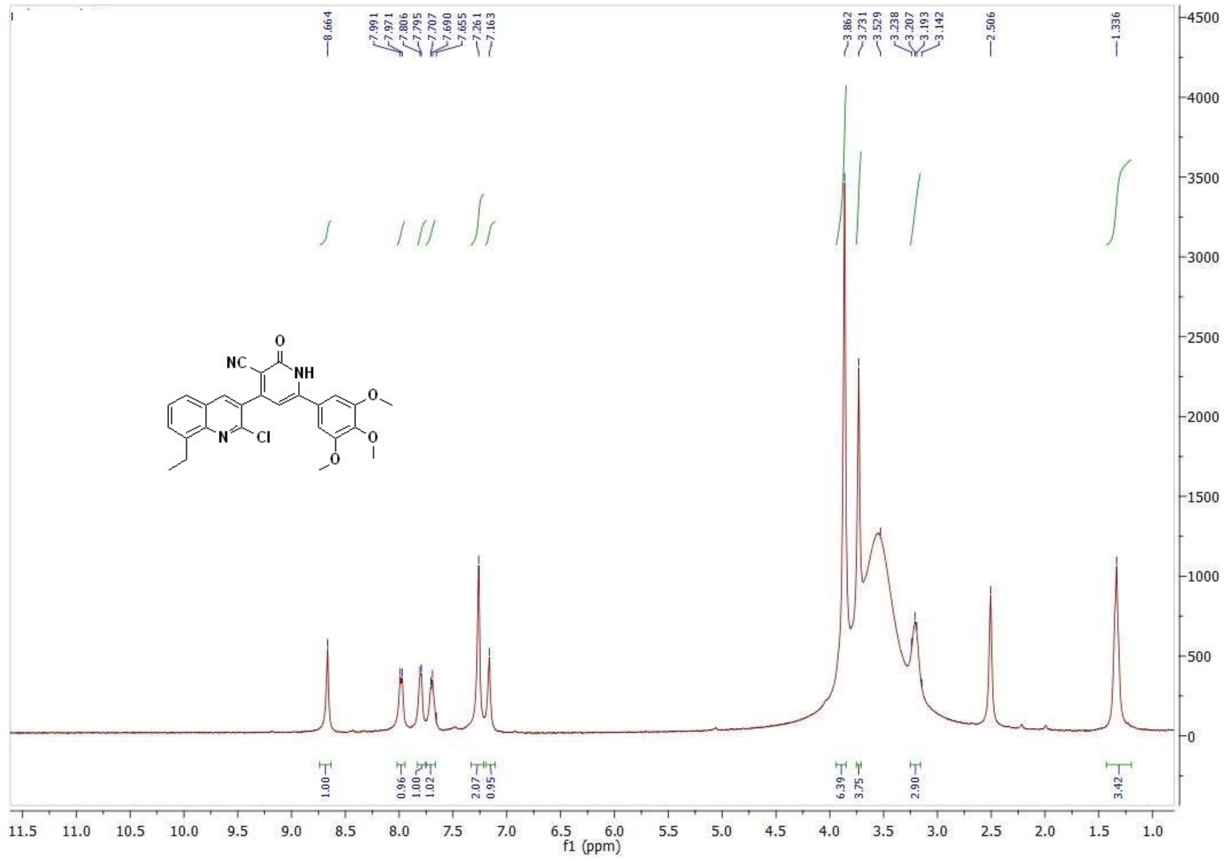

Fig. S25: ^{1}H NMR (400 MHz, DMSO-d_6) magnified spectrum of the compound **4c**.

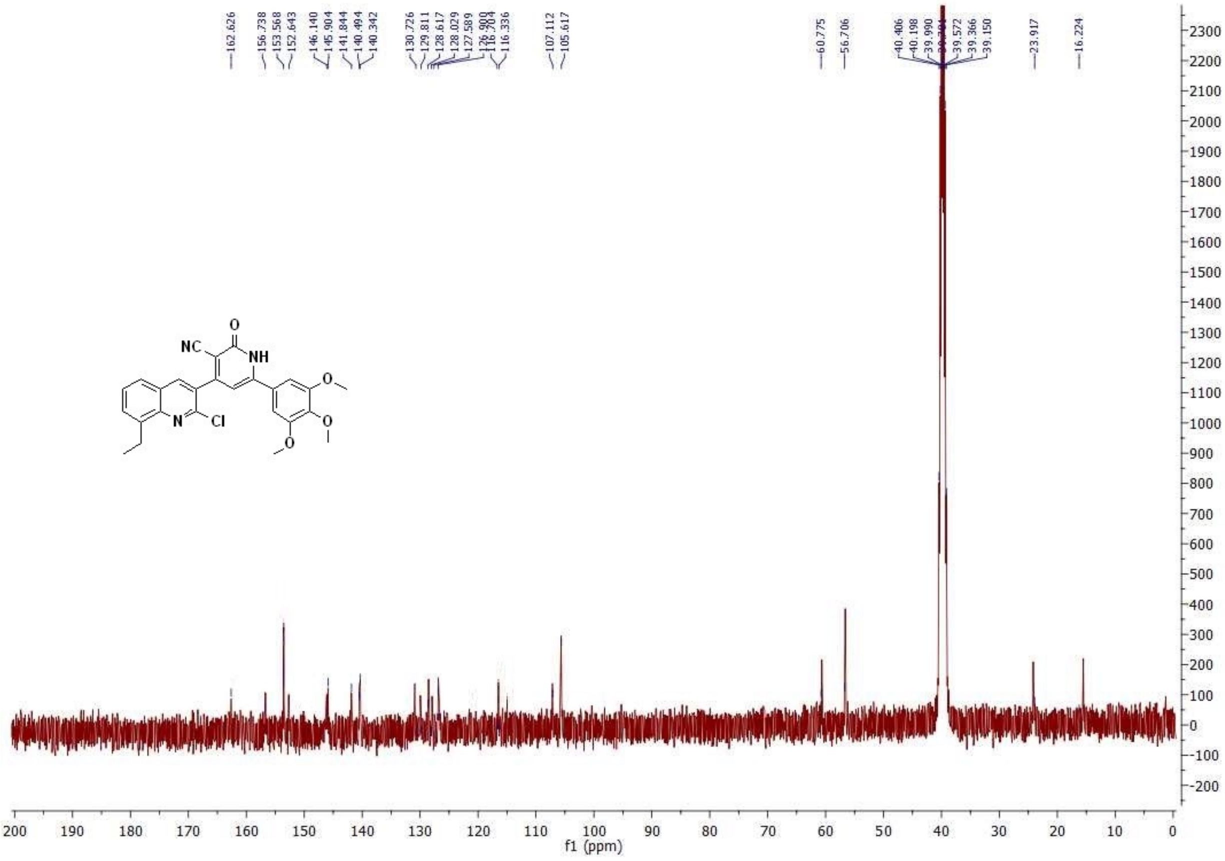

Fig. S26: ^{13}C NMR (100 MHz, DMSO-d_6) spectrum of the compound **4c**.

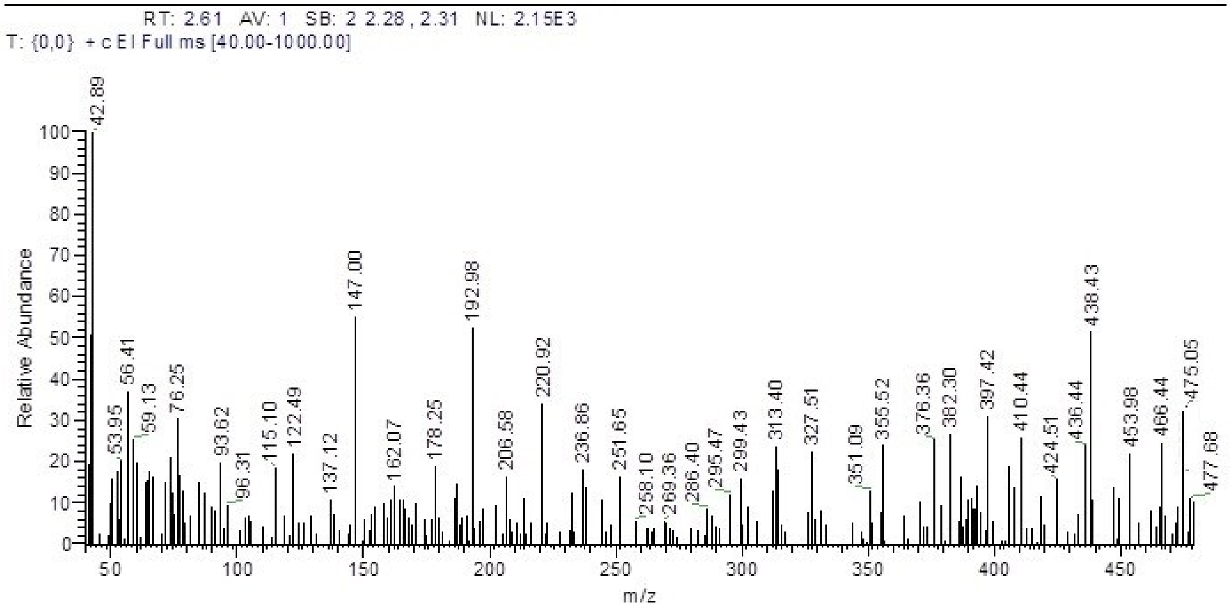

Fig. S27: MS of the compound **4c**.

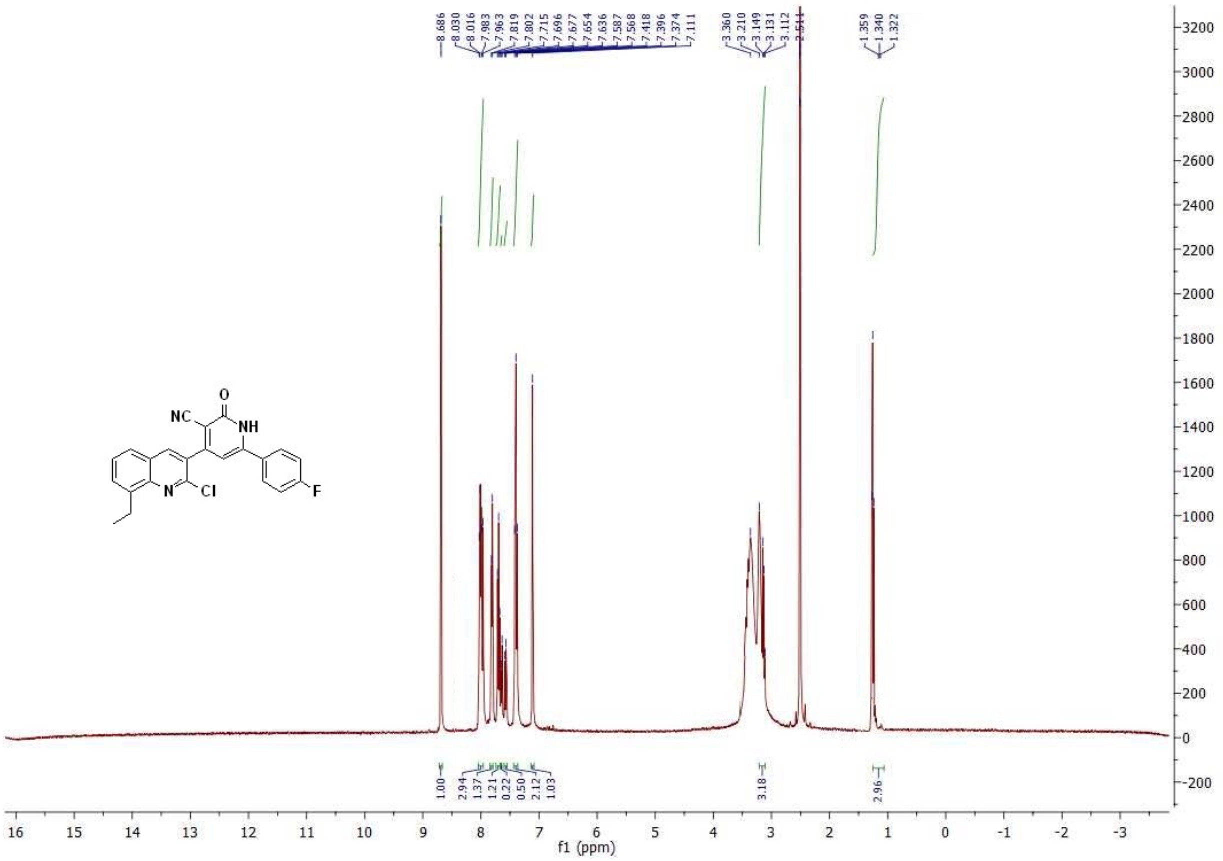

Fig. S28: ^{1}H NMR (400 MHz, DMSO-d_6) spectrum of the compound **4d**.

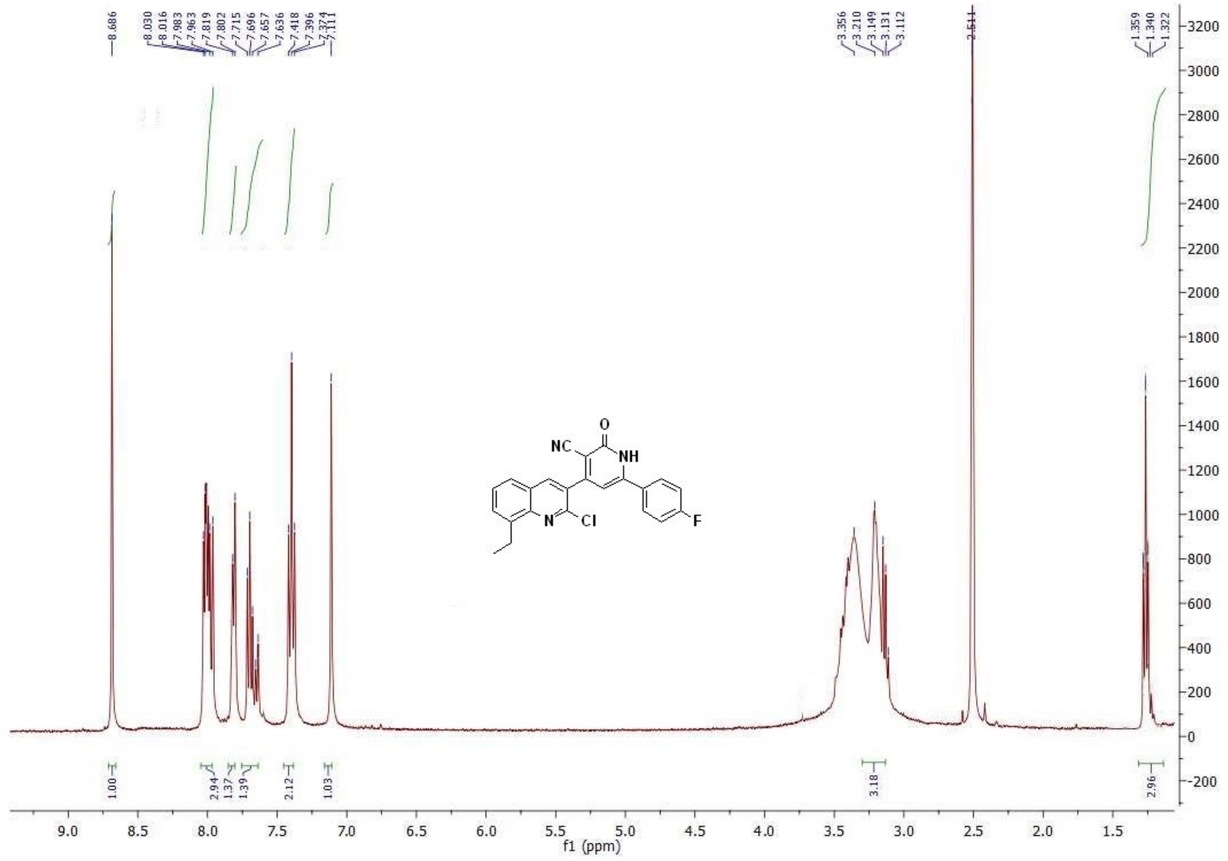

Fig. S29: ^{1}H NMR (400 MHz, DMSO-d_6) magnified spectrum of the compound **4d**.

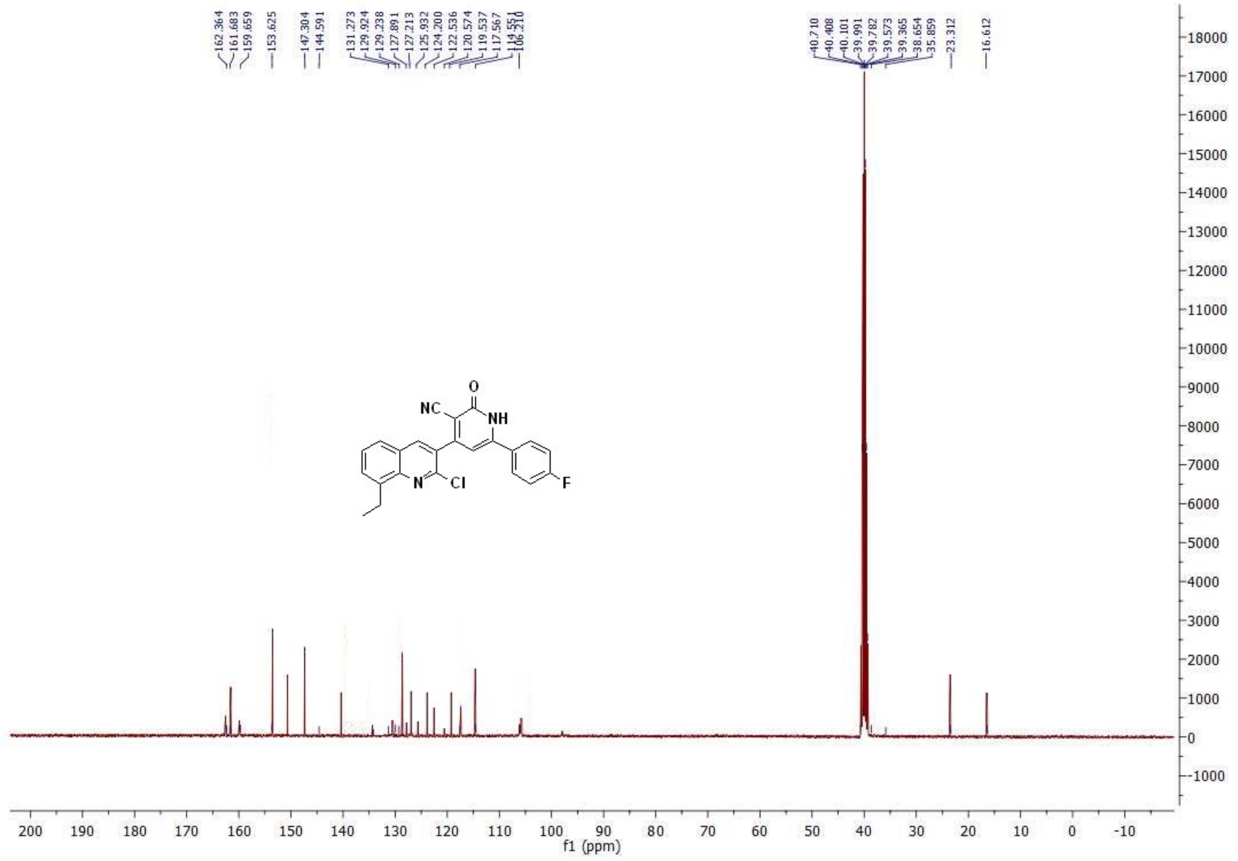

Fig. S30: ^{13}C NMR (100 MHz, DMSO-d_6) spectrum of the compound **4d**.

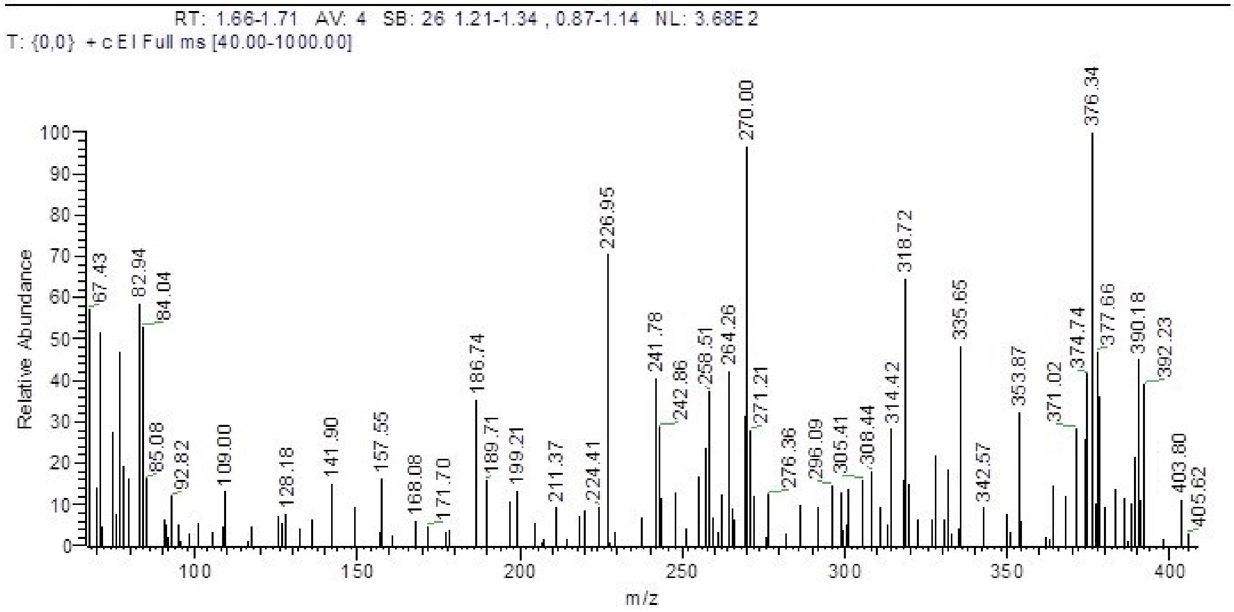

Fig. S31: MS of the compound **4d**.

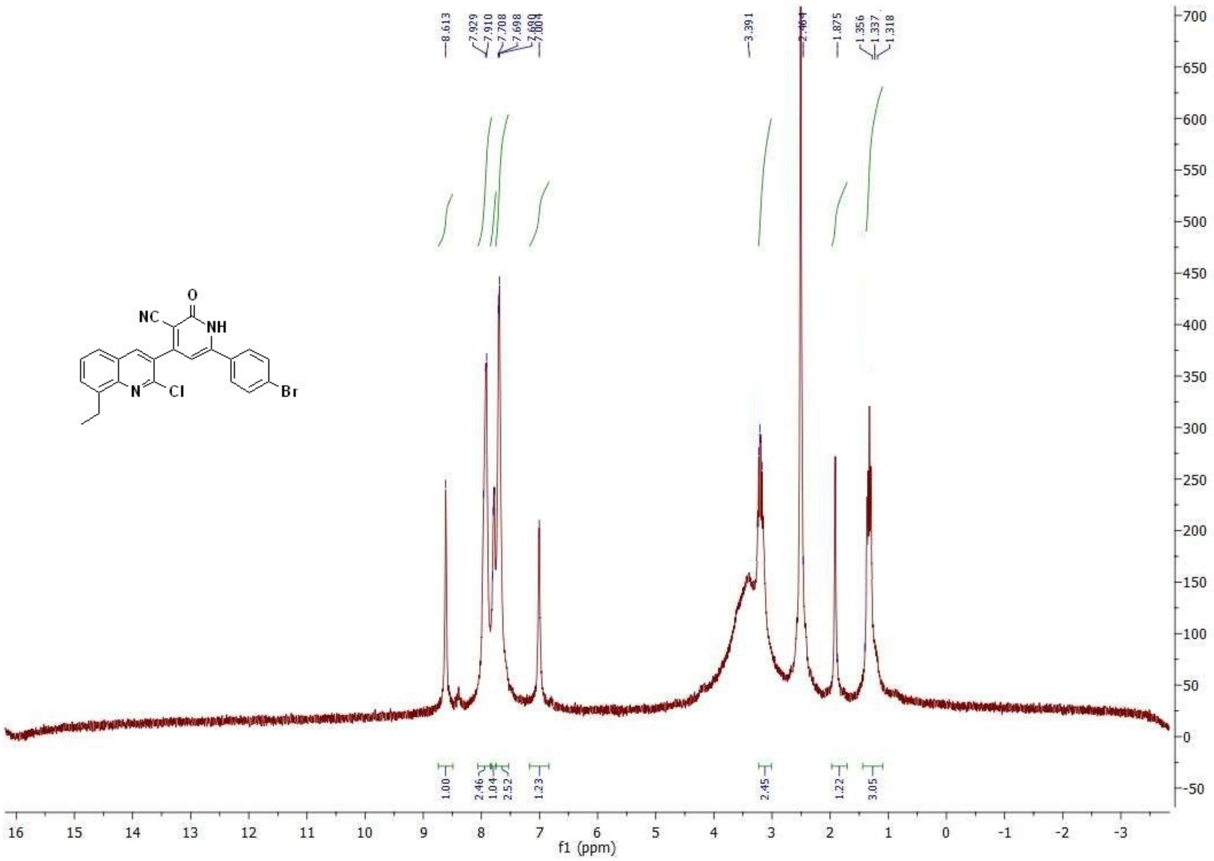

Fig. S32: ^{1}H NMR (400 MHz, DMSO-d_6) spectrum of the compound **4e**.

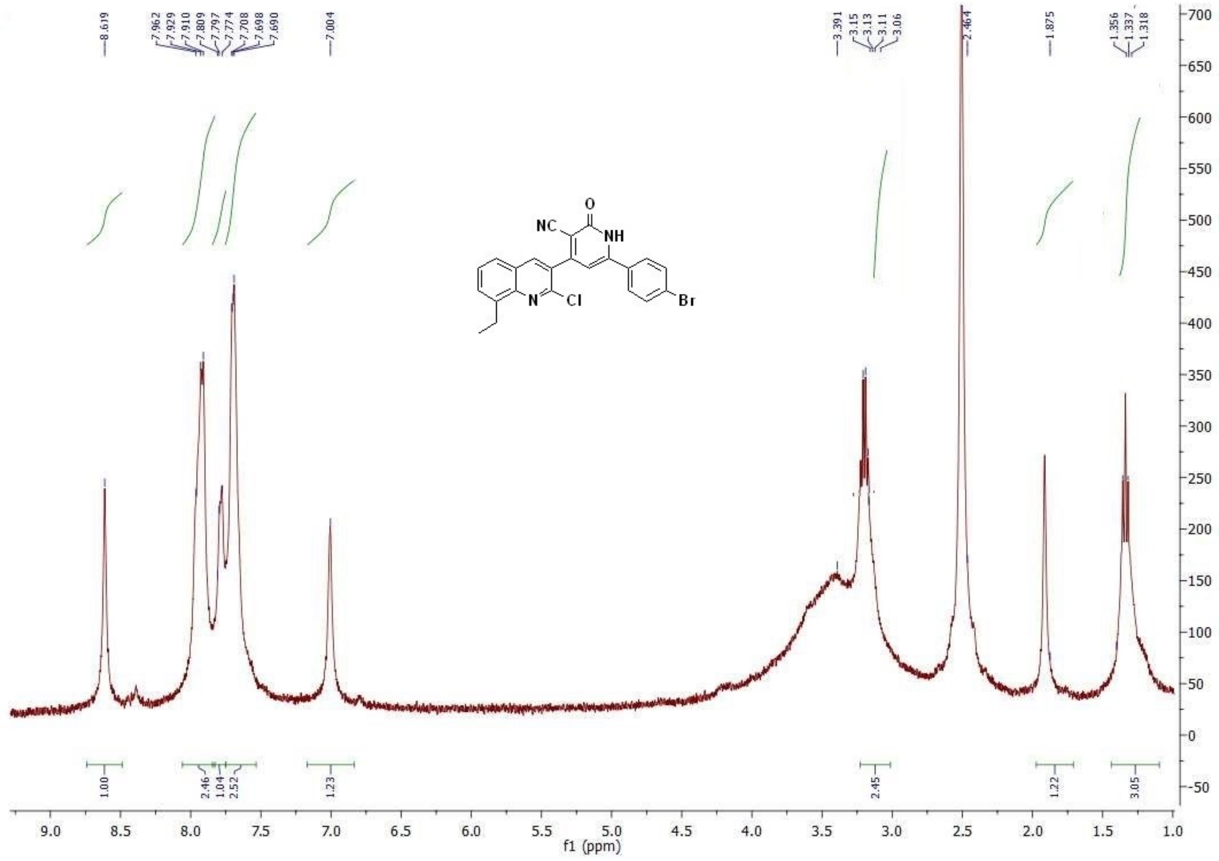

Fig. S33: ^{1}H NMR (400 MHz, DMSO-d_6) magnified spectrum of the compound **4e**.

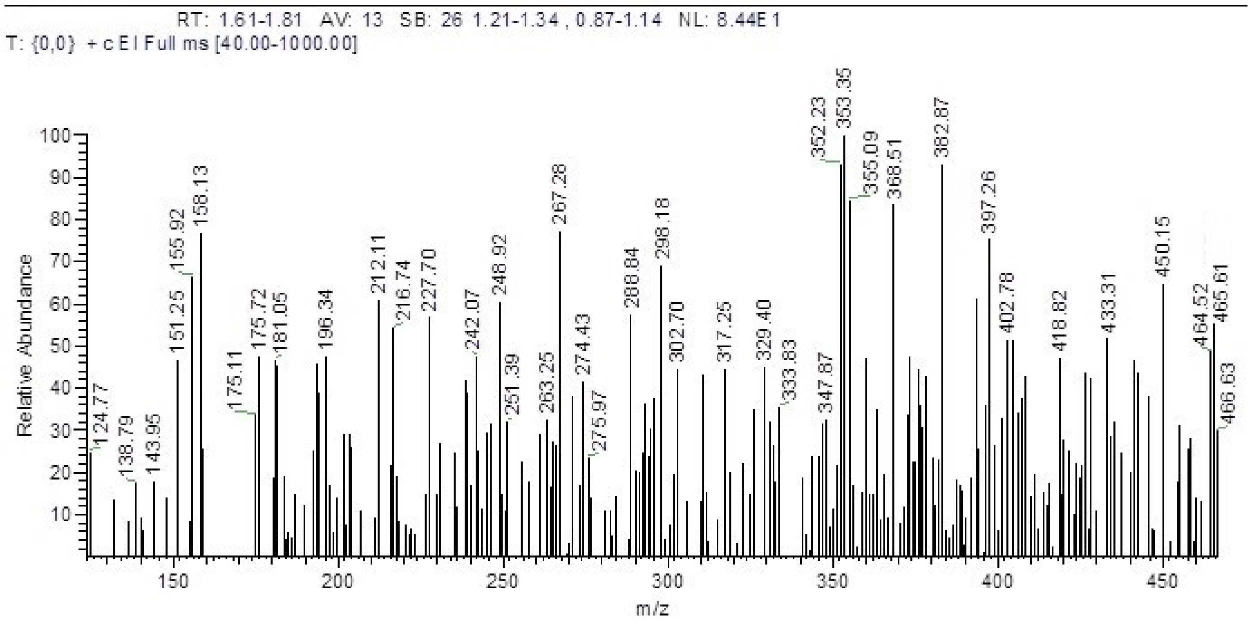

Fig. S34: MS of the compound **4e**.

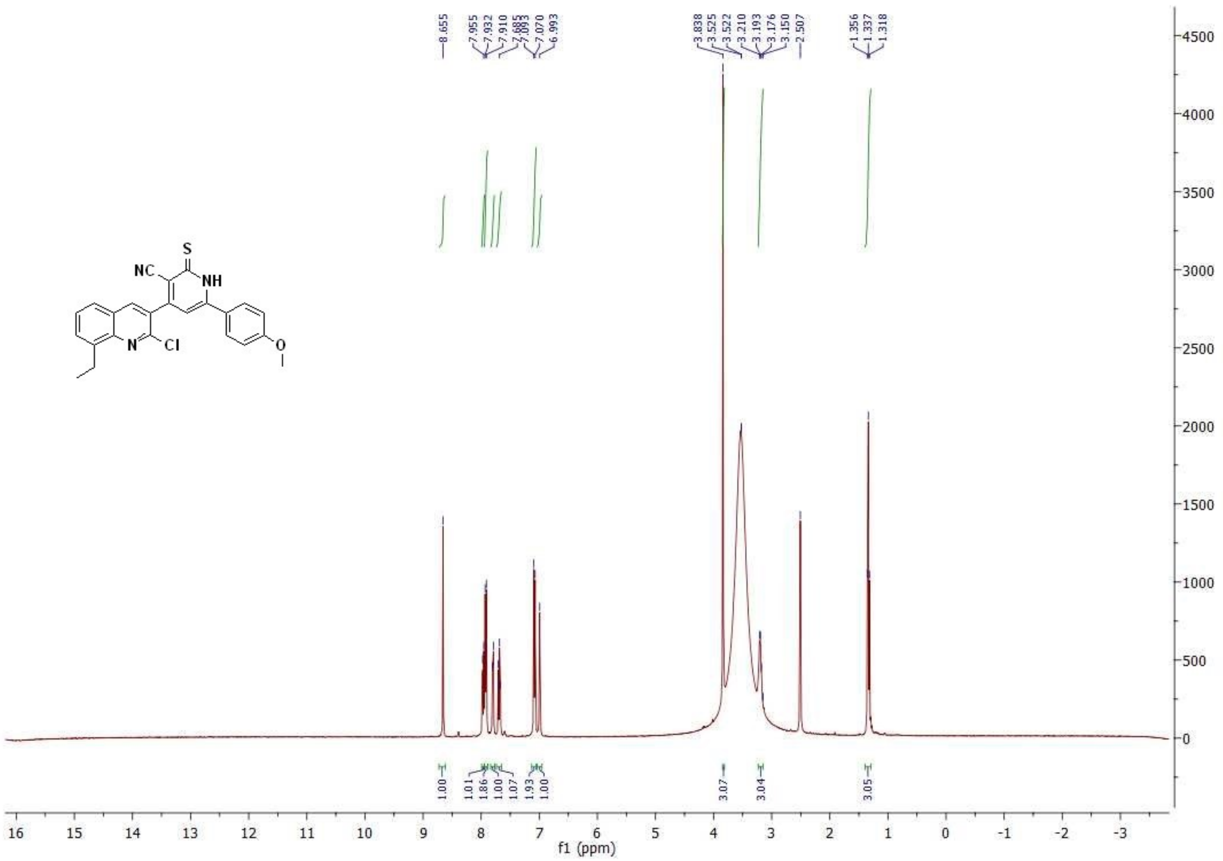

Fig. S35: ^{1}H NMR (400 MHz, DMSO-d_6) spectrum of the compound **5a**.

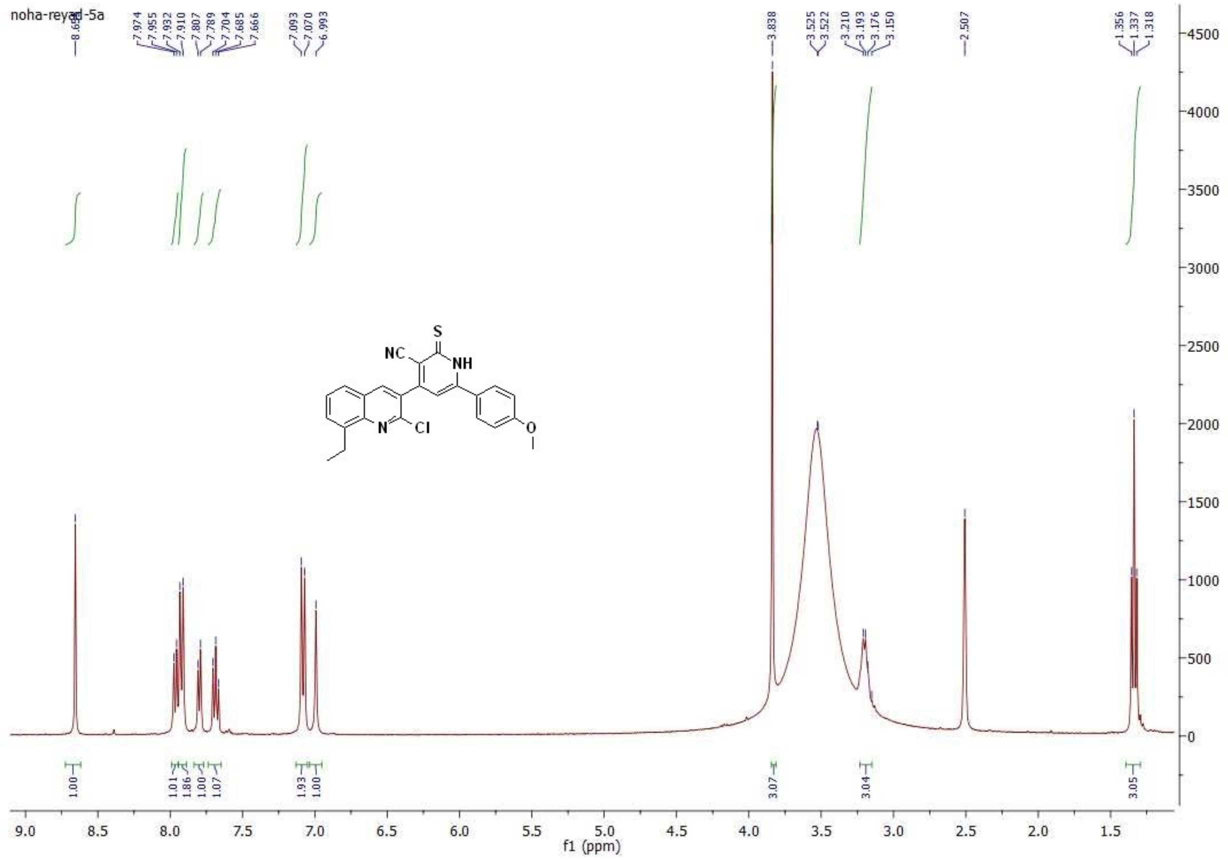

Fig. S36: ^{1}H NMR (400 MHz, DMSO-d_6) magnified spectrum of the compound **5a**.

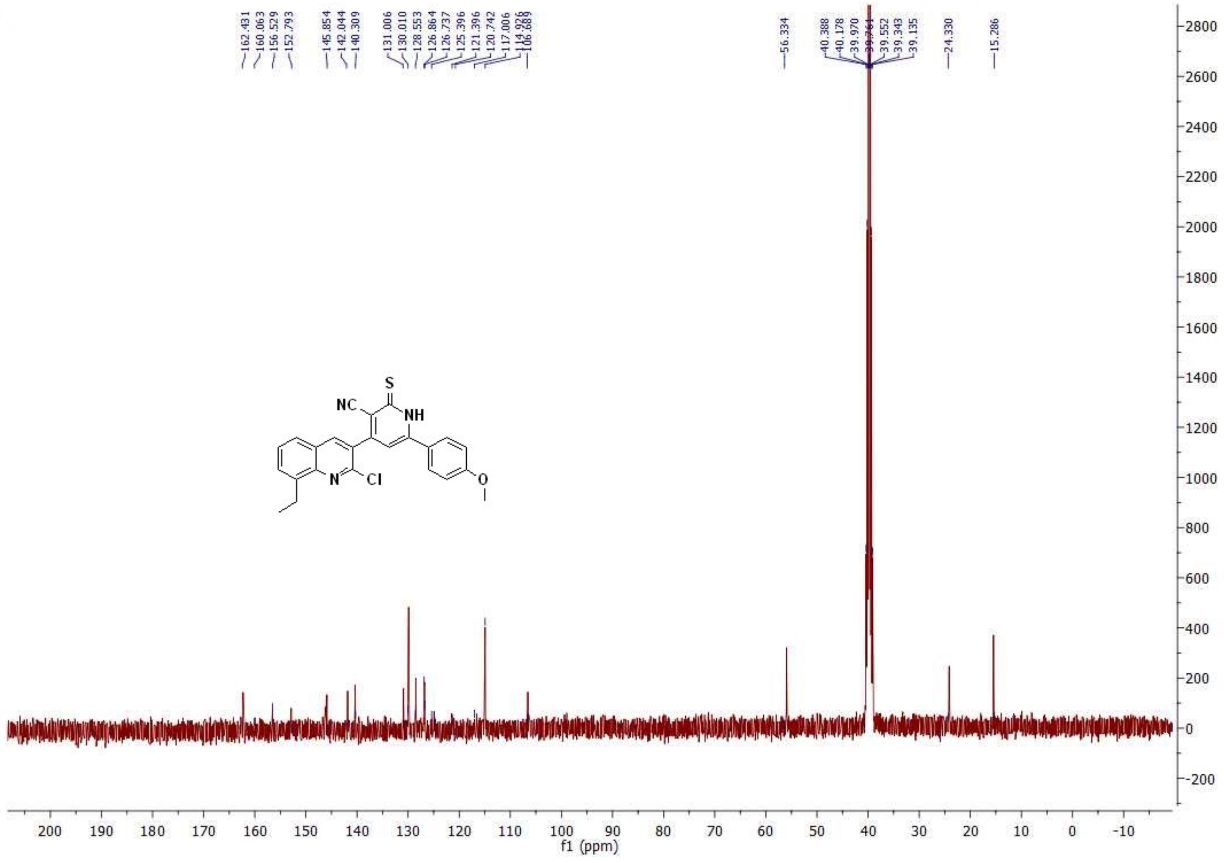

Fig. S37: ^{13}C NMR (100 MHz, DMSO-d_6) spectrum of the compound **5a**.

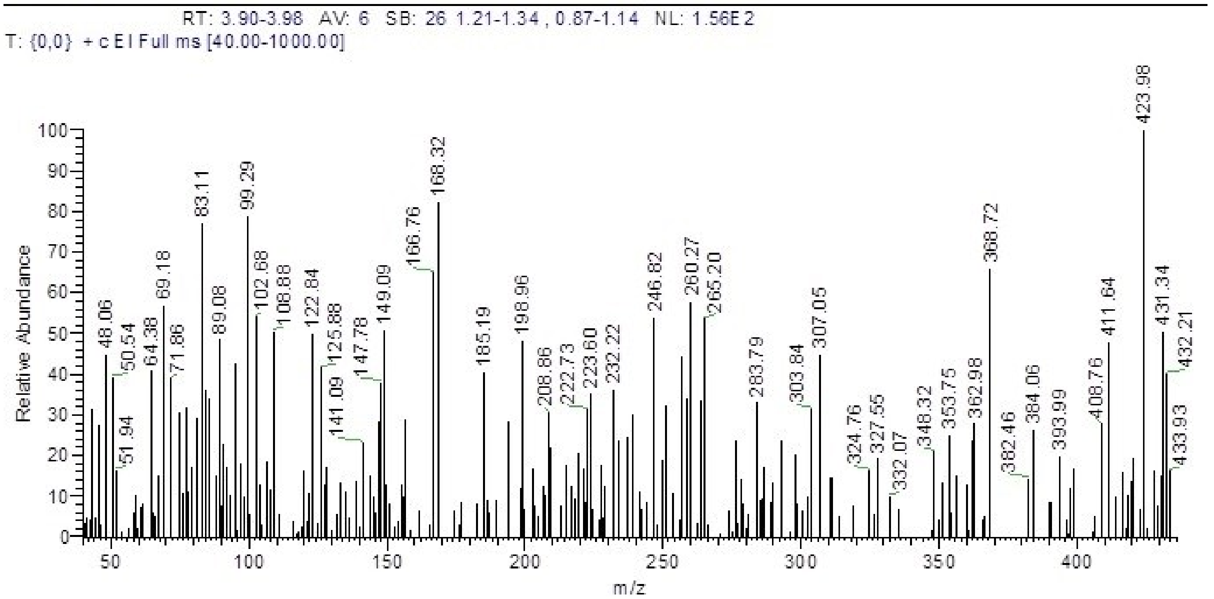

Fig. S38: MS of the compound **5a**.

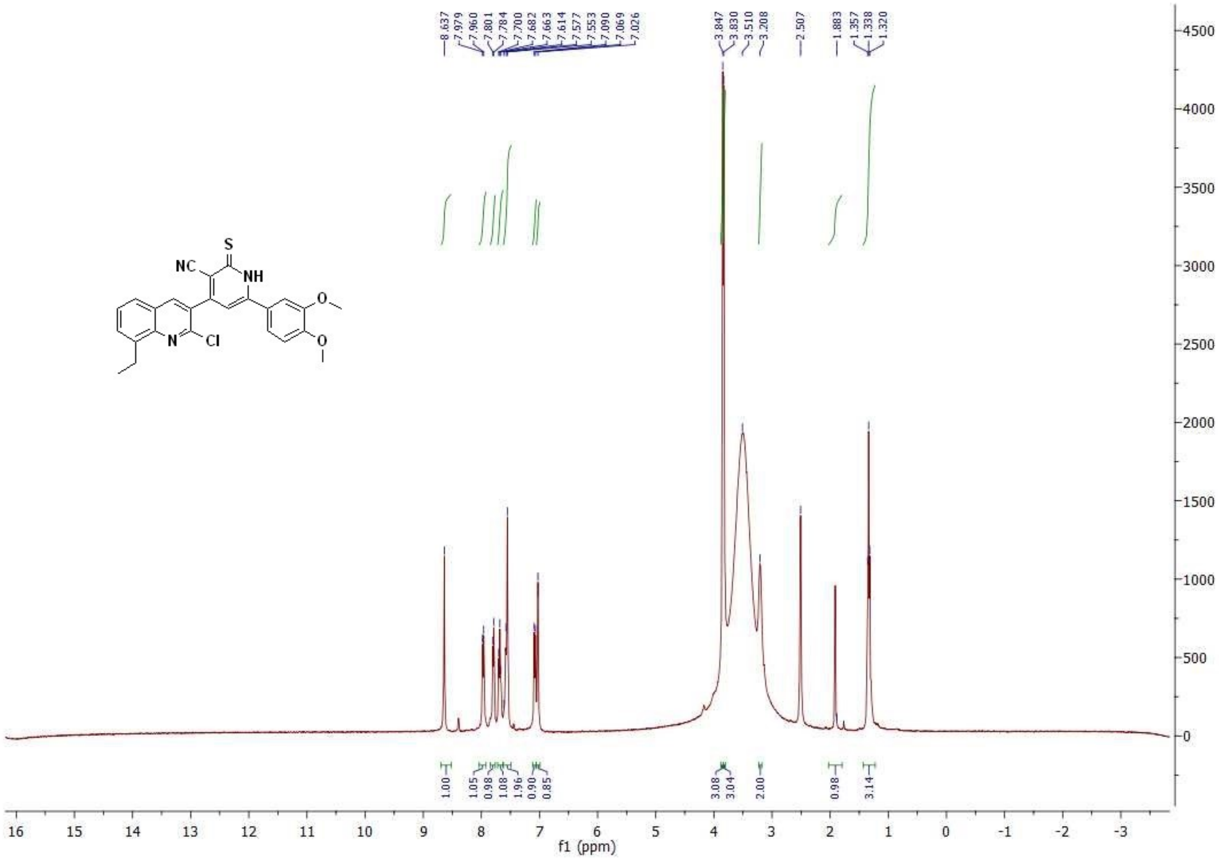

Fig. S39: ^{1}H NMR (400 MHz, DMSO-d_6) spectrum of the compound **5b**.

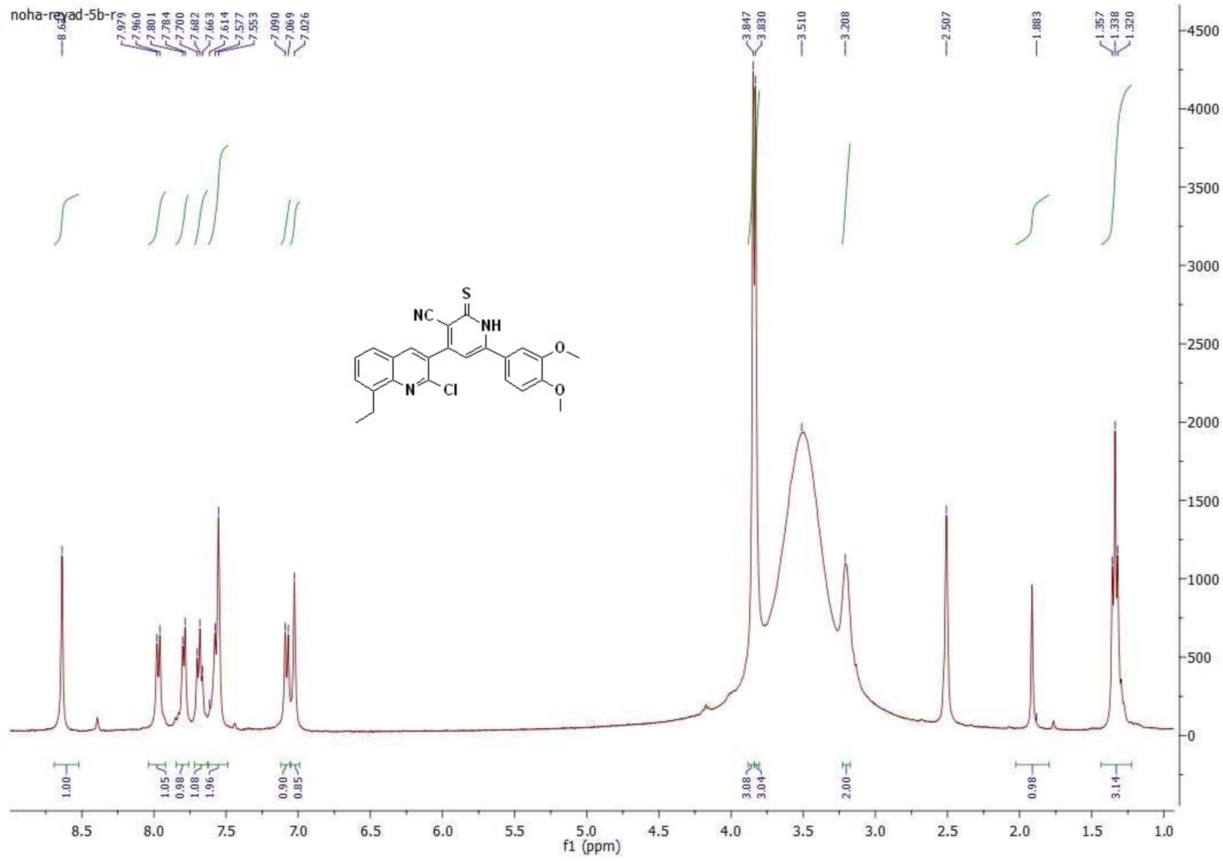

Fig. S40: ^{1}H NMR (400 MHz, DMSO-d_6) magnified spectrum of the compound **5b**.

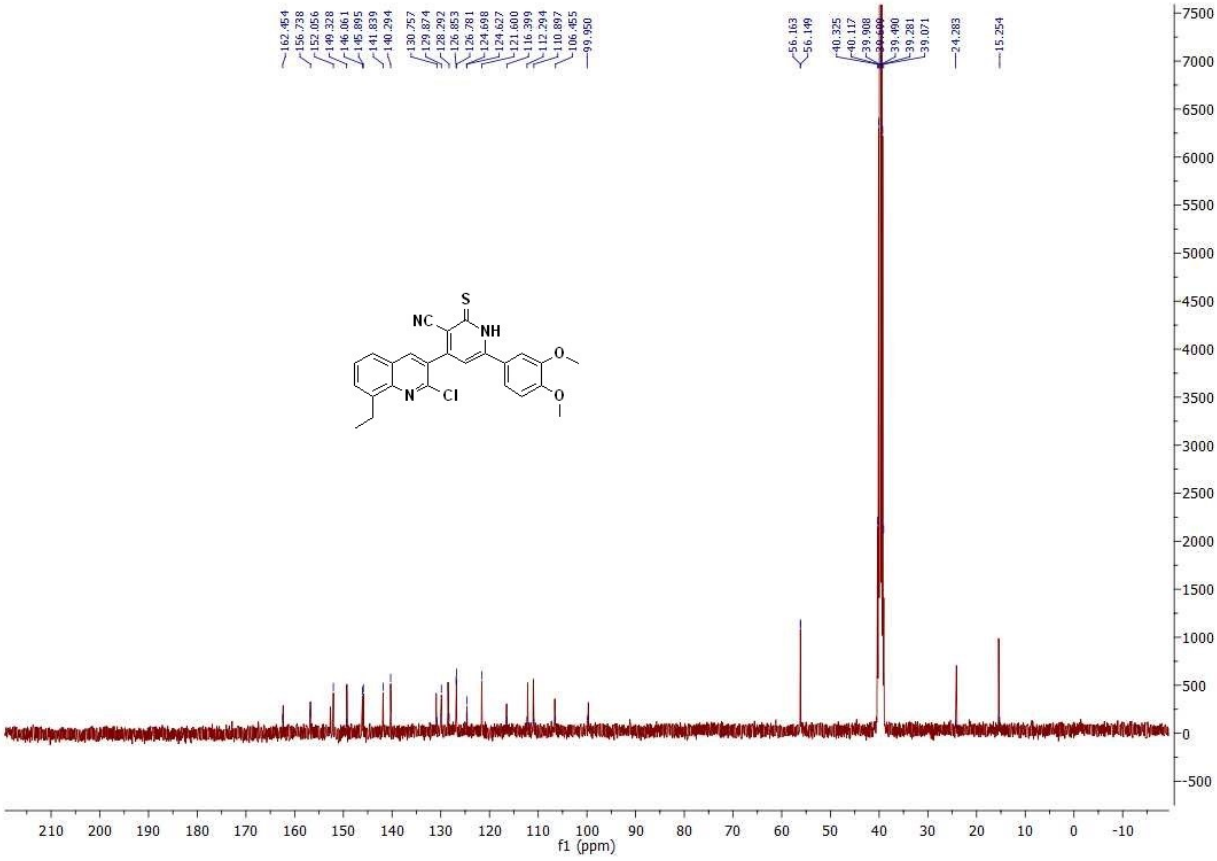

Fig. S41: ^{13}C NMR (100 MHz, DMSO-d_6) spectrum of the compound **5b**.

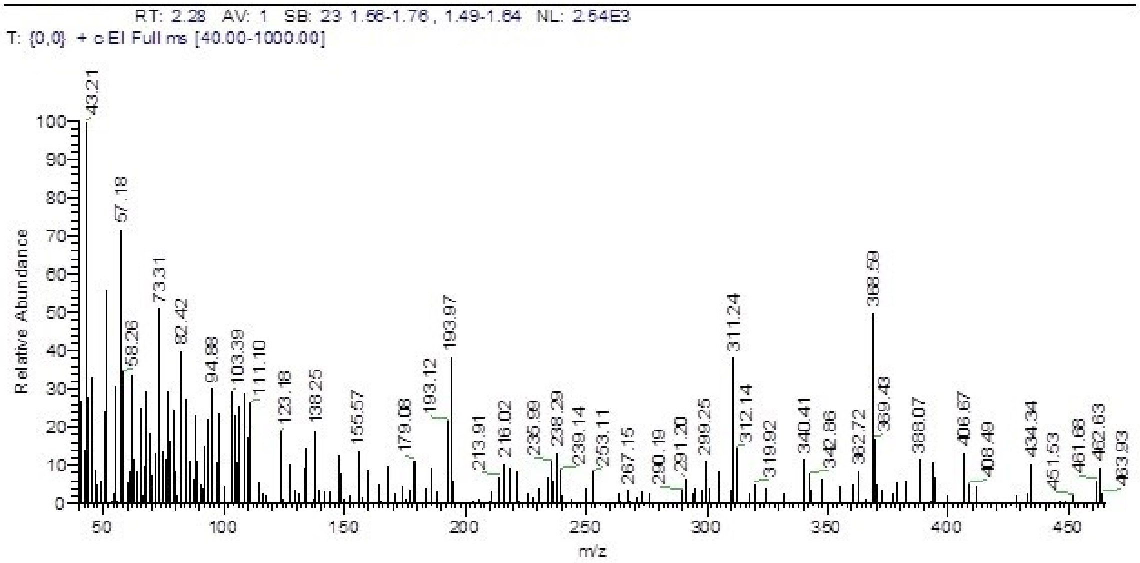

Fig. S42: MS of the compound **5b**.

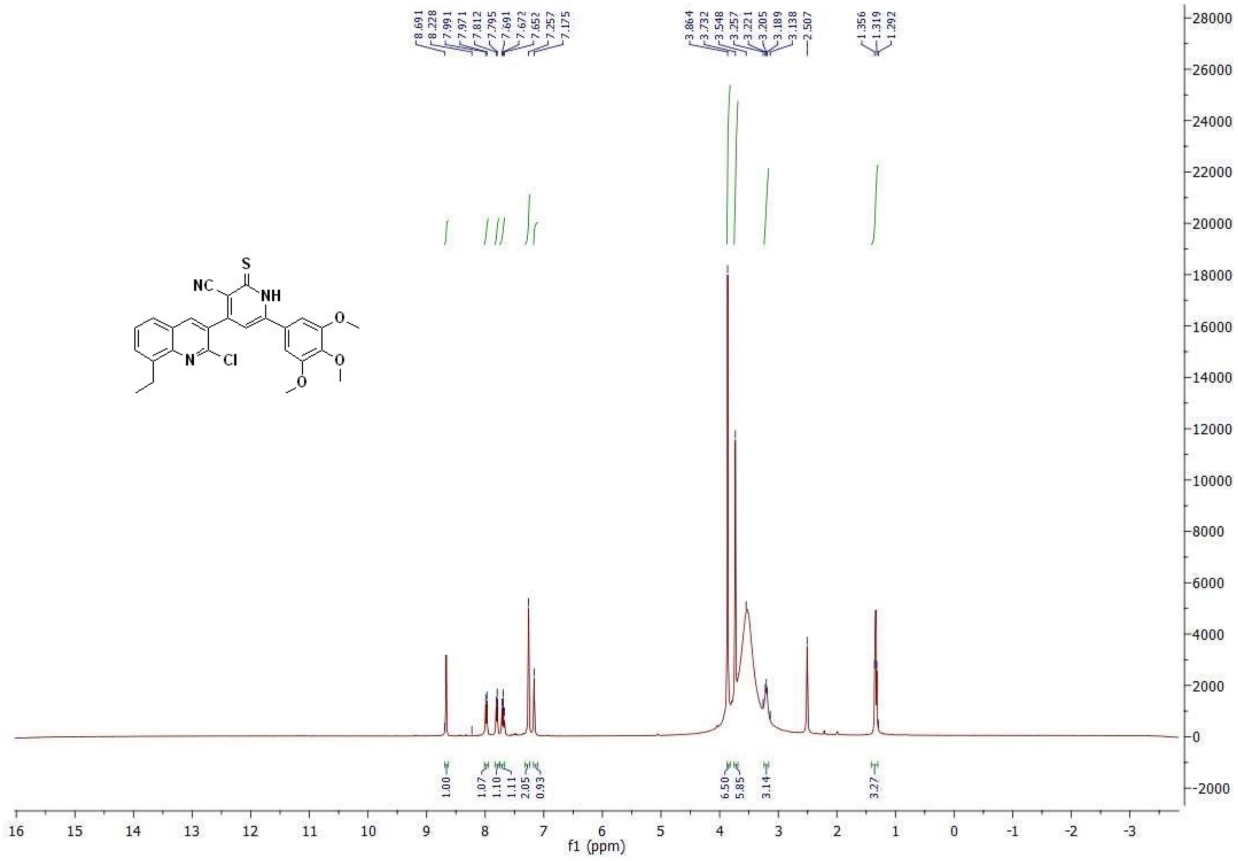

Fig. S43: ^{1}H NMR (400 MHz, DMSO-d_6) spectrum of the compound **5c**.

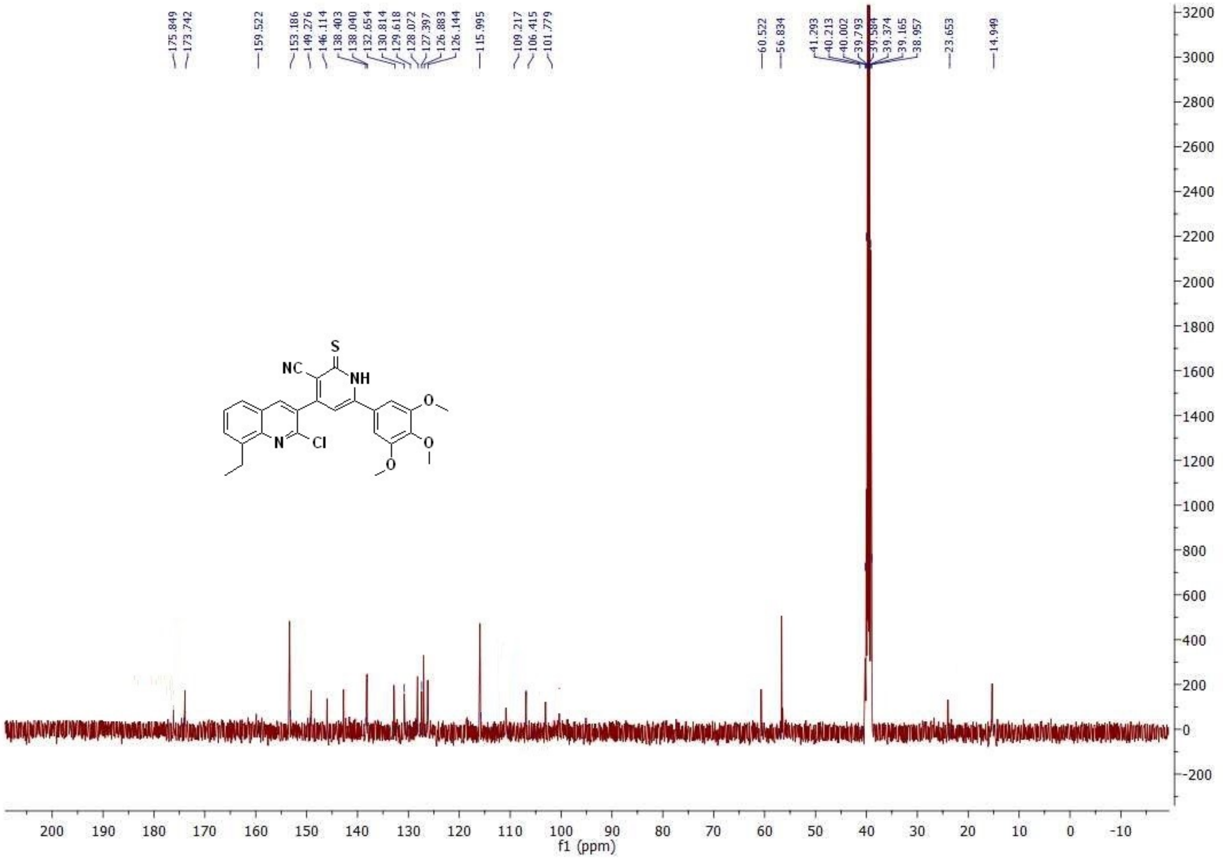

Fig. S44: ^{13}C NMR (100 MHz, DMSO-d_6) spectrum of the compound **5c**.

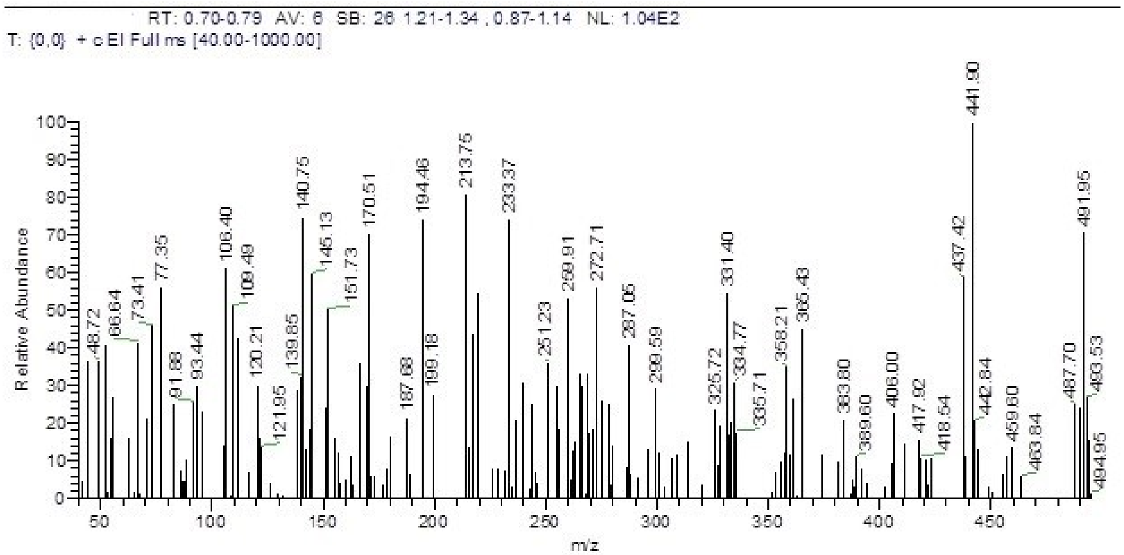

Fig. S45: MS of the compound **5c**.

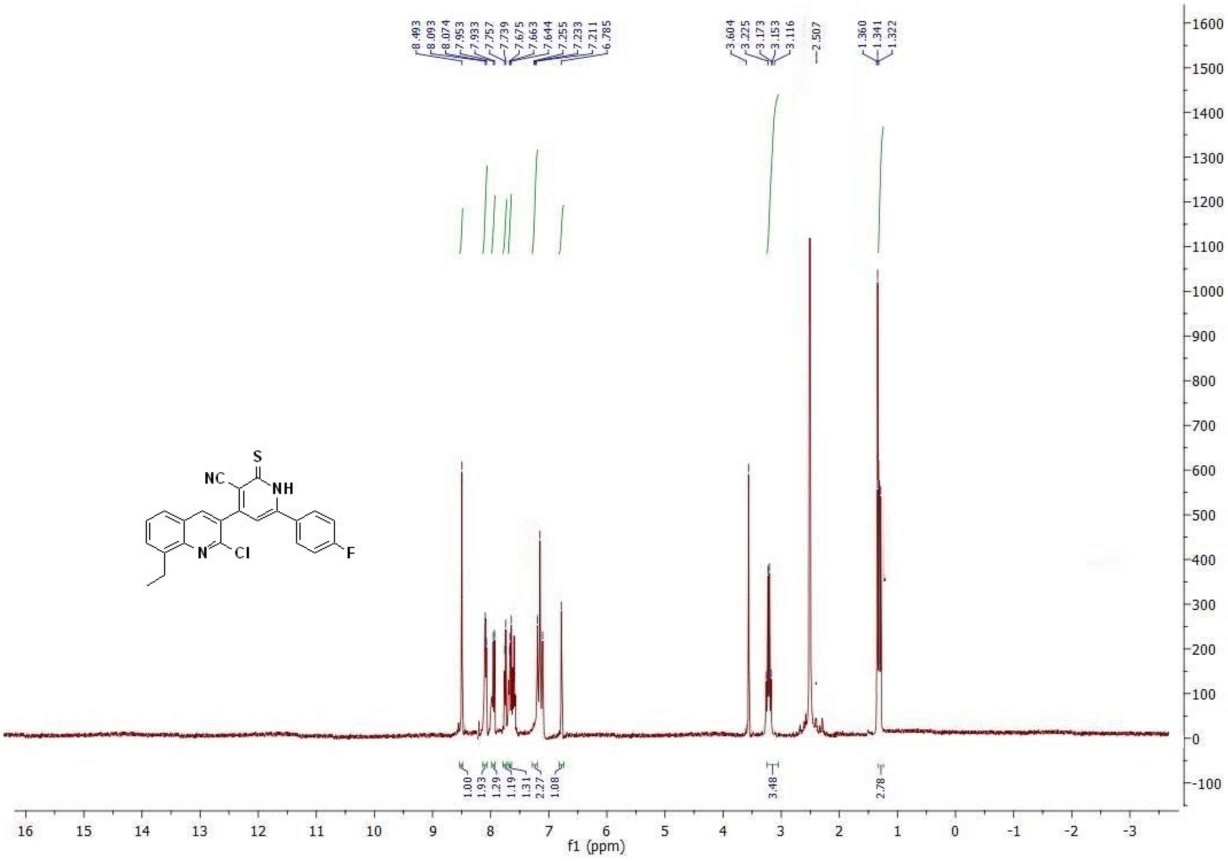

Fig. S46: ^{1}H NMR (400 MHz, DMSO-d_6) spectrum of the compound **5d**.

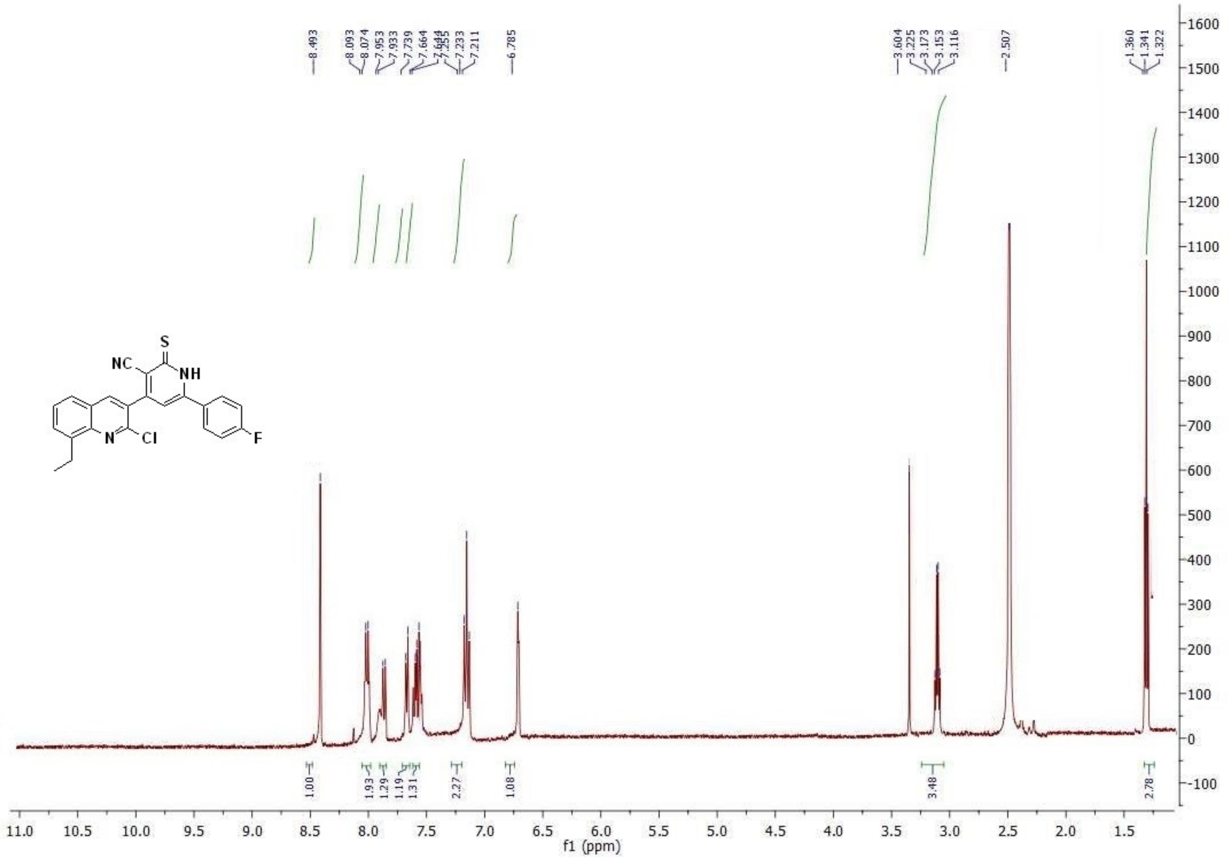

Fig. S47: ^{1}H NMR (400 MHz, DMSO-d_6) magnified spectrum of the compound **5d**.

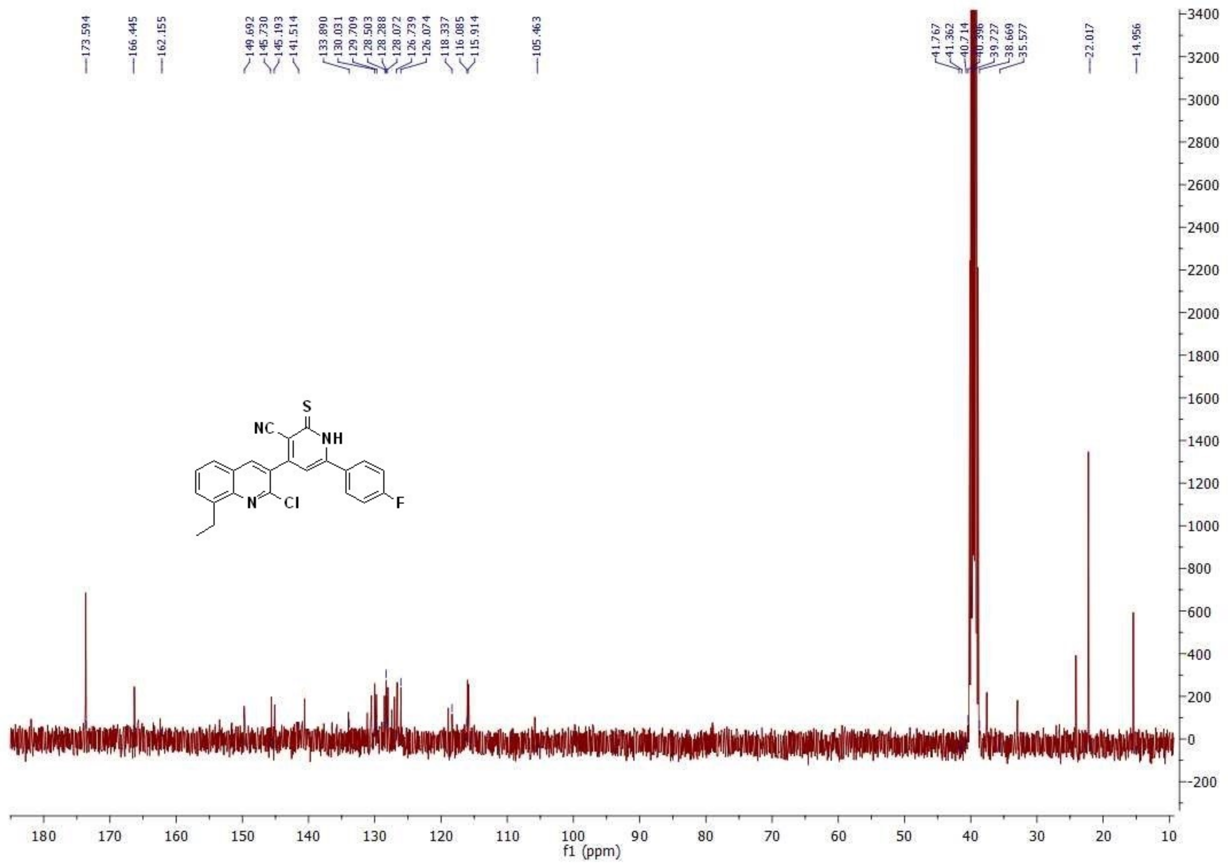

Fig. S48: ^{13}C NMR (100 MHz, DMSO-d_6) spectrum of the compound **5d**.

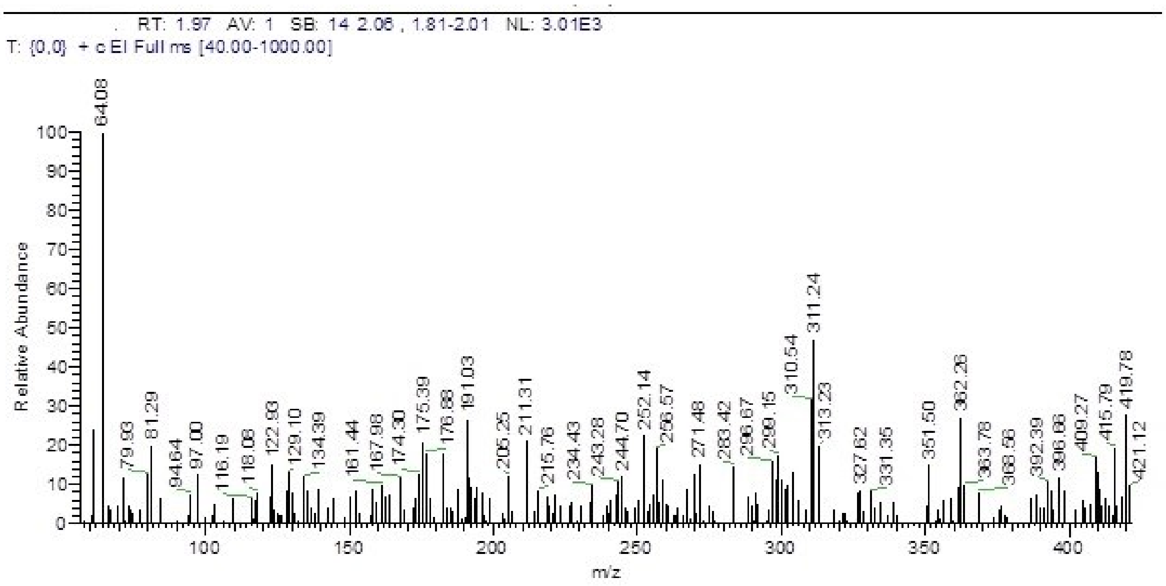

Fig. S49: MS of the compound **5d**.

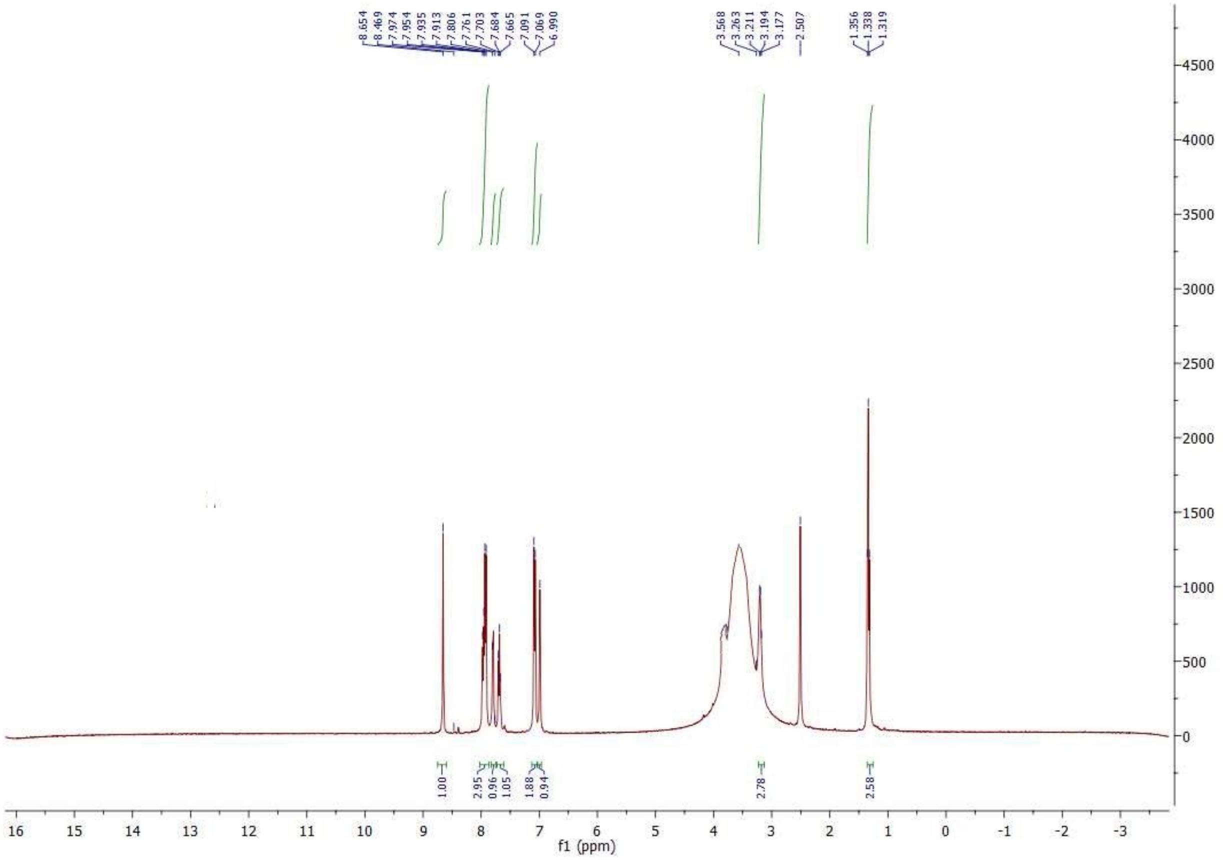

Fig. S50: ^{1}H NMR (400 MHz, DMSO-d_6) spectrum of the compound **5e.**

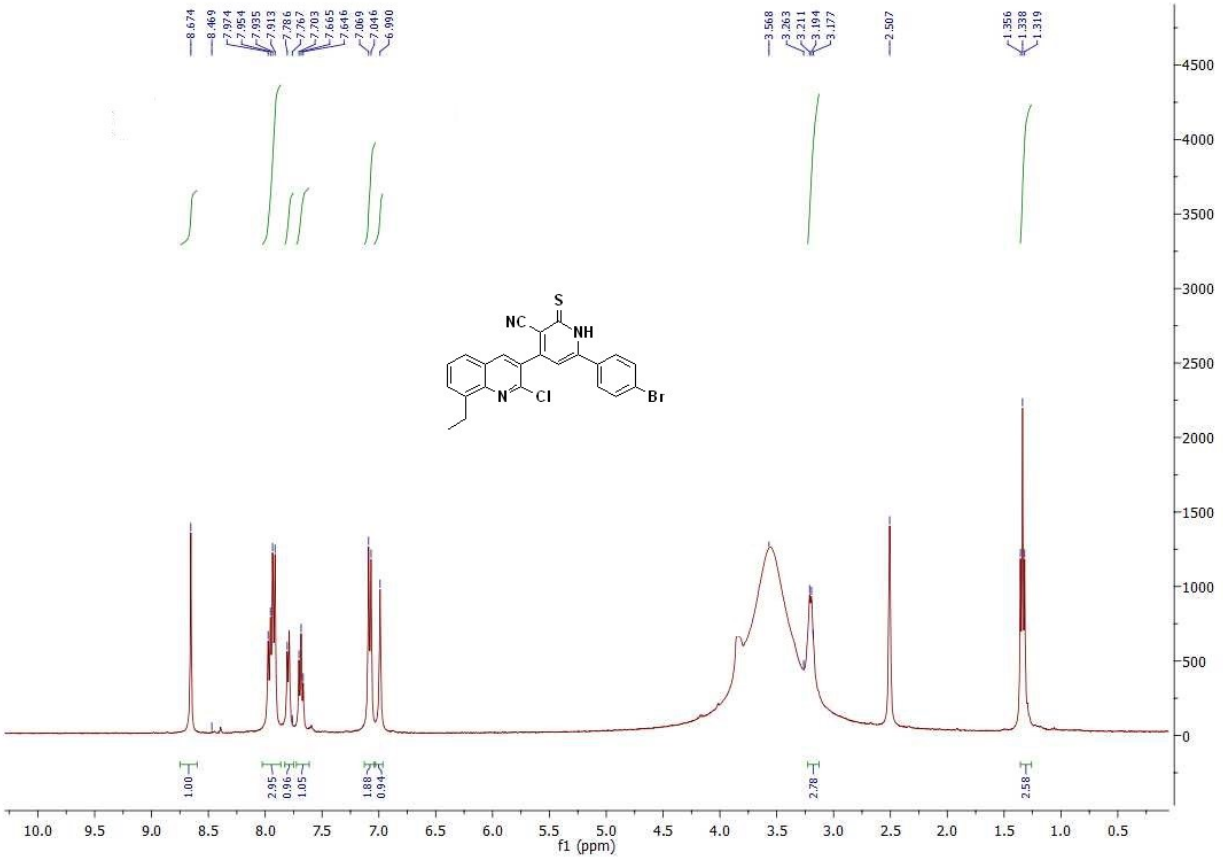

Fig. S51: ^{1}H NMR (400 MHz, DMSO-d_6) magnified spectrum of the compound **5e.**

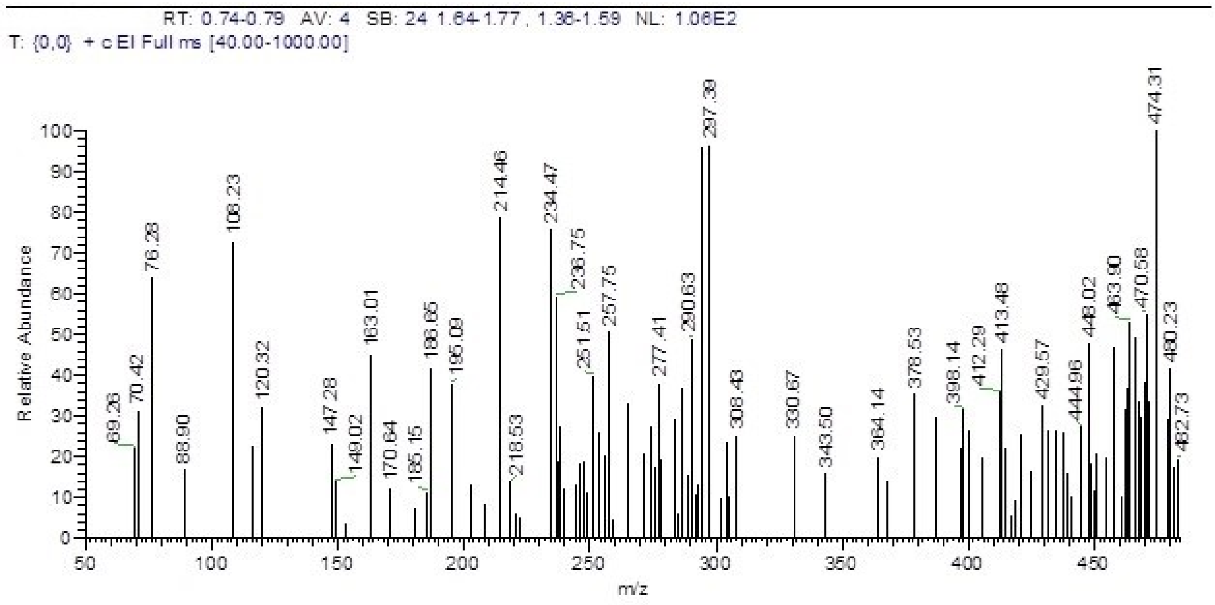

Fig. S52: MS of the compound **5e**.

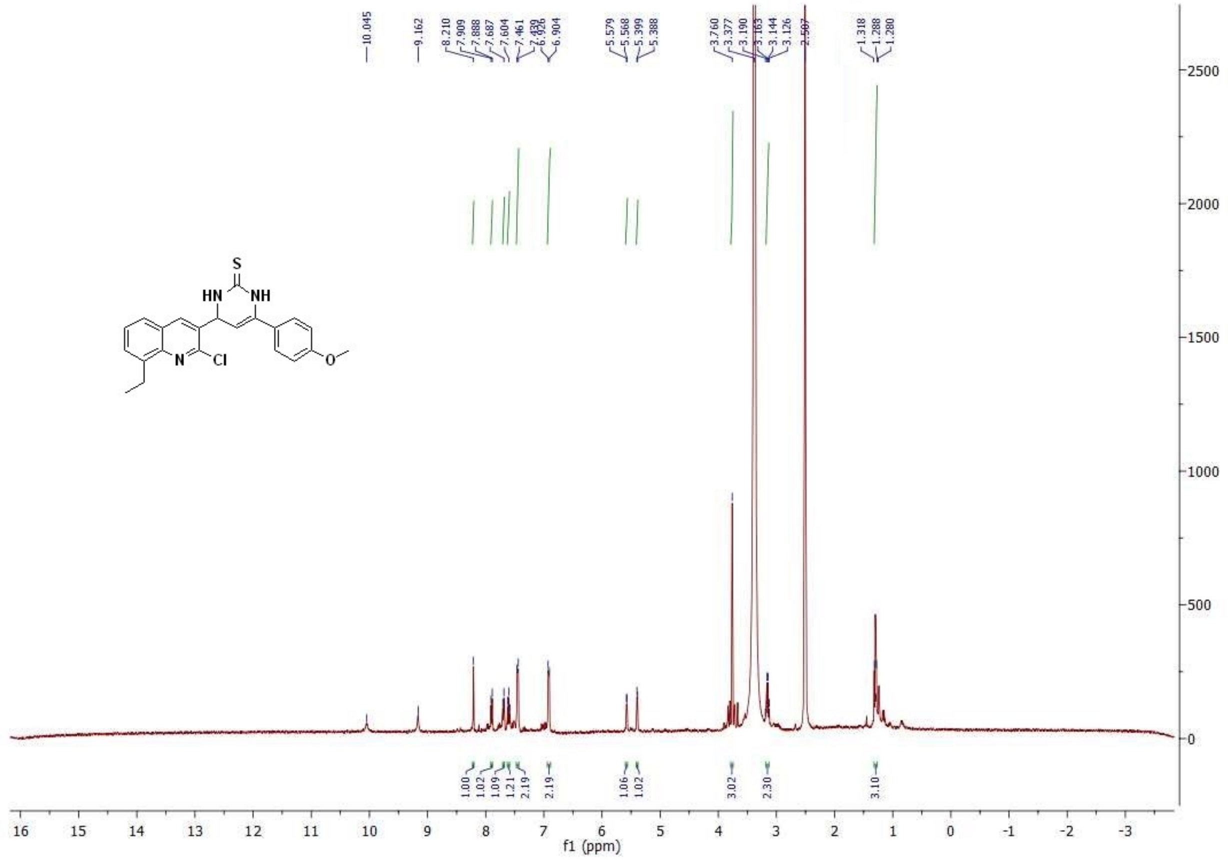

Fig. S53: ^{1}H NMR (400 MHz, DMSO-d_6) spectrum of the compound **6a.**

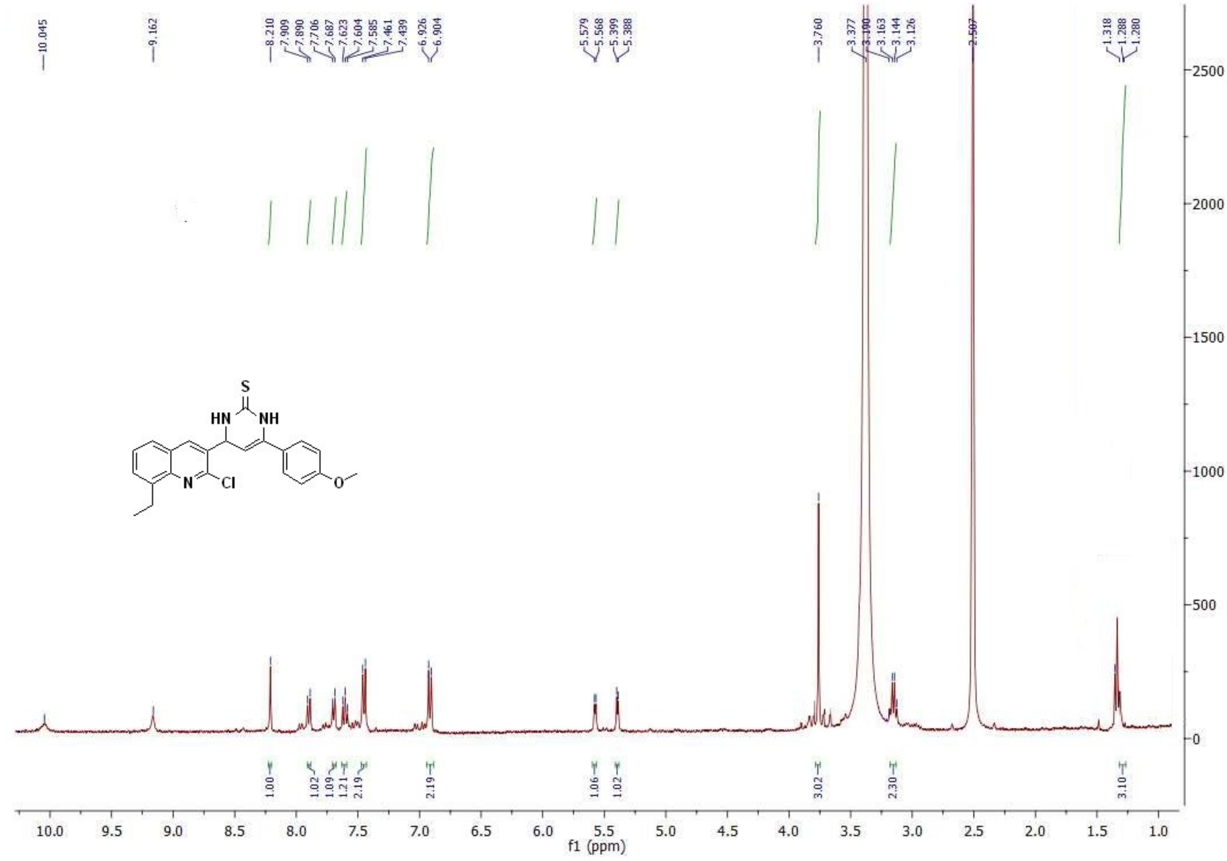

Fig. S54: ^{1}H NMR (400 MHz, DMSO-d_6) magnified spectrum of the compound **6a.**

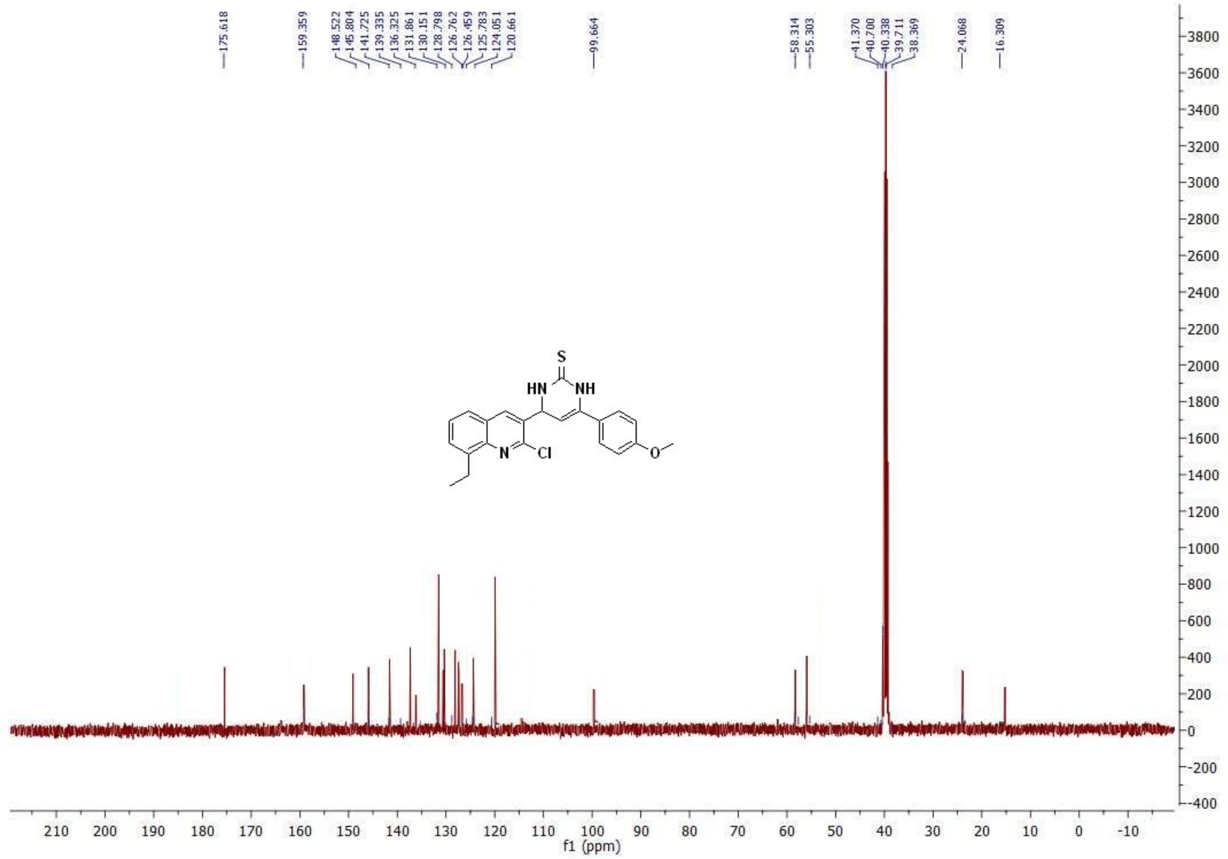

Fig. S55: ^{13}C NMR (100 MHz, DMSO-d_6) spectrum of the compound **6a**.

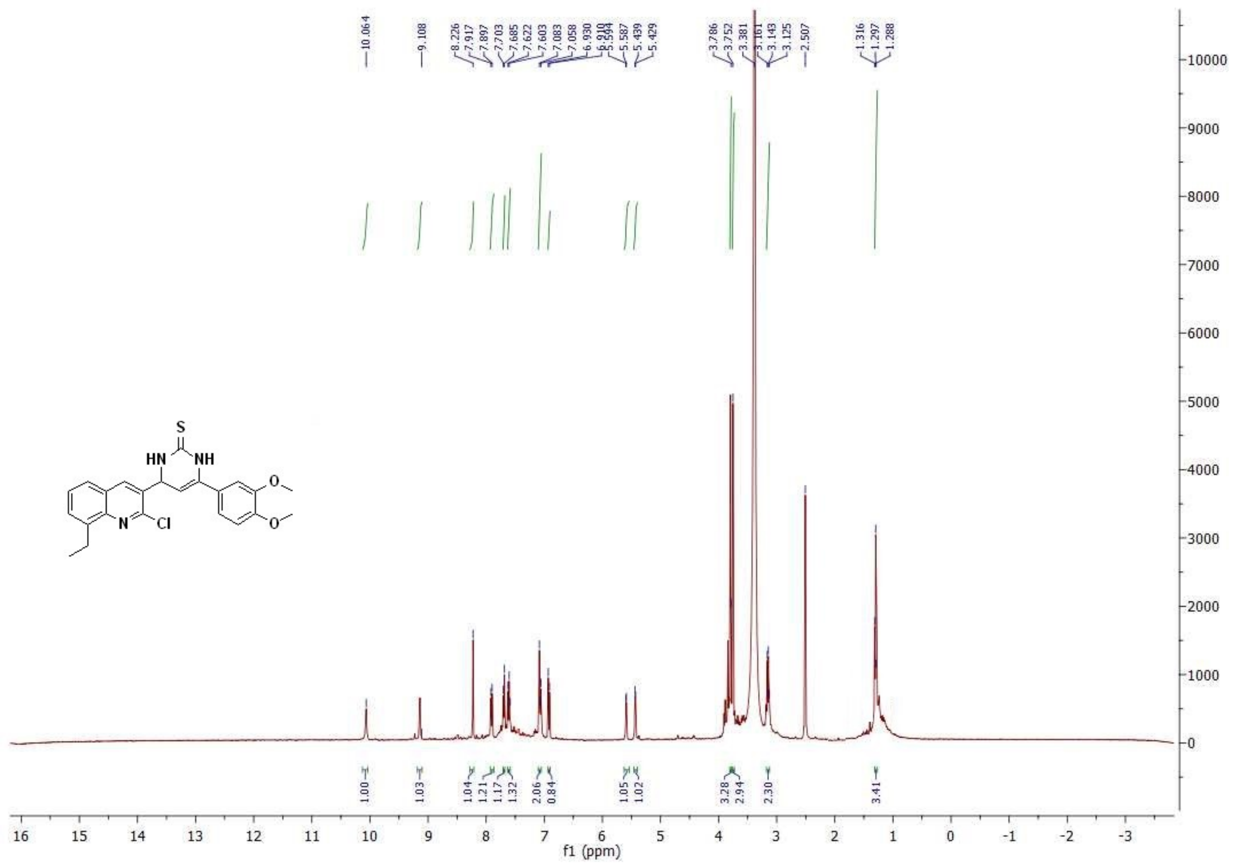

Fig. S56: ^{1}H NMR (400 MHz, DMSO-d_6) spectrum of the compound **6b.**

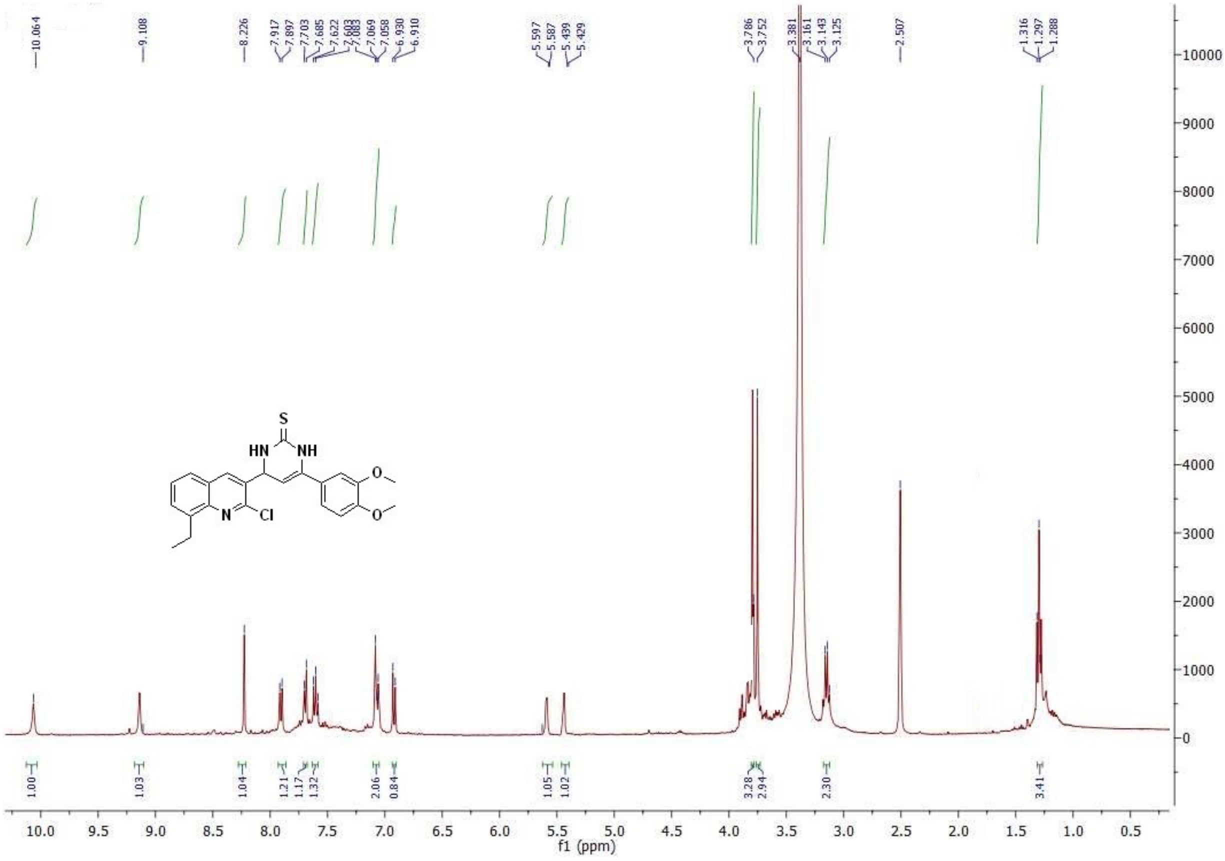

Fig. S57: ^{1}H NMR (400 MHz, DMSO-d_6) magnified spectrum of the compound **6b.**

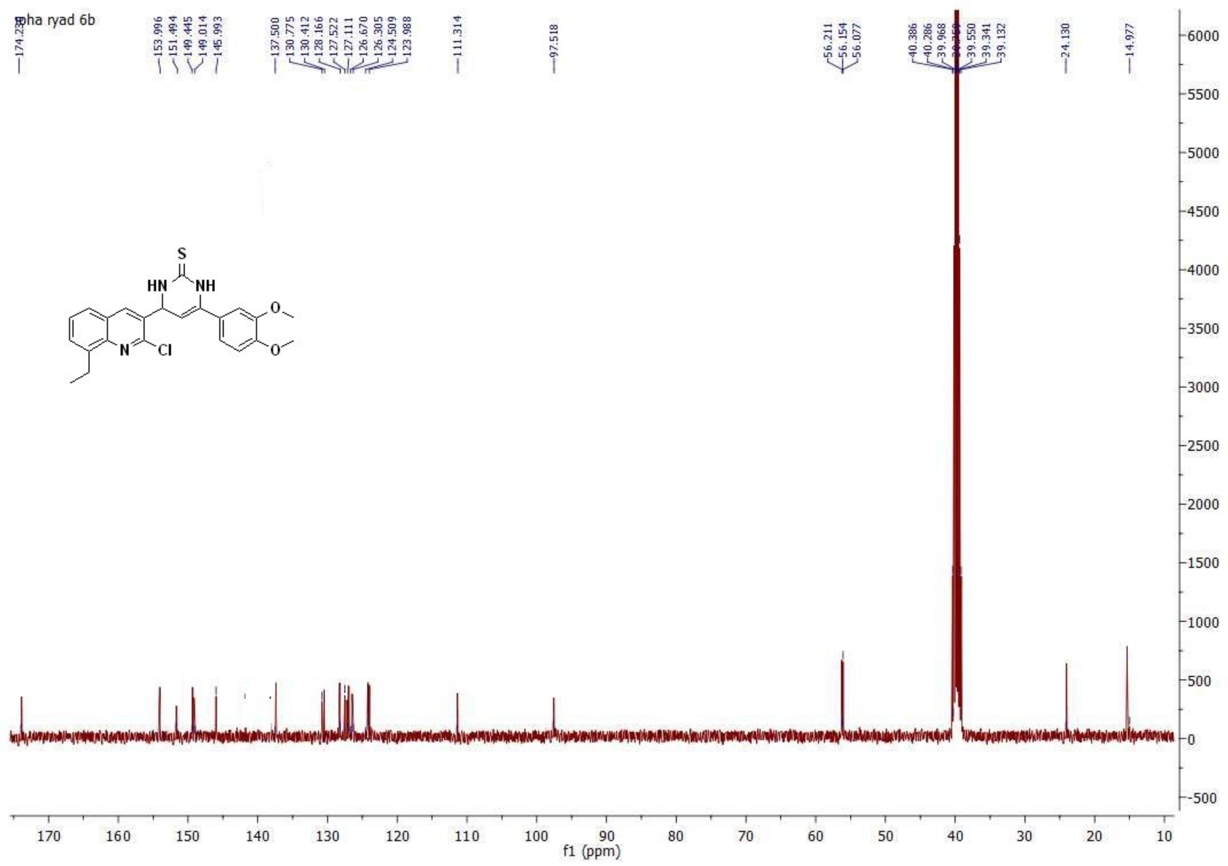

Fig. S58: ^{13}C NMR (100 MHz, DMSO-d_6) spectrum of the compound **6b.**

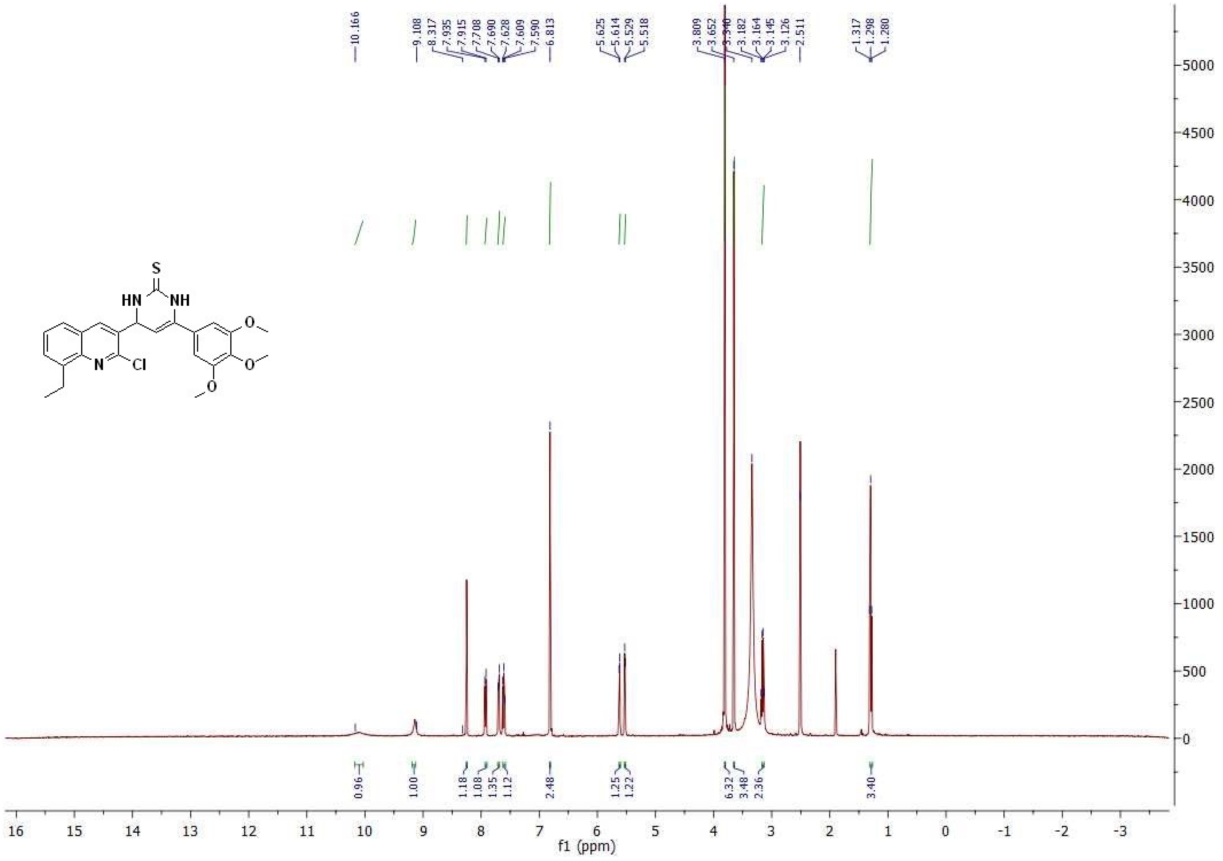

Fig. S59: ^{1}H NMR (400 MHz, DMSO-d_6) spectrum of the compound **6c.**

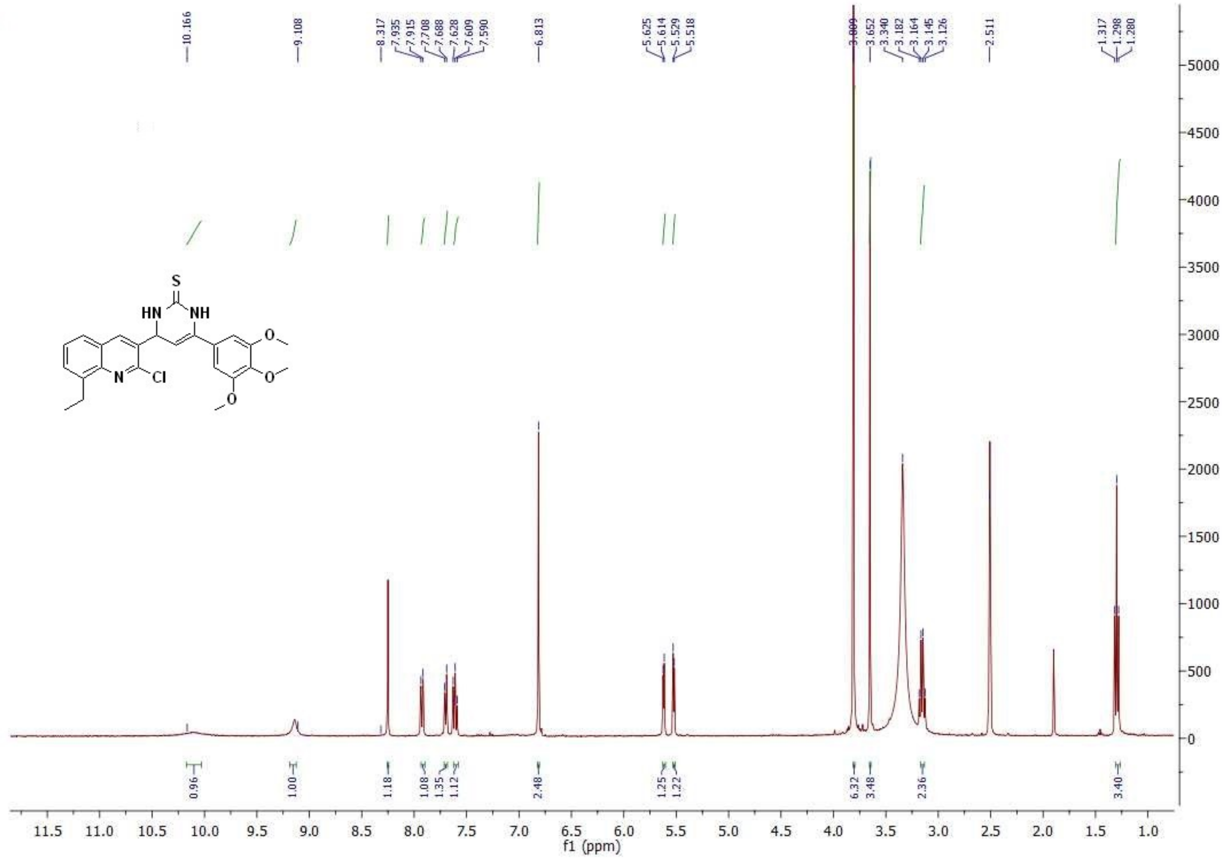

Fig. S60: ^{1}H NMR (400 MHz, DMSO-d_6) spectrum of the compound **6c.**

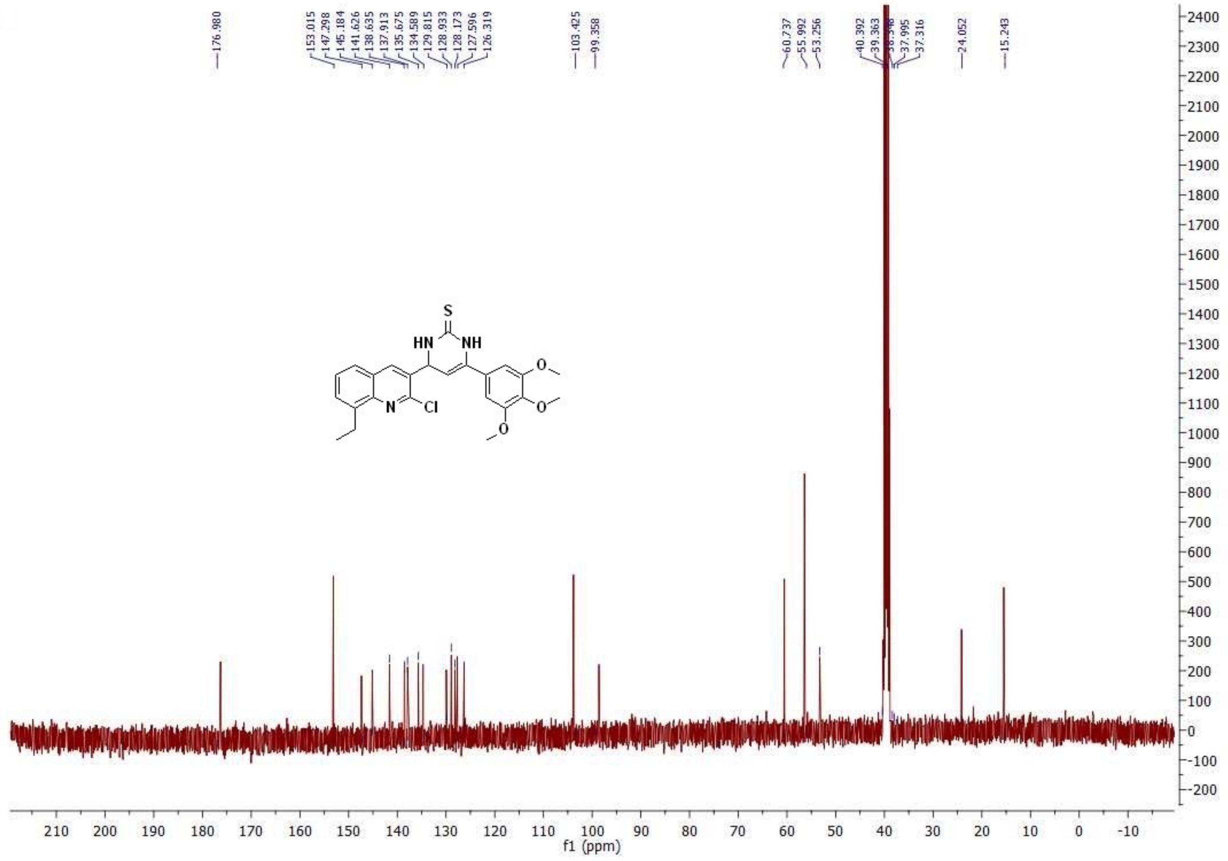

Fig. S61: ^{13}C NMR (100 MHz, DMSO-d_6) spectrum of the compound **6c.**

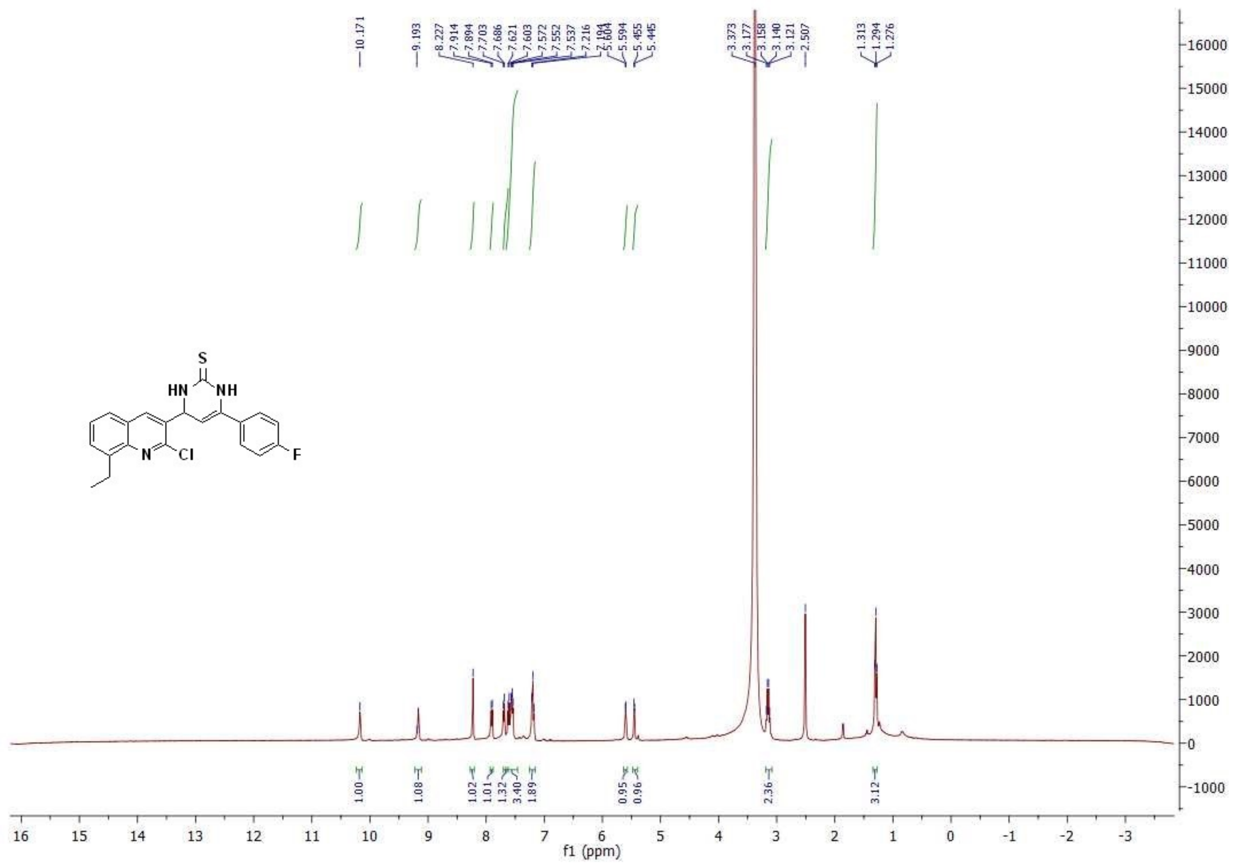

Fig. S62: ^{1}H NMR (400 MHz, DMSO-d_6) spectrum of the compound **6d.**

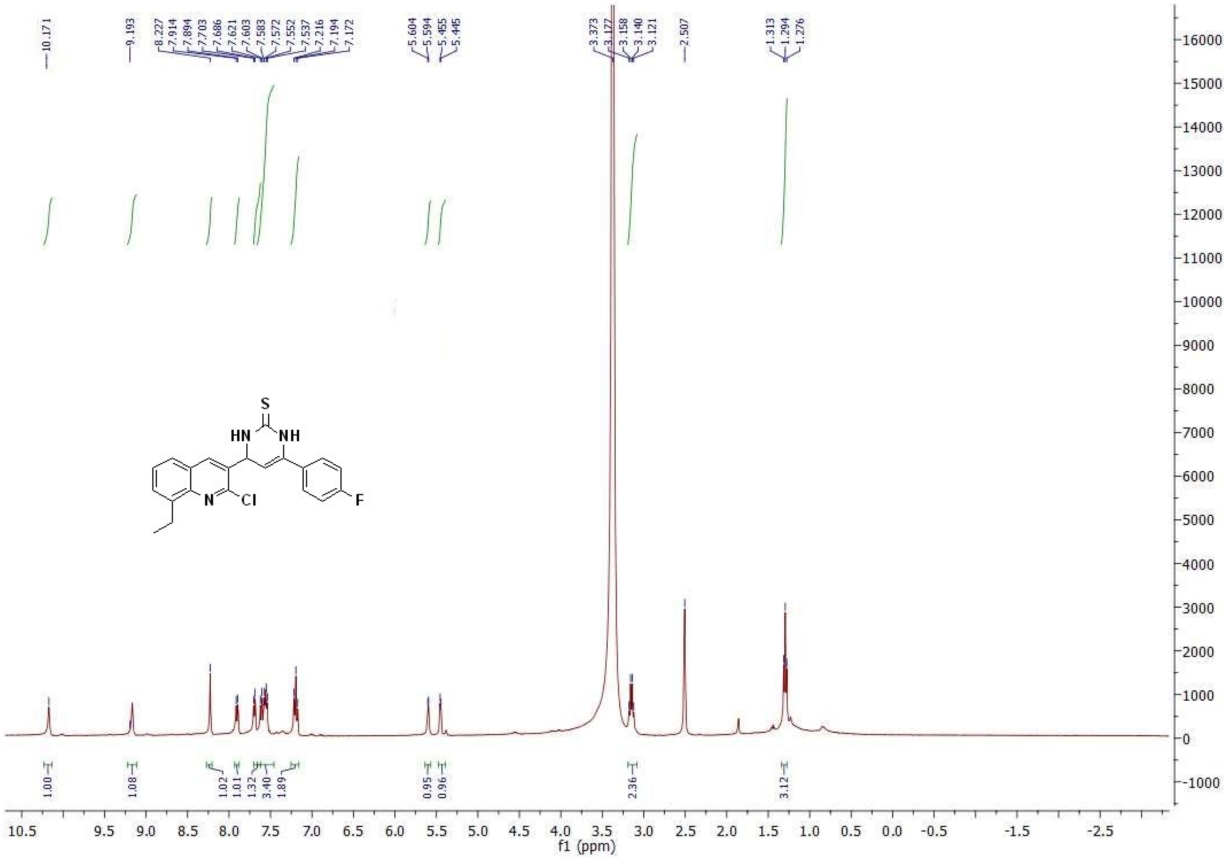

Fig. S63: ^{1}H NMR (400 MHz, DMSO-d_6) magnified spectrum of the compound **6d.**

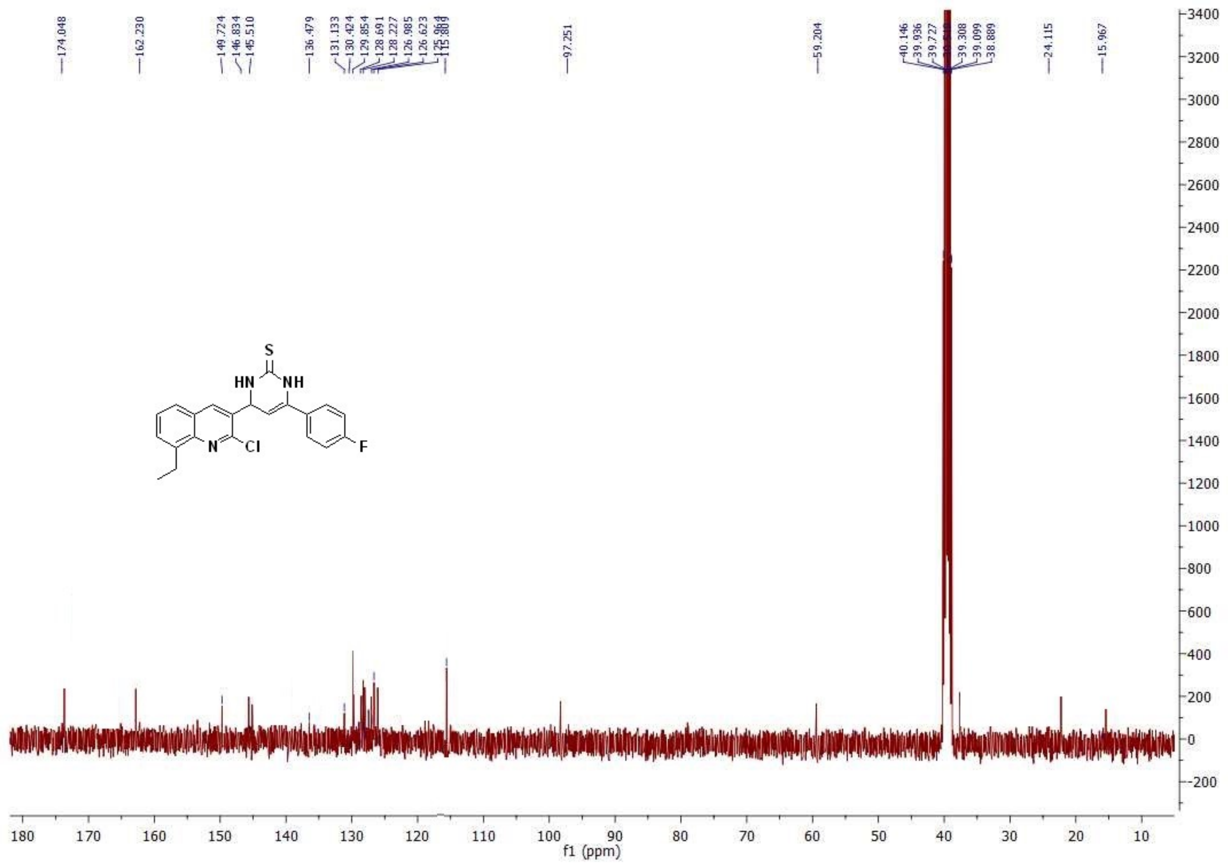

Fig. S64: ^{13}C NMR (100 MHz, DMSO-d_6) spectrum of the compound **6d.**

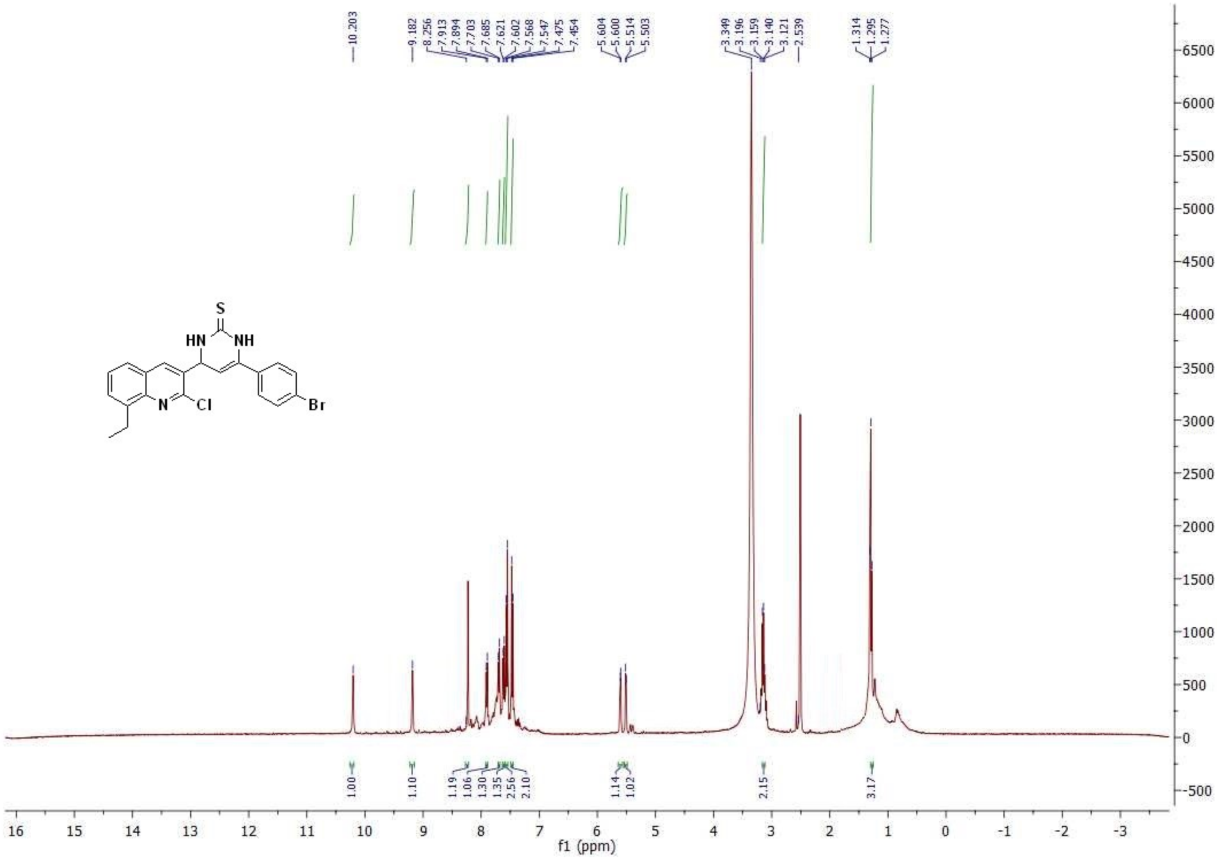

Fig. S65: ^{1}H NMR (400 MHz, DMSO-d_6) spectrum of the compound **6e.**

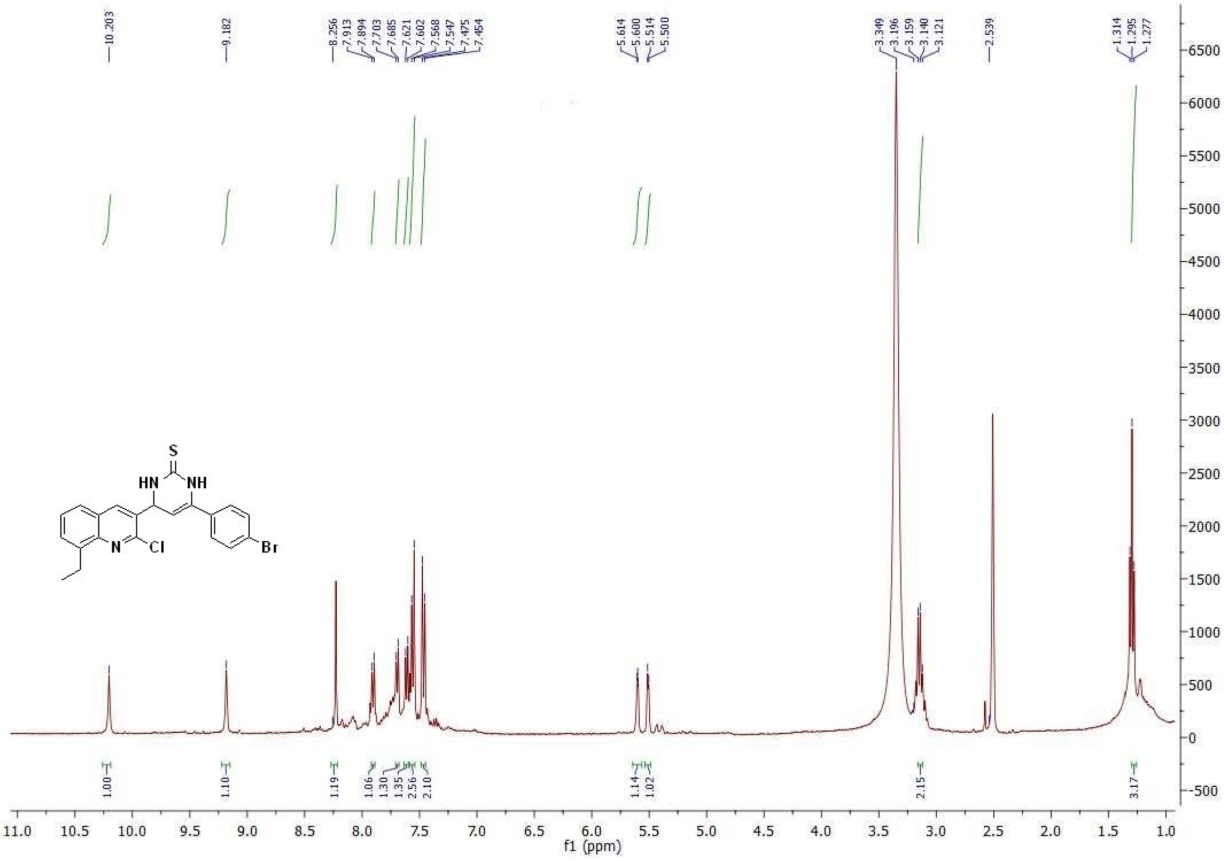

Fig. S66: ^{1}H NMR (400 MHz, DMSO-d_6) magnified spectrum of the compound **6e.**

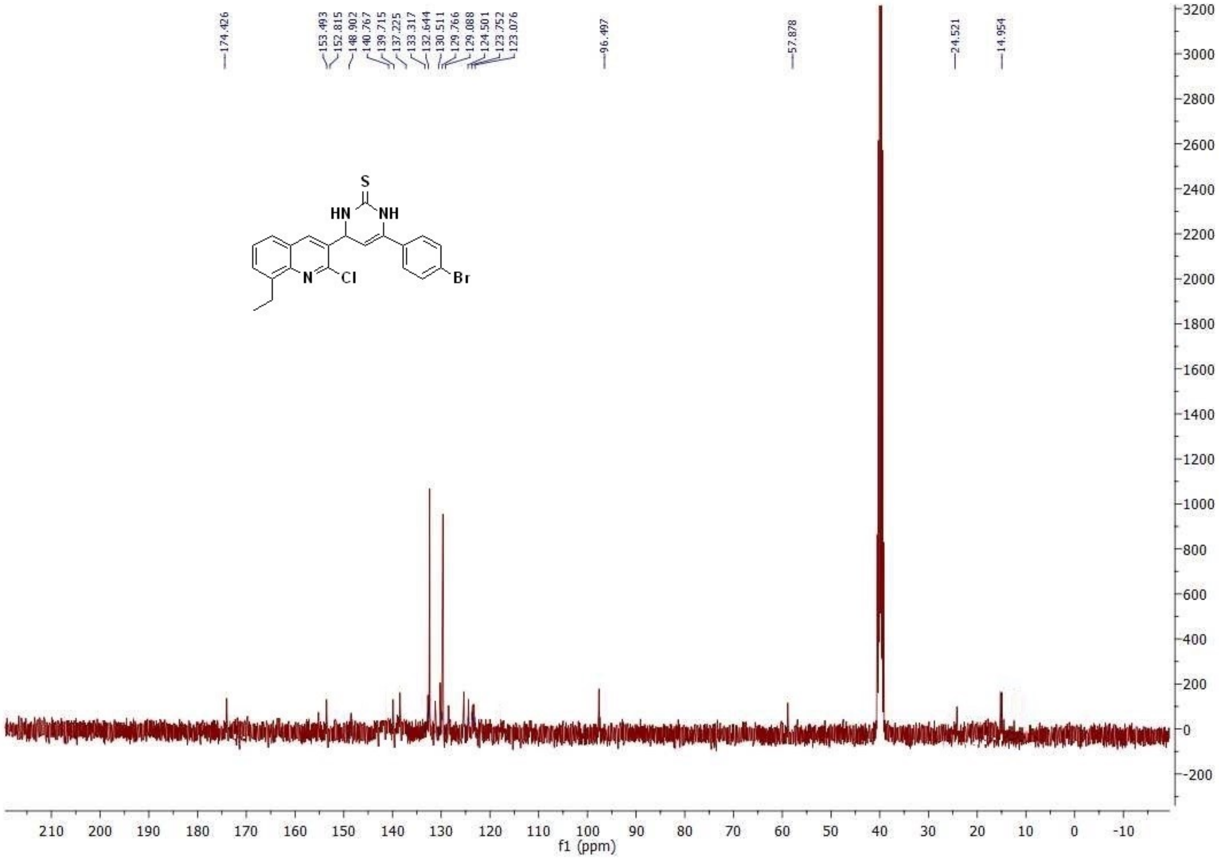

Fig. S67: ^{13}C NMR (100 MHz, DMSO-d_6) spectrum of the compound **6e.**

The biological studies

Table S1: *In vitro* growth inhibition (GI%) for the NCI60 cancer cell lines upon treatment with 10 µM of Compounds (3a-e and 4a-e).

	3a	3b	3c	3d	3e	4a	4b	4c	4d	4e
**Leukaemia**										
CCRF-CEM	26.85	64.95	87.78	a	a	a	a	**80.39**	a	a
HL-60(TB)	45.75	61.03	73.45	a	a	a	a	71.28	a	a
K-652	a	55.74	68.13	a	a	19.93	20.02	31.45	27.17	33.46
MOLT-4	16.79	49.22	49.91	a	a	11.88	a	76.43	15	a
RPMI-8226	22.15	56.17	78.23	a	a	a	a	74.51	14.03	a

SR	38.39	**80.06**	61.54	a	ND	a	a	77.32	ND	ND
**Non-small cell lung cancer**										
A549/ATCC	a	43.48	52.07	a	a	a	a	88.16	a	a
EKVX	a	a	17.12	a	a	12.28	14.24	50.12	a	a
HOP-62	a	a	A	a	a	a	a	87.86	a	a
HOP-92	27.31	27.39	26.83	a	a	29.84	11.29	**L**	a	a
NCI-H226	79.88	**L**	**L**	a	a	15.4	a	**L**	14.27	a
NCI-H23	a	20.25	27.39	a	a	a	a	68.61	12.04	a
NCI-H322M	a	22.35	31.08	a	a	a	a	53.67	a	a
NCI-H460	a	27.68	18.35	a	a	a	a	58.58	a	a
NCI-H522	21.13	41.47	80.68	a	a	11.87	14.74	**L**	a	a
**Colon Cancer**										
COLO 205	a	22.4	18.42	a-	a	a	a	a	a	a
HCC-2998	a	17.71	63.82	a	a	a	a	46.95	a	a
HCT-116	a	62.78	61.58	a	a	a	a	69.06	a	a
HCT-15	a	20.39	44.52	a	a	a	a	35.78	11.14	a
HT29	a	26.93	77.4	a	a	a	a	a		a
KM12	a	23.52	80.66	a	a	a	a	55.14	a	a
SW-620	a	72.24	65.44	a	a	a	a	17.43	a	a
**CNS Cancer**										
SF-268	a	a	32.21	a	a	21.44	16.08	34.36	a	a
SF-295	a	21.83	31.32	a	a	16.73	a	96.38	a	a
SF-539	18.88	99.38	**L**	11.01	a	a	a	**L**	a	a
SNB-19	a	54.44	66.41	a	a	a	a	89.58	a	a
SNB-75	a	**L**	61.72	a	a	45.69	29.64	**L**	a	a
U251	17.87	65.25	78.15	a	a	a	a	**L**	a	a
**Melanoma**										
LOX IMVI	11.32	43.44	71.98	a	a	a	a	91.58	15.3	a

MALME-3M	a	a	14.77	a	a	a	a	89.41	a	a
M14	a	a	31.93	a	a	a	a	42.76		a
MDA-MB-435	a	17.15	59.18	a	a	a	a	56.48	a	a
SK-MEL-2	a	a	44.49	a	a	a	a	29.76	a	a
SK-MEL-28	a	a	15.04	a	a	a	a	68.91	a	a
SK-MEL-5	a	23.24	39.88	a	a	13.93	a	78.58	16.75	12.97
UACC-257	a	a	43.59	a	a	a	a	26.54	a	a
UACC-62	17.15	35.88	41.92	a	a	a	a	**L**	16.46	a
**Ovarian Cancer**										
IGROV1	a	a	a	a	a	a	a	66.3	a	a
OVCAR-3	a	31.69	**L**	a	a	a	a	**L**	a	a
OVCAR-4	13.47	42.33	41.97	a	a	21.9	14.03	**L**	11.03	10.78
OVCAR-5	a	12.51	26.77	a	a	a	a	33.33	a	a
OVCAR-8	a	57.79	84	a	a	a	a	92.11	a	a
NCI/ADR-RES	a	45.75	67.57	a	a	a	a	59.91	a	a
SK-OV-3	a	a	17.38	a	a	a	a	61.41	a	11.74
**Renal Cancer**										
786-0	a	25.74	41.69	a	a	11.45	a	**L**	a	a
A498	a	a	a	a	a	a	a	**L**	a	a
ACHN	a	24.48	32.01	a	a	a	a	75.69	a	a
CAKI-1	19.34	40.25	38.71	11.8	12.83	34.88	39.22	95.06	19.48	17.59
RXF 393	23.77	22.09	42.58	a	a	a	33.79	**L**	a	a
SN12C	a	23.68	42.34	a	a	a	a	**L**	a	a
TK-10	a	a	a	a	a	a	a	**L**	a	a
UO-31	a	41.81	45.05	a	a	a	a	**L**	27.95	17.79
**Prostate Cancer**										
PC-3	a	32.05	53.5	a	a	a	a	67.59	a	a
DU-145	a	15.79	29.03	a	a	a	a	80.55	a	a

**Breast Cancer**										
MCF7	18.04	37.15	70.94	a	a	16.04	11.32	54.75	24.64	20.65
MDA-MB-231/ATCC	a	29.32	39.55	a	a	10.04	a	**L**	19.85	a
HS 578T	a	a	a	a	a	14.91	10.34	**L**	a	a
BT-549	22.52	50.34	76.12	a	a	15.31	a	57.52	17.79	27.68
T-47D	44.03	57.87	72.8	a	a	17.11	16.55	91.56	14.44	a
MDA-MB-468	a	39.76	61.14	a	a	13.97	a	51.91	a	a

a=GI % < 10

L= Letha

ND=Not Determined

Table S2: *In vitro* growth inhibition percent (GI%) for the NCI60 cancer cell lines upon treatment with 10 µM of Compounds (5a-e and 6a-e)

	**5a**	**5b**	**5c**	**5d**	**5e**	**6a**	**6b**	**6c**	**6d**	**6e**
**Leukaemia**										
CCRF-CEM	a	28.8	57.63	a	a	a	11	32.85	a	a
HL-60(TB)	14.94	a	51.76	a	a	a	10.73	41.55	a	a
K-652	28.78	27.99	33.86	29.26	a	a	10.53	44.64	27.55	a
MOLT-4	12.65	44.31	61.16	26.8	a	a	53.3	67.65	48.79	26.25
RPMI-8226	15.71	29.16	63.06	23.03	14.71	a	36.1	57.59	19.32	10.65
SR	11.58	a	49.05	ND	ND	ND	ND	ND	ND	ND
**Non-small cell lung cancer**										
A549/ATCC	a	19.71	38.11	a	a	a	a	26.33	a	a
EKVX	10.28	23.9	34.88	19.65	a	a	a	37.93	a	a
HOP-62	a	a	10.62	a	a	a	10.96	10.46	15.15	a
HOP-92	36.16	47.37	77.38	11.4	a	a	a	28.27	a	a
NCI-H226	15.89	20.27	63.27	25.9	a	a	15.39	57.97	21.71	a
NCI-H23	11.79	20.32	33.67	16.45	a	a	a	25.78	11.77	a

NCI-H322M	17.55	a	33.97	12.42	a	a	a	18.3	a	a
NCI-H460	a	a	21.83	a	a	a	a	29.96	a	a
NCI-H522	19.63	a	65.68	11.75	a	a	22.15	50.3	25.04	a
**Colon Cancer**										
COLO 205	a	a	12.69	a	a	a	a	24.36	a	a
HCC-2998	a	a	33.01	a	a	a	a	26.08	a	a
HCT-116	14.77	a	27.1	a	a	a	17.19	41.18	21.51	10.5
HCT-15	a	12.16	28.96	14.84	a	a	a	30.95	26.18	a
HT29	a	-	20.39	a	a	a	a	36.36	28.26	11.92
KM12	a	19.91	37.2	a	a	a	a	28.05	13.15	a
SW-620	a	a	10.03	a	a	a	a	13.45	a	a
**CNS Cancer**										
SF-268	23.06	32.23	31.72	21.02	a	a	16.63	42.42	14.86	a
SF-295	13.79	16.52	30.97	12.28	a	a	a	29.97	a	a
SF-539	a	21.97	29.68	a	a	a	a	30.33	a	a
SNB-19	13.2	22.6	39.09	10.5	a	a	a	23.35	a	a
SNB-75	21.56	10.81	a	39.15	19.75	14.94	a	23.2	a	a
U251	a	13.68	30.97	a	a	a	a	26.6	a	a
**Melanoma**										
LOX IMVI	a	a	38.34	12.37	a	a	a	33.32	a	a
MALME-3M	a	a	a	a	a	a	a	a	a	a
M14	a	a	31.17	a	10.98	13.19	a	30.14	10.84	a
MDA-MB-435	a	a	24.92	a	a	a	a	43.45	a	a
SK-MEL-2	a	a	33.02	a	a	a	a	10.15	a	a
SK-MEL-28	a	a	a	a	a	a	a	-	a	a
SK-MEL-5	a	21.02	52.17	15.32	a	a	14.13	38.21	a	a
UACC-257	a	a	20.13	a	a	a	a	13.06	a	a
UACC-62	20.48	19.06	33.49	25.7	a	10.41	10.28	35.55	14.25	a

**Ovarian Cancer**										
IGROV1	12.91	a	33.4	28.58	a	a	a	14.31	a	a
OVCAR-3	a	a	16.86	-	a	a	a	14.21	a	a
OVCAR-4	24.5	22.4	41.62	13.35	a	a	19.9	48.88	21.02	10.18
OVCAR-5	a	a	a	a	a	a	a	A	a	a
OVCAR-8	10.13	31.46	36.71	a	a	a	a	12.25	a	a
NCI/ADR-RES	a	24.86	28.5	a	a	a	11.1	34.01	16.96	a
SK-OV-3	a	a	a	20.89	11.18	14.95	a	a	a	10.74
**Renal Cancer**										
786-0	a	18.71	27.85	a	a	a	a	18.24	a	a
A498	a	-	18.77	a	a	a	a	11.59	a	a
ACHN	a	15.69	32.54	a	a	a	a	24.38	a	a
CAKI-1	44.69	45.79	69.81	31.08	20.05	16.19	30.19	64.11	26.14	10.37
RXF 393	20.63	24.58	37.29	a	a	a	a	40.65	a	a
SN12C	10.35	21.7	39.67	a	a	a	a	21.68	a	a
TK-10	a	a	a	a	a	a	a	12.62	a	a
UO-31	19.73	23.28	45.23	40.15	24.05	18.59	37.17	67.70	49.53	32.66
**Prostate Cancer**										
PC-3	a	21.34	48.91	4.57	a	a	a	46.18	22.02	a
DU-145	a	20.2	32.26	-	a	a	a	21.68	a	a
**Breast Cancer**										
MCF7	20.75	26.65	46.53	40.43	19.71	a	15.73	44.31	24.62	18.42
MDA-MB-231/ATCC	18.14	19.15	52.51	31.21	12.89	a	a	46.06	a	a
HS 578T	19.77	23.11	32.67	18.23	a	a	12.89	39.64	a	a
BT-549	20.39	28.17	21.06	12.24	a	a	12.82	10.52	a	a
T-47D	12.67	41.08	60.59	21.1	10.4	a	24.95	48.61	23.88	a
MDA-MB-468	18.66	22.77	50.55	13.97	a	a	13.21	40.49	-	a

a=GI % < 10

L= Letha

ND=Not Determined

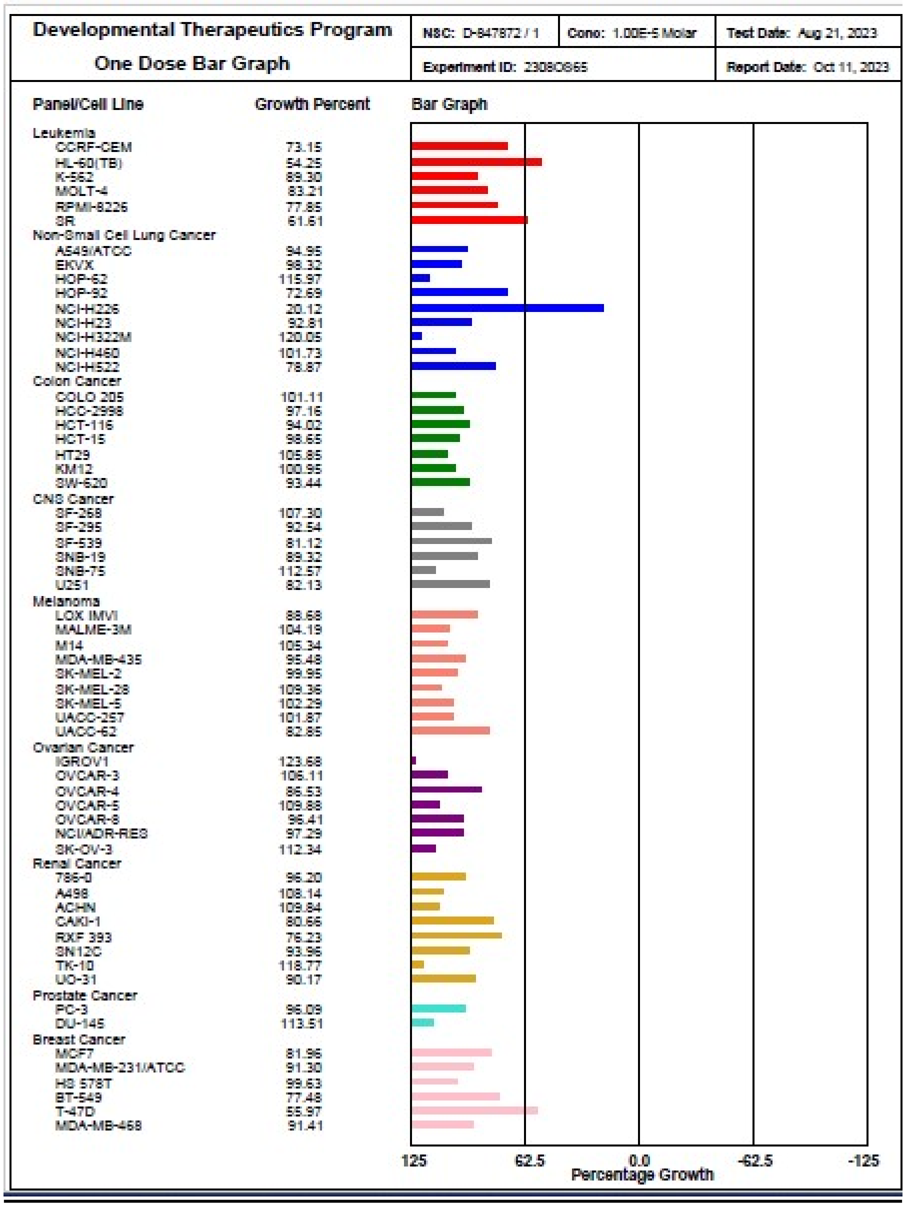

Fig. S68: ***In vitro*** **growth (G%) for the NCI60 cancer cell lines upon treatment with 10 µM of Compound 3a**

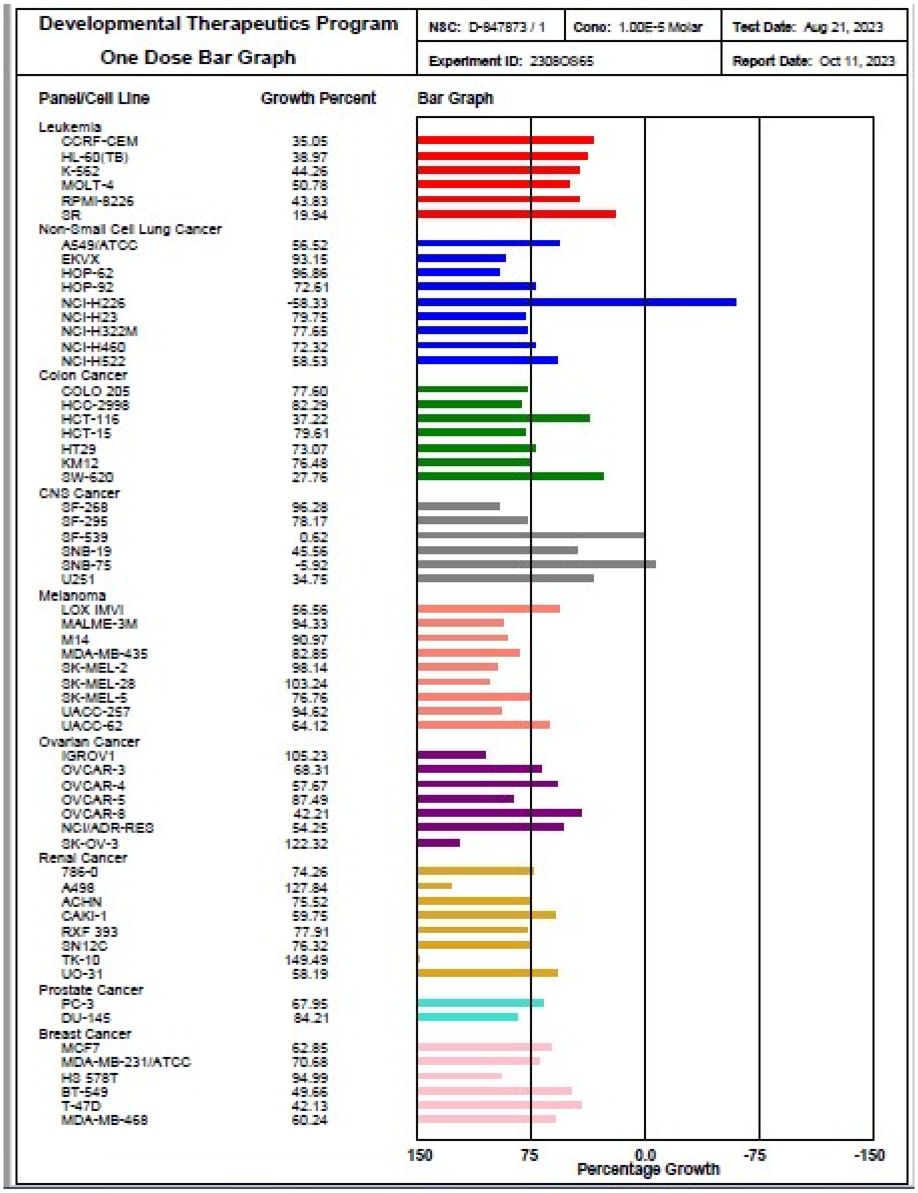

Fig. S69: ***In vitro*** **growth (G%) for the NCI60 cancer cell lines upon treatment with 10 µM of Compound 3b**

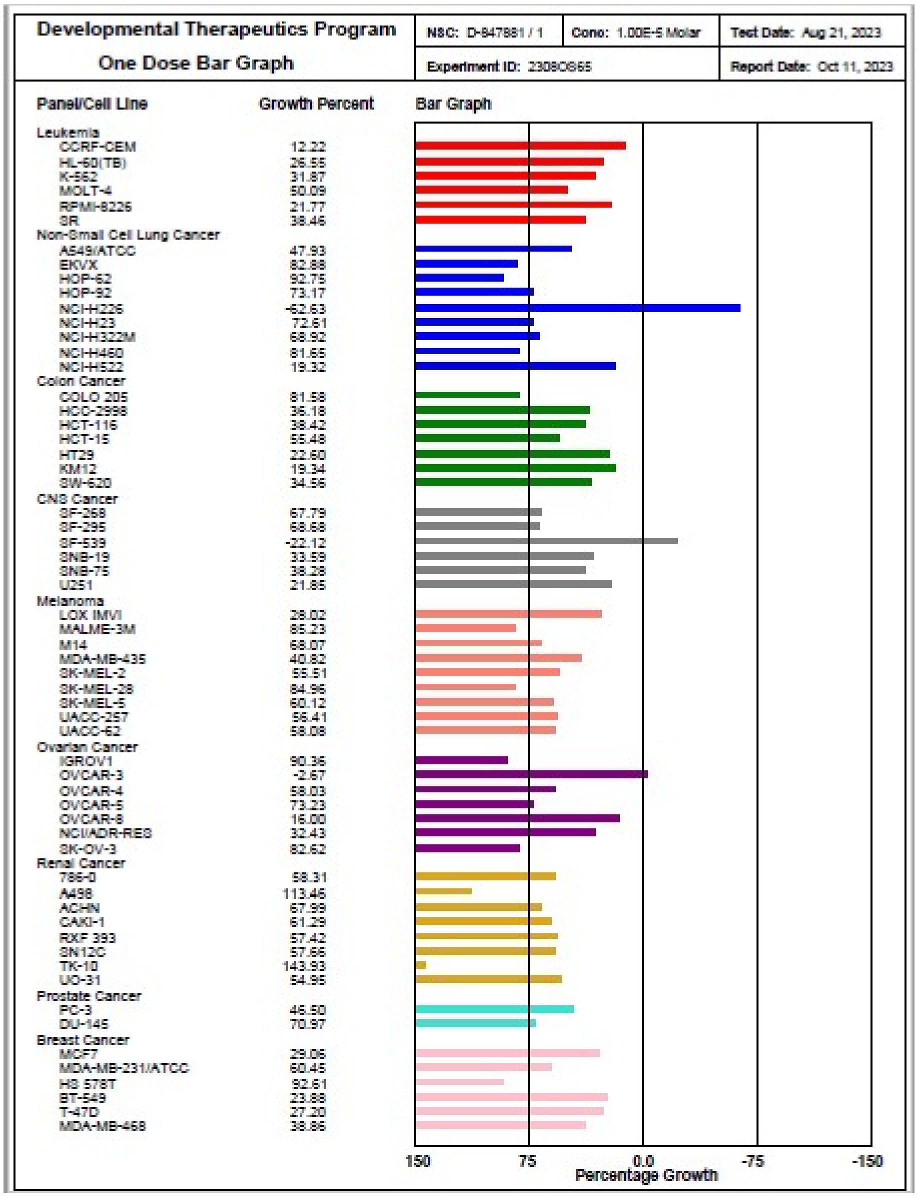

Fig. S70: ***In vitro*** **growth (G%) for the NCI60 cancer cell lines upon treatment with 10 µM of Compound 3c**

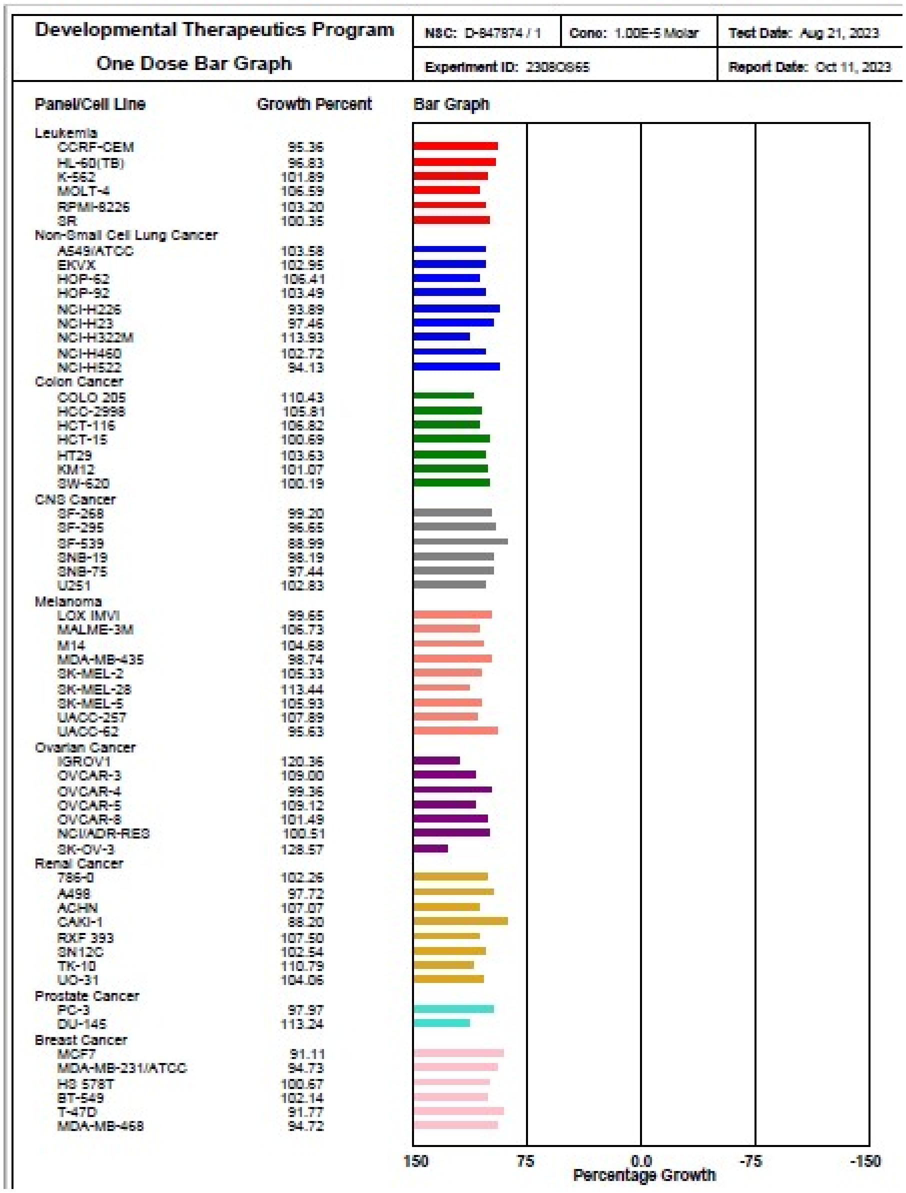

Fig. S71: ***In vitro*** **growth (G%) for the NCI60 cancer cell lines upon treatment with 10 µM of Compound 3d**

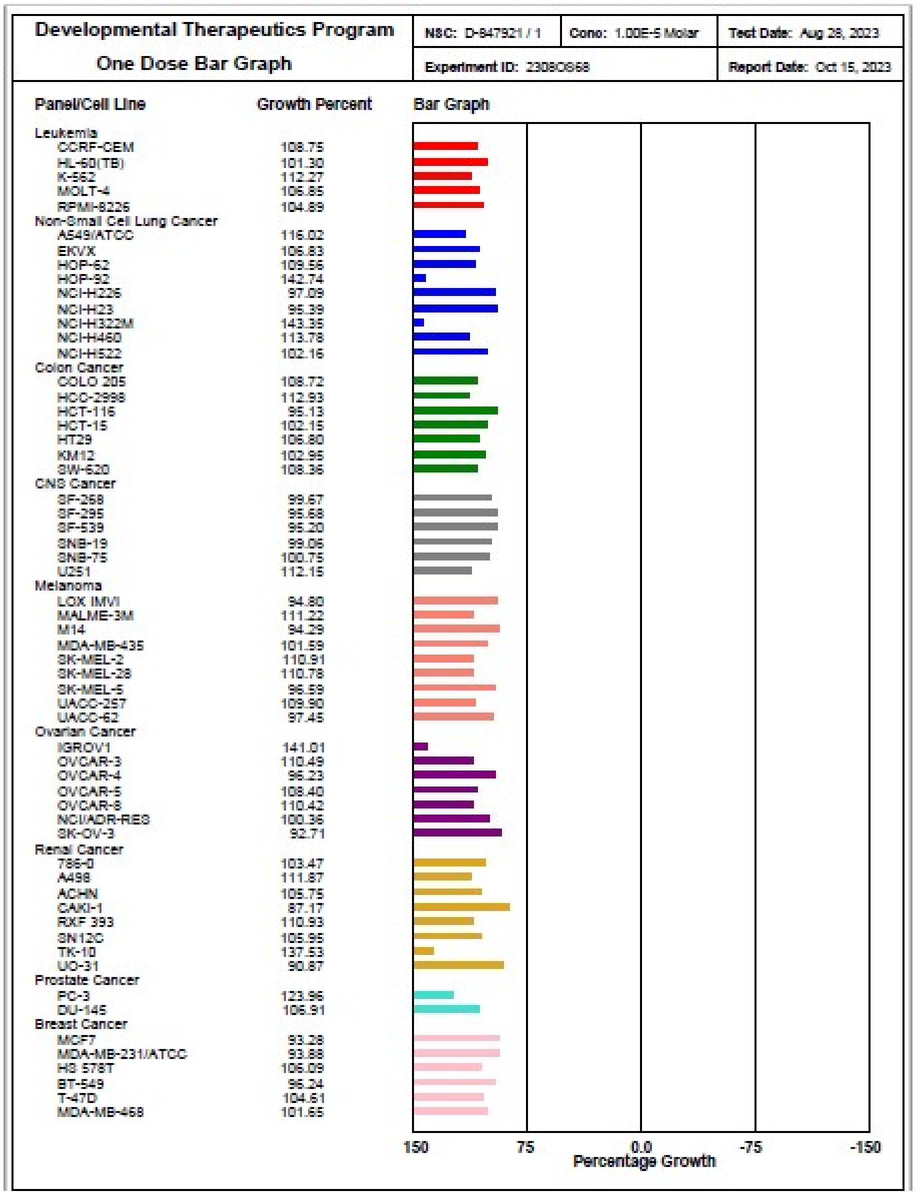

Fig. S72: ***In vitro*** **growth (G%) for the NCI60 cancer cell lines upon treatment with 10 µM of Compound 3e**

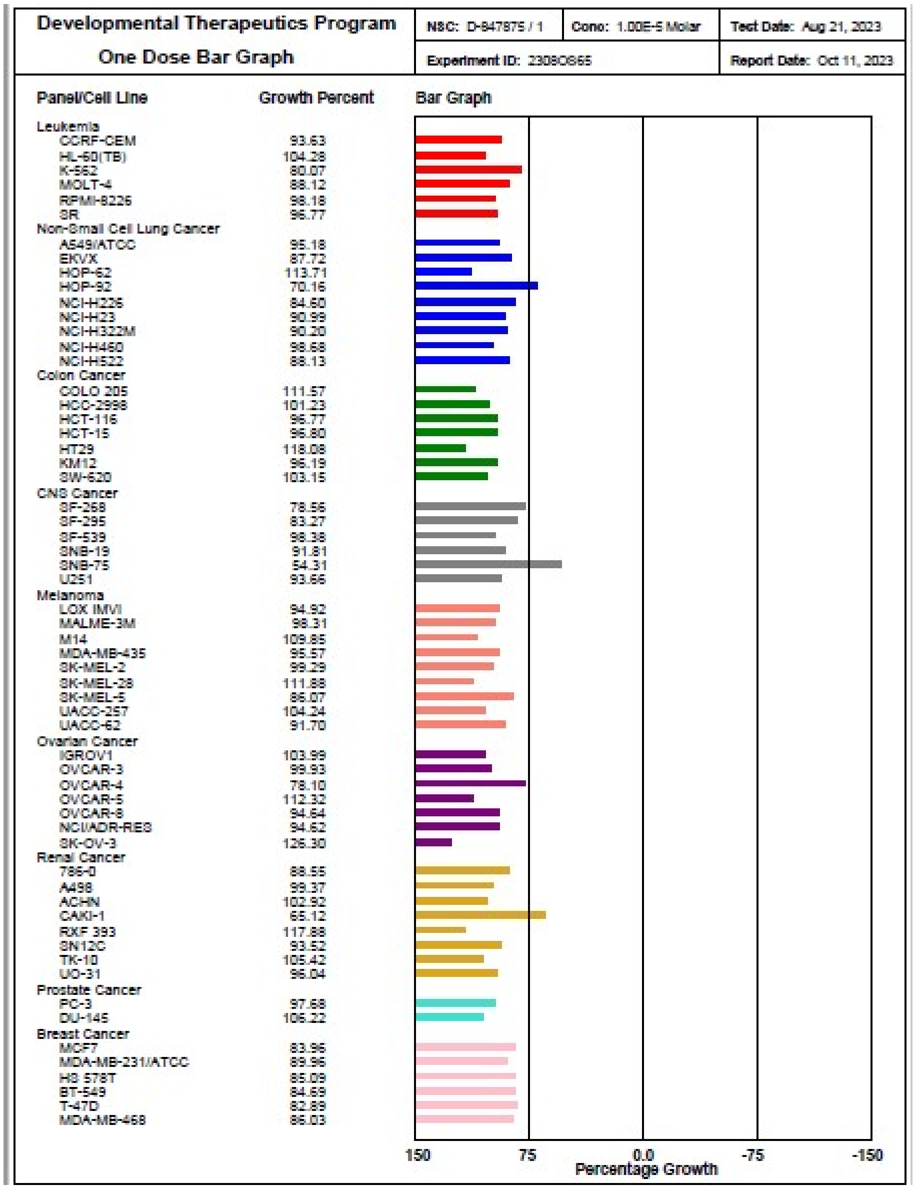

Fig. S73: ***In vitro*** **growth (G%) for the NCI60 cancer cell lines upon treatment with 10 µM of Compound 4a**

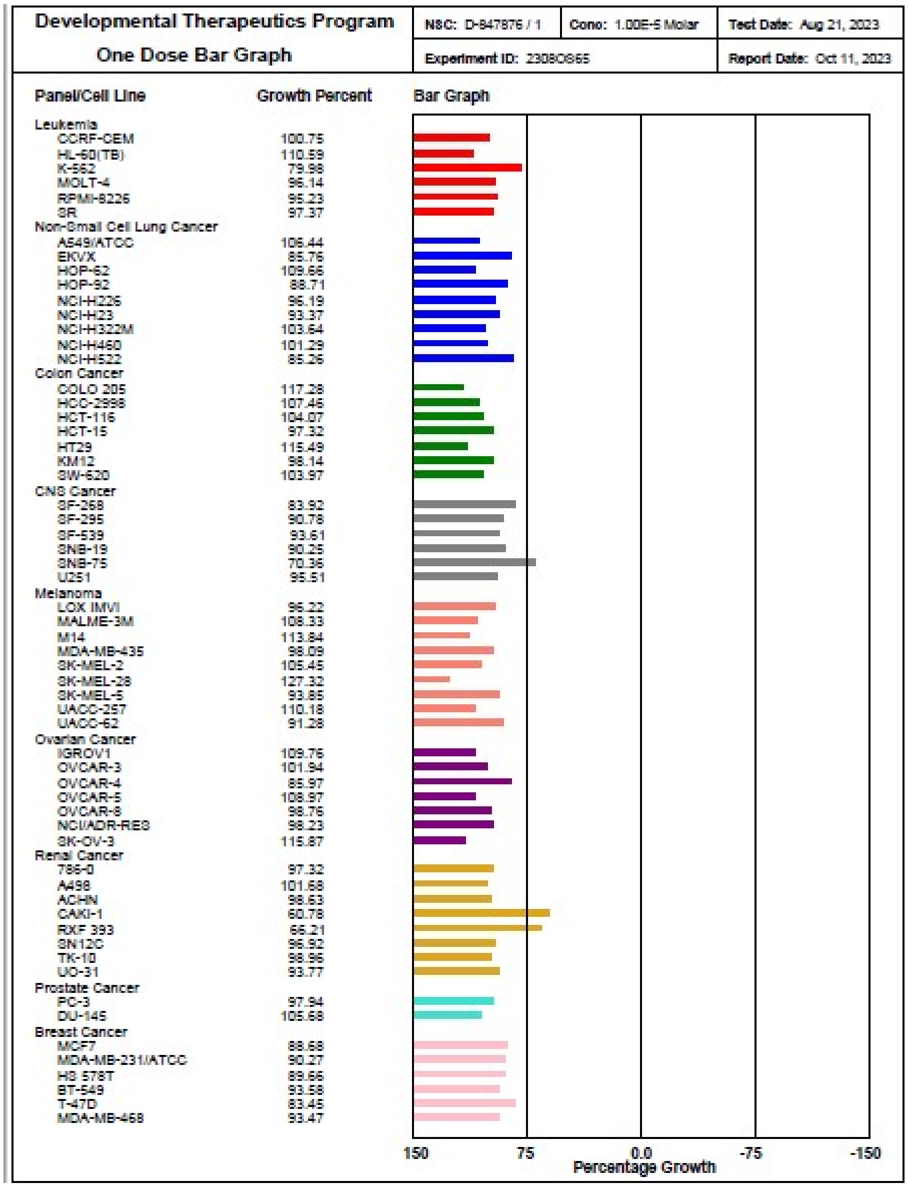

Fig. S74: ***In vitro*** **growth (G%) for the NCI60 cancer cell lines upon treatment with 10 µM of Compound 4b**

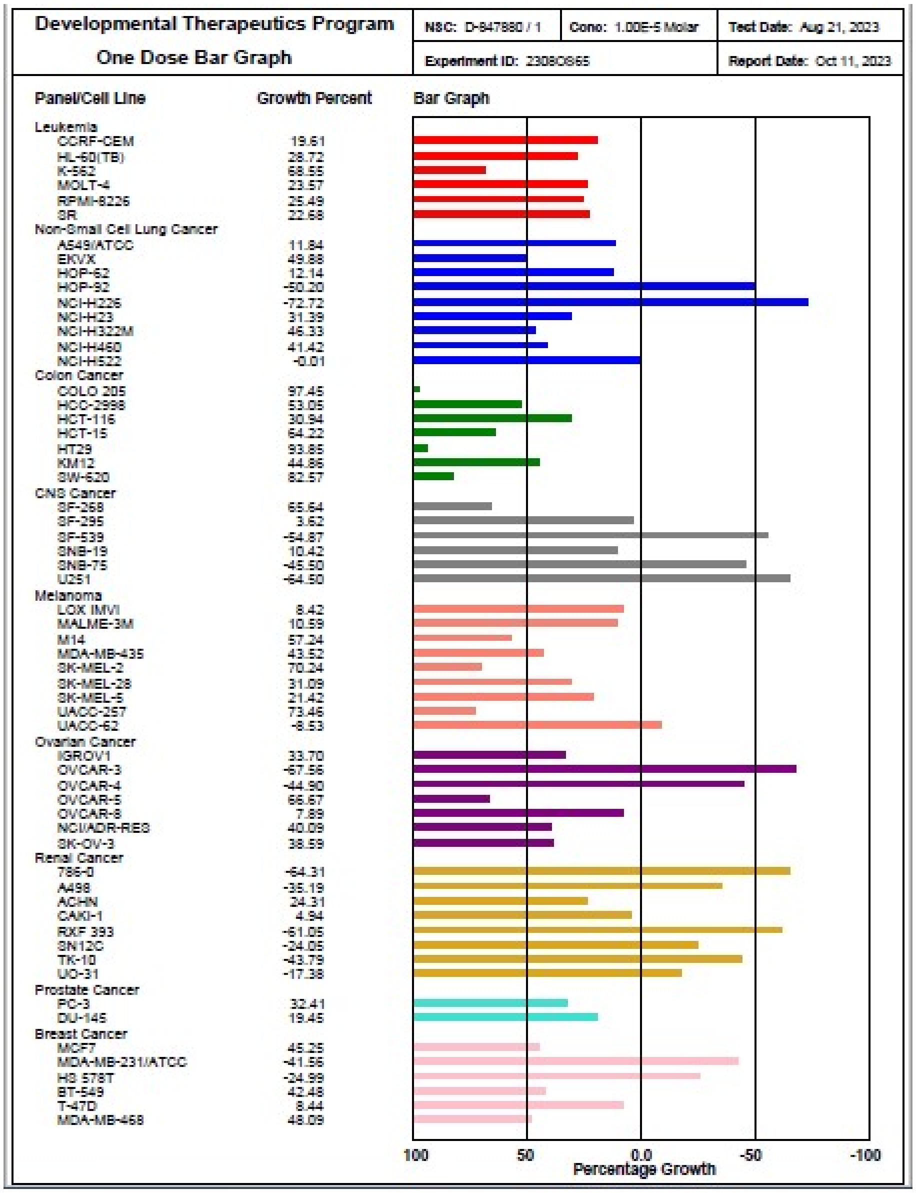

Fig. S75: ***In vitro*** **growth (G%) for the NCI60 cancer cell lines upon treatment with 10 µM of Compound 4c**

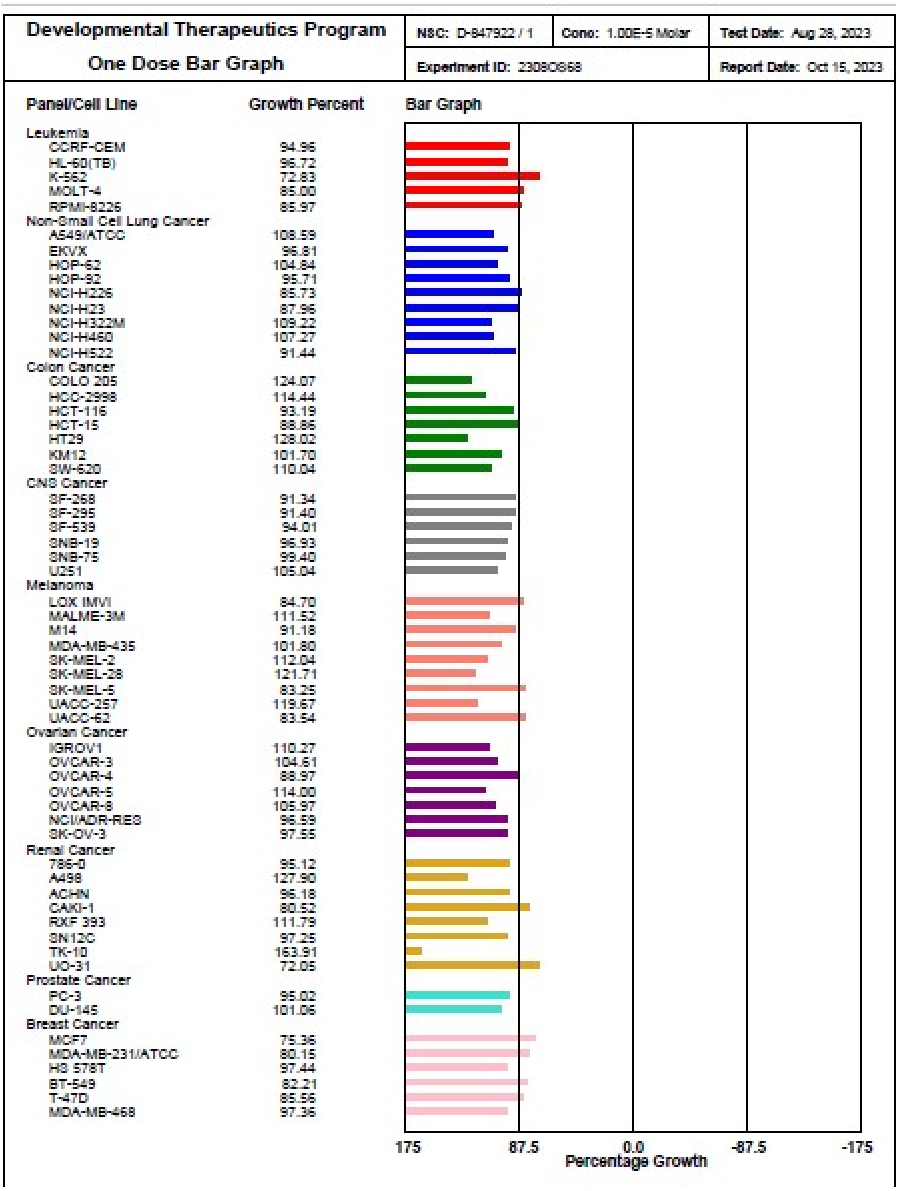

Fig. S76: ***In vitro*** **growth (G%) for the NCI60 cancer cell lines upon treatment with 10 µM of Compound 4d**

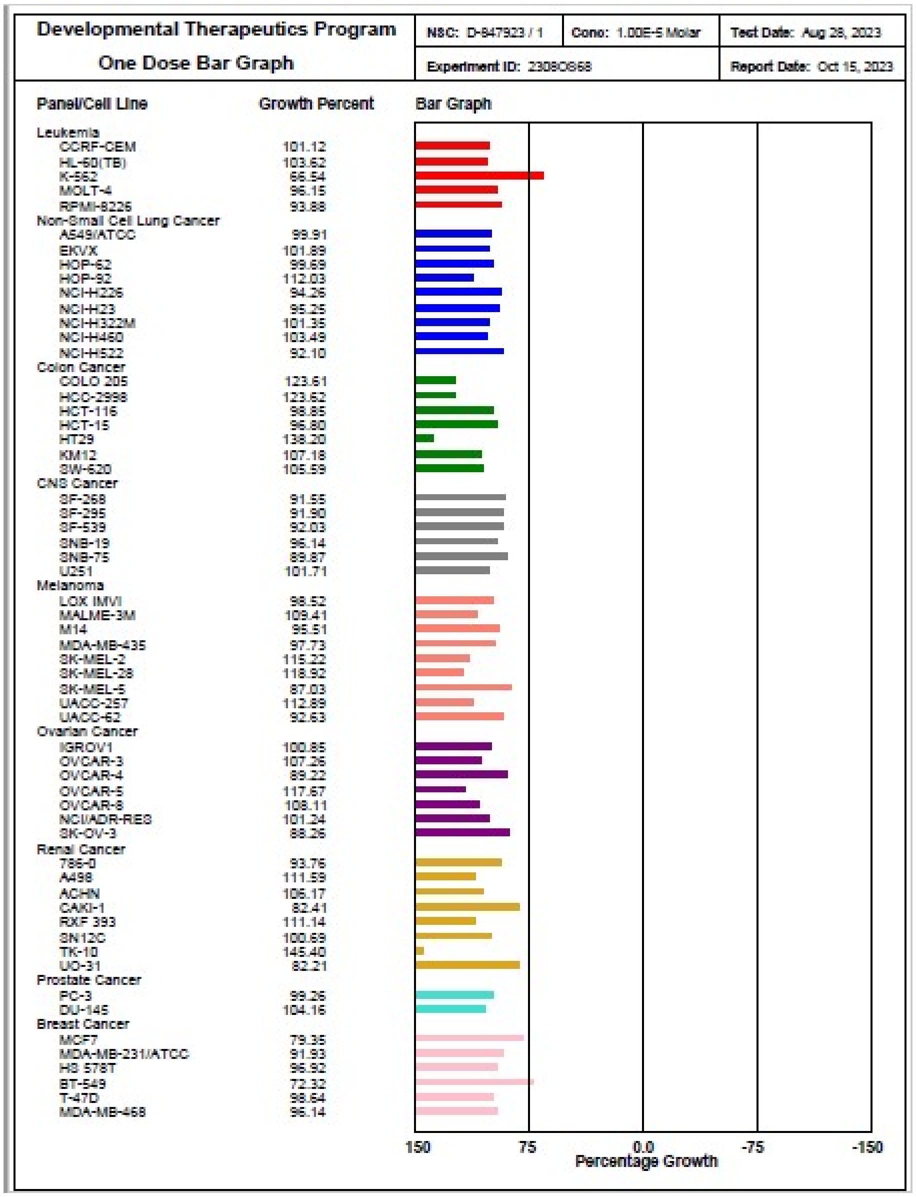

Fig. S77: ***In vitro*** **growth (G%) for the NCI60 cancer cell lines upon treatment with 10 µM of Compound 4e**

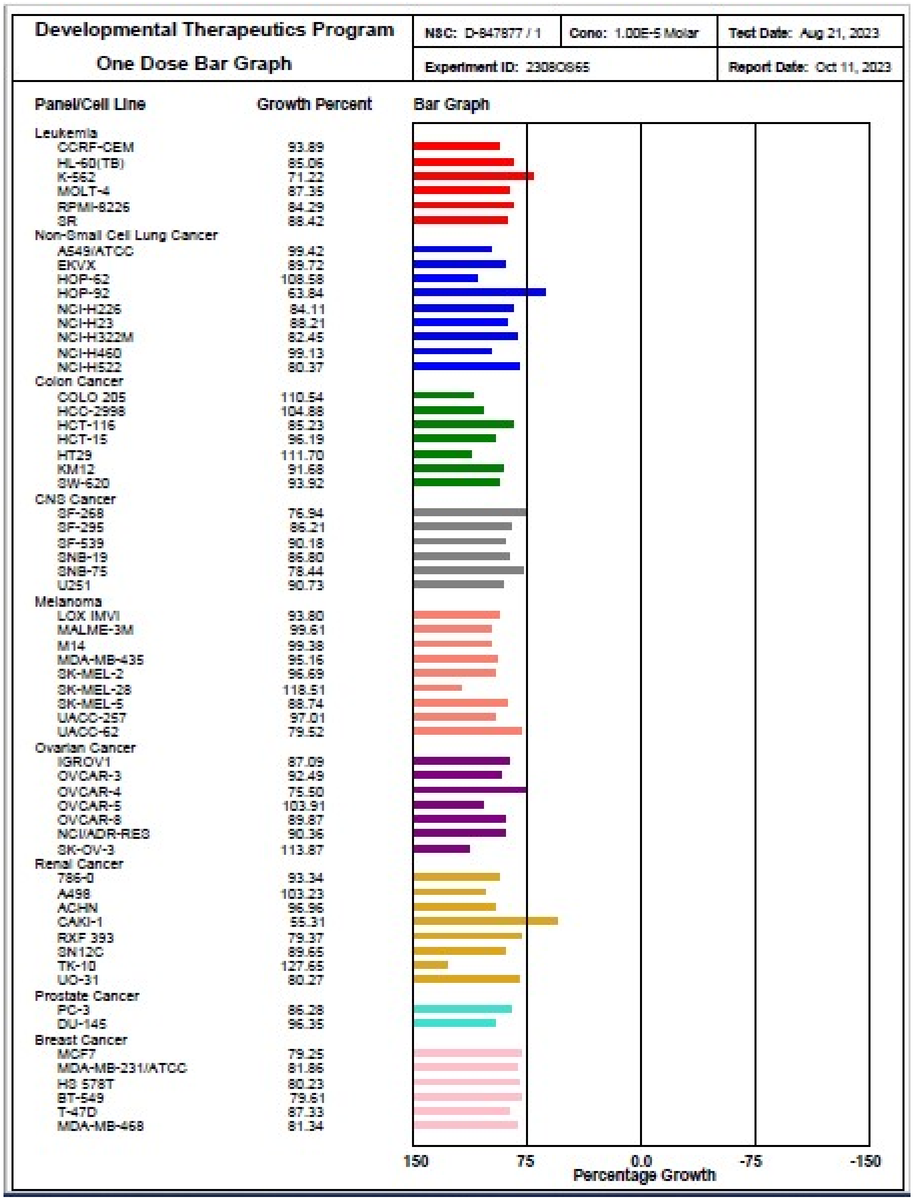

Fig. S78: ***In vitro*** **growth (G%) for the NCI60 cancer cell lines upon treatment with 10 µM of Compound 5a**

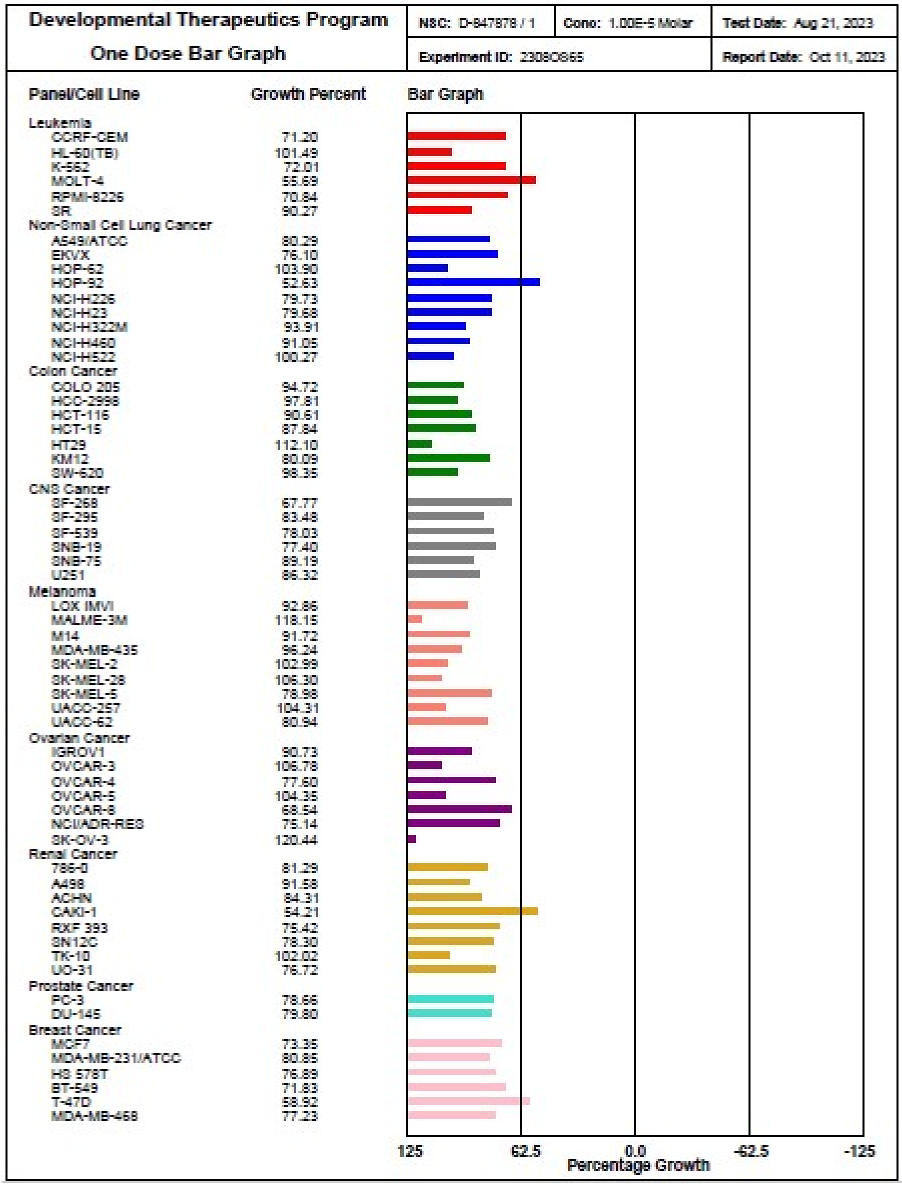

Fig. S79: ***In vitro*** **growth (G%) for the NCI60 cancer cell lines upon treatment with 10 µM of Compound 5b**

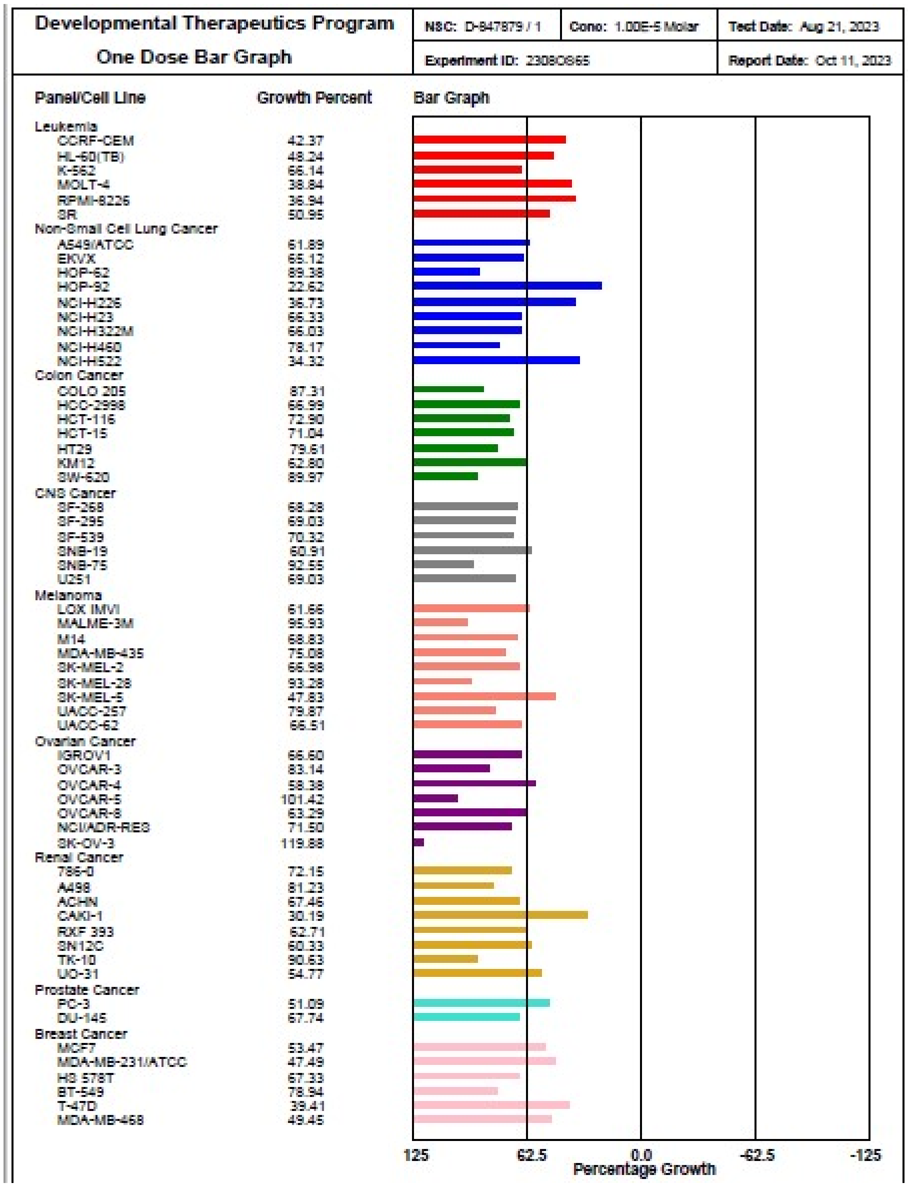

Fig. S80: ***In vitro*** **growth (G%) for the NCI60 cancer cell lines upon treatment with 10 µM of Compound 5c**

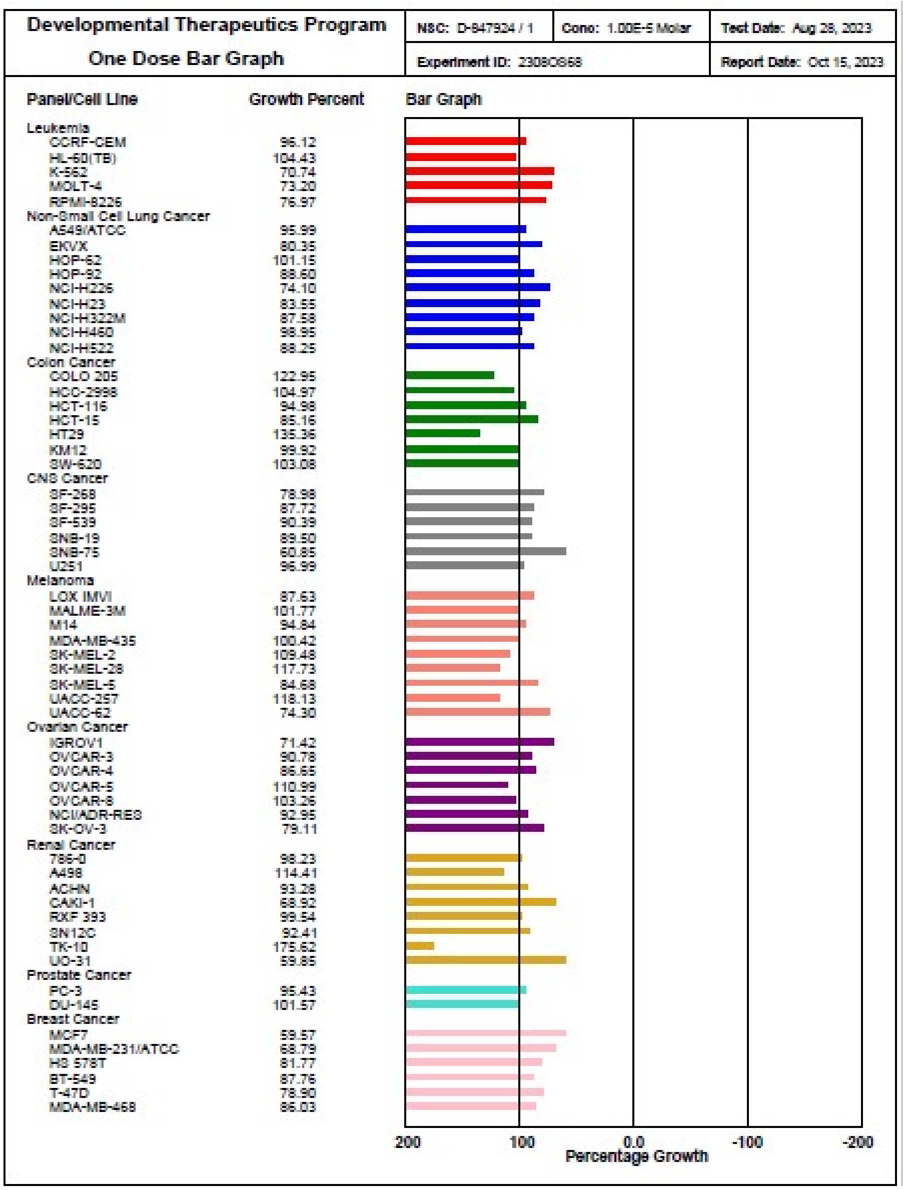

Fig. S81: ***In vitro*** **growth (G%) for the NCI60 cancer cell lines upon treatment with 10 µM of Compound 5d**

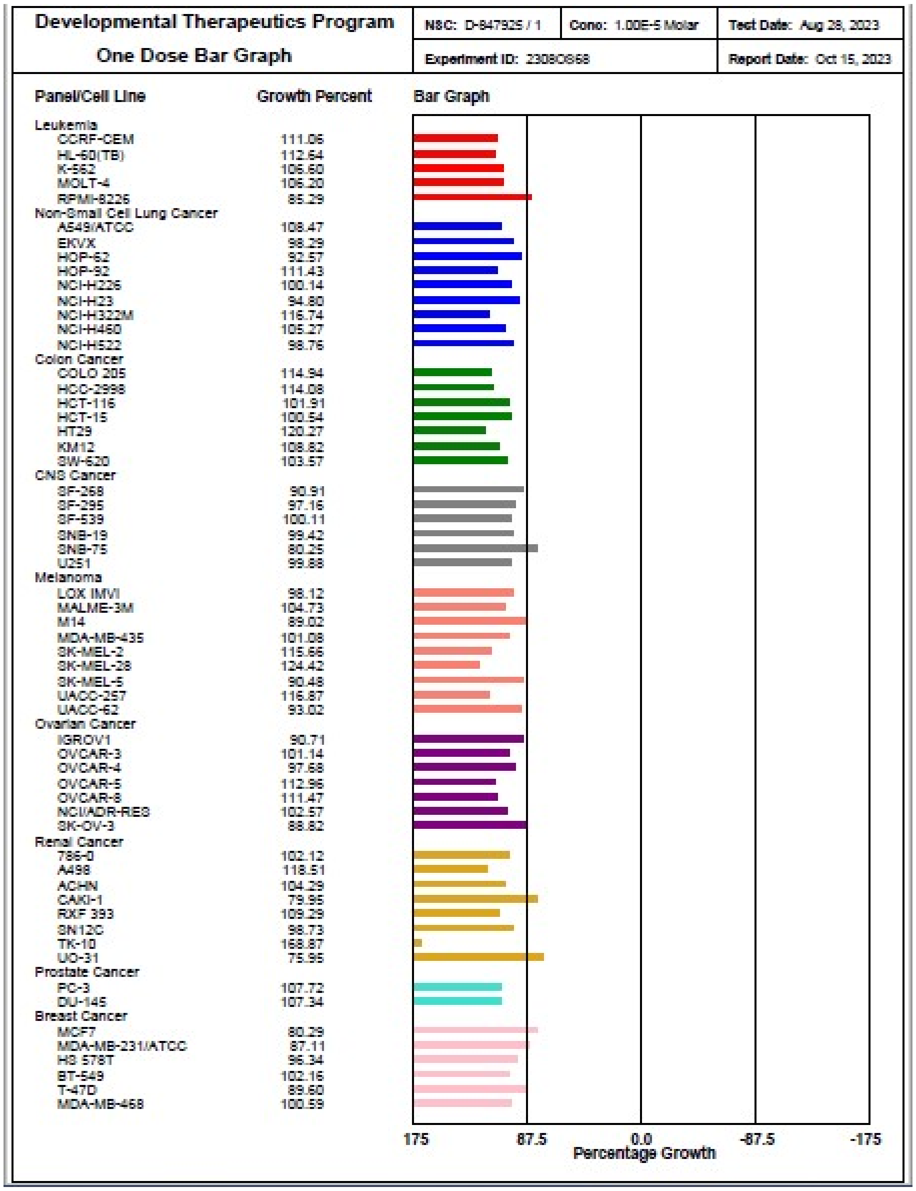

Fig. S82: ***In vitro*** **growth (G%) for the NCI60 cancer cell lines upon treatment with 10 µM of Compound 5e**

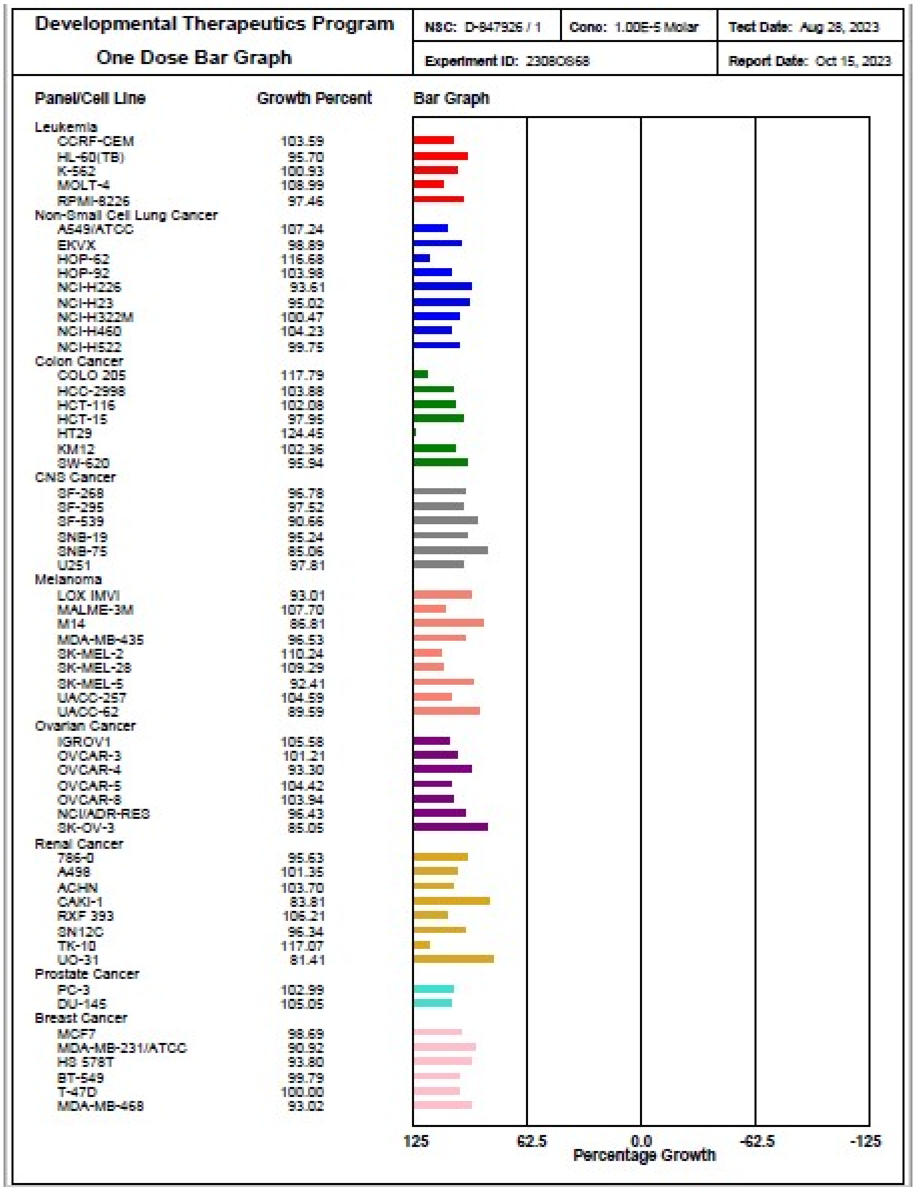

Fig. S83: ***In vitro*** **growth (G%) for the NCI60 cancer cell lines upon treatment with 10 µM of Compound 6a**

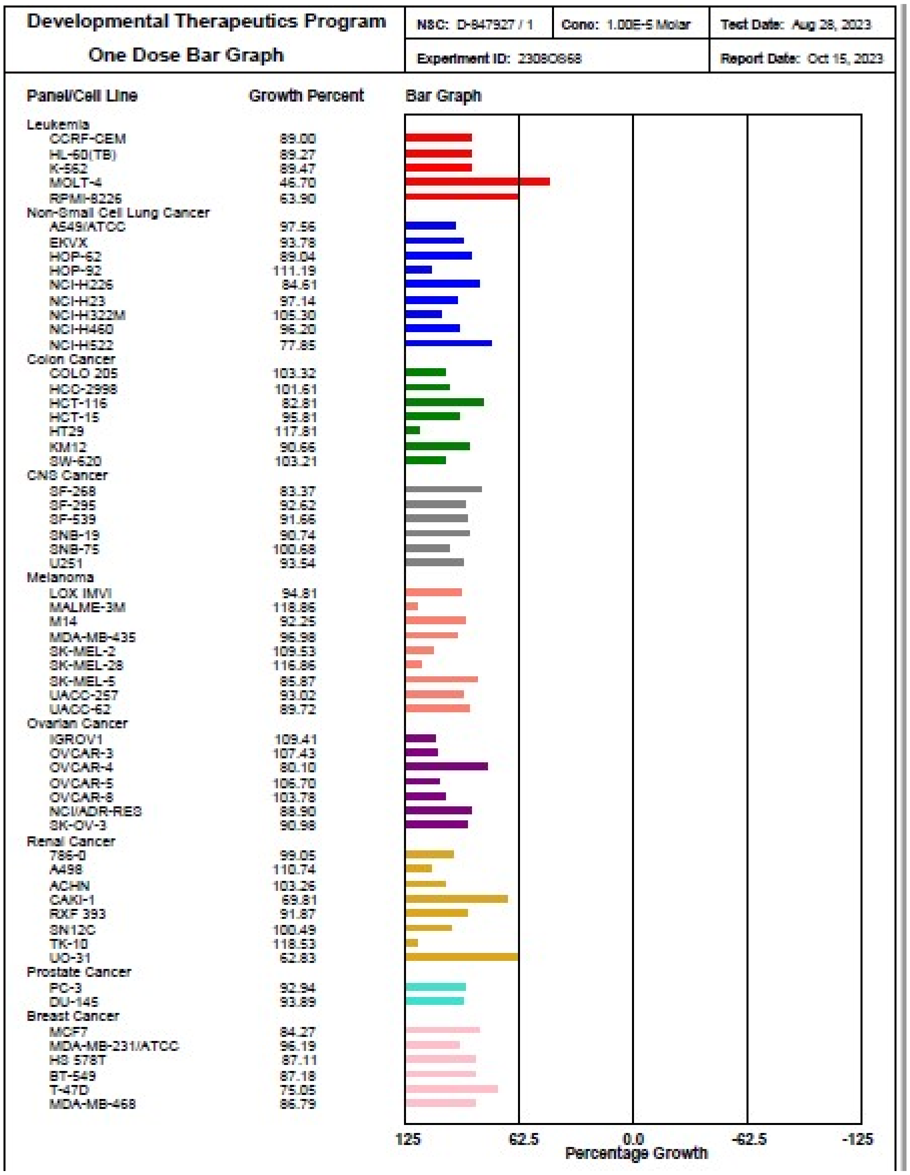

Fig. S84: ***In vitro*** **growth (G%) for the NCI60 cancer cell lines upon treatment with 10 µM of Compound 6b**

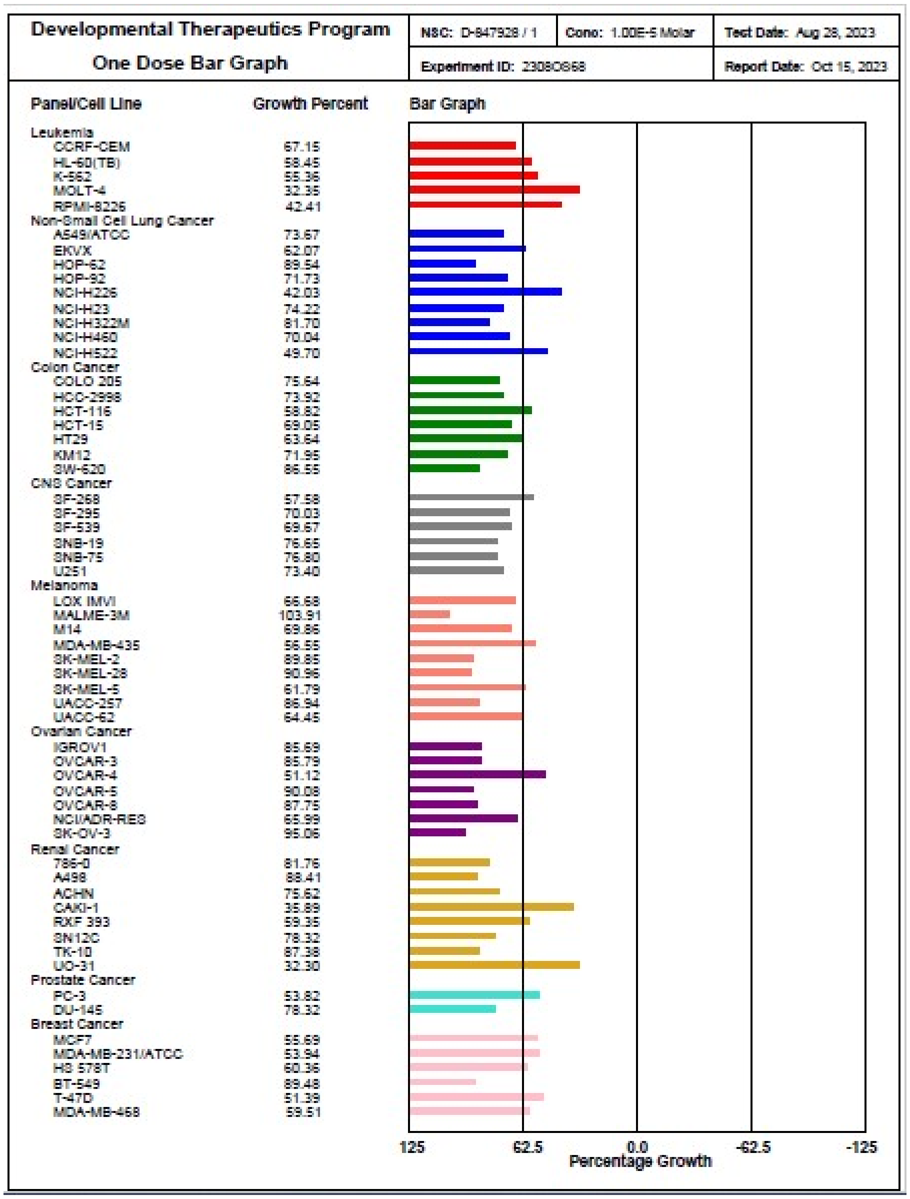

Fig. S85: ***In vitro*** **growth (G%) for the NCI60 cancer cell lines upon treatment with 10 µM of Compound 6c**

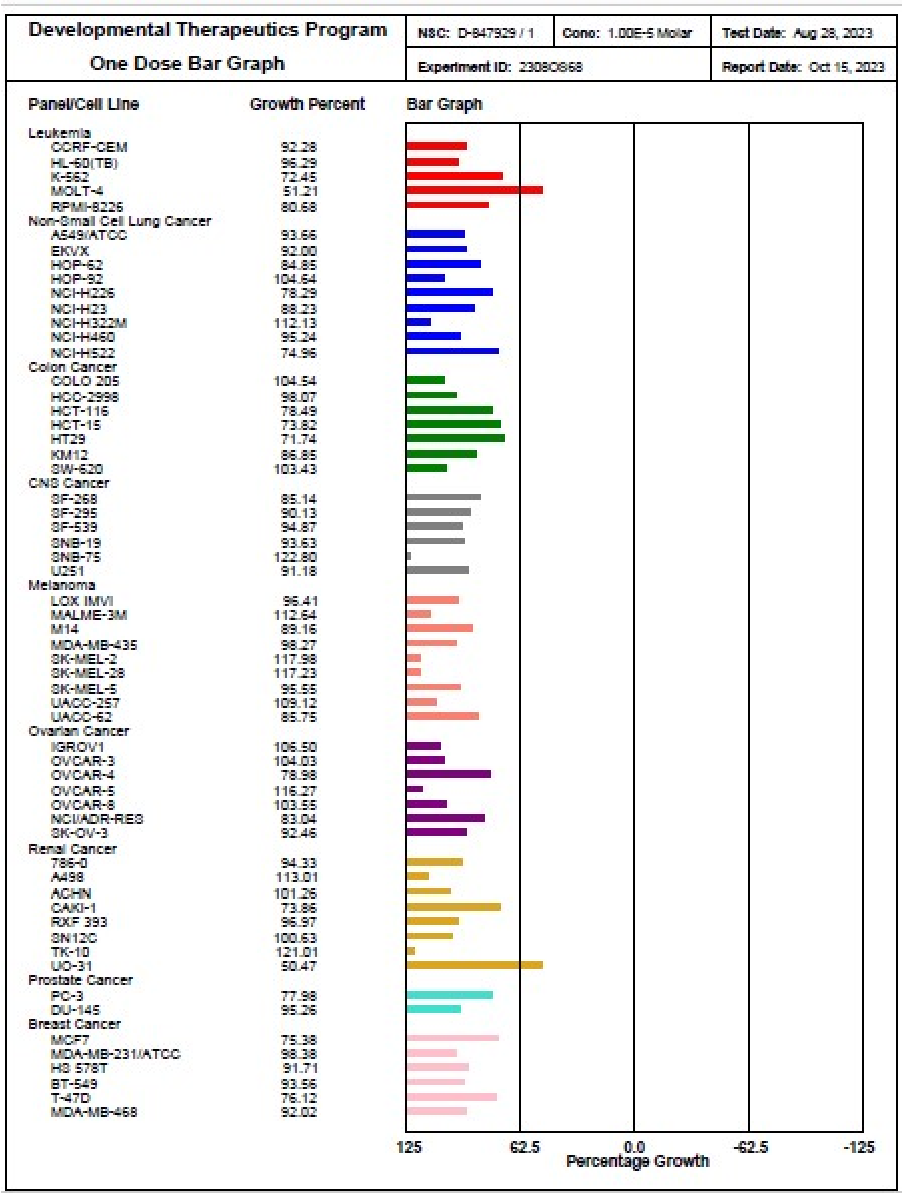

Fig. S86: ***In vitro*** **growth (G%) for the NCI60 cancer cell lines upon treatment with 10 µM of Compound 6d**

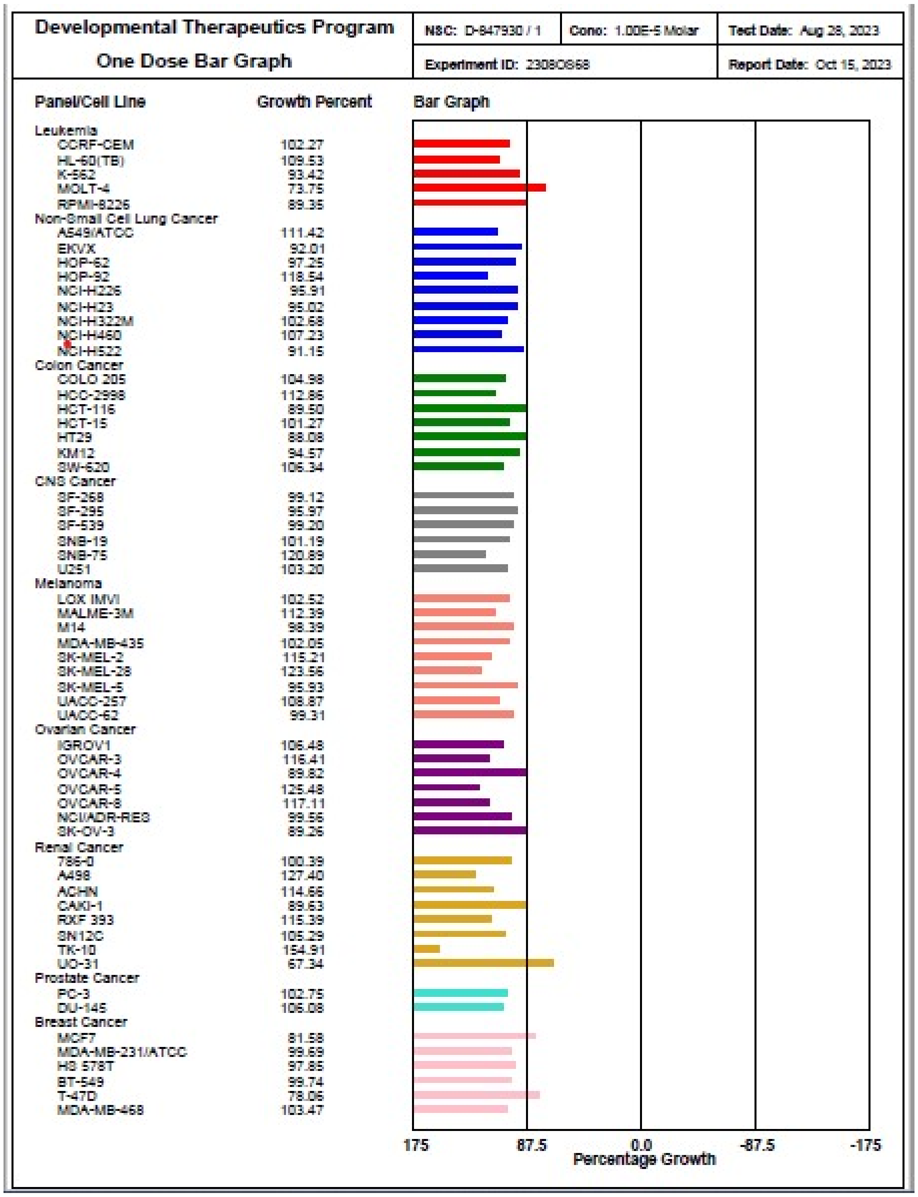

Fig. S87: ***In vitro*** **growth (G%) for the NCI60 cancer cell lines upon treatment with 10 µM of Compound 6e**

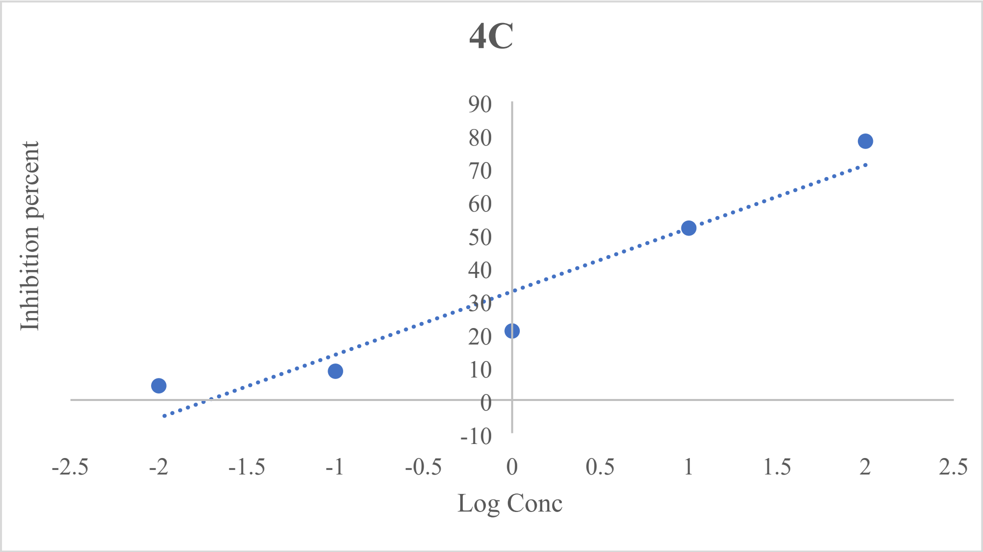

Fig. S88: A graph represent the tubulin polymerization assay of Compound 4C

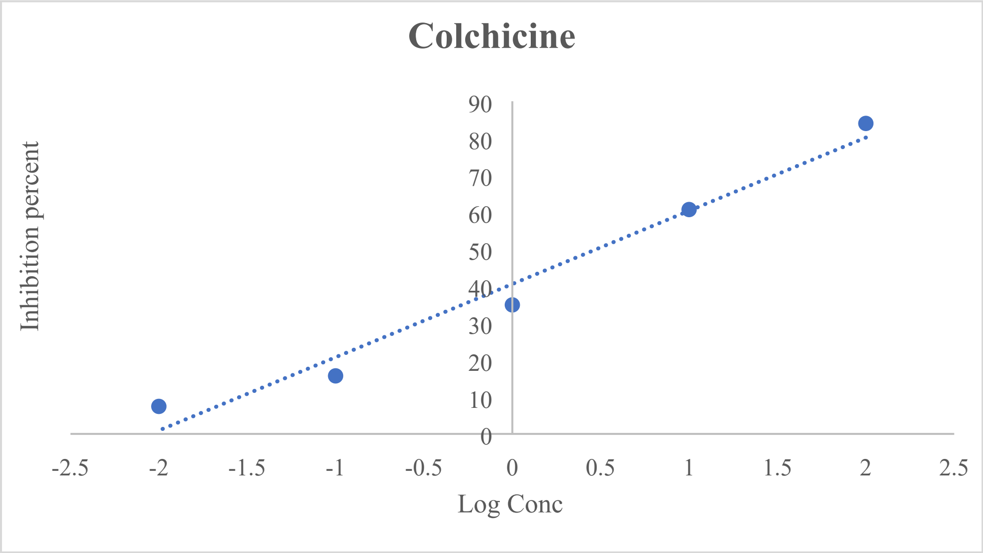

Fig. S89: A graph represent the tubulin polymerization assay of colchicine

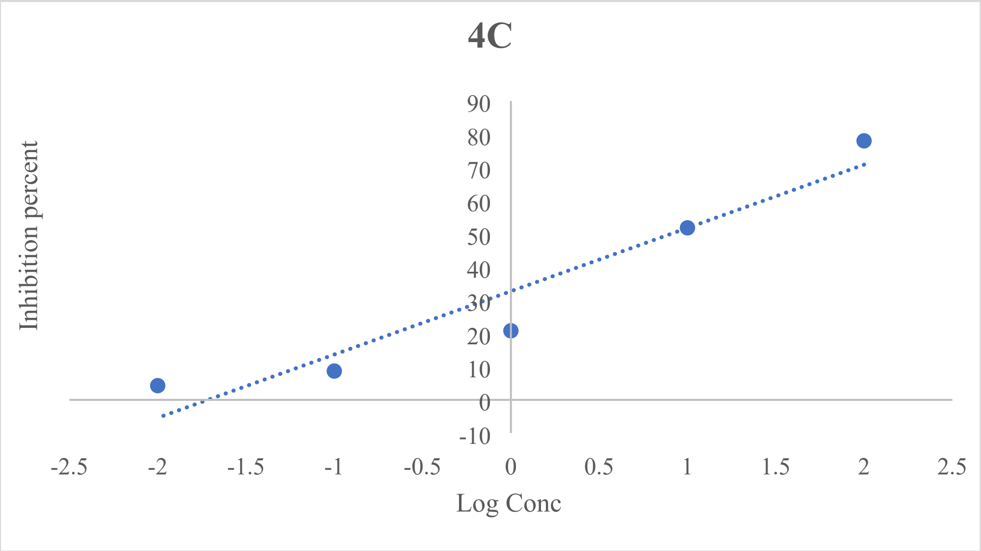

Fig. S90: A graph represent the tubulin polymerization assay of CA-4

Table S3: Average of percentages of cells at early apoptosis, late apoptosis, and necrosis of control non-treated cells, 4c, and colchicine-treated cells.

	Sample	Total Cell Death (%)	Early Apoptosis (%)	Late Apoptosis (%)	Necrosis (%)
1	**Control**	2.37 ± 1.56	0.52 ± 0.3	0.19 ± 0.08	1.66 ± 0.97
2	**4c**	29.15 ± 2.9	7.44 ± 1.59	17.1 ± 1.9	4.61 ± 2
3	**Colchicine**	37.22 ± 2.8	19.19 ± 0.87	6.62 ± 0.75	11.41 ± 2.8

Docking studies:

Table S4: Binding interactions, group of binding, type of interactions and interaction energies of Colchicine, 3b,c, 4c, and 5c into the colchicine binding site of tubulin enzyme.

Compound	Amino acids……..Interacted group	Type of interaction	Affinity (Kcal/mol)
Ligand (Colchicine)	Val 181…. Tetrahydrobenzoheptalene (C=O)	H-Bond	-9.8
	Val 238…. Trimethoxy phenyl	H-Bond	
	Cys 241….Trimethoxy phenyl	π-alkyl	

	Leu 242….Trimethoxy phenyl	π-alkyl	
	Leu 248…. Trimethoxy phenyl	π-sigma	
	Ala 250…. Trimethoxy phenyl	H-Bond	
	Asp 251…. Trimethoxy phenyl	H-Bond	
	Leu 255.... Trimethoxy phenyl	π-sigma	
	Met 259.… Tetrahydrobenzoheptalene	π-sulfur	
	Val 315…. Tetrahydrobenzoheptalene (OCH_3)	H-Bond	
	Ala 316….tetrahydrobenzoheptalene ring	π-alkyl	
	Ile 318…. Trimethoxy phenyl	π-alkyl	
	Asn 350…. Tetrahydrobenzoheptalene (OCH_3)	H-Bond	
	Lys 352…. tetrahydrobenzoheptalene	π-alkyl	
	Ile 378…. Trimethoxy phenyl	π-alkyl	
3b	Ala 180….dimethoxyphenyl ring	π-alkyl	-9.0
	Glu 183…. dimethoxyphenyl ring	H-bond	
	Ser 178…. dimethoxyphenyl ring	H-bond	
	Cys 241….chloroethylquinoline ring	π-alkyl	
	Leu 248…. dimethoxyphenyl ring, chloroethylquinoline ring	π- sigma	
	Ala 250…. chloroethylquinoline ring	π-alkyl	
	Leu 255…. chloroethylquinoline ring	π- sigma	
	Ala 316…. chloroethylquinoline ring	π-alkyl	
	Ile 318…. chloroethylquinoline ring	π-alkyl	
	Lys 352…. chloroethylquinoline ring	π-alkyl	
3c	Ala 180….trimethoxyphenyl ring	π-alkyl	-8.0
	Ser 178…. trimethoxyphenyl ring	H-bond	

	Cys 241….chloroethylquinoline ring	π-alkyl	
	Leu 248…. dimethoxyphenyl ring, chloroethylquinoline ring	π- sigma<br>π-alkyl	
	Ala 250…. chloroethylquinoline ring	π-alkyl	
	Lys 254…. trimethoxyphenyl ring	π- sigma	
	Leu 255…. chloroethylquinoline ring	van der waals	
	Ala 316…. chloroethylquinoline ring		
	Ile 318…. chloroethylquinoline ring	π-alkyl	
	Lys 352…. chloroethylquinoline ring		
	Ser 178…. Trimethoxyphenyl ring	H-bond	
	Thr 179…. 2-oxo-1,2-dihydropyridine ring	H-bond	
	Ala 180…. 2-oxo-1,2-dihydropyridine ring	H-bond	
	Tyr 224…. Trimethoxyphenyl ring	H-bond	
	Cys 241…. chloroethylquinoline ring	π-alkyl	
	Gln 247…. Trimethoxyphenyl ring	H-bond	
4c	Leu 248…. chloroethylquinoline ring	π- sigma	-11.5
	Ala 250…. chloroethylquinoline ring	π-alkyl	
	Leu 255…. chloroethylquinoline ring	π- sigma	
	Asn 258….2-oxo-1,2-dihydropyridine ring	H-bond	
	Ile 318…. chloroethylquinoline ring	π-alkyl	
	Lys 352….2-oxo-1,2-dihydropyridine ring	π-alkyl	
	Ala 180…. 2-thioxo-1,2-dihydropyridine ring, trimethoxy phenyl	π-alkyl<br>H-bond	
5c	Val 181….2-thioxo-1,2-dihydropyridine ring	H-bond	-10.9
	Tyr 224…. Trimethoxyphenyl ring	π-alkyl	

Cys 241…. chloroethylquinoline ring	H-bond
Gln 247…. Trimethoxyphenyl ring	π- sigma
Leu 248…. chloroethylquinoline ring, 2-thioxo-1,2-dihydropyridine ring	π-alkyl
Ala 250…. chloroethylquinoline ring	π- sigma
Leu 255…. chloroethylquinoline ring	π- alkyl
Ile 318…. chloroethylquinoline ring	π- alkyl
Lys 352….2-thioxo-1,2-dihydropyridine ring	
